# Bioinformatics analysis identifies potential hub genes and crucial pathways in the pathogenesis of asthenozoospermia

**DOI:** 10.1186/s12920-022-01407-5

**Published:** 2022-12-05

**Authors:** Ci Zou, Shen Xu, Hao Geng, Enlai Li, Wei Sun, Dexin Yu

**Affiliations:** 1 Department of Urology, The Second Affiliated Hospital of Anhui Medical of University, 230601 Hefei, China; 2 Department of Urology, The Second Affiliated Hospital of Anhui Medical University, NO.678 Furong Road, 230601 Hefei, China

**Keywords:** Asthenozoospermia, Bioinformatics analysis, Hub genes, Pathways, miRNAs

## Abstract

**Background:**

Asthenozoospermia is a troublesome disease experienced by men in their reproductive years, but its exact etiology remains unclear. To address this problem, this study aims to identify the hub genes and crucial pathways in asthenozoospermia.

**Methods:**

We screened two Gene Expression Omnibus (GEO) datasets (GSE92578 and GSE22331) to extract the differentially expressed genes (DEGs) between normozoospermic and asthenozoospermic men using the “Limma” package. Gene enrichment analyses of the DEGs were conducted using the “clusterProfiler” R package. The protein-protein interaction (PPI) network was then established using the STRING database. A miRNA-transcription factor-gene network was constructed based on the predicted results of hub genes using the RegNetwork database. The expression of four hub genes in asthenozoospermia and normal samples were verified using quantitative real-time reverse transcription-polymerase chain reaction (qRT-PCR) and western blotting.

**Results:**

We identified 271 DEGs, which included 218 upregulated and 53 downregulated in two asthenozoospermia datasets. These DEGs were observed to be markedly enriched in pathways with cell growth and embryonic organ development, phospholipase D signaling pathway, cGMP-PKG signaling pathway, and Wnt signaling pathway. The most significant genes were identified, including COPS7A, CUL3, KLHL7, NEDD4. We then constructed regulatory networks of these genes, miRNAs, and transcription factors. Finally, we found that the COPS7A was significantly upregulated in patients with asthenozoospermia, but CUL3, KLHL7 and NEDD4 were significantly downregulated compared with normal samples.

**Conclusion:**

We applied bioinformatics methods to analyze the DEGs of asthenozoospermia based on the GEO database and identified the novel crucial genes and pathways in this disease. Our findings may provide novel insights into asthenozoospermia and identify new clues for the potential treatment of this disease.

**Supplementary Information:**

The online version contains supplementary material available at 10.1186/s12920-022-01407-5.

## Introduction

Male infertility has become a primary human health concern due to its increasing incidence and unknown etiology[1]. This disease not only introduces great psychological pressure and a substantial economic burden to the patients but also affects their overall health[2]. Asthenozoospermia accounts for a large proportion of male infertility patients, and its main clinical manifestations are decreased sperm motility. Based on standards established by the World Health Organization (WHO), asthenozoospermia is characterized by a total sperm motility lower than 40% and a progressive sperm motility lower than 32% in a semen sample[3]. The process of spermatogenesis depends on the accurate regulation of key genes, and improper regulation of this process leads to abnormalities in spermatogenesis[4]. Several factors, including smoking, drugs, chromosomal abnormalities, or mutations, are associated with asthenozoospermia, but the etiology of this disease remains unclear[5]. Several studies have found high mRNA levels in human testicular tissue and sperm, and abnormal mRNA expression is closely related to poor sperm quality[6, 7]. Although great progress toward elucidating the etiology of male infertility has been achieved, the information on the pathogenesis of asthenozoospermia remains scarce, and a large number of genes with unknown functions need further study.

With advancements in microarray and high-throughput technology, bioinformatics analyses have recently been performed to understand the molecular mechanism of asthenozoospermia. Abnormalities at the genomic level, including genes and signaling pathways related to this disease, have increasingly been identified between fertile men and patients with asthenozoospermia[8–12]. The large amount of gene sequencing data available has provided a convenient resource for analyzing the regulation of gene expression in asthenozoospermia using increasingly complex analytical techniques[13]. In the current manuscript, we obtained differentially expressed genes (DEGs) in patients with asthenozoospermia using the GSE92578 and GSE22331 datasets from the Gene Expression Omnibus (GEO) database. Subsequently, we conducted a series of bioinformatics analyses to identify crucial genes and pathways involved in spermatogenesis. Our findings indicated the newly discovered crucial genes and key pathways may contribute to finding the new therapeutic targets and approaches for this disease.

## Methods

### Sample preparation and semen collection

After obtaining informed consent from these patients and healthy volunteers, we collected 30 healthy young men and 30 patients diagnosed with asthenozoospermia in our hospital from November 2021 to January 2022. Healthy volunteers with varicocele, reproductive tract infection, and abnormal sperm parameters were excluded. All subjects were Han Chinese with no genetic association. Three ml semen samples obtained through masturbation were analyzed after three to five days of abstinence. Asthenozoospermia is defined as progressive sperm motility lower than 32% in a semen sample, which is confirmed by routine semen analysis of three semen samples collected at different time points[14]. Semen samples containing the following parameters are considered normal: semen volume ≥ 2 mL, sperm concentration ≥ 15 × 10^6^/mL, pH ≥ 7.2, progressive motility (PR) ≥ 32%, PR + Non-progressive motility (NP) ≥ 40%. All semen samples were collected and processed by the guidelines of the World Health Organization (WHO) Laboratory Manual on Human Semen Examination and Processing (5th Edition 2010).

### RNA extraction and quantitative real-time polymerase chain reaction (qRT‐PCR) analysis

According to a previous report, the method of TRIzol (Invitrogen) extracted RNA from sperm cells [15]and then, reverse transcribed it into cDNA based on a reverse transcription kit (Novabio, China). Subsequently, experiments were performed in an SYBR qPCR Mix kit (Novabio, China) protocol using glyceraldehyde-3-phosphate dehydrogenase (GAPDH) expression as a reference using the CFX96 system (Bio-Rad Laboratories, Inc.). Real-time RT-PCR was carried out at 95 °C for the 30s, then at 95 °C for 10s, with 40 cycles, annealing, and extension at 60 °C for 30s. All samples were normalized according to GAPDH, and the oligonucleotide primers for five genes are provided in Table 1. The relative expression of these genes was measured using the 2 ^ΔΔ^ Ct method[16].

**Table 1:** Primer sequences used for qRT-PCR amplification

Gene	Forward primer (5’→3’)	Reverse primer (5’→3’)
COPS7A	CCACACTCATCCATCAGGTGCT	CGGAAGGTAGAGGCAAAGTCAC
CUL3	TCGACAGCTCACACTCCAGCAT	GTGCTTCCGTGTATTAGAGCCAG
KLHL7	TTGTGTGACGTGATCCTCATGG	TATCAGGTTCAGCATCTTTGAGTTC
NEDD4	CAGAAGAGGCAGCTTACAAGCC	CTTCCCAACCTGGTGGTAATCC
GAPDH	GTCTCCTCTGACTTCAACAGCG	ACCACCCTGTTGCTGTAGCCAA
qRT-PCR, quantitative real‐time polymerase chain reaction.		

### Sperm protein extraction and western blotting

The sperm cells in the semen sample was isolated and subsequently processed according to the previously method[17]. The samples were separated by SDS-PAGE in a 10% polyacrylamide gel and transferred to PVDF membranes. After the PVDF membrane was blocked, anti-COPS7A (cat. no.DF8375; 1:1000; Affinity Biosciences), anti-CUL3 (cat. no. DF6223; 1:1000; Affinity Biosciences), anti-KLHL7 (cat. no. A7817; 1:1000; ABclonal Technology), anti-NEDD4 (cat. no.AF4636; 1:1000; Affinity Biosciences), and anti-GAPDH (cat. no.AF7021; 1:1000; Affinity Biosciences) antibodies were incubated separately. During blotting, the blots were cut prior to hybridisation with antibodies based on the molecular weight. It was subsequently incubated with HRP-coupled goat anti-rabbit IgG (cat. no. S0001; 1:10,000;Affinity Biosciences) and goat anti-mouse IgG (cat. no. S0002; 1:10,000;Affinity Biosciences) secondary antibodies. The reaction band is detect by enhanced chemiluminescence (ECL kit, Thermo Fisher Scientific).

## Data collection

In our study, two asthenozoospermia datasets were selected from the GEO database based on previous literature reports, and these data proved to be reliable[13, 15]. Two asthenozoospermia GSE profiles include GSE92578 (GPL20301 Illumina HiSeq 4000 [Homo sapiens]), containing 3 asthenozoospermia sample and 4 normal sample[9], and GSE22331 (GPL570 [HG-U133_Plus_2] Affymetrix Human Genome U133 Plus 2.0 Array), containing one asthenozoospermia and normal sample[12]. To identify hub genes in asthenozoospermia, we filtered top genes among the DEGs based on protein-protein interaction (PPI) networks and Friends analyses.

## Data processing of DEGs

**Fig. 1:**
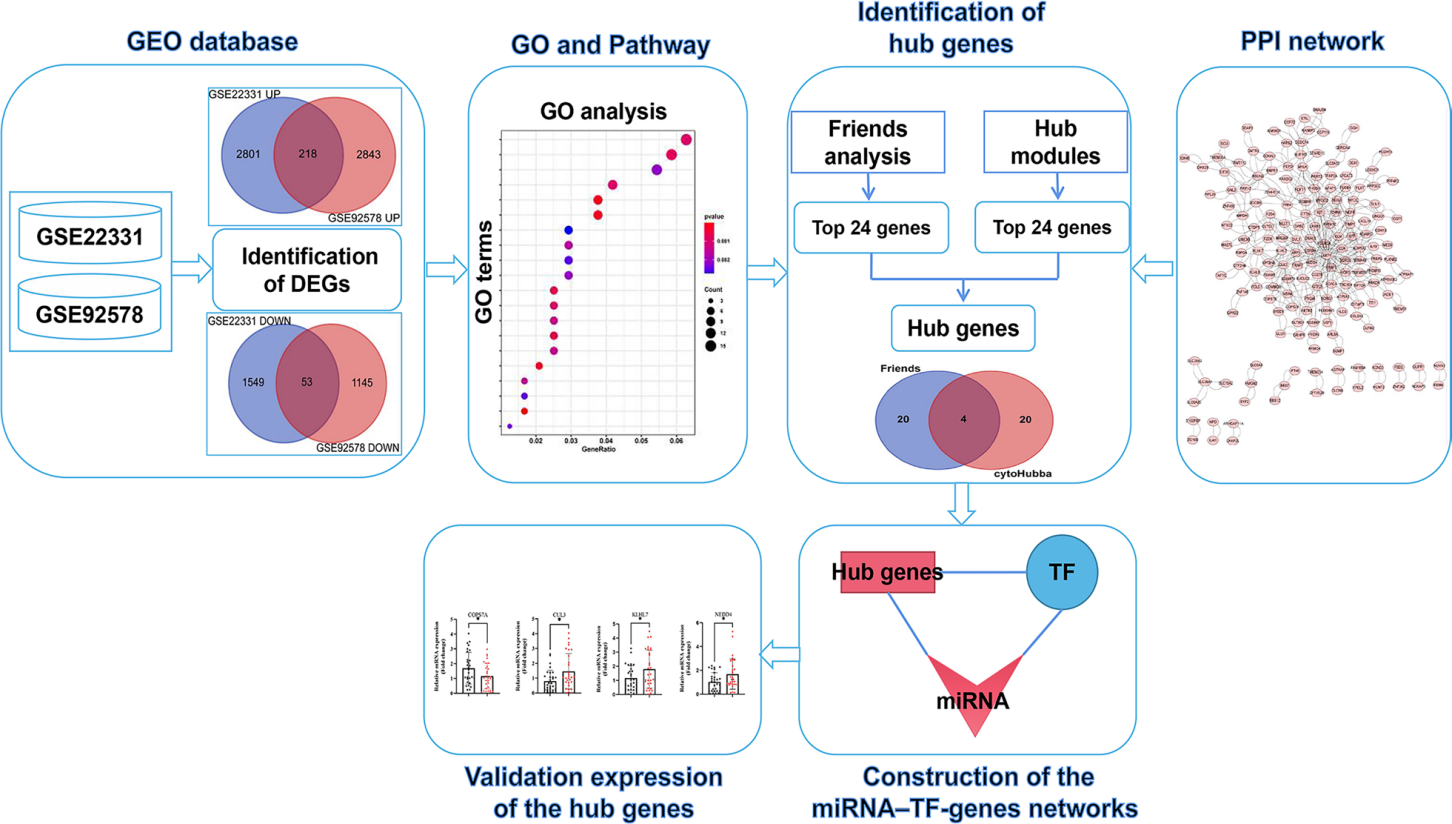
Study design and data preprocessing

The process used in this study is shown in Fig. 1. From the GSE22331 dataset, DEGs were screened using R software (v 4.1.1) based on the “Limma” package based on preset thresholds of |logFC| > 1.0 and P-value < 0.05[18]. The DEG results from the GSE92578 dataset were downloaded from the supplementary material of a previously published study (https://academic.oup.com/biolreprod/article/100/4/982/5230853#134234708)[9]. DEGs were defined as genes that met the criteria Q-value < 0.001 and |logFC | > 1.0 in the GSE92578 dataset. Intersecting genes were obtained by Venn analyses[19]. A visual hierarchical cluster analysis was also conducted to display a volcano plot of the DEGs.

## GO annotation and KEGG pathway enrichment analyses of DEGs

We performed GO annotation and KEGG pathway of DEGs[20–22]. The GO annotation (including biological processes, cellular components, and molecular functions) and KEGG pathway (*P-*value < 0.05) were enriched using the “clusterProfiler” R language package[23].

## Top genes based on the semantic similarities of GO terms

Subsequently, we analyzed the functional similarity between the common DEGs using the “GOSemSim” package[24], which analyses the semantic similarity of GO terms obtained for the genes (also called Friends analysis). The top 24 DEGs with high average functional similarity were considered hub genes in the Friends analysis.

## Construction of PPI network and screening of hub genes

To explore the correlation among DEGs, the PPI network was built by the STRING database (https://www.string-db.org/). We then visualized the results using Cytoscape software (v 3.7.2), and the top 24 DEGs were identified using the cytoHubba based on node degree[25]. To identify hub genes in asthenozoospermia, we filtered top genes among the DEGs based on protein-protein interaction (PPI) networks and Friends analyses.

## Construction of the miRNA-TFs-genes network

To study the regulatory mechanisms of four crucial genes, miRNAs and TFs that bind to hub genes were predicted using RegNetwork (http://www.regnetworkweb.org/), which is a target gene prediction database. We then constructed regulatory networks of these hub genes, including TF-miRNA-gene regulatory networks, using Cytoscape software (v 3.7.2).

### Statistical analysis

Experimental data were reported by x ± SD, and the differences among the experimental groups were compared by *Student’s t-*test. All experimental data analysis was performed using SPSS 23.0 (IBM Corp.), and P < 0.05 indicated statistical significance.

## Results

### DEGs in asthenozoospermia

**Fig. 2:**
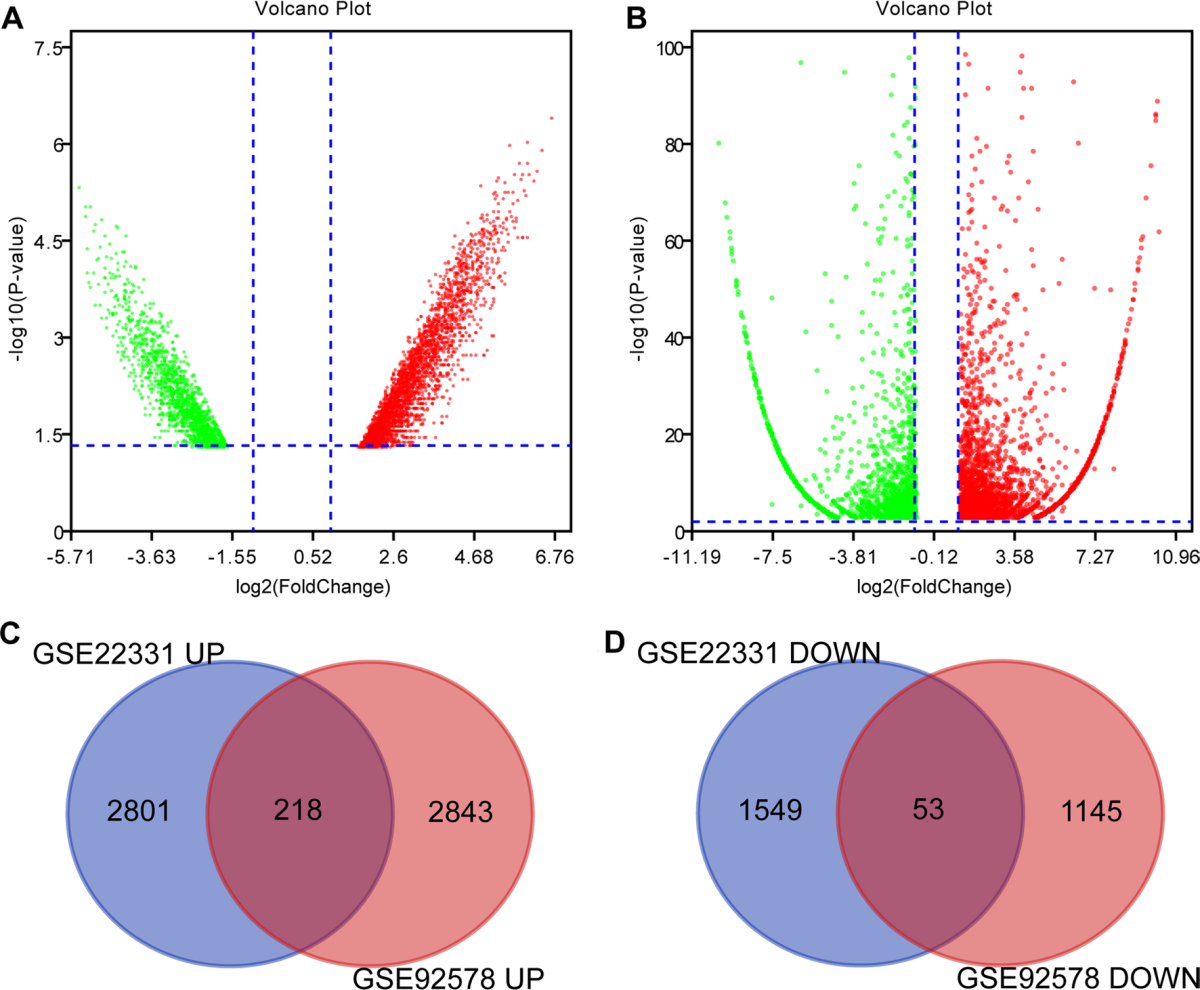
Identification of common DEGs from GSE22331 and GSE92578 datasets. **A** Volcano plot of the 4,621 DEGs in GSE22331 dataset. **B** Volcano plot of the 4,259 DEGs in GSE92578 dataset.Venn diagram of **C** upregulated and **D** downregulated DEGs based on the two GEO datasets. Red, upregulation; green, downregulation. DEG, differentially expressed gene; GEO, Gene Expression Omnibus

The analysis of the GSE22331 dataset successfully identified 4,621 DEGs, which included 3,019 upregulated and 1,602 downregulated genes (Additional file 1: Tables S1 and S2). The DEGs identified from the GSE22331 database are shown in Fig. 2 A, where the red and green dots represent the up-and down-regulated DEGs, respectively. From the GSE92578 dataset, we identified 4,259 DEGs, which included 3,061 upregulated and 1,198 downregulated genes (Additional file 1: Tables S3 and S4), and the DEGs identified from the GSE92578 database are shown in Fig. 2B. Venn analysis was then performed to compare the DEGs identified from the GSE22331 dataset with those obtained from the GSE92578 dataset. In this study, we identified 271 DEGs from two datasets involving 218 up-regulated and 53 down-regulated genes (Fig. 2 C and Fig. 2D; Additional file 1: Tables S5 and S6).

**Table 2:** Top 24 genes based on the Friends analysis and PPI network analysis

Rank	Friends analysis	PPI network analysis	Intersecting genes
1	ZNF490	AKT1	-
2	ZNF17	COMMD9	-
3	SAP30BP	TIMP1	-
4	ZNF783	ADIPOQ	-
5	COPS7B	CD4	-
6	COPS7A	COPS7A	COPS7A
7	ZNF415	ELN	-
8	MLLT1	GNL2	-
9	DAB2	KIT	-
10	MRGBP	DVL1	-
11	TTI1	ACACA	-
12	OLFM2	KLHL2	-
13	TRAF3	UCP3	-
14	CUL3	CUL3	CUL3
15	JADE1	SUCLG2	-
16	CTNNBIP1	RRP12	-
17	STRN4	KLHL8	-
18	PRKCH	NOS3	-
19	NEDD4	NEDD4	NEDD4
20	RBM4	CTTN	-
21	GLO1	ARF1	-
22	MIA3	MYLK	-
23	HDX	NSUN2	-
24	KLHL7	KLHL7	KLHL7
PPI, protein-protein interaction.			

**Table 3:** The basic characteristics and sperm parameters in patients with asthenozoospermia and healthy volunteers

Variables	Asthenozoospermia (n = 30)	Normozoospermia (n = 30)	*P-*value
Age (years)	30.40 ± 6.91	29.03 ± 5.34	0.395
BMI (kg/m^2)^	22.19 ± 1.60	21.94 ± 1.56	0.548
Smoking			0.584
Yes	21 (70.00%)	19 (63.33%)	
No	9 (30.00%)	11 (63.33%)	
Drinking			0.605
Yes	15 (50.00%)	13 (43.33%)	
No	15 (50.00%)	17(56.67%)	
Sperm volume (ml)	3.06 ± 0.38	3.84 ± 0.79	< 0.01
pH	7.70 ± 0.08	7.71 ± 0.07	0.799
Sperm density (×10^6^/ml)	11.53 ± 6.78	55.38 ± 10.55	< 0.01
PR	0.10 ± 0.04	0.48 ± 0.033	< 0.01
NP	0.001 ± 0.004	0.11 ± 0.028	< 0.01
IM	0.91 ± 0.01	0.30 ± 0.04	< 0.01
Sperm activity rate	0.11 ± 0.04	0.60 ± 0.03	< 0.01
BMI, body mass index; IM, inactive sperm rate; NP, non-progressive motility; PR, progressive motility.			

## GO annotation and KEGG pathway enrichment analysis

Functional enrichment analysis was performed on two datasets from the GEO database, respectively. In the GSE22331 dataset, the crucial biological functions were mainly enriched in related processes of cell signal transduction (Additional file 1: Fig. S1A-C), and the crucial pathways were mainly enriched in Cytokine-cytokine receptor interaction, Herpes simplex virus 1 infection, Neuroactive ligand-eceptor interaction, Toll − like receptor signaling pathway, and Viral protein interaction with cytokine and cytokine receptor (Additional file 1: Fig.S1D-E). In the GSE92587 dataset, the crucial biological functions were mainly enriched in cell growth and signal transduction (Additional file 1: Fig. S2A-C), and the crucial pathways were mainly enriched in Axon guidance, Focal adhesion, JAK − STAT signaling pathway, PD − L1 expression and PD − 1 checkpoint pathway in cancer, Ubiquitin mediated proteolysis (Additional file 1: Fig. S1D-E).

**Fig. 3:**
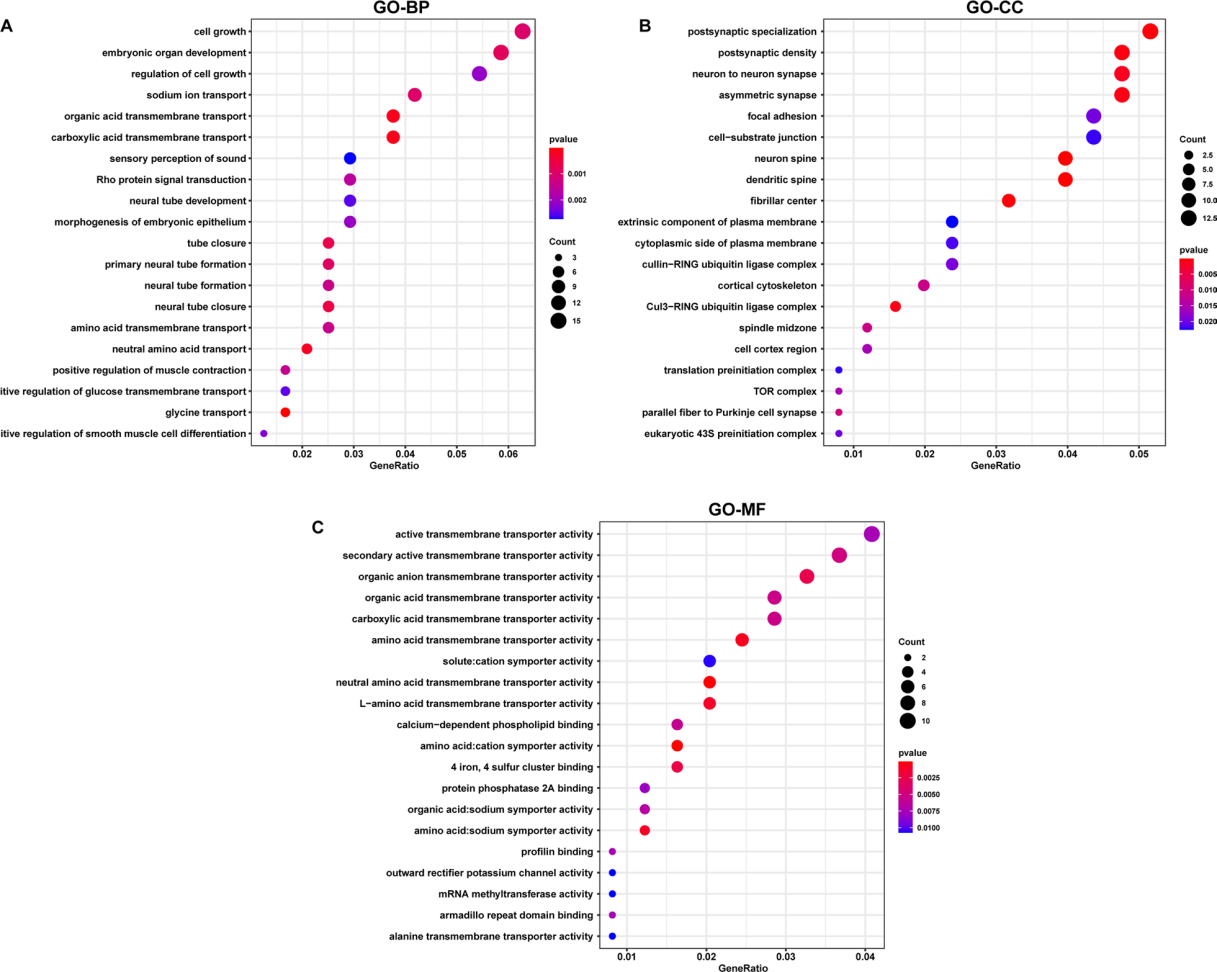
GO annotation enrichment analysis of DEGs. The top 20 enriched GO **A** BP, **B** CC and **C** MF terms. GO, gene ontology; DEG, differentially expressed gene; BP, biological process; CC, cellular component; MF, molecular function

**Fig. 4:**
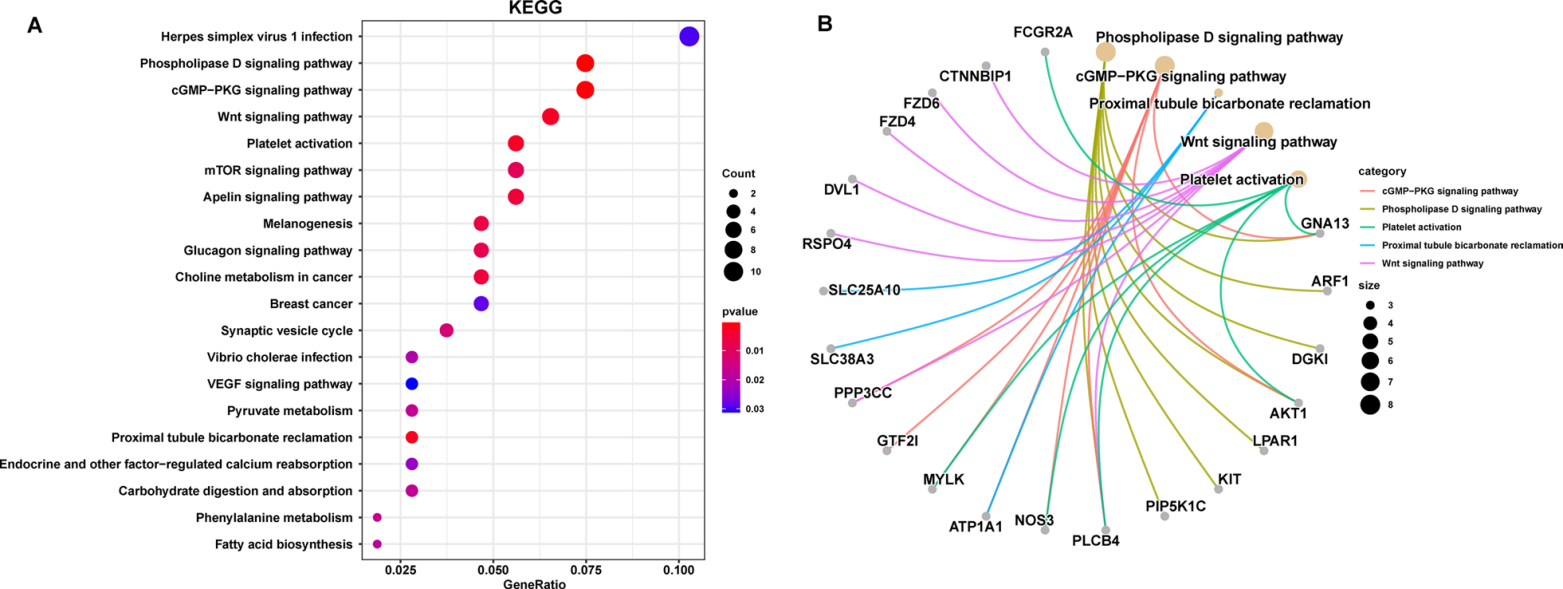
KEGG pathway enrichment analysis of DEGs. **A** The top 20 enriched KEGG pathways. **B** The underlying connection between DEGs and pathways in the top 5 KEGG pathway. KEGG, Kyoto encyclopedia of genes and genomes; DEG, differentially expressed gene

To clarify the mechanism of these DEGs in the pathogenesis of asthenozoospermia, the biological roles of the 271 DEGs were enriched by GO and KEGG pathway. We present the top 20 significant terms of GO and pathways. The crucial biological processes of these DEGs were mainly enriched in cell growth, embryonic organ development, regulation of cell growth, sodium ion transport, organic acid transmembrane transport, and carboxylic acid transmembrane transport (Fig. 3 A**)**. The cellular components analysis of these DEGs were significantly enriched in postsynaptic specialization, postsynaptic density, neuron-to-neuron synapse, asymmetric synapse, focal adhesion, cell-substrate junction, neuron spine, dendritic spine, and fibrillar center (Fig. 3B). These DEGs were significantly enriched in the following molecular functions: active transmembrane transporter activity, secondary active transmembrane transporter activity, organic anion transmembrane transporter activity, organic acid transmembrane transporter activity, carboxylic acid transmembrane transporter activity, and amino acid transmembrane transporter activity (Fig. 3 C). In addition, the pathways in which the DEGs were significantly enriched were the phospholipase D signaling pathway, cGMP-PKG signaling pathway, Wnt signaling pathway, mTOR signaling pathway, apelin signaling pathway, glucagon signaling pathway, and VEGF signaling pathway (Fig. 4 A). We also identified the underlying connection between the DEGs and the top 5 KEGG pathways, namely, herpes simplex virus 1 infection, phospholipase D signaling pathway, cGMP-PKG signaling pathway, Wnt signaling pathway, and platelet activation (Fig. 4B).

### Identification of top genes based on the Friends analysis

**Fig. 5:**
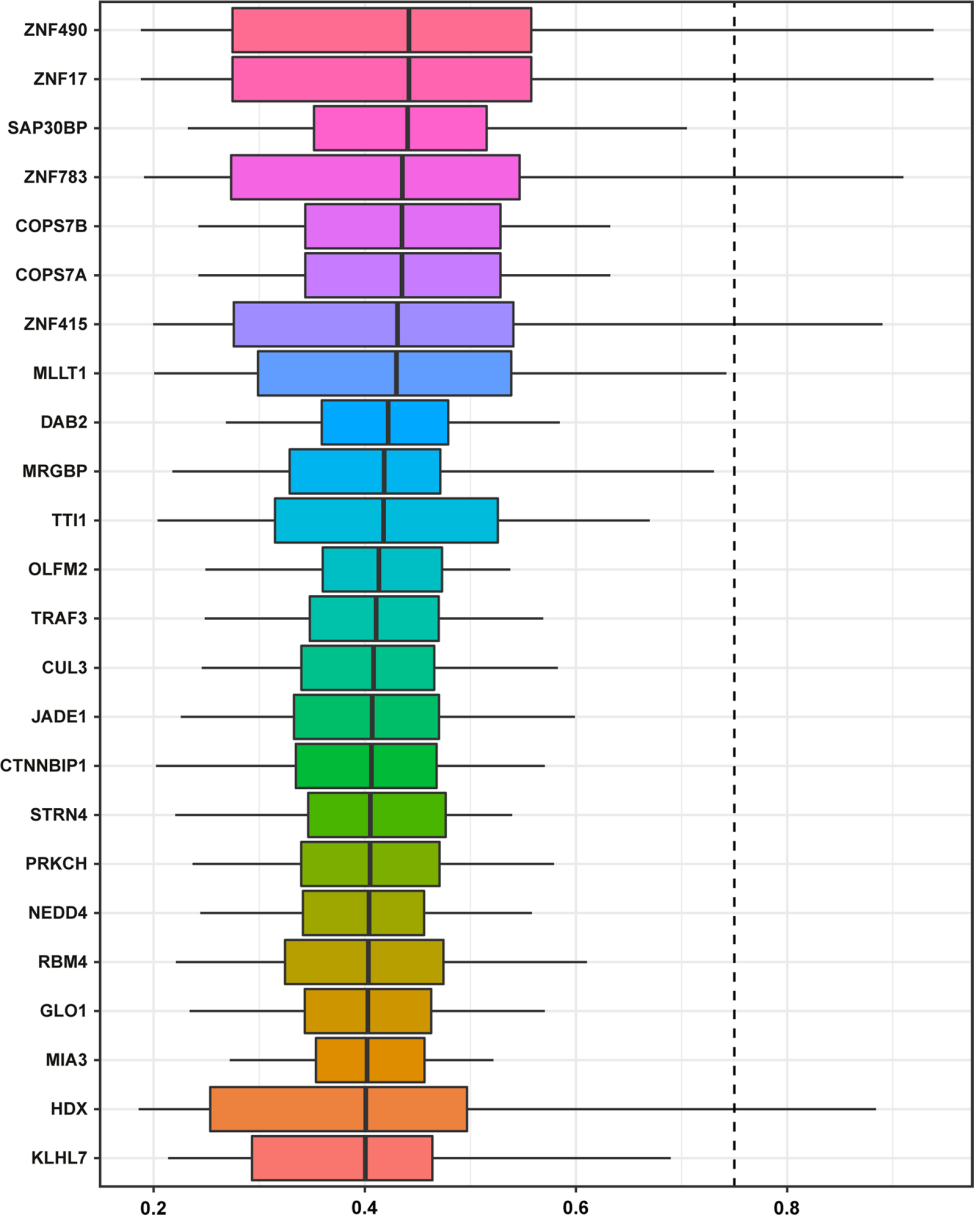
Summary of functional similarities of the interactome in asthenozoospermia. The box plot represents the middle 50% similarity (the upper and lower boundaries represent the 75th and 25th percentiles). The lines in the boxes represent the average of the functional similarities. Proteins with higher mean functional similarities (cutoff > 0.75) are considered to be central proteins in interacting groups in asthenospermia. The dashed line indicates the cutoff value

To identify interacting proteins in asthenozoospermia, proteins were ranked according to the average functional similarity relationship between proteins in the interactoms. The distribution of the functional similarities is summarized as box plots. The Friends analysis results showed that the top 24 genes were ZNF490, ZNF17, SAP30BP, ZNF783, COPS7B, COPS7A, ZNF415, MLLT1, DAB2, MRGBP, TTI1, OLFM2, TRAF3, CUL3, JADE1, CTNNBIP1, STRN4, PRKCH, NEDD4, RBM4, GLO1, MIA3, HDX, and KLHL (Fig. 5).

## Construction of protein-protein interaction (PPI) network and screening of hub genes

**Fig. 6:**
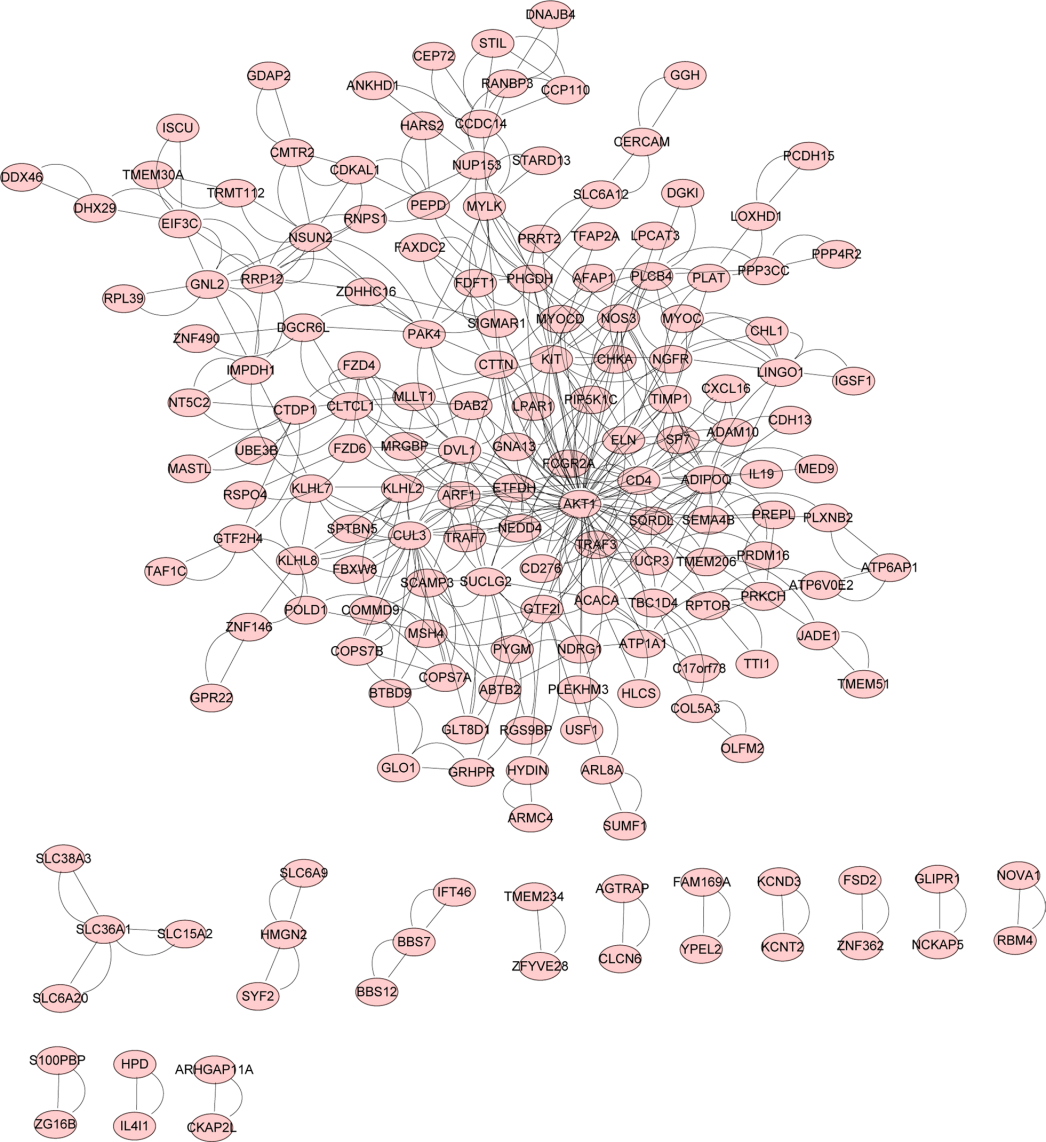
PPI network was constructed by all the 271 DEGs using STRING database. PPI, protein-protein interaction; DEG, differentially expressed gene; STRING, search tool for the retrieval of interacting genes

**Fig. 7:**
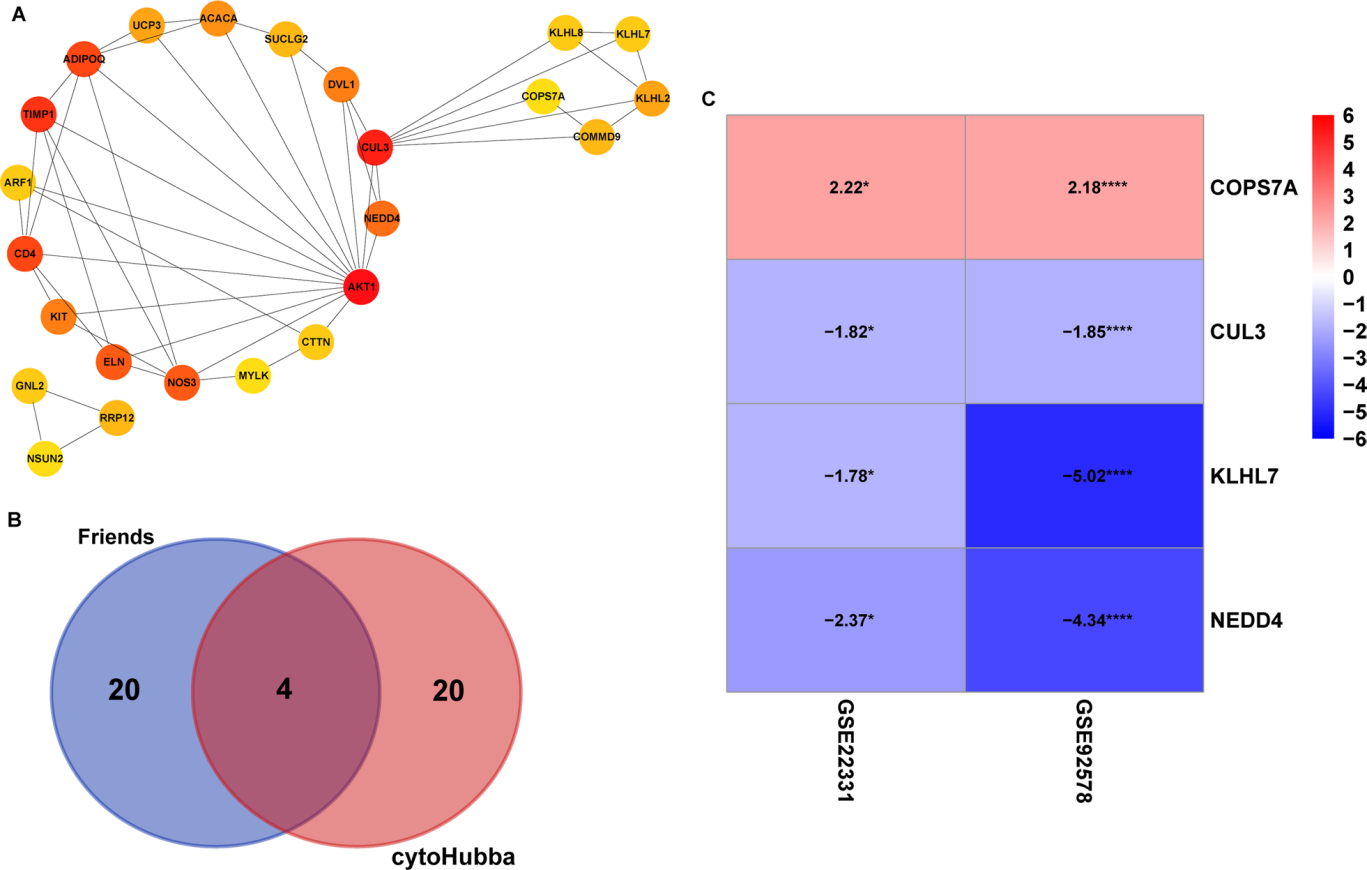
The hub genes identification. **A** Module analysis of PPI network. **B** The 4 common hub genes between the PPI network and Friends analysis were screened. **C** The heat map showed the expression of four hub genes in the GSE22331 and GSE92578 datasets. PPI, protein-protein interaction.*P < 0.05, ****P < 0.001

To understand the interaction between these DEGs, we performed PPI network analysis to screen of hub genes in asthenozoospermia. The PPI network included 175 nodes and 490 edges (Fig. 6 and Table S7 ). Subsequently, the most valuable module was identified from the PPI network using MCODE, and the top 24 genes were screened by maximal clique centrality algorithm (MCC) in the cytoHubba **(**Fig. 7 A). A Venn analysis was used to identify four key genes based on two methods, including COPS7A, CUL3, KLHL7, NEDD4, which may be potentially important genes in asthenozoospermia (Fig. 7B; Table 2). The mRNA expression of COPS7A was upregulated in patients with asthenozoospermia, but the expression of CUL3, KLHL7, and NEDD4 was downregulated in these patients (Fig. 7 C).

## Construction of the miRNA-TF-genes network

**Fig. 8:**
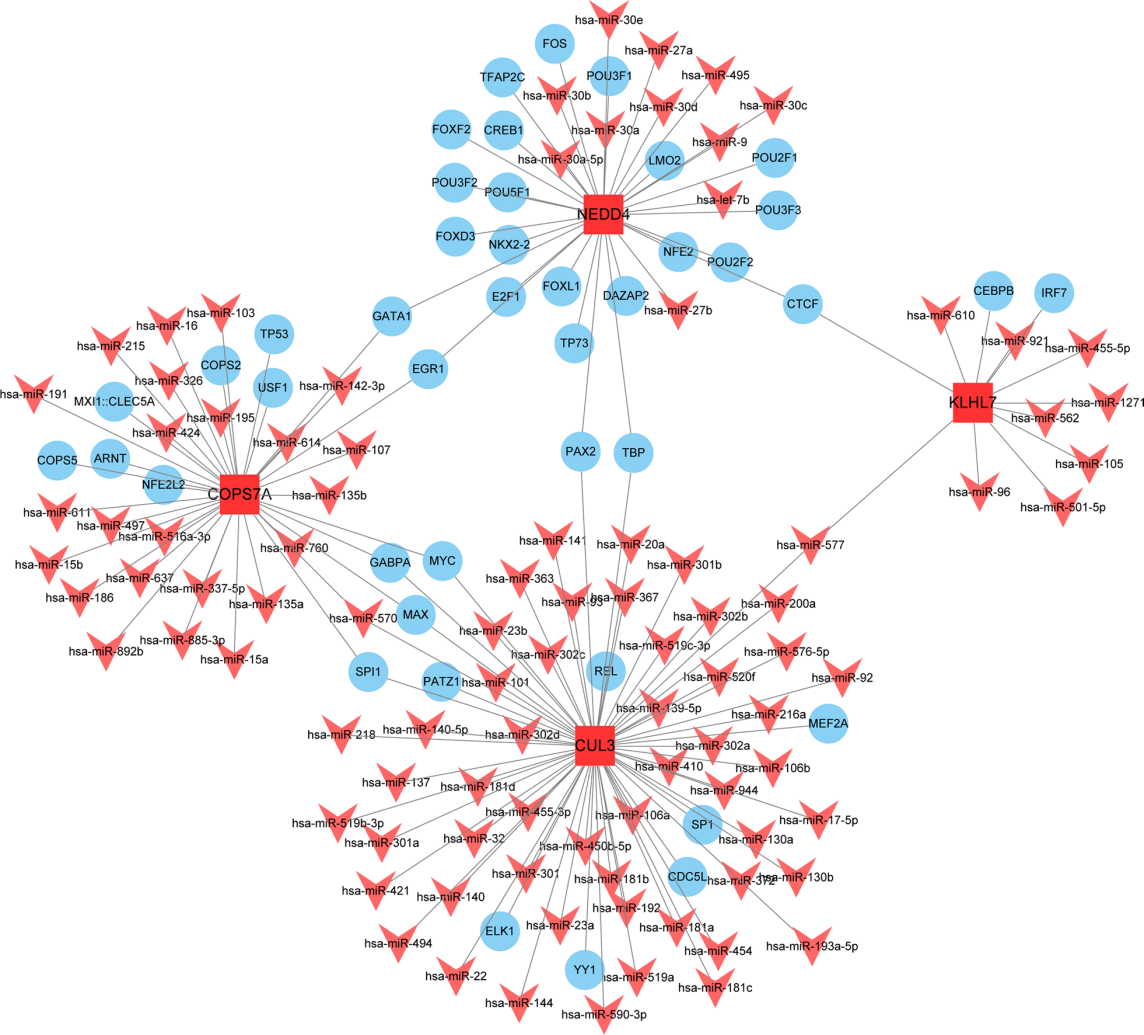
The miRNAs-TFs-genes networks. microRNAs, miRNAs; TFs, transcription factor

Then, we predicted miRNAs and transcription factors (TFs) upstream of the four hub genes in the RegNetwork database. Regulatory networks of the four hub genes were subsequently constructed using Cytoscape (Fig. 8 and Additional file 1: Table S8). In this network, miR-570 and four TF (SPI1, MAX, GABPA, and MYC), were verified to be coregulators of COPS7A and CUL3, and miR-577 was confirmed as a coregulator of CUL3 and KLHL7. In addition, the TF of CTCF was identified as a coregulator of KLHL7 and NEDD4, the TF of PAX2 and TBP were proven to be coregulators of NEDD4 and CUL3, and the TF of GATA1 and EGR1 were found to be coregulators of COPS7A and NEDD4.

**Validation expression of the hub genes by qRT-PCR and western blotting**.

**Fig. 9:**
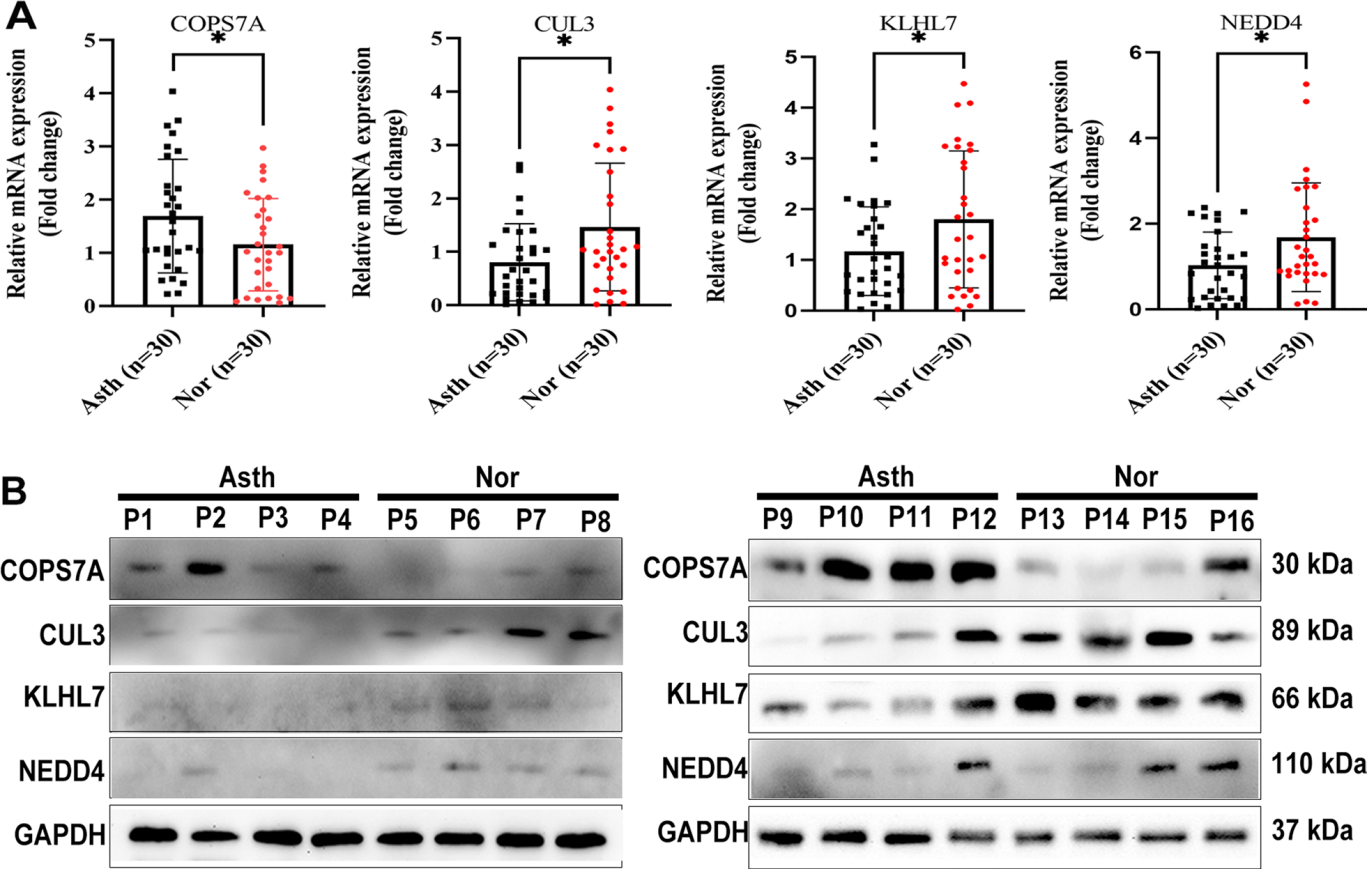
Hub genes expression in normozoospermia and asthenozoospermia samples **A** Expressions of four hub genes were validated by qRT-PCR in spermatozoa from normozoospermia and asthenozoospermia. **B** Expressions of four hub genes were validated by western blotting experiments in spermatozoa from normozoospermia and asthenozoospermia. *P < 0.05. Asth, asthenozoospermia; Noth, normozoospermia

The basic characteristics and sperm parameters from asthenozoospermia and normal sample were listed in Table 3. Expressions of four hub genes were validated by qRT-PCR in asthenozoospermia and normal samples, which was consistent with bioinformatical results (Fig. 9 A). A western blotting assay using 16 semen samples (including 8 asthenospermia and 8 normal samples) revealed similar results, indicating COPS7A was significantly upregulated in patients with asthenozoospermia, but CUL3, KLHL7 and NEDD4 were significantly downregulated compared with normal samples (Fig. 9B and Additional file 1: Fig. S3).

## Discussion

Approximately 15% of human couples worldwide are reportedly infertile, and more than half of these cases have been linked to male infertility[26]. Clinically, the main manifestation of this disease is poor sperm motility. For example, sperm functional or structural defects and seminal plasma abnormalities may impair sperm motility[1]. Therefore, exploring the molecular mechanisms of asthenozoospermia will help us identify breakthrough treatments for male infertility. In this study, we screened novel hub genes and crucial pathways in asthenozoospermia by comprehensive bioinformatics analysis.

Screening DEGs in patients with asthenozoospermia can be used to discover the pathogenesis of this disease. These DEGs are related to various molecular functions, especially the up-regulated DEGs, including cell growth, embryonic organ development, metabolism and transportation. Our results describe a general trend to up-regulation of the DEGs in asthenozoospermia, but previous investigations concur that asthenozoospermia is mostly characterized by gene down-regulation[8, 11, 15]. The differences may be associated with different data processing and diverse molecular functions. Together, these results suggest that dysregulation of key gene expression is an important pathogenesis of asthenozoospermia, as these genes are essential for sperm growth and development and energy metabolism. Sperm capacitation depends on a series of biochemical processes that occur in the female reproductive tract, which include the activation or inhibition of key signaling pathways[27]. Activation of the phospholipase D signaling pathway is thought to promote sperm capacitation[28]. A C-type natriuretic peptide secreted by the female reproductive tract may stimulate cGMP/PKG signaling in sperm cells, promote sperm activation, and induce acrosome reactions[29]. The Wnt signaling pathway has been proved to be involved in the regulation of GSK3 activity in sperm and affects sperm activity[30]. The Wnt signaling pathway has been proved to be involved in the regulation of GSK3 activity in sperm and affects sperm activity[31]. Although it has been reported that the Apelin signaling pathway, Glucagon signaling pathway, and VEGF signaling pathway are involved in the cell energy metabolism, proliferation, and apoptosis, their role in sperm function is unclear[32–34]. Studies have shown that sperm motility is regulated by the flagellum[35]. Defects in the function or structure of the flagellum are associated with abnormal energy metabolism and impairments in epididymal maturation and capacitation, and these effects lead to a decline in sperm motility and asthenozoospermia[36]. Taken together, our findings suggest that there is a potential correlation between these abnormal pathways and asthenozoospermia.

Sperm are highly functional cells that exhibit complex functions during reproduction[37]. The orderly expression of key genes during sperm development is crucial, and abnormal gene expression can lead to poor sperm quality or functional defects[36, 38, 39]. Moreover, the mRNA expression detected in testicular tissue was found to be consistent with that detected in sperm cells, which suggests that sperm mRNA may have potential roles to predict spermatogenesis and elucidate sperm function[7]. In this study, we studied mRNAs that exhibit significant dysregulation in patients with asthenozoospermia to explore the potential pathogenesis of this disease. In this manuscript, we identified novel key genes in asthenozoospermia based on two different algorithms. The intersection among two groups of hub genes included COPS7A, CUL3, KLHL7, and NEDD4, which can be considered the main key genes in asthenozoospermia. Moreover, evidence from the protein expression profiles of human sperm indicated that sperm protein exhibits a significantly higher or lower expression in asthenozoospermia[40]. In our study, the expression of these key genes was verified by collecting samples from asthenospermia patients, and the results were consistent with this hypothesis.

During spermatogenesis, protein ubiquitination has been shown to be involved in the stability of sperm cell structure and function[41–43]. The four hub genes are related to ubiquitin conjugation and protein degradation. COPS7A, the most significant gene, belongs to the COP9 signalosome (CSN) complex, which is an evolutionarily conserved multisubunit protease that regulates the activity of the ubiquitin conjugation pathway[44]. The interaction of COPS7A and polyamine regulator 1 (PMF-1) is reportedly involved in the transcriptional expression of growth-related genes[45]. We observed that COPS7A expression was upregulated in asthenospermia, but the correlation between its expression and spermatogenesis remains to be understood. CUL3 encodes a member of the cullin protein family and is involved in the regulation of multiple ubiquitin pathways and cell cycles[46, 47]. The expression of CUL3 in testicular tissue is reportedly involved in mediating protein ubiquitination in spermatogenesis by binding to KLHL10 to form the ubiquitin E3 ligase[48]. Moreover, KHLH7 has been reported to be involved in regulating nucleolar integrity and promoting terminal uryltransferase 1 ubiquitination[49]. Zhou et al. found that NEDD4 expression is critical for maintaining spermatogonial stem cell homeostasis and stress response[50]. Although the bioinformatics evidence indicates that the expression disorder of these genes can affect the development and function of male sperm, it is noteworthy that our results still need to be verified by further in vivo experiments.

Increasing lines of evidence show that sperm RNAs, including various mRNAs, miRNAs, and lncRNAs, play a crucial role in regulating male fertility[51]. Seminal plasma contains large amounts of miRNAs, and their epigenetic signatures in spermatogenesis remain unclear. These miRNAs participate in the spermatogenesis process, can reflect the pathophysiological changes of sperm cells, and serve as a potential biomarker[52]. Moreover, the synergistic effect of TFs and miRNA in the epigenetic regulatory network may be involved in the functional regulation mechanism of target genes[53]. In the current study, we constructed a miRNA-mRNA-TF network, and the interaction of all the centers provided new clues for exploring the potential mechanisms between miRNAs and their target mRNAs in asthenozoospermia. This network provides a new method for elucidating the potential mechanisms and molecular targets of asthenozoospermia.

Our study has some limitations. The two asthenozoospermia datasets retrieved from the GEO database have very small sample sizes, which could result in poor repeatability of the results. Due to insufficient clinical samples and funds, the pathogenic mechanism of four hub genes in the asthenozoospermia could not be verified. Moreover, the mechanism through which miRNAs and TFs participate in the regulation of hub genes has not been determined experimentally. We will conduct experimental verification in the future to confirm the potential mechanisms of the key genes and signaling pathways identified in this study.

## Conclusion

In summary, we firstly identified 271 DEGs that are potentially involved in asthenozoospermia based on the GEO database. Importantly, the interaction between the identified DEGs identified a regulatory pathway for asthenozoospermia, which includes the phospholipase D signaling pathway, the cGMP-PKG signaling pathway, and the Wnt signaling pathway. Among the identified DEGs, four novel key DEGs were observed in the pathogenesis of asthenozoospermia. Understanding the key genes and signaling pathways in asthenozoospermia can help determine the causes of male infertility, which will aid the search for new therapies and strategies for male contraception.

## Supplementary Information

**Additional file 1:** Supplementary results of gene differential analysis and functional enrichment analysis for asthenozoospermia.

## Data Availability

Publicly available datasets were analyzed in this study, and these datasets can be found here: https://www.ncbi.nlm.nih.gov/geo/query/acc.cgi?acc=GSE92578; https://academic.oup.com/biolrepr. od/article/100/4/982/5,230,853#134,234,708.

## Abbreviations

GEO: Gene Expression Omnibus
DEGs: Differentially expressed genes
PPI: Protein-protein interaction
qRT-PCR: quantitative real-time reverse transcription-polymerase chain reaction
WHO: World Health Organization
PR: Progressive Motility
NP: Non-progressive motility
MCC: maximal clique centrality algorithm
