# Supplementary material for: Bioinformatics analysis identifies potential hub genes and crucial pathways in the pathogenesis of asthenozoospermia
Source: BMC Med Genomics. 2022 Dec 5;15:252. doi: 10.1186/s12920-022-01407-5 (PMC9724253; doi:10.1186/s12920-022-01407-5)
Supplement: Supplementary file 1 — Additional file 1: Supplementary results of gene differential analysis and functional enrichment analysis for asthenozoospermia. [file 12920_2022_1407_MOESM1_ESM.pdf]

Supplementary Material

Supplementary Figures and Tables

1.1 Supplementary Figures 1

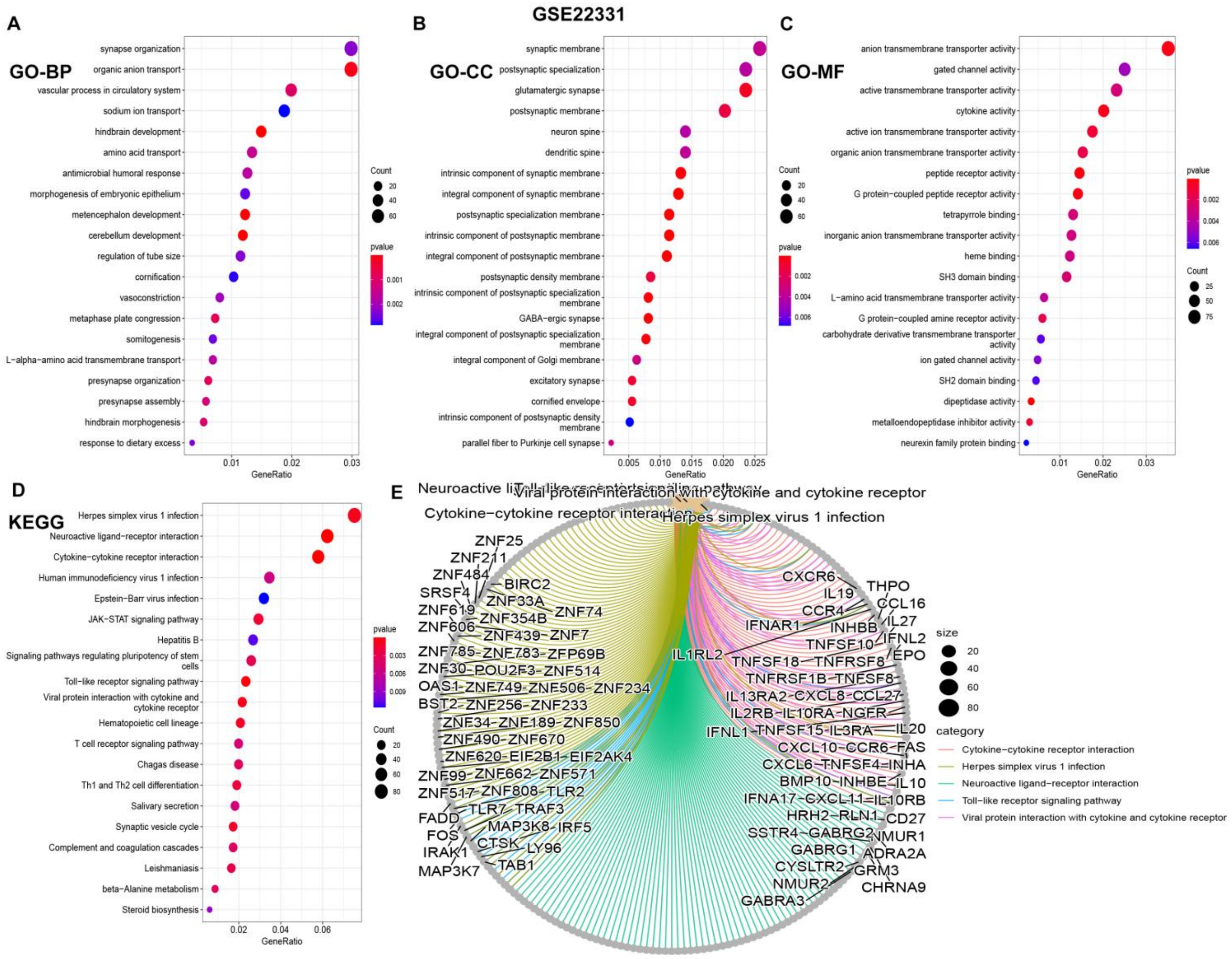

Supplementary Fig. 1: GO annotation enrichment and KEGG analysis in GSE22331 dataset. The top 20 enriched GO (A) BP, (B) CC and (C) MF terms. (D) The top 20 enriched KEGG pathways. (E) The underlying connection between DEGs and pathways in the top KEGG pathway. GO, gene ontology; DEG, differentially expressed gene; KEGG, Kyoto encyclopedia of genes and genomes.

1.2 Supplementary Figures 2

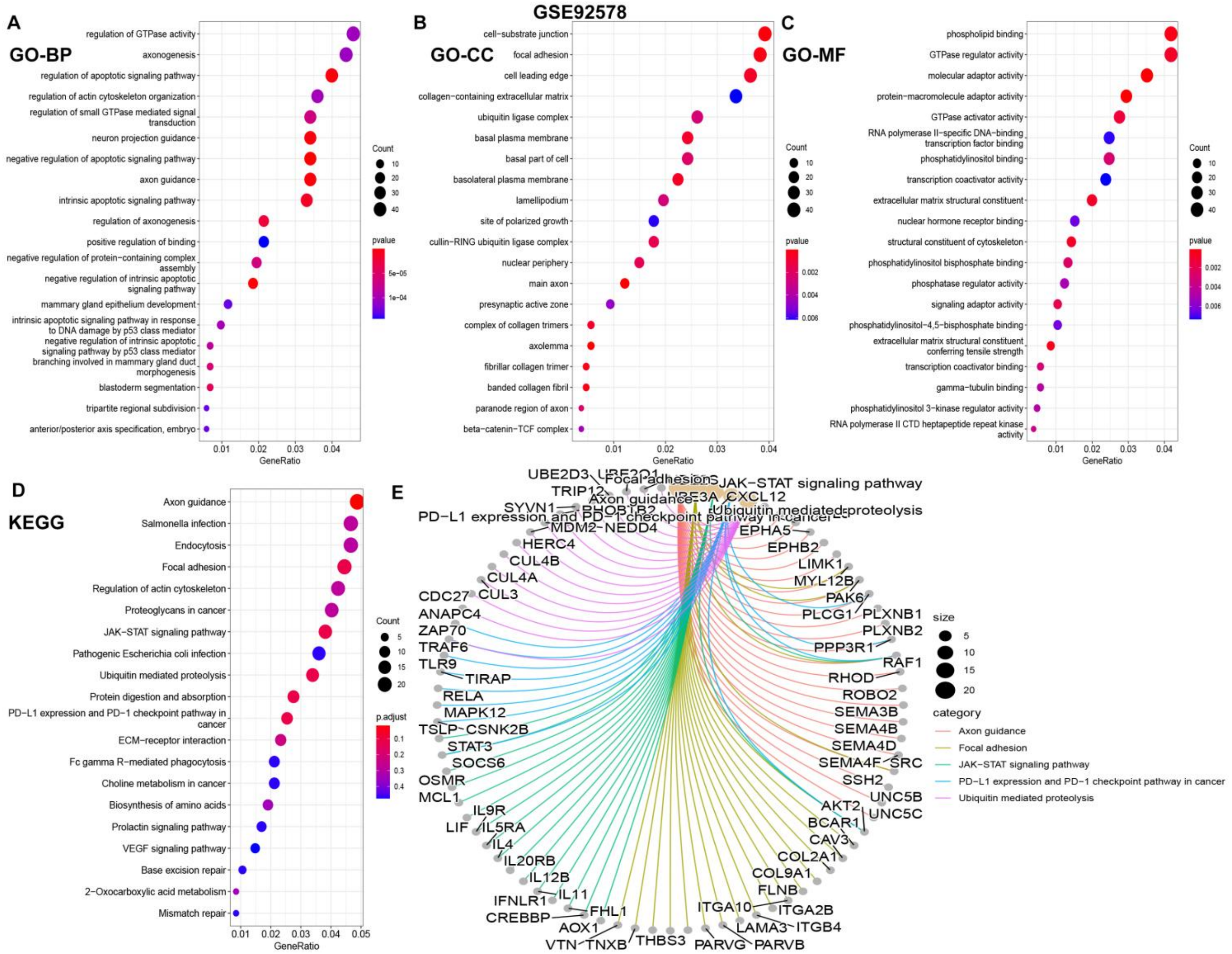

Supplementary Fig. 2: GO annotation enrichment and KEGG analysis in GSE92578 dataset. The top 20 enriched GO (A) BP, (B) CC and (C) MF terms. (D) The top 20 enriched KEGG pathways. (E) The underlying connection between DEGs and pathways in the top KEGG pathway. GO, gene ontology; DEG, differentially expressed gene; KEGG, Kyoto encyclopedia of genes and genomes.

1.3 Supplementary Figures 3

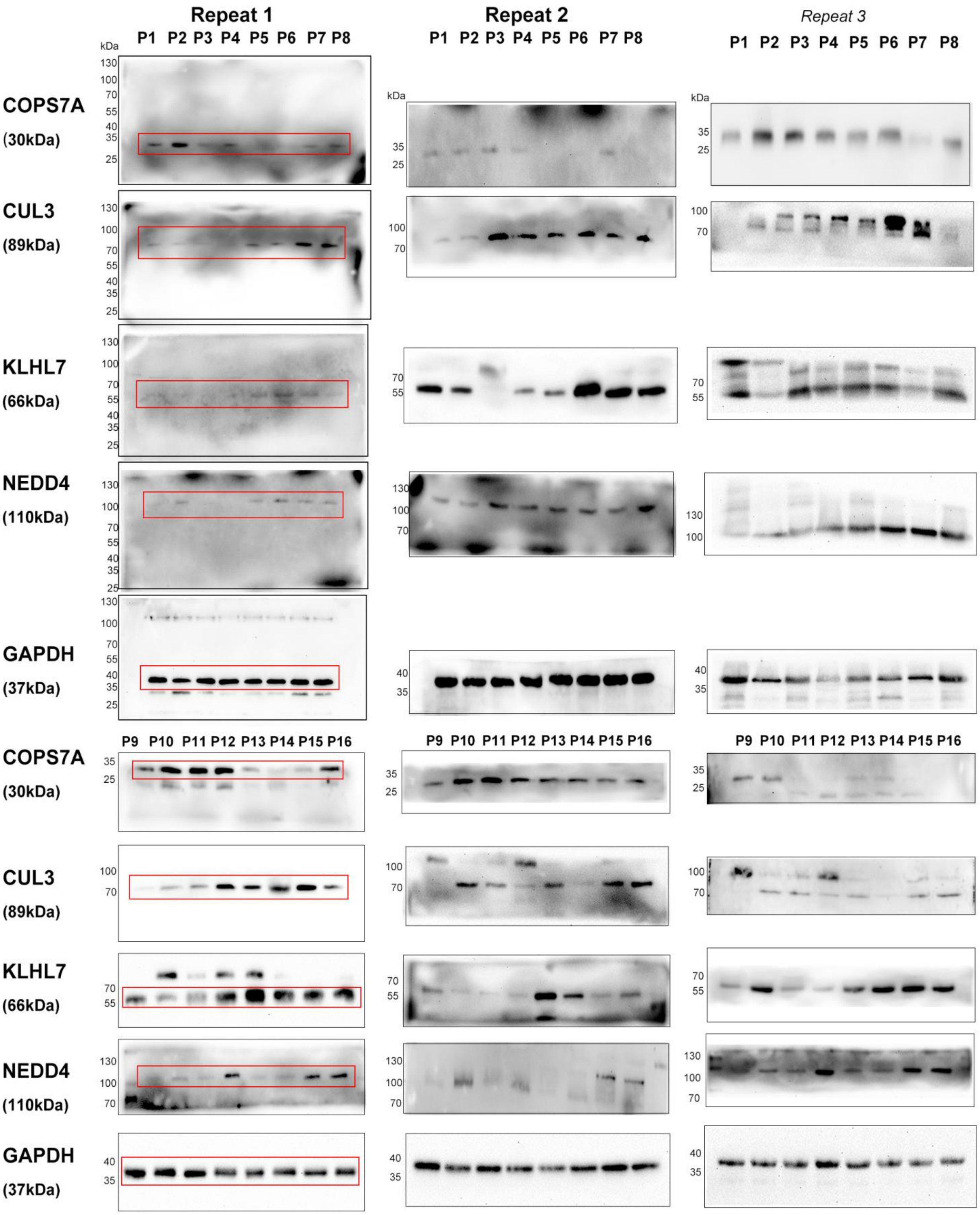

Supplementary Fig. 3: The original images of all blots with three replicates corresponding to Figure 9B. During blotting, the blots were cut prior to hybridisation with antibodies based on the molecular

weight.

2.1 Supplementary Table 1

Table S1. The 3019 upregulated DEGs in GSE22331.

Gene ID	logFC	logCPM	PValue	FDR
CASC16	6.758519429	5.29859382	3.13E-08	0.000736237
DCAF12L2	6.675581499	4.674082202	3.86E-07	0.0045298
LOC338694	6.387698748	4.388480993	1.23E-06	0.005359422
AGL	6.276248328	3.718542701	2.63E-06	0.005359422
DLX5	6.202779807	4.109793261	3.70E-06	0.005359422
LOC101928618	6.077698845	4.173197795	2.89E-06	0.005359422
SYNE4	6.019342896	4.801736144	9.28E-07	0.005359422
KIAA1598	6.017756299	4.279445429	1.91E-06	0.005359422
EP300-AS1	6.012428929	3.592319955	2.73E-05	0.006472164
BLNK	5.972671544	3.980282215	6.28E-06	0.005459891
LOC101927196	5.933213594	4.031588022	5.12E-06	0.005359422
NT5C2	5.919972752	4.183413635	2.89E-06	0.005359422
LINC01215	5.90321342	3.605364571	2.73E-05	0.006472164
RP11-488L18.10	5.89758908	3.599939939	2.73E-05	0.006472164
LOC100505635	5.870418178	4.134859435	3.47E-06	0.005359422
SPOCK1	5.812940765	4.295368078	1.91E-06	0.005359422
DKFZp667F0711	5.792018601	3.607519495	2.73E-05	0.006472164
IRX3	5.77126937	3.958123264	7.21E-06	0.005648908
LOC101929261	5.759243218	3.772661714	1.41E-05	0.006472164
LINC00622	5.751722886	3.765381204	1.52E-05	0.006472164
LOC100289058	5.699201319	4.183984185	2.89E-06	0.005359422
ZNF808	5.679575561	3.695668234	1.94E-05	0.006472164
LOC102723704	5.673089828	4.017954709	5.48E-06	0.005359422
MTUS2-AS1	5.64641515	3.91645932	8.32E-06	0.006107357
PIR	5.643715812	3.750196248	1.52E-05	0.006472164
RP11-116O18.1	5.598877206	3.790889968	1.30E-05	0.006472164
C17orf82	5.584762083	5.058870984	1.02E-06	0.005359422
RP1-30M3.5	5.560086945	3.832824956	1.12E-05	0.006472164
RP11-456P18.2	5.527871271	4.017040186	5.48E-06	0.005359422
LOC283516	5.526403287	3.548526519	3.25E-05	0.006649247
DMBX1	5.524421072	3.635334452	2.50E-05	0.006472164
SOWAHA	5.506195685	3.780784964	1.41E-05	0.006472164
KCNQ1-AS1	5.491619146	4.109716334	1.45E-05	0.006472164
SERPINI2	5.480307459	3.676654357	2.11E-05	0.006472164
GABARAPL3	5.470662528	4.026821409	5.48E-06	0.005359422

TVP23B	5.460270817	3.485392875	4.29E-05	0.007089
DCXR	5.458004764	4.453256102	3.96E-06	0.005359422
OR2B2	5.444620128	3.276331083	9.52E-05	0.008697326
RP11-1017G21.4	5.440640953	3.165273216	0.000147935	0.010097727
GUSBP3 /// GUSBP9	5.419286625	3.977021845	6.73E-06	0.005569828
RLN1	5.413422209	3.691526958	1.94E-05	0.006472164
RP11-119F7.5	5.397352854	3.751102015	1.65E-05	0.006472164
PROCR	5.391337939	3.5080843	3.90E-05	0.006860454
BC022892	5.369965152	3.487742683	4.29E-05	0.007089
SOX30	5.36518943	3.987071135	2.29E-05	0.006472164
TAS2R10	5.36005995	3.196875807	0.000132109	0.009926277
DIRC1	5.323420004	3.262176135	0.000105985	0.009086856
SLC26A4-AS1	5.323420004	3.262176135	0.000105985	0.009086856
LINC00867	5.30874999	4.049760491	1.88E-05	0.006472164
TTC8	5.299944121	3.582960357	2.98E-05	0.006649247
ERVV-1	5.289076944	4.768733723	3.26E-06	0.005359422
GCM2	5.287687376	3.40969504	5.71E-05	0.007848076
DDHD2	5.281669005	3.779255069	1.41E-05	0.006472164
BAGE2 /// BAGE4	5.279824623	3.842421525	1.12E-05	0.006472164
ZNF208 /// ZNF595 /// ZNF718	5.276722057	3.56083204	3.25E-05	0.006649247
AFAP1	5.275634431	3.217364593	0.000118214	0.009478097
CLCA4	5.271312257	3.394212787	6.31E-05	0.007934068
CXCL3	5.265810945	4.265956065	7.98E-06	0.006048435
SLC6A3	5.262324668	4.742589035	3.55E-06	0.005359422
STATH	5.244719217	3.369107028	6.97E-05	0.007990346
RP11-676J12.4	5.235598197	3.667286325	2.30E-05	0.006472164
KIF9-AS1	5.226936486	4.130533667	1.36E-05	0.006472164
RNF220	5.226233652	4.601277554	6.27E-06	0.005459891
CTB-78F1.1	5.224689692	3.656868262	2.30E-05	0.006472164
TCRA /// TRAV8-6 /// TRAV8-6	5.217540845	3.717796029	1.79E-05	0.006472164
NLRC5	5.209109798	4.584587815	6.88E-06	0.005569828
LLNLR-246C6.1	5.207797901	3.495342176	4.29E-05	0.007089
DKFZP434I0714 /// MIR4453	5.188395522	3.689935848	2.11E-05	0.006472164
LOC100289094	5.188395522	3.689935848	2.11E-05	0.006472164
STRIP1	5.187791185	4.861346093	5.44E-06	0.005359422

KIAA0247	5.183034291	3.983581163	2.45E-05	0.006472164
HOXA5	5.183031011	3.47188029	4.71E-05	0.007278211
KCND1	5.179684914	4.360211783	5.72E-06	0.005373572
PANK1	5.171022795	3.11984965	0.000166	0.010613982
ANKFN1 /// BC037494	5.17012723	3.020345208	0.000237677	0.012400588
LINC00052	5.168955164	3.297841219	8.57E-05	0.008464697
MCF2L2	5.166998753	3.116115275	0.000166	0.010613982
LOC729173	5.164010274	4.018403553	2.14E-05	0.006472164
EGFEM1P	5.15830842	3.287858456	9.52E-05	0.008697326
SOX2-OT	5.155226317	2.665542255	0.000935753	0.024755296
RP11-305O4.3	5.15446542	3.843738114	4.03E-05	0.006860454
IFNA5	5.152746152	3.004333876	0.000269148	0.013255415
ZNF280A	5.148171507	4.054428512	1.88E-05	0.006472164
MOGS	5.144017211	4.194409025	1.07E-05	0.006472164
CSAG2 /// CSAG3	5.141331127	4.097397264	1.55E-05	0.006472164
POLE3	5.139348789	3.886445152	3.48E-05	0.006712069
LOC340017	5.127979412	2.202755507	0.000900684	0.024718291
KMT2A /// LOC101929115	5.11510191	3.74657612	5.90E-05	0.007872709
MFSD3	5.109809548	3.96621795	2.63E-05	0.006472164
ZNF517	5.106072297	3.399227289	6.31E-05	0.007934068
SHISA2	5.099310043	2.95526843	0.000305554	0.013911006
SRGAP2C	5.098596096	3.145077366	0.000166	0.010613982
DNMBP	5.094397078	3.60040625	2.98E-05	0.006649247
LOC101060233 /// OPN1LW /// OPN1MW /// OPN1MW2	5.093003899	3.663615777	2.30E-05	0.006472164
PCDH8	5.092723242	3.226543388	0.000118214	0.009478097
ZW10	5.091189907	4.096957428	1.65E-05	0.006472164
CTD-2076M15.1	5.085635931	2.942752163	0.000347793	0.01501903
HMGB3P19 /// HMGB3P19	5.082924729	3.58951451	2.98E-05	0.006649247
HAUS7	5.06263136	3.634726944	2.50E-05	0.006472164
RP11-466A19.8	5.051980503	4.011320503	2.29E-05	0.006472164
ADAM1A /// ADAM1A	5.043430421	3.18067005	0.000147935	0.010097727
POLDIP3 /// RRP7B	5.038763454	3.845052984	4.03E-05	0.006860454
ABHD15	5.038389631	3.673450668	8.14E-05	0.008218811
AJ606316 ///	5.031811933	3.169884617	0.000147935	0.010097727

LOC644135				
LOC440173	5.025659441	3.885473491	3.48E-05	0.006712069
ERMAP	5.013728471	3.650013714	8.85E-05	0.008464697
PP12719	5.008361725	4.275644618	2.22E-05	0.006472164
ODAM	5.006805016	3.379902397	6.97E-05	0.007990346
TMPRSS11E	4.997278528	3.858321132	4.03E-05	0.006860454
BTLA	4.983949272	3.946030535	2.81E-05	0.006546022
IL9	4.983939934	3.428741515	5.71E-05	0.007848076
LOC283357	4.982505417	2.512546183	0.001507645	0.032523036
TOLLIP-AS1	4.982243549	4.169260413	1.21E-05	0.006472164
RPS4XP3 /// RPS4XP3	4.977604607	3.201368298	0.000132109	0.009926277
KCNK13	4.977345668	3.615502677	9.63E-05	0.008697326
LOC284023	4.973803088	4.11881639	1.45E-05	0.006472164
DUSP5P1	4.935580626	2.994873474	0.000269148	0.013255415
LOC101928700	4.935114697	3.161996187	0.000147935	0.010097727
NBPF4 /// NBPF5P	4.931287759	3.687813575	7.50E-05	0.008199045
HNRNPU-AS1	4.926702996	2.986757767	0.000305554	0.013911006
LOC101927934	4.924308137	3.888981461	3.48E-05	0.006712069
ACTN4	4.92420089	4.271385253	2.35E-05	0.006472164
ZNF571	4.91780527	2.456061122	0.001782286	0.034862167
LINC00415 /// LINC00415	4.914909731	3.880008285	3.74E-05	0.006855942
MTND1P33 /// MTND1P33	4.91117276	2.450297274	0.001782286	0.034862167
LYSMD3	4.910171018	3.057612631	0.000237677	0.012400588
TAS2R16	4.909703755	3.490122063	4.71E-05	0.007278211
RP11-589P10.5	4.907118956	3.966469762	2.63E-05	0.006472164
ANXA2P1	4.904411627	4.05218284	2.00E-05	0.006472164
APMAP	4.898858649	4.246980984	2.49E-05	0.006472164
MST4	4.895444615	3.202488312	0.000132109	0.009926277
FAM114A1	4.894831412	4.606161909	1.55E-05	0.006472164
ZNF662	4.887587473	4.036061607	2.00E-05	0.006472164
ADIPOQ	4.884668241	3.466592357	5.18E-05	0.007562486
GABPB2	4.879485383	4.437323126	1.23E-05	0.006472164
IL36A	4.876006827	4.297996737	2.10E-05	0.006472164
TMC1	4.871100532	3.574117211	0.000114341	0.009478097
SKIV2L	4.870055107	4.639541795	1.35E-05	0.006472164
EID2	4.868934943	4.291189442	2.22E-05	0.006472164
SCN1A	4.862721738	3.134224969	0.000166	0.010613982
LINC01093	4.862459866	3.381834431	6.97E-05	0.007990346
GPR101	4.862032412	3.923390922	3.24E-05	0.006649247
C11orf42	4.854275565	3.72217033	6.92E-05	0.007990346

LINC00619	4.850863909	3.957483011	2.81E-05	0.006546022
RP11-352B15.2	4.847139567	2.727204778	0.0006939	0.021170904
MANBA	4.842310543	3.603622643	0.000104849	0.009086856
BC024169	4.842188377	3.362904441	7.72E-05	0.008199045
CD99P1	4.841107306	4.191471493	3.14E-05	0.006649247
RP11-714L20.1	4.840295574	4.263643481	2.49E-05	0.006472164
MCOLN1	4.836125415	5.7012913	4.29E-06	0.005359422
FGF13-AS1	4.832950146	2.810882272	0.000521585	0.018706971
SYT8	4.824968766	3.694412266	7.50E-05	0.008199045
ZNHIT1	4.820473678	4.824940491	1.39E-05	0.006472164
LOC389906 /// LOC441528	4.816673275	3.880159259	3.74E-05	0.006855942
ABHD17C	4.815552981	3.463042052	0.000179717	0.011018451
LOC100128554	4.814872056	3.784794078	5.46E-05	0.007677442
LINC00244	4.811448111	3.681623563	8.14E-05	0.008218811
ARHGAP39	4.810600598	3.73161797	6.38E-05	0.007934068
GYS2	4.809431888	2.965575599	0.000305554	0.013911006
MPP1	4.80661557	3.824510441	4.68E-05	0.007278211
LOC100133669	4.793634383	3.946293531	3.02E-05	0.006649247
LOC101927067	4.789738923	3.554112677	0.000124843	0.009674353
AC099850.1 /// BC017255	4.788465947	4.503474429	2.26E-05	0.006472164
BTBD1	4.786746967	3.494796519	0.000163744	0.010613982
RP11-251G23.5	4.78296211	3.241280615	0.000118214	0.009478097
LOC340085	4.776420944	4.052838993	5.49E-05	0.007677442
LINC01169	4.769722075	3.359118466	7.72E-05	0.008199045
RP11-171I2.4	4.76628472	3.920248341	3.24E-05	0.006649247
KRT19P2	4.764506325	3.637310657	9.63E-05	0.008697326
BGLAP /// PMF1 /// PMF1-BGLAP	4.763263939	3.874037171	4.03E-05	0.006860454
WNT8B	4.758551244	3.468390063	0.000179717	0.011018451
CCDC113	4.753367425	3.071714409	0.000210393	0.011648108
LOC158402	4.753367425	3.071714409	0.000210393	0.011648108
FTHL17	4.750704621	3.40242972	0.000217408	0.011660639
FRMD6	4.749676538	3.648898324	8.85E-05	0.008464697
LINC00852	4.747267539	3.274669629	0.000105985	0.009086856
ZNF99	4.74536831	2.778221104	0.000600631	0.020128424
SERPINB4	4.744931059	3.064002476	0.000237677	0.012400588
HAUS4 /// MIR4707	4.743827285	3.764976737	5.90E-05	0.007872709
RWDD2B	4.74032616	4.018395235	6.26E-05	0.007934068
SPANXA2-OT1	4.738174551	4.016344157	6.26E-05	0.007934068
TMEM61	4.73775401	3.805211859	5.05E-05	0.007509449

DBIL5P2	4.737174459	3.55913432	0.000124843	0.009674353
RPLP2P1 /// RPLP2P1	4.735221473	3.660396054	8.85E-05	0.008464697
IGHD /// IGHG1 /// IGHM	4.720537433	3.742953179	6.38E-05	0.007934068
OR7A5	4.720078729	3.432448865	0.000197524	0.011215713
TUBB8P2 /// TUBB8P2	4.707251177	3.583452171	0.000114341	0.009478097
C9orf152	4.706344751	3.02880891	0.000269148	0.013255415
TCR-alpha /// TRAV12-3 /// TRAV12-3 /// YME1L1	4.706186365	2.952446514	0.000347793	0.01501903
RP11-227D13.1	4.705943881	4.169992966	3.54E-05	0.006712069
PCOLCE2	4.705013184	2.499789883	0.001782286	0.034862167
NCOA5	4.704239113	3.554668055	0.000124843	0.009674353
ATP13A1	4.70110664	3.525320394	0.000149393	0.010097727
CTSW	4.697621125	4.091045947	4.83E-05	0.00730635
CTD-3025N20.3	4.694440058	2.687376769	0.000804374	0.022821694
JAZF1-AS1	4.694440058	2.687376769	0.000804374	0.022821694
RP11-90P13.1	4.686581572	4.490529689	2.49E-05	0.006472164
BEST4	4.681622194	3.277403333	0.000105985	0.009086856
KIRREL3-AS3	4.677374444	4.176954406	3.54E-05	0.006712069
AES	4.670559026	4.203911373	3.14E-05	0.006649247
PKD2	4.668865069	3.202316439	0.000132109	0.009926277
TCEAL2	4.666697987	2.992786279	0.000305554	0.013911006
NRTN	4.657308627	3.191693734	0.000147935	0.010097727
HIST1H3B	4.651377073	2.823042169	0.000521585	0.018706971
LOC93622	4.639041728	4.472179879	2.61E-05	0.006472164
DHX29	4.633591847	3.233109217	0.000118214	0.009478097
KANK3	4.631457741	3.749298012	6.38E-05	0.007934068
MOS	4.623425702	3.651483998	8.85E-05	0.008464697
LINC01141	4.621885341	3.982209652	7.15E-05	0.008080681
KIF21B	4.621758043	3.944404303	8.20E-05	0.008218811
ZFP3	4.619856407	2.621697023	0.001092649	0.027076494
STK24-AS1	4.614684331	3.215723755	0.000132109	0.009926277
DFNA5	4.613113234	3.732020198	6.92E-05	0.007990346
RP11-143I21.1	4.611243929	2.866923839	0.000454346	0.017555101
PCDHB2	4.602725134	4.139223564	4.00E-05	0.006860454
LAMTOR2	4.601832706	3.96319324	7.66E-05	0.008199045
LOC100130642	4.597002586	3.428232693	0.000197524	0.011215713
SPIN4	4.596182571	4.19763903	3.14E-05	0.006649247

SAMD5	4.590320687	3.330932359	0.000292548	0.013800282
RBMS3-AS3	4.587363894	2.498978253	0.001782286	0.034862167
MYO5C	4.585831034	4.622654229	3.07E-05	0.006649247
LOC101928417	4.583978098	2.918094207	0.000396951	0.01620832
RMI1	4.581778483	4.151960237	3.76E-05	0.006855942
HRH2	4.576166948	3.782251675	5.46E-05	0.007677442
DSC1	4.576109269	3.180345984	0.000147935	0.010097727
BC039686	4.575266223	3.354106777	0.000264573	0.013112527
LOC101927365	4.574487245	2.909565847	0.000396951	0.01620832
CTNNBIP1	4.571551194	4.32432486	4.62E-05	0.007278211
TEX12	4.569608656	3.1114466	0.000186674	0.011018451
IGHG1 /// IGHM /// LOC102725426 /// MIR8071-1 /// MIR8071-2	4.564520147	4.102893561	4.53E-05	0.007278211
MRGPRX3	4.564332713	3.169570518	0.000166	0.010613982
LRP11	4.563020831	3.448767718	0.000197524	0.011215713
FTO	4.562700954	4.425852732	3.20E-05	0.006649247
LINC00621	4.559616049	3.847011848	0.000117022	0.009478097
ATP5J2-PTCD1 /// PTCD1	4.558097978	4.687259092	2.47E-05	0.006472164
FLJ16779	4.552662446	2.651582594	0.000935753	0.024755296
LIFR-AS1 /// MIR3650	4.552662446	2.651582594	0.000935753	0.024755296
SLC4A3	4.544677663	4.408635391	3.36E-05	0.006712069
FUT4	4.544056417	3.269414284	0.000359459	0.015133359
LONRF3	4.54017829	3.526464125	0.000149393	0.010097727
CNTN3	4.535886872	2.874970231	0.000454346	0.017555101
IPW /// LOC101930404 /// PWARSN /// SNORD107 /// SNORD115-13 /// SNORD115-26 /// SNORD115-7 /// SNORD116-22 /// SNORD116-28 /// SNORD116-4	4.525319501	2.790283995	0.000600631	0.020128424

CTC-444N24.11	4.521987256	3.960127703	8.20E-05	0.008218811
LRTM2	4.518980611	4.059690362	5.49E-05	0.007677442
SLC18B1	4.517895692	3.457148246	0.000197524	0.011215713
MYH13	4.512404759	3.50062853	0.000163744	0.010613982
LINC00485	4.510027621	2.226168082	0.004429481	0.058645551
SLITRK2	4.506404765	3.631948285	9.63E-05	0.008697326
CTD-2547L24.4	4.504395478	4.496928306	5.12E-05	0.007523628
RP1-100J12.1	4.503963312	3.114521061	0.000186674	0.011018451
RP11-274H2.5	4.503963312	3.114521061	0.000186674	0.011018451
PP12613	4.503215174	2.917380716	0.000396951	0.01620832
LOC101928597	4.500771542	4.966605	1.62E-05	0.006472164
KLHDC2	4.490542205	4.577739231	3.68E-05	0.006855942
CHMP2A	4.488329661	3.327745592	0.000292548	0.013800282
STK11IP	4.486296786	3.272156923	0.000359459	0.015133359
ATP6V1E2 /// FLJ41757	4.485049686	3.852906378	0.000117022	0.009478097
BC037892 /// RP11-1129I3.1	4.484079964	3.695800972	8.14E-05	0.008218811
CA5A	4.481424525	3.990822743	7.15E-05	0.008080681
CYP51A1-AS1	4.48037783	3.732941701	0.000184391	0.011018451
HMHB1	4.47164078	4.257442184	6.09E-05	0.007934068
SIGMAR1	4.46549512	3.636703278	9.63E-05	0.008697326
C11orf73	4.464916103	3.571319299	0.000124843	0.009674353
ZNF788 /// ZNF788	4.46459096	2.486942845	0.001782286	0.034862167
PNPLA1	4.464102127	3.251813897	0.000399481	0.01620832
LOC254057	4.463908042	3.757026214	0.000170538	0.010740662
BC028670	4.463641217	3.974035185	7.66E-05	0.008199045
PURB	4.462489167	4.070708084	5.49E-05	0.007677442
BMS1P20 /// LOC101929959	4.455976352	2.728985761	0.000804374	0.022821694
CTA-384D8.35	4.453454508	4.183188186	8.15E-05	0.008218811
RBM3	4.452684216	3.624756291	0.000104849	0.009086856
TMEM262	4.443752669	4.173994118	8.15E-05	0.008218811
PEPD	4.439152432	4.016793961	6.69E-05	0.007990346
RP11-395B7.7	4.439135228	3.479645956	0.000179717	0.011018451
FCGR1A	4.436217135	2.99069895	0.000305554	0.013911006
RP11-378J18.8	4.433602264	3.564119004	0.000124843	0.009674353
LOC101928554	4.433212685	2.630423436	0.001092649	0.027076494
AL832909 /// LINC00984	4.432303387	4.191906475	7.68E-05	0.008199045
IFITM3	4.431641551	3.909828842	9.43E-05	0.008697326

PBX4	4.429651854	3.72496149	0.000184391	0.011018451
COL19A1	4.428792359	3.106360845	0.000696625	0.021170904
OR11A1	4.428611159	4.350071723	4.38E-05	0.00718988
GOLT1B	4.428214917	3.817922992	0.000135681	0.009926277
TSPO2	4.426057153	4.004440742	6.69E-05	0.007990346
OR2S2	4.424780014	4.320691551	4.88E-05	0.00730635
LOC100130964	4.422503305	3.213797469	0.000444759	0.017384807
RP11-44N11.3	4.418945728	3.714956343	0.000199564	0.011215713
ABCD2	4.418786465	2.770933669	0.0006939	0.021170904
ATP6V1G2	4.418721278	4.365973674	4.15E-05	0.007011187
CECR9	4.416929331	3.388267373	0.00023965	0.012400588
HBQ1	4.41595637	4.312315707	4.88E-05	0.00730635
IRF7	4.407590274	3.78021672	0.000157876	0.010536446
ZDHHC11	4.403964028	3.98362244	7.66E-05	0.008199045
BC127192 /// SHANK2	4.399297102	3.192654407	0.000496089	0.018410938
ADAMTS14	4.398891303	3.487849596	0.000179717	0.011018451
RFXAP	4.395271346	3.712182453	0.000199564	0.011215713
SIGLEC9	4.393830095	3.342669618	0.000292548	0.013800282
STGC3	4.3911687	3.524760464	0.000149393	0.010097727
LOC101930149 /// LOC101930286	4.389555065	4.653003817	5.31E-05	0.007677442
FGF7 /// KGFLP1 /// KGFLP2	4.389541421	2.231956155	0.004429481	0.058645551
ATP4A	4.389064691	4.806057332	3.09E-05	0.006649247
LOC100132891	4.388867125	2.6701513	0.000935753	0.024755296
TSLP	4.387791404	2.508500577	0.001782286	0.034862167
PCBD1	4.386865642	3.563560187	0.00013648	0.009926277
FLJ13744	4.384938794	3.831168566	0.000135681	0.009926277
SGTB	4.378940234	2.321455149	0.003029173	0.047599554
FUNDC1	4.37785851	3.555210116	0.00013648	0.009926277
TTC30A	4.377372361	2.660123405	0.000935753	0.024755296
LOC101927482	4.37057179	2.994051643	0.000305554	0.013911006
NMUR1	4.370043104	4.529622885	4.65E-05	0.007278211
LOC100631377 /// LOC101928673	4.368957702	4.160174633	8.65E-05	0.008464697
IGHG1 /// LOC100293211	4.368562321	3.743740303	0.000184391	0.011018451
LOC285889	4.367948279	2.864746579	0.000521585	0.018706971
CLRN1-AS1	4.366958706	2.928666996	0.000396951	0.01620832
OR2B3	4.365922531	3.741276797	0.000184391	0.011018451

KRTAP5-2	4.364303219	3.410584383	0.00023965	0.012400588
ACTG1P4 /// AMY2B /// RNPC3	4.362605133	4.007653346	6.69E-05	0.007990346
BTBD18	4.361527192	2.985943921	0.000305554	0.013911006
LOC101927901	4.361446513	3.361223724	0.000264573	0.013112527
SKA3	4.360504099	3.452410428	0.000197524	0.011215713
TCRDV2 /// TRDC /// TRDC /// YME1L1	4.360120228	4.03580195	0.000142344	0.010097727
TRBV7-3 /// TRBV7-3	4.356505343	4.229820943	6.83E-05	0.007990346
KDM2B	4.356454231	4.700237131	4.47E-05	0.007278211
LOC284263	4.355410949	3.206138476	0.000496089	0.018410938
SP7	4.355314804	3.731382242	0.000184391	0.011018451
SLC18A1	4.353357771	3.574066023	0.000124843	0.009674353
SERPINB2	4.351538139	3.202617916	0.000496089	0.018410938
MAGEE2	4.348228264	2.634764588	0.001092649	0.027076494
HYDIN	4.3477459	3.090590031	0.000696625	0.021170904
NID2	4.3471895	3.962042068	8.20E-05	0.008218811
U47924.27	4.347175542	3.993145078	7.15E-05	0.008080681
CD3G	4.344523603	3.645625263	0.000254555	0.012805157
RP11-395N3.1	4.34311347	2.907419303	0.000396951	0.01620832
STXBP1	4.34311347	2.907419303	0.000396951	0.01620832
LINC00507	4.342761163	2.774859435	0.0006939	0.021170904
HTN3	4.340920549	3.434375104	0.000217408	0.011660639
SP8	4.338474429	4.199528621	7.68E-05	0.008199045
BOK	4.336329125	3.240268364	0.000444759	0.017384807
LEP	4.336012422	3.637721224	0.000276657	0.013428147
MSANTD1	4.334098632	4.620318445	6.05E-05	0.007934068
VAC14-AS1	4.332935564	3.884427507	0.000108817	0.009228654
RP1L1	4.332384474	3.554649303	0.00013648	0.009926277
LOC101929486	4.330516417	3.379547996	0.000264573	0.013112527
RP11-539I5.1	4.328847035	3.509880511	0.000163744	0.010613982
FKTN	4.324770878	2.95307721	0.000347793	0.01501903
LOC101928230	4.32260185	3.504117614	0.000163744	0.010613982
BBS12	4.319869081	2.363895004	0.003029173	0.047599554
MYF6	4.318627517	3.457791201	0.000197524	0.011215713
CYP1A1	4.318357957	3.273638599	0.000359459	0.015133359
SPINK4	4.317055163	4.218713375	7.24E-05	0.008139604
MED9	4.312994992	4.497020489	5.12E-05	0.007523628
CA5B /// CA5BP1	4.312811751	3.929797606	9.43E-05	0.008697326

COX6A2	4.312353865	4.768951342	3.49E-05	0.006712069
SLITRK5	4.311471024	2.676859682	0.000935753	0.024755296
NPTN	4.31049051	3.927623088	9.43E-05	0.008697326
LINC01213	4.304932181	3.720900011	0.000199564	0.011215713
HOXD12	4.302018436	2.806445478	0.000600631	0.020128424
BOLA3	4.299871238	3.948756456	8.79E-05	0.008464697
LOC101929132	4.298373589	3.101262171	0.000696625	0.021170904
LOC100129112	4.298098471	3.750075734	0.000184391	0.011018451
MAN1C1	4.293250297	3.27500553	0.000359459	0.015133359
PCDHGB6	4.291334912	2.65934941	0.001092649	0.027076494
HNF1A	4.289805491	4.028810661	0.000142344	0.010097727
MIR6516 /// SCARNA16 /// SNHG20	4.288725189	2.727469646	0.000804374	0.022821694
CASC14	4.288535104	4.242734796	6.45E-05	0.00797739
CENPC	4.288112057	2.582761192	0.001280849	0.029320273
CFLAR-AS1	4.283945136	3.382078793	0.000264573	0.013112527
SH3RF1	4.283224305	2.885513054	0.000454346	0.017555101
LINC01023	4.282740435	4.630179111	6.05E-05	0.007934068
CST11	4.282234846	3.380513055	0.000264573	0.013112527
LSM2	4.28159705	3.662837515	0.000254555	0.012805157
SAA3P	4.280304376	4.048554732	0.000133449	0.009926277
ACACA	4.279265574	4.889174299	4.00E-05	0.006860454
ZFYVE19	4.27770436	4.420189411	6.90E-05	0.007990346
BC047615	4.27767081	2.717814155	0.000804374	0.022821694
SLC2A2	4.274170611	2.846312242	0.000521585	0.018706971
MOAP1	4.269884809	4.39060755	7.64E-05	0.008199045
ARNTL2-AS1	4.269578177	3.688059767	0.000216204	0.011660639
H1FX	4.267568623	4.925298651	3.45E-05	0.006712069
KHK	4.265787977	4.515095417	4.88E-05	0.00730635
TARDBPP1 /// TARDBPP1	4.261784722	4.24232599	6.83E-05	0.007990346
PGLYRP1	4.260472167	3.315505415	0.00032401	0.014451669
CYP4F8	4.259467434	3.119318873	0.000696625	0.021170904
LINC00698	4.254408253	4.398171631	7.64E-05	0.008199045
CER1	4.253355979	4.329601219	9.95E-05	0.008850671
LINC01419	4.252239592	2.393257494	0.002525671	0.042380758
RP11-58O3.2	4.252026132	3.775774262	0.000170538	0.010740662
GCHFR	4.249916645	3.705306198	0.000216204	0.011660639
LOC101928476	4.248592316	3.900798195	0.000101272	0.00891045
KLRG1	4.242166661	3.430168234	0.000217408	0.011660639
C4orf27	4.240386352	3.642960762	0.000276657	0.013428147
UBR7	4.239044857	3.829178873	0.000135681	0.009926277
LOC100294145	4.236858157	3.97920372	0.000173451	0.010889271

UBE2Q2	4.236401662	3.761253306	0.000170538	0.010740662
FAM69C	4.234397611	3.917937933	0.000228024	0.01214276
PCED1B-AS1	4.233781053	3.758819462	0.000170538	0.010740662
RBBP4P4 /// RBBP4P4	4.231301259	2.456880698	0.002116575	0.038694623
DLEU2 /// DLEU2L /// MIR15A	4.230580997	3.144496836	0.000620821	0.020256018
POU5F1B	4.230420911	4.793969327	5.67E-05	0.007848076
BNIPL /// C1orf56	4.230290278	4.00168525	0.000162279	0.010613982
ONECUT1	4.229903872	3.943515392	0.000198579	0.011215713
LOC401098	4.221466694	3.812822657	0.000146292	0.010097727
PIGW	4.216752898	3.275744161	0.000399481	0.01620832
INA	4.215133026	3.180022581	0.000554409	0.019220167
LONRF2	4.214520782	3.446163276	0.000217408	0.011660639
TRIM68	4.21383698	4.465986071	5.94E-05	0.007877669
CKMT1A /// CKMT1B	4.213388092	4.146822377	9.76E-05	0.008749187
DACT2	4.21291078	4.120738189	0.000103754	0.009086856
SLITRK3	4.210571961	3.633450857	0.000276657	0.013428147
RUSC1	4.204405227	4.648691619	5.79E-05	0.007872709
RP5-1039K5.17	4.203012543	3.435630106	0.000217408	0.011660639
LOC283278 /// PLEKHA7	4.201410996	4.280673454	0.000117132	0.009478097
LOC101927503	4.201286479	4.057210117	0.000133449	0.009926277
LOC101928612	4.200780987	4.256869899	0.000130937	0.009926277
GNG11	4.200047106	2.812179713	0.000600631	0.020128424
ADAT2	4.198401728	2.841122074	0.000521585	0.018706971
GPIHBP1	4.197984123	3.585570921	0.000327888	0.014451669
DDIT4L	4.196583743	3.724325874	0.000199564	0.011215713
ITFG2	4.192826508	3.207721716	0.000496089	0.018410938
LOC101928288	4.19043423	2.893090918	0.001458763	0.031878391
CCDC24	4.190354454	3.614799749	0.000301013	0.013911006
KRT5	4.18806852	4.148270489	9.76E-05	0.008749187
HOGA1	4.18306962	3.333777376	0.00032401	0.014451669
SCG3	4.181792311	3.807614291	0.000146292	0.010097727
SLC6A9	4.177888789	3.371452372	0.000264573	0.013112527
SNTA1	4.176873422	4.325102112	9.95E-05	0.008850671
RGS1	4.176458297	3.177203194	0.000554409	0.019220167
LOC100507165	4.176358087	3.411276135	0.00023965	0.012400588
FSIP2	4.175900896	3.893171733	0.000244627	0.012493007
C20orf96	4.170739713	2.875724302	0.001664638	0.033998787
FAM46B	4.161692654	4.476954165	0.000110818	0.009297647

HTN1	4.156685945	3.027269096	0.000882847	0.024285527
ARMCX5-GPRASP2 /// GPRASP2	4.154697768	4.066256631	0.000133449	0.009926277
EPN2-AS1	4.154631577	2.677806309	0.000935753	0.024755296
FLJ35816	4.154424865	3.12541982	0.000696625	0.021170904
AKR7A2	4.147768041	6.13878702	2.53E-05	0.006472164
GJD2	4.14630704	4.714369007	7.84E-05	0.008218811
WBSCR27	4.145771143	3.38340271	0.000264573	0.013112527
BC048103 /// CSGALNACT1	4.144701579	3.210586288	0.000496089	0.018410938
MIR622	4.141503354	2.300819682	0.00365261	0.051799007
NYNRIN	4.140684472	2.849300823	0.001664638	0.033998787
GMNC	4.137376273	3.827686002	0.000303509	0.013911006
TOP1P2	4.136845327	2.37739146	0.002525671	0.042380758
JAKMIP1	4.135765271	3.33316446	0.00032401	0.014451669
ELOVL3	4.135613823	2.844852423	0.001664638	0.033998787
N4BP3	4.134717171	2.955113492	0.00112921	0.027319677
PRO2958	4.134261086	3.527045469	0.000426839	0.017053238
CHMP4B	4.130335517	4.225431596	0.000146664	0.010097727
SLC17A3	4.124727834	3.931290375	0.000212712	0.011648108
RBM4B	4.124485469	4.710209948	8.18E-05	0.008218811
ZNF738	4.123103488	3.121377471	0.000696625	0.021170904
NUDT13	4.118518293	3.475593872	0.000512118	0.018706971
LOC441666	4.113270423	2.909191618	0.001458763	0.031878391
LOC100133445 /// LOC115110	4.112623925	4.264809901	0.000130937	0.009926277
TAF1A-AS1	4.111007325	3.351820514	0.000292548	0.013800282
NDNF	4.110194256	3.267758167	0.000399481	0.01620832
CLK1	4.109530946	2.189804223	0.005404101	0.065480303
CTD-2194D22.4	4.10790414	2.637533979	0.001092649	0.027076494
LOC100507419 /// LOC101929668	4.105886344	3.220421439	0.000496089	0.018410938
LRRC47	4.105676435	5.382591346	3.91E-05	0.006860454
C11orf45	4.102996097	2.696790227	0.000935753	0.024755296
BC022568 /// CTC-265F19.1	4.102898217	3.498360361	0.000467231	0.017818504
CTCFL	4.099744404	3.667939842	0.000254555	0.012805157
AFP	4.099611321	2.813348165	0.001904897	0.036328095
SLC17A8	4.097248753	3.52931801	0.000426839	0.017053238
CYP27B1	4.094707458	3.817855339	0.000326678	0.014451669
BC031255 ///	4.093164624	2.970902522	0.00112921	0.027319677

RP11-260O18.1				
FZD4	4.092612703	4.534271009	8.72E-05	0.008464697
LOC100505918	4.092296307	3.928644163	0.000212712	0.011648108
RGS9BP	4.092184724	3.117325205	0.000696625	0.021170904
BEND4	4.091647502	3.413154367	0.000617763	0.020256018
ISX	4.09033233	3.206435403	0.000496089	0.018410938
MIR424 /// MIR503HG	4.09023735	3.353111271	0.000749483	0.022287152
ANG	4.089739706	3.3325515	0.00032401	0.014451669
LINC00558	4.089062079	2.337612855	0.003029173	0.047599554
LOC101060019	4.086957346	3.483811569	0.000467231	0.017818504
LOC647323	4.084487248	3.893947872	0.000244627	0.012493007
PF4V1	4.083960342	2.616996807	0.001280849	0.029320273
SH3RF3-AS1	4.082819361	3.746981449	0.000184391	0.011018451
GABRG2	4.07898716	3.322824711	0.00032401	0.014451669
C1orf53	4.077468747	2.511216595	0.001782286	0.034862167
CTC-462L7.1	4.076159636	3.237089815	0.000444759	0.017384807
EIF2AK4	4.07431552	2.672058933	0.001092649	0.027076494
PTPRT	4.071837756	2.606624883	0.001280849	0.029320273
RP11-96H17.1	4.071185593	3.098586195	0.000783358	0.022635483
NSUN2	4.068264841	4.011312845	0.000162279	0.010613982
DPYS	4.065597468	3.636573186	0.000276657	0.013428147
CPEB2-AS1	4.064757654	3.138853762	0.000620821	0.020256018
ARSF	4.064524623	3.309758219	0.000359459	0.015133359
LOC101927331	4.064340011	3.463205843	0.000512118	0.018706971
AS3MT	4.063872543	3.955693512	0.000198579	0.011215713
RP11-433A10.3	4.062699604	3.091025859	0.000783358	0.022635483
GPR37 /// SEL1L2	4.058437817	3.08723164	0.000783358	0.022635483
THEM5	4.057189129	3.692533264	0.000234473	0.01235033
GPX8	4.055570176	2.937738102	0.001281795	0.029320273
PDE2A	4.050697438	3.17089928	0.000554409	0.019220167
EFS	4.049162895	2.619187615	0.001280849	0.029320273
JPX	4.047638645	3.253572126	0.000444759	0.017384807
LOC101060604 /// SLC7A5P1 /// SMG1P1 /// SMG1P3	4.0381365	2.215989315	0.004429481	0.058645551
COPS7B	4.037016262	4.39861831	0.000149583	0.010097727
LOC101929341	4.036888949	4.359819055	0.000174751	0.010889271
HMCN1	4.035885662	1.623892932	0.008564159	0.087893936
IL12A	4.035101126	4.21415921	0.000155342	0.010396859
CEACAM19	4.034048584	4.255772435	0.000138542	0.010014253
PDCD2L	4.032266349	3.397158908	0.000679941	0.021170904

LOC101928082 /// SH2D6	4.030150599	3.975952126	0.000185523	0.011018451
LINC00939	4.028843738	3.394059756	0.000679941	0.021170904
RP11-357G3.2	4.028843738	3.394059756	0.000679941	0.021170904
GBP3	4.027206597	2.63164553	0.001280849	0.029320273
MYO1H	4.026908701	3.31568864	0.000914836	0.024755296
ERAL1	4.02425077	4.044508025	0.000284665	0.013582158
NUP85	4.023760139	3.9445146	0.000212712	0.011648108
EIF2B1	4.023444171	3.497212983	0.000467231	0.017818504
COG4	4.02327939	4.694431648	8.90E-05	0.008464697
CHCHD1	4.017751917	4.689242574	8.90E-05	0.008464697
TULP4	4.017435269	3.976771902	0.000185523	0.011018451
PTCSC1	4.014770162	4.279180795	0.000123812	0.009674353
LOC101929717	4.014726416	3.910199584	0.000244627	0.012493007
EIF4A1 /// SNORA48 /// SNORA67 /// SNORD10	4.013136326	4.058065545	0.000284665	0.013582158
IFNB1	4.012596775	2.847843107	0.001664638	0.033998787
LINC01012	4.01207132	2.899551524	0.001458763	0.031878391
ADRA2A	4.010836826	2.42039932	0.002525671	0.042380758
MED15	4.010405688	4.124900769	0.000221854	0.011844986
IQCD	4.008960978	3.259735751	0.000444759	0.017384807
USHBP1	4.008960978	3.259735751	0.000444759	0.017384807
GNPDA1	4.008247952	5.023255951	6.91E-05	0.007990346
OSBPL3	4.008079801	2.309036865	0.00365261	0.051799007
UVRAG	4.007490229	2.843392622	0.001904897	0.036328095
LINC01010	4.003823676	3.580064121	0.000357582	0.015133359
LRRC37A5P	4.002932163	3.846004811	0.000303509	0.013911006
RP1-265C24.8	4.00175136	3.082755341	0.000783358	0.022635483
PLXNB3	3.999805077	4.456259836	0.000122278	0.009674353
RP11-112L7.1	3.997927145	3.814011657	0.000326678	0.014451669
FASTKD3	3.997488515	3.123431282	0.000696625	0.021170904
LOC101929473	3.996693968	2.408609245	0.002525671	0.042380758
CST7	3.993630469	3.205148953	0.000496089	0.018410938
CCDC102A	3.992753219	2.981763025	0.00112921	0.027319677
RP11-550I24.2	3.991184098	2.661281631	0.003367649	0.050071404
SCGB2A2	3.990856795	3.396561434	0.000679941	0.021170904
RP11-29P20.1	3.990837126	2.600625907	0.001280849	0.029320273
LOC100996654	3.990279744	5.146114645	6.68E-05	0.007990346
LMAN1L	3.989893392	4.42897555	0.000135135	0.009926277
CCDC69	3.98914244	3.395012284	0.000679941	0.021170904
MYF5	3.987326279	2.877919497	0.001664638	0.033998787
LOC157273	3.986200207	2.002313797	0.010215989	0.097518902

LOC102723757	3.98534922	3.155795022	0.000620821	0.020256018
LOC101928389	3.98492575	3.112264704	0.000696625	0.021170904
HEPH	3.983069575	3.132344559	0.000696625	0.021170904
CD160	3.982921991	2.924403873	0.001458763	0.031878391
C8orf22	3.98098682	2.766445263	0.002516721	0.042380758
ASIC5	3.980539974	3.387242766	0.000679941	0.021170904
KRTAP4-7	3.979001054	3.272609391	0.001013156	0.025675357
RP11-245P10.8	3.977897386	4.139197193	0.000208764	0.011621533
AKT1	3.975553325	2.761751698	0.002516721	0.042380758
AZGP1P1 /// RP4-604G5.3	3.97539721	3.975022589	0.000185523	0.011018451
SLFN12	3.974975849	3.013296102	0.000997265	0.025675357
LOC441528	3.973483479	2.646148625	0.003367649	0.050071404
IRX5	3.973440011	4.112996856	0.000235904	0.012397899
LOC401134	3.969409933	3.162711724	0.000620821	0.020256018
AC005592.3	3.969381551	3.340384228	0.000827393	0.023222371
PCNAP3 /// PCNAP3	3.968263627	3.44714341	0.000562075	0.019220167
SLC47A1	3.964828012	3.137534992	0.000696625	0.021170904
IGHV5-78	3.964213418	3.54399415	0.000390437	0.01620832
LOC101927287	3.961561252	2.635976934	0.003915642	0.054412443
RP11-339B21.15	3.956772351	2.997265751	0.00112921	0.027319677
LOC101929949	3.95540531	2.798165441	0.00218621	0.038849044
RP11-422P24.11	3.95502694	3.327474711	0.000827393	0.023222371
MGC2889	3.952373697	3.126473416	0.000696625	0.021170904
ATRN	3.951531996	3.65671341	0.000608184	0.020256018
ANO8	3.951273654	5.075750256	8.64E-05	0.008464697
ESX1	3.944487951	2.140398255	0.006635587	0.074656714
EGOT	3.943691223	3.805025563	0.000351919	0.015133359
NANP	3.943008254	2.292744732	0.00365261	0.051799007
ANKRD26	3.942807172	3.371281924	0.000749483	0.022287152
RP1-151F17.2	3.942138096	1.874841125	0.016225581	0.127354276
GNA15	3.941214273	5.054901269	9.54E-05	0.008697326
ALPK3	3.940670686	4.355643953	0.000174751	0.010889271
PON3	3.940314869	4.189406961	0.000174548	0.010889271
LOC100127974	3.938336227	2.981066164	0.00112921	0.027319677
TMEM45B	3.931423551	3.780300139	0.000379448	0.015804955
BCDIN3D	3.931382022	3.190380259	0.000554409	0.019220167
RP11-66N11.7	3.930317295	4.004905225	0.000345488	0.015002232
XKRY /// XKRY2	3.929899901	3.576209924	0.000357582	0.015133359
EPDR1	3.929160391	3.980399369	0.000369019	0.015425261

DQ570835	3.928166589	3.478573695	0.000512118	0.018706971
RP11-421E14.2	3.927391373	3.186828405	0.000554409	0.019220167
A2M-AS1	3.925488681	3.602895294	0.000327888	0.014451669
RP11-210M15.2	3.924595097	3.407708833	0.000679941	0.021170904
USF1	3.923835643	3.26186325	0.001123917	0.027319677
ADSL	3.923429177	3.864817542	0.000282223	0.013582158
SSTR4	3.923324859	3.951209173	0.000421881	0.016941606
MAGED1	3.920514487	5.75288891	6.91E-05	0.007990346
FBP2	3.917757977	3.401550114	0.000679941	0.021170904
SMIM2-AS1	3.916809139	2.915215542	0.001458763	0.031878391
LINC00636	3.916534267	4.16737241	0.000196561	0.011215713
LOC100506258	3.915866244	2.938064316	0.001281795	0.029320273
PINK1-AS	3.915300358	4.145436073	0.000208764	0.011621533
LOC101928622	3.912339657	2.761002595	0.002516721	0.042380758
UCMA	3.91110717	3.814855224	0.000351919	0.015133359
ABCC6 /// ABCC6P1 /// ABCC6P2 /// LOC101930322	3.910816852	4.373307051	0.000165853	0.010613982
LOC100506472 /// TAB1	3.91056985	3.58933638	0.000357582	0.015133359
CTD-2311B13.7 /// P775P	3.909275284	4.008569235	0.000345488	0.015002232
AX746627	3.905119256	4.136019621	0.000221854	0.011844986
LINC01133	3.903645331	3.456421682	0.000562075	0.019220167
LOC100268168	3.902715697	3.039586798	0.000997265	0.025675357
EDA2R	3.900522058	3.386043296	0.000679941	0.021170904
CNP	3.898499097	4.901840232	0.000109628	0.009264001
LINC00626	3.898381342	3.694977209	0.000517642	0.018706971
LOC100132661	3.89735497	2.4593341	0.002116575	0.038694623
GNL3	3.895061711	4.28533907	0.000228465	0.01214276
CHRNA9	3.894382275	3.345579453	0.000827393	0.023222371
DAGLA	3.89368115	3.772577522	0.000409502	0.01650089
KIAA1804	3.890211299	2.984436203	0.00112921	0.027319677
C8B	3.888642282	4.93528766	0.00014949	0.010097727
PRELID1	3.887499567	3.506473074	0.000467231	0.017818504
CTD-2541M15.1	3.887266789	2.889498046	0.001664638	0.033998787
SLC35E3	3.886763972	3.4576172	0.000562075	0.019220167
BC070490	3.886146202	3.189733602	0.001390333	0.031017767
ABCD1	3.878607416	4.877049262	0.000122571	0.009674353
RP11-613M5.1	3.877913924	3.497814746	0.000467231	0.017818504
CTD-2540F13.	3.876598311	2.442068342	0.002525671	0.042380758

2				
TCRAVN1 /// TRAV25 /// TRAV25	3.875750254	3.015900995	0.000997265	0.025675357
LAGE3	3.874150264	4.822338769	0.000148292	0.010097727
PRIM1	3.866648595	3.007923766	0.000997265	0.025675357
ITLN1	3.865217445	3.421815041	0.000617763	0.020256018
TMEM43	3.865153814	3.933286583	0.000451574	0.017555101
RP3-337H4.8	3.864095866	3.872639312	0.000282223	0.013582158
BC031864 /// LOC100507330 /// RP11-588H23.3	3.863660787	4.097748888	0.000250996	0.012762743
ZNF620	3.862403876	2.609466443	0.003915642	0.054412443
ASCC2	3.856803743	2.76494925	0.002516721	0.042380758
KIAA0895L	3.853733395	2.068330708	0.008203511	0.084939983
PPP1R16A	3.852956259	3.625369299	0.000660263	0.021170904
GRSF1	3.850334746	3.233910353	0.001248936	0.029078292
XDH	3.849863874	2.903837091	0.001458763	0.031878391
TTI1	3.841504686	3.150192447	0.001550588	0.032822782
FETUB	3.84132613	2.412857431	0.002525671	0.042380758
DTWD2	3.840060809	4.196405097	0.000320149	0.014451669
GRM3	3.836086447	3.293470695	0.001013156	0.025675357
DSP	3.835504995	4.78630752	0.000166719	0.010613982
FAM198A	3.831649976	3.424265289	0.000617763	0.020256018
SOX21	3.830311079	4.280717406	0.000241382	0.012462734
RP11-504A18.1	3.82849842	1.788847582	0.020717199	0.148245028
ZNF670	3.82849842	1.788847582	0.020717199	0.148245028
GABRG1	3.826582892	2.634420037	0.003915642	0.054412443
BAI3	3.82546894	1.878077172	0.016225581	0.127354276
RP11-798M19.6	3.825355147	2.046041638	0.008203511	0.084939983
CXorf22	3.824861115	3.418176255	0.000679941	0.021170904
LOC101927314	3.823935185	3.247000425	0.001123917	0.027319677
SLC7A11-AS1	3.823859447	3.095240662	0.000783358	0.022635483
GLUD2	3.816545512	3.829185121	0.000326678	0.014451669
LOC100131043	3.813504769	3.375458694	0.000749483	0.022287152
SLC35B3	3.811714061	3.27178207	0.001123917	0.027319677
KCNJ9	3.81092113	3.083872651	0.000783358	0.022635483
DDX5	3.810478336	3.723056559	0.000478283	0.018180935
CITED1	3.810435977	4.723912523	0.000212148	0.011648108
SCAMP3	3.809634407	5.009852734	0.000166425	0.010613982
ZNF490	3.808969567	3.435782956	0.000617763	0.020256018
LINC01119	3.806485568	4.222125747	0.00030226	0.013911006

CNIH2	3.80389871	4.915064136	0.000160663	0.010613982
C8orf44-SGK3 /// SGK3	3.803326651	2.76888384	0.002516721	0.042380758
AMER1	3.802860645	3.521940836	0.001012527	0.025675357
RAB3GAP2	3.800987169	3.549590605	0.000927373	0.024755296
FAM111B	3.798691429	2.66397869	0.003367649	0.050071404
LINC00265 /// LOC101929038	3.797878385	3.441507917	0.000617763	0.020256018
HDHD2	3.796631411	4.825275314	0.000148292	0.010097727
CXCL2	3.795784405	2.931636699	0.001458763	0.031878391
AC005606.14	3.794154381	3.925590582	0.000483714	0.018357691
AKR1C3	3.793247609	2.551301832	0.005352624	0.065287566
DBNDD1	3.791847236	4.227051201	0.000285518	0.013582158
SNHG9 /// SNORA78	3.790326269	4.030353371	0.000323678	0.014451669
TRIM5	3.788636409	2.33774969	0.00365261	0.051799007
AARS	3.788597337	5.572309252	0.00010664	0.0091098
CLIC2	3.786818262	2.653932366	0.003367649	0.050071404
LHFPL3	3.786727413	2.303744473	0.00365261	0.051799007
MAP2K1	3.785928759	4.067499904	0.000284665	0.013582158
ERVK3-2	3.784180332	2.800530281	0.00218621	0.038849044
EVPLL	3.783752627	3.504759093	0.001012527	0.025675357
PGLYRP2	3.782041817	3.473323884	0.000562075	0.019220167
RP1-217P22.2	3.779590411	3.311986713	0.000914836	0.024755296
SIGLEC11	3.778827477	2.795944118	0.00218621	0.038849044
CAND1.11	3.777192545	2.929998899	0.001458763	0.031878391
ACKR4	3.775905544	3.308711192	0.000914836	0.024755296
CD8A	3.775644349	3.839754119	0.000641766	0.020766359
KIAA0556	3.773386913	4.398609995	0.000271337	0.013307414
ENPP7	3.773036925	3.201932235	0.001390333	0.031017767
EXOSC9	3.767153001	4.376682093	0.000300604	0.013911006
CLSTN1	3.766854679	4.959155611	0.000204667	0.011475056
C2orf44	3.763418843	4.556472396	0.000248232	0.012649611
H2BFM	3.763082942	3.680107833	0.000560802	0.019220167
LRFN5	3.760682554	2.524122804	0.005352624	0.065287566
LINC00514	3.76031545	3.753553469	0.000442347	0.017384807
MAP3K7CL	3.758509904	3.259345011	0.001123917	0.027319677
LOC100996455	3.756501142	4.31725015	0.000370839	0.015473811
KCNN2	3.75616181	2.954333626	0.001281795	0.029320273
CUZD1	3.756074084	4.01972664	0.000345488	0.015002232
CTRC	3.755338878	3.11267238	0.001939802	0.036328095
RP11-326I11.3	3.753474219	3.033613373	0.000997265	0.025675357
LOC100506282	3.753156948	2.545504745	0.005352624	0.065287566
PPP1R26	3.751856441	4.855125548	0.000208152	0.011621533

ECHDC3	3.750789545	3.793465785	0.000742826	0.022258248
WNT9B	3.750654134	3.885918134	0.000556286	0.019220167
GALNT12	3.750002077	3.574531776	0.0008504	0.023670138
CASP14	3.749000991	2.861893877	0.001904897	0.036328095
GLO1	3.745949772	4.496625626	0.000313228	0.014232773
FAS-AS1	3.745901677	2.566589238	0.004569388	0.058850908
NEGR1-IT1	3.744350581	4.35566633	0.000316599	0.014358199
LGR5	3.740788092	2.640893601	0.003915642	0.054412443
FGFBP1	3.739885969	3.021777482	0.000997265	0.025675357
LYG2	3.739673774	3.684695057	0.000560802	0.019220167
BC034788	3.739504448	2.561246178	0.004569388	0.058850908
GOLGA2P7 /// LOC101929479 /// LOC642423 /// LOC727751 /// LOC80154	3.731938465	4.09705751	0.000486202	0.01836311
LOC100288238	3.728520643	3.162731663	0.001550588	0.032822782
PEX1	3.72736542	2.496458119	0.006295194	0.072628703
ZBTB42	3.727212285	2.929290315	0.001458763	0.031878391
DEK	3.725649265	3.885434106	0.000556286	0.019220167
SOX3	3.723647829	3.405975104	0.000679941	0.021170904
MDFI	3.723368256	3.591469724	0.000780726	0.022635483
HOXD3 /// HOXD4 /// LOC401021	3.723061534	2.474236592	0.006295194	0.072628703
LOC100996457	3.720608658	2.490865385	0.006295194	0.072628703
FGB	3.719805814	4.27507974	0.000436136	0.017384807
PYHIN1	3.719641126	3.679311955	0.000560802	0.019220167
PPP1R3A	3.718633898	2.281002384	0.004429481	0.058645551
AC013463.2	3.717980902	3.475105277	0.001211515	0.028546542
TTLL7-IT1	3.717695439	2.87866726	0.001664638	0.033998787
C6orf147 /// KHDC1	3.714785185	2.245706133	0.004429481	0.058645551
LCAT	3.712800382	2.808556037	0.00218621	0.038849044
B3GALT1	3.712547621	3.660229733	0.000608184	0.020256018
LMO7DN	3.712143658	3.074899497	0.002176202	0.038849044
ZNF850	3.711778409	3.25192381	0.001248936	0.029078292
RPP14	3.710195757	3.588565468	0.000780726	0.022635483
KLHL26	3.708201134	3.630616273	0.000717575	0.02158421
BPIFA1	3.706249228	4.869810098	0.000193096	0.011215713
KRT9	3.706043257	3.953393873	0.000451574	0.017555101
KIAA0319L	3.705144623	3.588473534	0.000780726	0.022635483
GPR75	3.705106785	3.575073637	0.0008504	0.023670138
FAM110D	3.704684505	3.434019504	0.00132779	0.0300216

AK024936	3.703647339	3.140918615	0.001732606	0.034862167
FAM19A4	3.70318445	3.028993542	0.002446565	0.041860671
BC040412 /// CTC-400I9.1	3.702845017	4.637394218	0.000296957	0.013911006
KLK6	3.702542453	3.676480641	0.000608184	0.020256018
E2F8	3.700852029	2.581718435	0.004569388	0.058850908
LOC101928865	3.698336019	2.985712411	0.00112921	0.027319677
CYSLTR2	3.694892538	4.004450019	0.000667078	0.021170904
MRPL12	3.694773463	4.471459376	0.000344607	0.015002232
SFTPA1 /// SFTPA2	3.694133946	3.219564703	0.001390333	0.031017767
MUC3B	3.69316291	4.233652109	0.00051512	0.018706971
A1BG	3.690616314	4.02040377	0.00062539	0.020376796
TMEM255A	3.689404095	3.297601709	0.001013156	0.025675357
BC041998	3.68778002	2.088507459	0.008203511	0.084939983
MMP21	3.684651947	2.715817994	0.002906428	0.0463016
RP11-2E11.9	3.683365757	3.708455896	0.000517642	0.018706971
BC041451 /// RP1-206D15.3	3.682976357	2.283781613	0.004429481	0.058645551
CASC6	3.68061586	2.512310181	0.006295194	0.072628703
OR4D1	3.676372812	3.702158293	0.000560802	0.019220167
MTHFD1L	3.67439153	2.753292993	0.002516721	0.042380758
FURIN	3.671736616	3.571768232	0.0008504	0.023670138
AF070581	3.668898342	2.448263755	0.007434817	0.079735439
UBL5	3.66888686	7.092901855	0.000100496	0.008908833
NR2E1	3.668864624	2.555088439	0.005352624	0.065287566
FLNB	3.667363374	3.854479907	0.000641766	0.020766359
AC009237.8 /// AK055967	3.666942583	4.481802144	0.000344607	0.015002232
CYP2S1	3.665171235	2.957064657	0.001281795	0.029320273
LY6G6E	3.664732278	3.21052821	0.001390333	0.031017767
DES	3.664723318	4.40701377	0.000440359	0.017384807
BC043355 /// RP11-481J13.1	3.664186476	3.616773665	0.000717575	0.02158421
NMUR2	3.664005252	3.784802289	0.000800201	0.022821694
RGS18	3.663618533	3.175804246	0.001550588	0.032822782
GABRA3	3.66286098	3.828525103	0.000690159	0.021170904
GRTP1-AS1	3.662153146	1.571863886	0.034780972	0.205039552
C2CD5	3.66124473	2.327163735	0.00365261	0.051799007
HYI-AS1 /// HYI-AS1	3.660565808	3.758807231	0.000862769	0.023929359
MIR3185 /// PRAC2	3.660422057	4.475812364	0.000344607	0.015002232
NCKAP5	3.656239486	2.199015625	0.005404101	0.065480303

SGCG	3.656239486	2.199015625	0.005404101	0.065480303
LOC101927606	3.65474364	3.043656236	0.002446565	0.041860671
PCDHB17	3.65403567	3.132918408	0.001732606	0.034862167
LOC101928968	3.652190208	2.864838695	0.001904897	0.036328095
RP11-324J3.1	3.65130594	3.165033401	0.001550588	0.032822782
FSD2	3.651148713	3.579451684	0.0008504	0.023670138
ZNF362	3.649719513	3.98322167	0.000712016	0.021499589
HAND2-AS1	3.64717828	3.161426216	0.001732606	0.034862167
LOC101927044	3.645871911	2.940451663	0.001458763	0.031878391
SPAG11A	3.645574565	3.125540712	0.001939802	0.036328095
NAV2-AS5	3.645160049	3.438868803	0.00132779	0.0300216
ZNF189	3.644751748	4.708934145	0.000361543	0.015166709
RP11-394I13.2	3.644278559	2.123576691	0.006635587	0.074656714
LOC283713 /// OTUD7A	3.641157383	2.369338506	0.008819448	0.0893286
RP3-522P13.1	3.64100647	2.936270232	0.001458763	0.031878391
RP11-443C10.1	3.640507515	4.194041709	0.000577	0.019559727
HIST1H3A /// HIST1H3B /// HIST1H3C /// HIST1H3D /// HIST1H3E /// HIST1H3F /// HIST1H3G /// HIST1H3H /// HIST1H3I /// HIST1H3J	3.638139891	3.460671619	0.001211515	0.028546542
SNX31	3.63763938	3.714934115	0.001005703	0.025675357
RNF2	3.636098036	3.513390859	0.001012527	0.025675357
PPEF2	3.633116064	3.043315731	0.002446565	0.041860671
DPPA2	3.632493661	3.182295246	0.001550588	0.032822782
GALNT13	3.632429066	3.733480737	0.000931068	0.024755296
LOC142937	3.631756336	3.369302851	0.00176232	0.034862167
DEFT1P /// DEFT1P2	3.631571122	3.843691472	0.000641766	0.020766359
RP5-892K4.1	3.629905694	3.396779546	0.001601365	0.033409649
KIR2DS3	3.629657697	3.507657017	0.00110686	0.027284747
HSD17B11	3.629444792	3.610874298	0.000780726	0.022635483
CNDP1	3.62931063	2.621834192	0.003915642	0.054412443
VIP	3.628710824	2.964604524	0.003112808	0.048204405
LEFTY1	3.627879058	3.682576872	0.001176763	0.028065499
DSG4	3.6258445	1.813243538	0.020717199	0.148245028
EGFL6	3.62469687	3.420721249	0.001457173	0.031878391
PLAT	3.621927596	3.069721261	0.002176202	0.038849044

OR51B6	3.620215996	3.171572157	0.001550588	0.032822782
CCP110	3.620063502	3.344211753	0.001942282	0.036328095
MCHR2	3.619708164	4.225125391	0.00051512	0.018706971
GCM1	3.617437343	3.35665793	0.00176232	0.034862167
ZNF646	3.614707727	4.08317903	0.000517304	0.018706971
MMGT1	3.614308674	2.952225472	0.003523399	0.051251816
LINC00555	3.614268085	2.656646053	0.003915642	0.054412443
MESDC1	3.613835279	3.353480244	0.00176232	0.034862167
TMSB4Y	3.613649821	3.827531771	0.000690159	0.021170904
LOC101929078	3.613244763	3.783789277	0.000800201	0.022821694
PTGER2	3.612917024	2.164784434	0.006635587	0.074656714
CLDN17	3.609531284	4.096366505	0.000486202	0.01836311
LOC100130429	3.608793691	3.37811732	0.00176232	0.034862167
BC042029	3.607961362	2.398522843	0.008819448	0.0893286
LGALS2	3.607044122	4.354210388	0.000539589	0.019150697
HTR7	3.606772162	3.181863435	0.001550588	0.032822782
ZNF34	3.604626741	2.943917086	0.003523399	0.051251816
RHNO1	3.604250963	2.844601137	0.001904897	0.036328095
LDHD	3.60279738	3.706884079	0.001087349	0.027076494
GNMT	3.602196218	2.646560064	0.003915642	0.054412443
RP11-24P14.1	3.6006201	2.392564695	0.008819448	0.0893286
EIF1AY	3.600450436	3.399272006	0.001601365	0.033409649
LOC100506405	3.598145094	2.733539858	0.002906428	0.0463016
AX747135 /// RP11-863K10.7	3.598139205	2.859719781	0.001904897	0.036328095
EPB41L4A-AS2	3.598134284	2.976187787	0.003112808	0.048204405
LINC00462	3.597074991	3.556822345	0.000927373	0.024755296
C17orf53	3.596843145	4.866021293	0.000304643	0.013911006
C11orf52 /// HSPB2 /// HSPB2-C11orf52	3.596702646	3.117464216	0.001939802	0.036328095
CTSLP8	3.596346339	3.790436661	0.000800201	0.022821694
PRG4	3.593381054	2.972104311	0.003112808	0.048204405
LOC100128288	3.591746884	3.526499971	0.001012527	0.025675357
PLA1A	3.590022485	2.636407877	0.003915642	0.054412443
SPINK7	3.589333676	3.076700431	0.002176202	0.038849044
AK055055 /// RP11-752D24.2	3.587218287	3.965463681	0.000760495	0.022444169
CTB-92J24.2	3.585719077	3.780890471	0.000800201	0.022821694
IBSP	3.58403413	3.832579784	0.000690159	0.021170904
ZNF233	3.583266758	3.122698014	0.001939802	0.036328095
RP4-813F11.4	3.582217889	2.483628082	0.006295194	0.072628703

MAP7D2	3.581732979	3.170921906	0.001550588	0.032822782
C2CD2L	3.580640585	3.567344182	0.000927373	0.024755296
KLHL14	3.580333758	3.427831951	0.001457173	0.031878391
MIR6824 /// SLC26A6	3.580155	3.352873476	0.001942282	0.036328095
IQCH-AS1	3.58004407	4.329575398	0.00059879	0.020128424
CCDC39 /// LOC101928882	3.579656089	3.489750122	0.001211515	0.028546542
DQ599616	3.578843616	5.636368052	0.000191803	0.011215713
CEACAM7	3.578666988	2.856540849	0.001904897	0.036328095
ISOC2	3.578553781	3.407907865	0.001601365	0.033409649
LOC101930578 /// TPTE2P2	3.57846496	2.802149746	0.00218621	0.038849044
FAM179B	3.578369478	2.374551728	0.008819448	0.0893286
LOC101927150	3.577935381	3.661447823	0.001274794	0.029320273
ARRDC2	3.575915225	4.481129819	0.000344607	0.015002232
CILP	3.574224144	4.339089847	0.000568298	0.019404732
AC106801.1	3.572686503	3.195188059	0.001550588	0.032822782
LOC101927021	3.572323959	3.76887219	0.000862769	0.023929359
BRIX1	3.572269283	2.313802252	0.010510413	0.098019301
EBF4	3.571775416	4.197809421	0.000577	0.019559727
C16orf54	3.571541866	1.998647523	0.010215989	0.097518902
OR51I1	3.571303106	3.559041344	0.000927373	0.024755296
LINC00856	3.571007013	4.810634844	0.000368173	0.015417344
CPS1-IT1	3.565362443	2.615901446	0.004569388	0.058850908
OR12D3	3.564407984	2.307507099	0.010510413	0.098019301
HMGB1P17 /// HMGB1P17	3.56288828	2.747062826	0.002906428	0.0463016
BIN2	3.561777754	4.188732516	0.000611145	0.020256018
RDH12	3.561483276	2.82859715	0.00218621	0.038849044
GP5	3.558859369	2.997417502	0.002756531	0.04462882
N6AMT2	3.558803303	3.756757471	0.000931068	0.024755296
TRMT112	3.558123377	6.725297354	0.000154143	0.010375748
ECI1	3.554064057	4.148980446	0.000686704	0.021170904
RASD2	3.553778002	4.36403479	0.000539589	0.019150697
LOC440028	3.548438335	2.776807678	0.002516721	0.042380758
CASP7	3.547663601	4.175931541	0.00064765	0.020899176
ANXA2P3	3.547411162	3.295120229	0.002369975	0.041241075
DKFZP434L187	3.547315597	2.951171365	0.003523399	0.051251816
C21orf119	3.545983534	3.561256494	0.000927373	0.024755296
KRT79	3.545342392	3.700794245	0.001087349	0.027076494
LOC100506922	3.544967028	2.930662166	0.003523399	0.051251816
TMEM51	3.544967028	2.930662166	0.003523399	0.051251816

BLID	3.544654649	3.868560405	0.001068447	0.026816186
LOC100507391	3.543725171	3.262335977	0.001248936	0.029078292
DCUN1D3	3.54191064	3.980908712	0.000760495	0.022444169
CNTNAP3	3.540035054	2.926455917	0.003523399	0.051251816
DCD	3.539932699	3.101858848	0.002176202	0.038849044
VN1R1	3.539882329	3.198071071	0.001550588	0.032822782
LOC645261	3.53985236	3.258954998	0.001248936	0.029078292
NASP /// NASP	3.539625378	3.844014905	0.000690159	0.021170904
LOC102723990 /// TMEM53	3.539425039	4.716279326	0.000361543	0.015166709
BC040577 /// RP11-10K16.1	3.535898321	3.225364765	0.001390333	0.031017767
RBM24	3.534277821	2.680043361	0.003367649	0.050071404
FANCG	3.532412924	3.935071786	0.000869392	0.024056245
OR2L1P	3.53237547	2.253373696	0.004429481	0.058645551
RPL13A /// RPL13AP5 /// RPL13AP6 /// SNORD32A /// SNORD33 /// SNORD34 /// SNORD35A	3.529793811	3.471635087	0.001211515	0.028546542
LOC101928669 /// LOC644450	3.52862721	3.307665929	0.002143838	0.038830418
IL15RA	3.525731007	3.984628469	0.000760495	0.022444169
CDCA7	3.523033759	3.136124214	0.001939802	0.036328095
RP5-856G1.1	3.522330002	3.887775189	0.001068447	0.026816186
LINC00382	3.521482221	4.184247065	0.000611145	0.020256018
CRYGC	3.51984482	3.885545204	0.001068447	0.026816186
LOC101928173	3.51976389	3.462788341	0.00132779	0.0300216
CD3EAP	3.519024158	2.83235099	0.00218621	0.038849044
LA16c-380H5.5	3.518301094	4.099984138	0.000821282	0.023189378
BHLHE23	3.514087275	3.974150267	0.000760495	0.022444169
TMEM200C	3.514023985	3.418653115	0.001457173	0.031878391
SFN	3.513953132	9.810975795	0.000132194	0.009926277
MIR22 /// MIR22HG	3.510400161	4.407975011	0.000463028	0.017818504
OR1F1	3.508966933	4.337535386	0.00059879	0.020128424
FKBP10	3.508442053	3.041272606	0.002446565	0.041860671
SEPSECS-AS1	3.50828614	2.568743963	0.005352624	0.065287566
LOC102723932	3.506684354	2.204025365	0.005404101	0.065480303
LINC00682	3.505925316	2.471521155	0.007434817	0.079735439
LOC101929034	3.50372189	4.018353848	0.000667078	0.021170904
BBOX1	3.503275712	4.000326259	0.000712016	0.021499589

FZD6	3.501020783	2.929954536	0.003523399	0.051251816
BC035096 /// RP11-433J8.1	3.500747357	3.366890358	0.00176232	0.034862167
LOC101929279	3.500747357	3.366890358	0.00176232	0.034862167
LOC101928963	3.499975473	3.338873447	0.001942282	0.036328095
AL928742.12	3.497759341	3.702267672	0.001087349	0.027076494
ACN9	3.497732118	1.550381726	0.045799632	0.240968635
PTPN20B	3.497732118	1.550381726	0.045799632	0.240968635
ALDH1A1	3.497336942	2.414606503	0.008819448	0.0893286
CTC-203F4.2 /// H2AFY	3.493043888	4.496451697	0.000534505	0.019112011
LOC101928464	3.491179791	4.830472613	0.000502267	0.018581509
DPT	3.490319571	3.264663655	0.002624146	0.043412985
SMNDC1	3.488747638	2.552700243	0.005352624	0.065287566
RP11-649A18.7	3.486216597	3.151568173	0.001732606	0.034862167
USP35	3.485986123	2.639940478	0.003915642	0.054412443
RP11-326I11.5	3.485506634	3.021619097	0.002756531	0.04462882
BC039357 /// RP11-661G16.2	3.482646418	3.351052916	0.001942282	0.036328095
LOC100129175	3.482004581	3.147931892	0.001732606	0.034862167
C2orf83	3.481486863	4.312629139	0.000665641	0.021170904
HIST1H1E	3.481239635	3.831506469	0.001230541	0.028878999
PWAR5	3.479650895	2.759713721	0.002906428	0.0463016
ZNF256	3.478725064	3.706779533	0.001087349	0.027076494
LOC100505774	3.478645703	4.828994179	0.000502267	0.018581509
ACOT4	3.477776898	2.448681615	0.007434817	0.079735439
PSG2	3.477774485	2.121795287	0.008203511	0.084939983
SLC30A2	3.476650172	3.450328752	0.00132779	0.0300216
ARL8A	3.473017873	4.360496094	0.000539589	0.019150697
TICAM1	3.472465561	2.831620107	0.005186874	0.063963283
C8orf66	3.471973244	2.712782396	0.003367649	0.050071404
LOC283737	3.471568301	3.009705251	0.002756531	0.04462882
CACNG3	3.468231023	3.819895177	0.001322163	0.0300216
ATHL1 /// LOC101930595	3.466395107	4.102534909	0.000821282	0.023189378
GIF	3.464315485	3.335049404	0.001942282	0.036328095
GDAP2	3.46416462	3.606252639	0.001500646	0.03246148
BST2	3.463585313	3.873373294	0.001146185	0.027503751
RP11-469M7.1	3.460702134	1.982233284	0.012821408	0.110735482
LOC101928433	3.45735388	3.273910126	0.002624146	0.043412985
UBE2C	3.456826971	4.217855103	0.000924518	0.024755296
VSTM1	3.454917152	3.028826838	0.002756531	0.04462882
SHOX	3.45185938	3.61775657	0.001500646	0.03246148
KRT19	3.451392319	3.042203267	0.002446565	0.041860671

SCNN1B	3.449624088	3.879621357	0.001068447	0.026816186
POP1	3.448034252	2.989641445	0.003112808	0.048204405
LOC283484	3.447277607	2.399416983	0.008819448	0.0893286
GEMIN6	3.446549435	3.667565908	0.001274794	0.029320273
PCMTD2	3.445106195	3.687540832	0.001176763	0.028065499
USP13	3.44200648	3.847827412	0.001230541	0.028878999
BX538226 /// RP11-438B23.2	3.441304233	4.318134502	0.000631192	0.020508941
UCP1	3.441257749	3.99688166	0.001193496	0.028406889
HDDC3	3.441155317	4.744341995	0.000484745	0.01836311
RP11-285E9.5	3.441014824	3.926684013	0.000930551	0.024755296
FAM84A	3.438985159	4.257159736	0.00078281	0.022635483
LOC101928157	3.437138234	2.261851043	0.012586629	0.109472446
MTCL1	3.43462032	3.534422257	0.001932181	0.036328095
IL23A /// TCRBV6S2A1N1T	3.433512632	3.196140046	0.003233264	0.049354021
ZNF711	3.433140441	2.056867735	0.010215989	0.097518902
NAP1L3	3.431686275	3.009016758	0.002756531	0.04462882
TCF7L1	3.430721175	3.305813557	0.002369975	0.041241075
ZNF234	3.429904101	2.1729338	0.006635587	0.074656714
TRPM4	3.429407691	4.321074682	0.000631192	0.020508941
TMEM185A	3.429110072	3.888942756	0.001068447	0.026816186
CCDC106	3.428617691	3.683452291	0.001176763	0.028065499
FGF19	3.428327933	3.408007139	0.001601365	0.033409649
FAM131B	3.424970561	3.52594169	0.002106798	0.038575922
CECR5-AS1	3.423991578	4.38271719	0.000790067	0.0228013
SV2B	3.42286233	4.310626975	0.000665641	0.021170904
AK025975 /// RP1-74M1.3	3.422160291	3.452141738	0.00132779	0.0300216
LOC101928961	3.42120004	3.185525723	0.003233264	0.049354021
SPATA31E1	3.418647925	2.66857672	0.003915642	0.054412443
IL17F	3.416545645	2.707172632	0.003367649	0.050071404
COMMD8	3.415975339	1.580014843	0.034780972	0.205039552
C14orf93	3.415255822	3.517413554	0.002106798	0.038575922
PPCDC	3.4151114	3.650432237	0.001382397	0.031017767
CC2D1B	3.414680293	3.5626234	0.001774082	0.034862167
TGM1	3.412884173	3.08854779	0.002176202	0.038849044
ABL1	3.412292434	4.508676352	0.00075956	0.022444169
LOC101928012	3.411572002	3.900414013	0.000996744	0.025675357
KRTAP10-11	3.410670092	3.392602907	0.00176232	0.034862167
REPIN1 /// REPIN1	3.410577572	4.176233108	0.001096763	0.027121211
LOC101059948	3.409071536	3.259822613	0.002624146	0.043412985

RUNDC1	3.409049934	2.79727737	0.005932006	0.069745944
LOC100507006	3.405539919	4.244105588	0.000827053	0.023222371
ADCY10P1	3.403932348	3.866520939	0.001146185	0.027503751
HOXC8	3.403281328	4.199068053	0.000978199	0.025675357
HAND1	3.402556047	3.410476861	0.001601365	0.033409649
LZTS3	3.398796672	4.150745705	0.001162234	0.027860418
ZNF506	3.39793708	4.134909256	0.000773287	0.022635483
RPS29P28 /// RPS29P28	3.395723372	3.590295518	0.001630752	0.033902317
C2orf76	3.395073947	3.073335811	0.002446565	0.041860671
CXCL16	3.394300025	2.648516983	0.003915642	0.054412443
NRG4	3.392896228	2.687615375	0.003367649	0.050071404
CTR9	3.392155106	2.873622994	0.004547753	0.058830288
MFNG	3.390862246	2.963379745	0.003523399	0.051251816
C6orf118	3.390688507	2.836867327	0.005186874	0.063963283
NOA1	3.389139888	2.799196825	0.005932006	0.069745944
SUMO3	3.388879343	6.607502455	0.000281484	0.013582158
PTTG3P	3.388447107	3.398187881	0.00176232	0.034862167
ZFYVE21	3.385078768	4.32800782	0.000969613	0.025536037
A1CF	3.383160776	4.003539773	0.001193496	0.028406889
ANXA8 /// ANXA8L1 /// LOC100996760 /// LOC102725207	3.380025384	4.381577761	0.000831057	0.023297366
C7orf49 /// LOC653739	3.379810419	3.835959917	0.001322163	0.0300216
LOC101927095	3.379645594	2.162138827	0.018334706	0.136997112
DHFRL1	3.378952221	3.629140775	0.001500646	0.03246148
HSD11B1	3.378716988	4.275443712	0.00078281	0.022635483
FAT1	3.374428058	4.85783281	0.000643982	0.020809386
MGC4859	3.374336304	1.981314316	0.012821408	0.110735482
LOC285957	3.37367858	2.412952127	0.008819448	0.0893286
LOC102723645	3.37190099	2.821167484	0.005186874	0.063963283
AC007967.3 /// RBMY2TP /// RBMY2TP	3.370105898	4.959366479	0.000610281	0.020256018
COQ3	3.369877655	4.499698118	0.000795707	0.022821694
C15orf54	3.369874009	1.628777035	0.034780972	0.205039552
CCKBR	3.367991897	3.740678546	0.001776932	0.034862167
KRT13	3.367402726	4.251628348	0.000827053	0.023222371
HOXC11	3.366695775	4.548156048	0.000692814	0.021170904
CHL1	3.366663398	2.383665455	0.008819448	0.0893286
TOE1	3.366498923	3.842367205	0.001230541	0.028878999

DDX51	3.365703793	3.098747445	0.002176202	0.038849044
DRG2	3.364358966	3.573636226	0.001774082	0.034862167
SPRED3	3.362824837	4.464032571	0.000916702	0.024755296
TWISTNB	3.361090554	3.245609227	0.002910357	0.0463016
PRDM8	3.360020008	3.147458414	0.004011765	0.054539569
MED29	3.356920963	3.26867825	0.002624146	0.043412985
TIMMDC1	3.353142219	3.238771281	0.002910357	0.0463016
MORC3	3.352161761	3.462262821	0.002513798	0.042380758
HSPB2	3.350259379	4.046186236	0.001051105	0.026579731
DEGS2	3.350223763	2.766840598	0.006803492	0.075213009
MCHR2-AS1	3.347056852	3.46934542	0.002513798	0.042380758
BARX2	3.345855504	4.23232223	0.000924518	0.024755296
MSI1	3.344335697	2.607600737	0.004569388	0.058850908
MRPL13	3.343614436	2.241381597	0.015150304	0.122275726
C2CD4A	3.342441025	1.827692675	0.020717199	0.148245028
RNF126	3.341775957	3.714332638	0.001917416	0.036328095
LOC102724162	3.338693629	2.720166822	0.007826126	0.082555619
INSL4	3.338541439	3.955616589	0.001451105	0.031878391
SPICE1	3.338223532	2.642686542	0.003915642	0.054412443
PRRT2	3.336925579	3.197733045	0.003233264	0.049354021
LOC285902 /// ZNF273 /// ZNF479 /// ZNF733P	3.33601695	2.33535336	0.010510413	0.098019301
BC040311	3.334136419	2.127114905	0.022314819	0.155835874
LOC51145	3.333556361	2.070283331	0.010215989	0.097518902
LOC101927040	3.33333537	3.248385925	0.002910357	0.0463016
RTN4RL1	3.33333537	3.248385925	0.002910357	0.0463016
LINC01111	3.332951435	3.865944158	0.001146185	0.027503751
RPRML	3.32837844	3.696064573	0.0020709	0.038306756
IGH /// IGHA1 /// IGHG1 /// IGHG2 /// IGHG3 /// IGHM /// LOC102725526	3.325143757	2.591974218	0.005352624	0.065287566
SLC38A5	3.324849101	3.992051804	0.001272992	0.029320273
RTP4	3.321396385	3.23813802	0.002910357	0.0463016
CCL7	3.320319523	2.667038894	0.003915642	0.054412443
KEL	3.31944515	4.181036829	0.001096763	0.027121211
DUSP12	3.318475094	3.287462112	0.002369975	0.041241075
ZNF749	3.317613407	2.811388925	0.005932006	0.069745944
PLEKHH1	3.317395041	2.368181183	0.010510413	0.098019301
GAFA2	3.317024547	4.590409533	0.000873346	0.024108864

SERPINA4	3.315813099	3.999874649	0.001272992	0.029320273
LOC101929319	3.314375901	3.934190636	0.001550881	0.032822782
LOC101928259	3.312303363	2.316678686	0.012586629	0.109472446
PIAS3	3.312303363	2.316678686	0.012586629	0.109472446
BANF1	3.312269433	4.132130662	0.001307232	0.029844021
SERPINI1	3.312089648	3.002955565	0.003112808	0.048204405
C7orf13 /// LINC01006	3.310677822	3.880706525	0.001901354	0.036328095
RP11-416I2.1	3.307036301	4.437388853	0.001009214	0.025675357
MRPL22	3.30586996	2.359070051	0.010510413	0.098019301
LOC285847	3.305621019	3.554384411	0.001932181	0.036328095
ADAM3B /// ADAM3B	3.304376386	2.53409009	0.006295194	0.072628703
MDGA2	3.30359483	2.403271595	0.008819448	0.0893286
IL12RB2	3.303242083	4.035241097	0.001119686	0.027319677
LOC100507634	3.302400917	2.130208973	0.022314819	0.155835874
SOHLH1	3.302227158	3.673120121	0.002238765	0.039633051
ZMAT4	3.300912651	3.082747909	0.005015008	0.062599664
LOC285627	3.300806205	2.208140348	0.015150304	0.122275726
TMEM186	3.300661285	3.528779327	0.002106798	0.038575922
GRIK4	3.29986248	2.400309191	0.008819448	0.0893286
GCNT2	3.298977699	6.121892817	0.000468284	0.017829708
CDKN2C	3.297669507	3.860686654	0.001230541	0.028878999
SGOL1	3.296242889	3.190031631	0.003598289	0.051799007
ARL11	3.295565295	3.330078422	0.002143838	0.038830418
PPBPP2	3.295006755	3.458142261	0.002751	0.04462882
THAP4	3.294764716	4.420562551	0.0011127	0.027319677
SERPINA9	3.294747732	3.916827706	0.001658676	0.033998787
FKBPL	3.289731972	4.083063686	0.00156584	0.033079785
LOC100507389	3.289051075	3.058210273	0.005015008	0.062599664
LOC101927093	3.288577468	4.554264785	0.000998144	0.025675357
S100PBP	3.288193472	2.297776916	0.012586629	0.109472446
C1orf115	3.285142528	4.370475399	0.000874528	0.024113156
CD4	3.283850459	2.884485806	0.004547753	0.058830288
CLEC4C	3.283320802	2.194642322	0.018334706	0.136997112
LOC100287525	3.283018221	3.555678514	0.001932181	0.036328095
PRSS36	3.282266816	2.850232017	0.005186874	0.063963283
LOC101928877	3.279344035	2.554887903	0.005352624	0.065287566
RAB36	3.278970339	4.291024151	0.001135603	0.027417875
SYT2	3.278576816	3.652429407	0.002422557	0.041860671
GABRA2	3.278019264	2.44210789	0.008819448	0.0893286
DHRS11	3.277263305	2.382415156	0.010510413	0.098019301
MAD1L1	3.277042291	4.326736373	0.001021612	0.025861756
SCRN2	3.276075669	3.337510514	0.002143838	0.038830418

RP11-353N14.2	3.275198746	2.909498264	0.00399788	0.054449116
HTRA3	3.272701543	4.03836915	0.001771198	0.034862167
DAPL1	3.270331325	3.810731136	0.001421769	0.031688985
COMMD5 /// LOC101928879 /// ZNF250	3.270324512	3.575535699	0.001774082	0.034862167
UBE2E2	3.269997647	6.356241064	0.000447446	0.017460803
UCN3	3.269650888	2.376404388	0.010510413	0.098019301
LCOR	3.269066541	3.989633058	0.001358678	0.03063485
LOC100129884	3.269014925	3.522548118	0.002106798	0.038575922
PMS2P4	3.268198276	2.871461184	0.004547753	0.058830288
LOC101928304	3.267774676	4.216473416	0.001491156	0.03246148
OAS1	3.26679884	3.671038273	0.002422557	0.041860671
KRT24	3.266637849	1.901402998	0.016225581	0.127354276
SLCO4A1-AS1	3.266637849	1.901402998	0.016225581	0.127354276
HSD3B1	3.26612598	3.150946033	0.004011765	0.054539569
TBC1D8B	3.265849218	3.324722787	0.002143838	0.038830418
ASUN	3.26183618	3.21331729	0.003233264	0.049354021
DEPDC4	3.26183618	3.21331729	0.003233264	0.049354021
MICB	3.26183618	3.21331729	0.003233264	0.049354021
HPD	3.26001708	3.048196442	0.005623714	0.067405742
FAM227B	3.258438781	2.476343789	0.007434817	0.079735439
PIP5K1C	3.257131237	4.787976889	0.000871107	0.024075339
NPPC	3.256642282	3.129346171	0.004481104	0.058678986
MYBBP1A	3.256267321	4.199629394	0.001576883	0.03322345
GPRASP1	3.256090502	2.986175329	0.003523399	0.051251816
GP9	3.255192143	4.713410104	0.000803742	0.022821694
STAB2	3.254386487	3.411805907	0.003307485	0.049999632
LINC00482	3.253540962	4.108414633	0.00138752	0.031017767
AACS	3.248337657	5.083607256	0.00073805	0.0221717
LOC102724017	3.248266954	3.066813494	0.005015008	0.062599664
ABCC6P1	3.247701092	2.167289731	0.018334706	0.136997112
SYCN	3.247436637	2.787800195	0.005932006	0.069745944
TM4SF20	3.246569626	2.358231327	0.010510413	0.098019301
FUCA1	3.245657017	2.868919138	0.005186874	0.063963283
COL8A2	3.245416282	3.79753756	0.001530156	0.032822782
C9orf37	3.244366732	3.904916256	0.001775228	0.034862167
TMEM121	3.243195781	3.592146916	0.001774082	0.034862167
CTD-3080P12.3	3.242929572	3.98187813	0.001358678	0.03063485
ZNF514	3.240646061	3.85264647	0.002186027	0.038849044
KCNT2	3.239025022	2.780877692	0.006803492	0.075213009
IFNA2	3.238847703	2.43963614	0.008819448	0.0893286
LOC100507642	3.238186748	1.684502665	0.034780972	0.205039552

LOC101929268	3.236339783	4.580014169	0.000912824	0.024755296
GUCY1B2	3.234330886	3.896091321	0.001901354	0.036328095
DOK4	3.232847083	3.546390817	0.002106798	0.038575922
RP4-758J24.5	3.232460545	4.017660414	0.001885463	0.036328095
LINC00842	3.231400047	2.904527015	0.004547753	0.058830288
FAM206A	3.231322565	3.262806899	0.002910357	0.0463016
RP11-184E9.2	3.229286661	3.89165942	0.001901354	0.036328095
PIH1D1	3.228508617	4.762303339	0.000941601	0.024854042
TMEM47	3.227877647	2.430943986	0.008819448	0.0893286
LOC100996758 /// NPY4R	3.227613946	3.841213235	0.002186027	0.038849044
AK021933	3.227375414	3.144837768	0.004011765	0.054539569
PRKCQ	3.225756924	3.195482374	0.003598289	0.051799007
FAM225A /// FAM225B	3.225331536	3.047517974	0.005623714	0.067405742
TNFRSF11A	3.224640961	3.805036204	0.002521124	0.042380758
RP11-121C2.2	3.224475074	3.351957021	0.003998158	0.054449116
PDCL	3.222384615	4.847168659	0.000971264	0.025550888
MAGI2-AS2	3.2205986	2.660506044	0.009030241	0.0893286
POU2F3	3.220029408	2.910556534	0.004547753	0.058830288
RGS7BP	3.21907877	1.739167978	0.026706022	0.172890711
SNHG19	3.218694729	6.357636169	0.000511429	0.018706971
MED11	3.217147785	4.321854028	0.001585188	0.033338612
MYBPC2	3.216930772	2.796244092	0.005932006	0.069745944
ECSCR	3.215446145	3.048585837	0.005623714	0.067405742
GAGE1 /// GAGE12B /// GAGE12C /// GAGE12D /// GAGE12E /// GAGE12F /// GAGE12G /// GAGE12H /// GAGE12I /// GAGE12J /// GAGE13 /// GAGE2A /// GAGE2B /// GAGE2C /// GAGE2D /// GAGE2E /// GAGE3 /// GAGE4 /// GAGE5 ///	3.213886181	3.272259814	0.002624146	0.043412985

GAGE6 /// GAGE7 /// GAGE8				
IKBKAP	3.213703732	2.618164186	0.010453099	0.098019301
PLEKHA7	3.21294497	2.579891163	0.012140675	0.106947596
ISY1-RAB43 /// RAB43	3.212851294	4.127656432	0.001307232	0.029844021
LOC101060609	3.21186616	3.53498801	0.002106798	0.038575922
CDCA4	3.209483011	3.572780379	0.003355072	0.050071404
AC005838.2	3.20875811	2.537920077	0.006295194	0.072628703
PTPRG	3.208048445	2.797203815	0.005932006	0.069745944
LOC101928767	3.207755914	2.7552268	0.006803492	0.075213009
TMEM161B-AS1	3.206774601	2.456059171	0.007434817	0.079735439
GLS2	3.205039021	2.423457193	0.008819448	0.0893286
GSN-AS1	3.204786676	2.233058211	0.015150304	0.122275726
OR51A1P /// OR51A1P	3.204776203	3.215519686	0.003233264	0.049354021
FXYD1	3.203446315	4.211246106	0.001576883	0.03322345
ERVK3-1 /// ZNF8	3.202782943	4.184983175	0.00166836	0.033998787
USH1G	3.200720533	4.183159655	0.00166836	0.033998787
CTD-2366F13.2	3.200587456	3.135375259	0.004481104	0.058678986
KRTAP3-3	3.200257677	3.783723874	0.002710732	0.044469637
HCG26	3.196405282	4.450548252	0.001445739	0.031878391
PHACTR2	3.195668336	3.463393709	0.002751	0.04462882
CLEC18A /// CLEC18C	3.193380176	3.348169713	0.003998158	0.054449116
MPND	3.193169008	3.811075539	0.002521124	0.042380758
GAGE3	3.193155261	3.102697839	0.004481104	0.058678986
WEE1	3.192744493	4.169623103	0.001766028	0.034862167
HAVCR1P1	3.192607088	3.577707076	0.003355072	0.050071404
ZFAND2A	3.192428483	3.205054616	0.003233264	0.049354021
SCRN1	3.190080391	4.508137358	0.00119805	0.028486429
LOC101927849	3.189941461	2.173294608	0.018334706	0.136997112
TRMT10C	3.189439881	3.88847447	0.001901354	0.036328095
KRT83	3.188921203	4.008568035	0.002008336	0.037325816
CLEC1B	3.188591426	4.464977535	0.001378669	0.031017767
FBRSL1	3.187980606	3.752527717	0.001776932	0.034862167
AMDHD1	3.186911463	2.928496909	0.00399788	0.054449116
ROS1	3.185636147	3.692645947	0.002238765	0.039633051
RASSF10	3.183193489	1.900468866	0.016225581	0.127354276
RP11-546O6.4	3.183150902	2.308704887	0.012586629	0.109472446

ATF7IP2	3.183083563	3.607751441	0.002845074	0.045841213
ATP1A1	3.18126012	3.957785496	0.002436393	0.041860671
LILRA1	3.180420009	4.283331703	0.00176037	0.034862167
CCNT2-AS1	3.179815702	4.111992175	0.001473565	0.032142043
LOC101927131	3.17955331	4.00033922	0.002140559	0.038830418
LOC101060405 /// RRN3P3	3.178696119	3.11694094	0.004481104	0.058678986
CYBRD1	3.178523675	3.063545936	0.005623714	0.067405742
LOC102725198 /// RASA4 /// RASA4B /// RASA4CP	3.178269973	4.287390053	0.00176037	0.034862167
EPN2 /// EPN2-IT1	3.178180551	3.273658516	0.002624146	0.043412985
DIRAS3	3.17708942	3.464110342	0.002751	0.04462882
SLC35B1	3.175234415	3.087647191	0.005015008	0.062599664
PRDM11	3.17462018	3.332162338	0.004405204	0.058628437
LOC101929456	3.172695785	2.886653315	0.004547753	0.058830288
ZFP69B	3.171879871	1.892245668	0.016225581	0.127354276
HIST1H2AM	3.171796293	2.159494461	0.018334706	0.136997112
CCNE2	3.165705102	2.560928169	0.012140675	0.106947596
UBTD1	3.165570442	4.139144728	0.001981897	0.036997277
RP11-63A11.1	3.16463946	3.131047797	0.004481104	0.058678986
SELENBP1	3.16463946	3.131047797	0.004481104	0.058678986
PCTP	3.16311445	4.225632866	0.001491156	0.03246148
CTB-12A17.3	3.161419695	4.211795907	0.001576883	0.03322345
S100B	3.160886031	2.049989167	0.027322057	0.174558001
LOC101927228	3.160587155	2.936165173	0.00399788	0.054449116
AV8S2A1N1T /// hADV36S1 /// TCR-alpha /// TCR-alpha /// TCRA /// TRAC /// TRAJ20 /// TRAJ20 /// TRAV36DV7 /// TRAV36DV7 /// YME1L1	3.159271761	2.781705974	0.006803492	0.075213009
RP11-1260E13.2	3.159271761	2.781705974	0.006803492	0.075213009
LINC01069	3.158891215	4.052798371	0.001771198	0.034862167
LOC100133985	3.156216745	2.903101828	0.004547753	0.058830288
LCK	3.155061169	3.648178423	0.002624011	0.043412985
LOC101929787	3.151518513	3.67197284	0.002422557	0.041860671

PLA2G5	3.151139588	2.604470059	0.012140675	0.106947596
TRIM65	3.150364525	2.283337391	0.015150304	0.122275726
FSD1	3.149036997	3.805384334	0.002521124	0.042380758
TCN1	3.147836246	1.932006106	0.016225581	0.127354276
LOC374890	3.145547686	4.539011788	0.001526005	0.032822782
FAM218A	3.144533859	3.404845476	0.003307485	0.049999632
RP4-561L24.3	3.143704453	3.034461014	0.006319303	0.072628703
BDNF-AS	3.142975499	3.515449875	0.003973636	0.054449116
CCDC6	3.142911675	4.613197569	0.001171665	0.028029287
hsa-let-7a-3 /// hsa-let-7b /// hsa-mir-4763 /// RP4-695O20__B.10	3.142614438	3.112564833	0.004481104	0.058678986
MIA3	3.142086475	3.777595737	0.002917009	0.0463016
BC038205	3.14035373	2.403743802	0.008819448	0.0893286
PGM3	3.139767293	2.962026324	0.007116069	0.077573403
IGHV7-81 /// IGHV7-81	3.139658224	1.683527713	0.034780972	0.205039552
AC002059.10	3.139408591	4.334017102	0.001505204	0.032500237
PMVK	3.136481763	4.450240048	0.00151661	0.032656464
OCIAD2	3.135947701	4.29681995	0.00176037	0.034862167
C21orf128	3.135786593	3.365353276	0.003998158	0.054449116
LINC00309	3.135710824	2.03127355	0.027322057	0.174558001
C15orf52	3.135271173	3.428302788	0.003014449	0.047508741
RP11-429B14.4	3.135138354	4.615587515	0.001171665	0.028029287
ZNF30	3.134479572	2.999507997	0.007116069	0.077573403
SAPCD1-AS1 /// SAPCD1-AS1 /// XXbac-BPG32J3.18	3.133814504	4.016975644	0.002008336	0.037325816
KRTAP4-2	3.132468867	3.296345481	0.004860852	0.061355847
APOLD1 /// DDX47	3.131601086	2.588829497	0.012140675	0.106947596
MAP3K5	3.130891317	4.134523611	0.002101185	0.038575922
LINC01126	3.130208302	3.739662063	0.003141633	0.048522849
HOXA4	3.129745488	3.851983636	0.002186027	0.038849044
LOC101929122	3.128305112	3.380412489	0.003633928	0.051799007
TMEM257	3.127707971	3.597242784	0.003087939	0.048200573
DTD2	3.127353646	2.93754756	0.008030972	0.084337772
PHF14	3.12564376	1.915855198	0.016225581	0.127354276
DAB2	3.125029004	2.583580748	0.012140675	0.106947596

RP11-646E18.4	3.125029004	2.583580748	0.012140675	0.106947596
EMR3	3.124631592	3.398402592	0.003307485	0.049999632
LOC100507403	3.12457396	3.57604894	0.003355072	0.050071404
BRI3 /// FLJ30064	3.124468036	3.980734855	0.002282946	0.040173011
CD3E	3.124436479	4.128859119	0.002101185	0.038575922
SNX29	3.120963494	3.374167106	0.003633928	0.051799007
C9orf142	3.12038873	3.144366098	0.004481104	0.058678986
RP5-1065J22.8	3.119560816	2.872936598	0.005186874	0.063963283
LOC101928809	3.119236304	2.780964664	0.006803492	0.075213009
SP4	3.119177702	2.765076396	0.006803492	0.075213009
CTNND1 /// TMX2-CTNND1	3.118783371	4.198836817	0.00166836	0.033998787
LINC01310	3.117896431	4.11025589	0.00222885	0.039517083
RP11-410L14.2	3.117896431	4.11025589	0.00222885	0.039517083
LOC101060181 /// LOC101928574 /// ZNF44	3.116260379	2.165530066	0.018334706	0.136997112
TMSB15B	3.115966917	3.727337532	0.003386477	0.050129249
LOC100507577 /// LONP2 /// SIAH1	3.114461114	2.89811482	0.004547753	0.058830288
ADRB1	3.114394782	3.634006388	0.002845074	0.045841213
FAM188B /// INMT-FAM188B	3.114168894	3.869123029	0.002037955	0.037756813
NPY1R	3.111794336	2.5730297	0.012140675	0.106947596
SH3RF3	3.111300464	3.835955959	0.002346676	0.041048479
OLFM2	3.109535538	3.653351277	0.002624011	0.043412985
SLC46A2	3.107634217	4.485686288	0.001831372	0.035585267
SYT17	3.1074029	5.400972274	0.001000988	0.025675357
HEBP2	3.106982619	3.108177968	0.005015008	0.062599664
LOC101927124	3.104007479	2.889530352	0.004547753	0.058830288
CLCA3P	3.102771834	2.414697099	0.008819448	0.0893286
GTF3C5	3.101011864	3.01243439	0.006319303	0.072628703
COA6	3.100864199	3.479403138	0.00472768	0.060415632
KRTAP4-12	3.100342909	3.959693937	0.002436393	0.041860671
PLCXD2	3.100077255	2.811881597	0.005932006	0.069745944
OR6W1P	3.099855367	3.930679902	0.002780495	0.044893058
RPAP1	3.099478668	4.287552661	0.001856299	0.036039812
CCDC61	3.099472148	3.397808129	0.003633928	0.051799007
LINC00263 ///	3.097479102	3.289122126	0.004860852	0.061355847

PP7080				
LOC100289061	3.097083918	3.776413276	0.002917009	0.0463016
LOC101927406	3.096983301	2.794029673	0.006803492	0.075213009
BLK	3.096826349	3.963692989	0.002436393	0.041860671
PTTG2	3.096659144	1.58711595	0.045799632	0.240968635
UBALD1	3.096208201	3.526716526	0.003973636	0.054449116
PCDHB10 /// PCDHB9	3.095842899	3.394724929	0.003633928	0.051799007
LOC158434	3.094649136	3.790211776	0.002710732	0.044469637
RAMP3	3.094628662	4.65193249	0.001470585	0.032106863
MIR133A1HG	3.09431104	2.367862485	0.010510413	0.098019301
GTF2H4	3.092586815	2.59327566	0.012140675	0.106947596
FAM166A	3.091400188	3.575219699	0.003355072	0.050071404
KIF1C	3.089583639	3.498838239	0.004331767	0.057983971
TRPV2	3.087359766	3.768007537	0.002917009	0.0463016
FAM91A1	3.085055951	3.514032278	0.003973636	0.054449116
CADM2-AS1	3.085036006	3.25629323	0.005371775	0.065317664
LPCAT3	3.082794573	3.930207282	0.002780495	0.044893058
KDELC1	3.0827759	2.687185641	0.009030241	0.0893286
LL0XNC01-116E7.2	3.082190901	1.939093123	0.041756881	0.227810645
FAM174A	3.081869844	3.403362856	0.003307485	0.049999632
LOC643201	3.081103476	2.273932439	0.015150304	0.122275726
CLCA1	3.077984877	4.489064063	0.001831372	0.035585267
DNASE2B	3.075374902	3.081852627	0.005015008	0.062599664
SPIB	3.073760393	2.835497805	0.005932006	0.069745944
RAB3A	3.073647699	4.195330149	0.002436766	0.041860671
ITGB7	3.073419598	4.830568126	0.001434383	0.031878391
LOC100131262	3.07306965	2.743543077	0.007826126	0.082555619
FRMPD3	3.070833236	3.566705601	0.003649256	0.051799007
C17orf100	3.070179262	3.372966699	0.003998158	0.054449116
SNX17	3.069552328	4.566797401	0.001986653	0.037010667
KB-1836B5.1	3.068977853	3.432571064	0.003307485	0.049999632
TMEM52	3.066267122	3.449939711	0.005165949	0.063963283
SSSCA1-AS1	3.065854777	1.816213366	0.020717199	0.148245028
C9orf173	3.065179688	3.970962753	0.002436393	0.041860671
FAM169A	3.063450858	2.841807515	0.005932006	0.069745944
NLRP11	3.061722649	4.196721454	0.002436766	0.041860671
TAF1C	3.060127732	3.852300954	0.003651944	0.051799007
HADHAP1 /// HADHAP1	3.059625677	4.403077939	0.001754586	0.034862167
MT4	3.058519323	2.25668945	0.015150304	0.122275726
ARPC3	3.058115858	2.66740981	0.010453099	0.098019301
FLG	3.057735015	3.255665187	0.005371775	0.065317664

AC004158.3 /// AK055364	3.057607095	2.530093576	0.014149938	0.117459486
LOC101928697	3.055609551	1.625821304	0.034780972	0.205039552
LOC100133039	3.055402432	3.691760502	0.003945405	0.054449116
TMOD2	3.055246529	2.835133693	0.005932006	0.069745944
LOC100131655	3.054970081	3.114293992	0.005015008	0.062599664
SH2B1	3.054514009	4.105640417	0.002365563	0.041241075
FAM43A	3.054114626	3.778736239	0.002917009	0.0463016
PCDHA2	3.053710542	3.533864544	0.003973636	0.054449116
CPSF4	3.053428375	5.104650368	0.001514354	0.032637799
LINC00911	3.053113245	4.141352958	0.002101185	0.038575922
TAF1A	3.052986279	2.986059721	0.007116069	0.077573403
GOLGA6L6	3.051884419	2.662426199	0.010453099	0.098019301
CNGA4	3.050892453	2.116454635	0.022314819	0.155835874
SCYL2	3.049608007	4.166393003	0.002725941	0.04462882
TEX36	3.048956815	3.375494488	0.003998158	0.054449116
B3GNTL1	3.047066051	3.913436942	0.002973425	0.046974924
SULT2B1	3.046578738	4.350193454	0.002142286	0.038830418
GRM7 /// LOC100996542	3.046107997	2.112889348	0.022314819	0.155835874
NUS1	3.046107997	2.112889348	0.022314819	0.155835874
TMEM55A	3.045730223	1.682553031	0.034780972	0.205039552
AX748273	3.045354454	2.752235888	0.007826126	0.082555619
CHD8	3.044717637	4.616650084	0.001670039	0.033998787
POU1F1	3.042307108	4.167954359	0.002725941	0.04462882
NCK2	3.041561524	3.595050827	0.003355072	0.050071404
C5orf22	3.04076881	3.949790135	0.002601882	0.043412985
LBX2-AS1	3.039878425	4.445781998	0.002209609	0.039235172
CXCR2	3.039761907	3.804731601	0.002710732	0.044469637
LOC100505817	3.036847237	4.000345684	0.003213032	0.049354021
CFHR4	3.035917326	1.546384561	0.045799632	0.240968635
PLBD1	3.033558946	3.829366615	0.003916714	0.054412443
ARL14EP	3.031596105	4.418782352	0.00243218	0.041860671
RP11-753A21.2	3.031363995	2.94168361	0.008030972	0.084337772
FOXS1	3.029554916	4.760719253	0.00180109	0.035142198
C19orf60	3.028782524	3.980053896	0.002282946	0.040173011
LOC101927272	3.028707868	1.790020673	0.026706022	0.172890711
COL6A5	3.028262913	1.926591659	0.041756881	0.227810645
ZNF783	3.028254061	4.719764877	0.001603289	0.033420107
CCDC121	3.027725931	2.738050224	0.007826126	0.082555619
RP11-195M16.3	3.027725931	2.738050224	0.007826126	0.082555619
LINC00841	3.027433702	3.65121133	0.004264421	0.057442534
TRIM22	3.027365566	2.275218643	0.015150304	0.122275726

FHL3	3.027332426	4.213029933	0.002436766	0.041860671
DOCK10	3.027077936	2.767879384	0.006803492	0.075213009
RHOD	3.027012816	4.279595638	0.001958322	0.036598959
RAC2	3.024737941	3.014686543	0.006319303	0.072628703
GRHPR	3.023173948	4.731343626	0.002024502	0.037566834
FLII	3.021468348	2.915598342	0.0090842	0.0893286
FAM127B	3.0201337	4.136741242	0.003055493	0.047916984
LOC731157	3.019518849	4.315978981	0.002372798	0.041259629
B3GNT2	3.016514089	2.89335396	0.010299849	0.097684319
DNAL4	3.01452834	4.77085332	0.00180109	0.035142198
CCL24	3.013919749	2.632183614	0.010453099	0.098019301
IDO1	3.013598559	3.325273457	0.004405204	0.058628437
ALKBH4	3.013169492	3.365489302	0.003998158	0.054449116
NDUFB1	3.012762487	6.248945105	0.001134391	0.027416792
LINC00493	3.011762329	4.421146348	0.00243218	0.041860671
TIMP4	3.007840327	3.583655893	0.003355072	0.050071404
AVPR2	3.007417321	3.521720512	0.004331767	0.057983971
HS3ST5	3.00714445	3.0987065	0.005015008	0.062599664
RP11-4M23.7	3.006735705	5.01209789	0.001663959	0.033998787
VIPAS39	3.006129068	3.446995345	0.005165949	0.063963283
POM121L8P	3.005825384	4.159721916	0.002885289	0.0463016
RP5-894A10.6	3.004984952	3.07290483	0.005623714	0.067405742
LOC100507384	3.004559665	1.525611015	0.045799632	0.240968635
KIAA0141	3.004258069	3.672486551	0.004264421	0.057442534
GTF2H2B	3.003270344	3.944581265	0.002601882	0.043412985
LINC00476	3.002898295	2.996749705	0.007116069	0.077573403
EU250746	3.001952943	4.759788024	0.001872236	0.036319206
ZNF785	3.001716466	3.148800878	0.008141953	0.084939983
TLL1	3.001635531	2.336473579	0.012586629	0.109472446
RCC2	3.001109907	4.392108038	0.00268106	0.044075203
ZAP70	2.998331364	4.069238136	0.002669137	0.043909927
LOC100506999	2.99721051	1.9301512	0.041756881	0.227810645
PEBP4	2.996949303	3.84169341	0.003651944	0.051799007
LINC01300	2.99672734	1.586124245	0.045799632	0.240968635
LOC101927539	2.99590032	3.159212279	0.008141953	0.084939983
CT55	2.995361493	2.91230337	0.0090842	0.0893286
HMGN2	2.993964238	7.657426243	0.000958217	0.025264223
SUCNR1	2.992639724	3.764257389	0.003141633	0.048522849
LIMD2	2.991308591	3.778160064	0.002917009	0.0463016
LOC101928495	2.990716364	2.512878794	0.016551802	0.128668079
PITRM1-AS1	2.99056919	3.34650298	0.004405204	0.058628437
PPIL4	2.989215745	4.371764909	0.002036797	0.037756813
BC015159	2.987551003	3.197059013	0.006591493	0.074401841
HOXA11-AS	2.98741536	3.421751028	0.005651771	0.067533779

SUMF1	2.985813723	3.92953251	0.002780495	0.044893058
BBS7	2.985175405	2.930549417	0.0090842	0.0893286
DRAP1	2.984466169	3.787188455	0.004515652	0.058830288
NAGPA	2.984161887	3.757001347	0.003141633	0.048522849
NUDT4 /// NUDT4P1	2.981926713	4.896016281	0.001982784	0.036997277
CCL21	2.979700536	3.73799568	0.003386477	0.050129249
BPIFB2	2.979652499	2.899538833	0.0090842	0.0893286
WFDC13	2.977642316	1.754396789	0.026706022	0.172890711
GLIS2	2.977253995	2.317849612	0.012586629	0.109472446
RP11-220I1.5	2.976582072	3.41263568	0.006191067	0.07202281
TBC1D10C	2.975829504	4.074246706	0.002669137	0.043909927
C20orf202	2.975540826	4.190601559	0.002576672	0.043174883
AHR	2.975320848	2.429738836	0.019434951	0.141967001
ACYP1	2.974008983	3.141091324	0.008141953	0.084939983
ZNF606	2.973577199	5.433187996	0.001654643	0.033998787
NEFH	2.973510796	7.374817951	0.001067898	0.026816186
DGKG	2.973340553	3.381194353	0.006790588	0.075213009
NCF4	2.972901376	3.501456251	0.004331767	0.057983971
ZNF264	2.972508201	4.544527325	0.002170692	0.038849044
C1orf192	2.972421431	1.806580152	0.020717199	0.148245028
SHISA6	2.971346349	3.116019177	0.009072677	0.0893286
ZNF687	2.971159887	3.875982569	0.003407625	0.050410528
NDRG1	2.969797025	7.851801169	0.000998895	0.025675357
TYROBP	2.969785788	3.246996243	0.00594568	0.069745944
LYSMD1	2.969767246	2.660888901	0.010453099	0.098019301
PROX2	2.967914152	3.424189727	0.005651771	0.067533779
MB21D2	2.96751257	3.042089583	0.006319303	0.072628703
LOC730202	2.966022152	3.631807391	0.00499616	0.062599664
SAMD12	2.965679277	3.325665415	0.004860852	0.061355847
DAB1	2.965438777	3.431438401	0.005651771	0.067533779
ZNF439	2.963823114	2.267925374	0.015150304	0.122275726
STARD13	2.961844502	2.911593718	0.0090842	0.0893286
NTRK1	2.961694304	2.590921252	0.012140675	0.106947596
LOC101929143	2.960857225	1.624836634	0.034780972	0.205039552
C11orf21	2.960108782	2.139416654	0.022314819	0.155835874
LONP1	2.959749196	2.523041524	0.016551802	0.128668079
NUDT5	2.959146744	3.15133699	0.008141953	0.084939983
POMK	2.959005029	2.713358709	0.009030241	0.0893286
LINC01214	2.958294823	3.779537532	0.004515652	0.058830288
LINC01243	2.957330972	3.298599276	0.004860852	0.061355847
RP11-400N9.1	2.957200675	3.357980062	0.007458047	0.079735439
DKFZp547J222	2.9559999	3.747979101	0.003386477	0.050129249
RUNX3	2.954605868	3.605772676	0.005415811	0.065480303

PRPS2	2.954569419	3.746759285	0.003386477	0.050129249
CDT1	2.953951232	2.738922812	0.007826126	0.082555619
TOMM34	2.953443893	4.740219463	0.002024502	0.037566834
PARD6G	2.952946794	3.710084387	0.003653625	0.051799007
FLJ36848	2.95099191	4.472038238	0.00285306	0.045906903
SNRNP25	2.94974339	3.727625149	0.003653625	0.051799007
CALML6	2.949409499	2.848125172	0.011706775	0.104767834
UBE2Q1	2.948300755	2.396758834	0.022910824	0.156863073
ITPKC	2.947067984	3.769955714	0.004854398	0.061355847
MAGT1	2.9435965	2.343559352	0.012586629	0.109472446
FAM181A-AS1	2.943385385	3.511772319	0.004331767	0.057983971
LOC283731	2.942094359	3.202790795	0.006591493	0.074401841
RP11-774O3.3	2.941611838	2.575160821	0.014149938	0.117459486
LOC101927869	2.941527631	2.971809743	0.008030972	0.084337772
LINC00280	2.94070569	2.69881494	0.009030241	0.0893286
LOC497256	2.940439936	2.209627055	0.018334706	0.136997112
PRSS53	2.939338141	3.993467668	0.003422044	0.050560166
PRKAG3	2.937256643	2.400432215	0.022910824	0.156863073
PCDHB9	2.937041627	1.937241673	0.041756881	0.227810645
LOC101930566 /// LOC102800447	2.936733473	3.686065234	0.003945405	0.054449116
CLVS2	2.936199777	2.665107289	0.010453099	0.098019301
ERCC6 /// ERCC6-PGBD3 /// PGBD3	2.933974246	3.950775712	0.004149868	0.05615708
PRR3	2.933974246	3.950775712	0.004149868	0.05615708
SCGB1A1	2.931479774	3.236130662	0.00594568	0.069745944
RP1-6P5.2	2.931452222	2.202913391	0.018334706	0.136997112
LINC00355	2.930321557	3.082297087	0.01012814	0.096916605
ZCWPW2	2.929269843	2.241905649	0.018334706	0.136997112
SYTL1	2.928304056	3.59985522	0.005415811	0.065480303
POU5F2	2.927892461	1.662573631	0.034780972	0.205039552
NOS3	2.926930207	2.717421249	0.009030241	0.0893286
IRGC	2.925262941	3.443464512	0.005651771	0.067533779
CPQ	2.923230749	3.319897972	0.008202272	0.084939983
TAS2R7	2.923085384	3.029691885	0.006319303	0.072628703
CD38	2.922453818	2.931231752	0.0090842	0.0893286
ARHGAP11A	2.922047758	3.052365736	0.006319303	0.072628703
GABRE /// MIR224 /// MIR452	2.921803839	1.771353068	0.026706022	0.172890711
CLSTN3	2.919873432	4.301503994	0.00263245	0.043519711
BPIFA4P	2.919699456	3.789920864	0.004515652	0.058830288

LINC00461 /// MIR9-2	2.917020974	2.310744326	0.012586629	0.109472446
SPRR2B	2.916870157	3.714695132	0.003653625	0.051799007
B4GALNT1	2.916682056	3.427252317	0.005651771	0.067533779
RP11-307L14.1 /// RP11-307L14.2	2.916572425	3.284779383	0.005371775	0.065317664
LOC100287015	2.915835087	2.602761208	0.012140675	0.106947596
LY9	2.915689093	2.784457773	0.013339641	0.113500488
MURC	2.913681276	1.652983316	0.034780972	0.205039552
LOC340340	2.912105532	3.839233167	0.003916714	0.054412443
SLC10A1	2.911155151	4.077915887	0.003862936	0.054405334
TMEM110 /// TMEM110-MUSTN1	2.910269237	2.971114498	0.008030972	0.084337772
TPTE2P6	2.90953142	2.842975367	0.011706775	0.104767834
LOC100506374	2.908706621	2.304459921	0.015150304	0.122275726
AK096159	2.908586742	3.017883144	0.007116069	0.077573403
LINC00869	2.908258798	3.55840071	0.006383312	0.072829899
GREM2	2.906257503	2.710740528	0.009030241	0.0893286
CLYBL-AS2	2.906196617	3.463060641	0.005165949	0.063963283
CLDN10-AS1	2.906031333	2.94302797	0.0090842	0.0893286
ASF1B	2.905658318	4.174344074	0.002885289	0.0463016
NYX	2.905653263	3.867549893	0.003651944	0.051799007
FOLH1B	2.90554517	3.107263409	0.009072677	0.0893286
CTD-2012K14.6	2.905421267	3.371896329	0.006790588	0.075213009
UPK3A	2.905269629	3.791787098	0.004515652	0.058830288
PYY2	2.905069365	3.704691517	0.003945405	0.054449116
VSTM2A	2.905016748	2.620155634	0.012140675	0.106947596
ARHGEF10L	2.904057232	3.918730153	0.004431119	0.058645551
AC145343.2	2.901659536	3.368763921	0.006790588	0.075213009
TRMU	2.901221482	5.026144167	0.002078522	0.038417494
LOC101927650	2.90068916	4.033613128	0.004632215	0.059529532
PACSIN3	2.900588283	2.543152236	0.014149938	0.117459486
CDC14C	2.89995359	2.781138204	0.013339641	0.113500488
LIPE-AS1	2.898839394	3.009960166	0.007116069	0.077573403
LOC101929210	2.897899126	2.634942264	0.012140675	0.106947596
RAVER2	2.897652519	2.388138155	0.022910824	0.156863073
CIB4	2.897486159	3.228634928	0.006591493	0.074401841
GRAMD4	2.897423773	2.765275746	0.015240223	0.122275726
ZNF619	2.897394401	4.156331323	0.003055493	0.047916984
GRID2	2.897342736	3.524279987	0.00694101	0.0762667
DEFA6	2.896207864	2.43967104	0.019434951	0.141967001

LINC00970	2.895856471	3.121435844	0.009072677	0.0893286
CGB1 /// LHB	2.89556901	4.229412333	0.003438679	0.050710217
C11orf30	2.8929161	3.330075199	0.008202272	0.084939983
GNPNAT1	2.892829483	4.9695806	0.002533061	0.042474419
RASL11A	2.891238558	4.420164286	0.003446213	0.050710217
ANKFN1	2.889411313	3.575167423	0.005876651	0.069745944
METTL25	2.887965336	2.467424548	0.019434951	0.141967001
LINC00672	2.887853302	3.949372263	0.004149868	0.05615708
FBXO3	2.887522202	3.03584455	0.011327543	0.102585446
STK32B	2.887185193	3.689561568	0.004264421	0.057442534
SDC4	2.887157152	4.341909581	0.003272616	0.049792937
BARD1	2.885579074	3.54208922	0.006383312	0.072829899
RP5-1119A7.11	2.884915364	2.950606179	0.0090842	0.0893286
CYP4F30P	2.884372374	2.654347559	0.010453099	0.098019301
DOC2B	2.883927141	4.817186271	0.002648235	0.043703961
PRPF40B	2.882882318	4.09945359	0.003640076	0.051799007
AC002064.5	2.88271283	4.110536264	0.003640076	0.051799007
DUSP9	2.88256892	5.0691269	0.002258907	0.039899436
NT5DC1	2.881898429	4.008567364	0.003422044	0.050560166
COX16 /// SYNJ2BP-COX16	2.881172771	4.71468228	0.002939169	0.04659039
GAGE1 /// GAGE12B /// GAGE12C /// GAGE12D /// GAGE12E /// GAGE12F /// GAGE12G /// GAGE12H /// GAGE12I /// GAGE12J /// GAGE13 /// GAGE2D /// GAGE4 /// GAGE5 /// GAGE6 /// GAGE7	2.878838438	3.017199032	0.007116069	0.077573403
CIPC	2.878454586	4.265754702	0.003086039	0.048200573
S100A1	2.878442147	3.890426565	0.005062312	0.062989321
RABEPK	2.878307241	3.395242296	0.006790588	0.075213009
SLC35D3	2.878133097	3.915840775	0.004734598	0.060415632
LINC00973	2.87760652	2.678444685	0.010453099	0.098019301
ENO1-AS1	2.873224618	3.88598748	0.005062312	0.062989321

TRAM1L1	2.873169783	2.456058526	0.019434951	0.141967001
SYAP1	2.872594689	3.616405639	0.005415811	0.065480303
GBE1	2.871425484	5.774209209	0.001814289	0.035311737
RBM28	2.870677367	1.564893912	0.045799632	0.240968635
HIGD2A	2.870422034	4.541041135	0.003015305	0.047508741
C3orf35	2.869146211	4.121104904	0.003431927	0.050674305
ADAMTS4	2.868671418	3.077164726	0.01012814	0.096916605
KANK4	2.868056409	1.936316244	0.041756881	0.227810645
RP11-483I13.5	2.867355145	3.340280412	0.007458047	0.079735439
MND1	2.867326687	2.071326565	0.027322057	0.174558001
CENPQ	2.866400555	2.272651906	0.015150304	0.122275726
C17orf78	2.86430649	2.934001367	0.0090842	0.0893286
KRTAP2-1 /// KRTAP2-2	2.864195521	2.981905229	0.008030972	0.084337772
MTIF2	2.862572779	4.024632527	0.004632215	0.059529532
ZNF7	2.859250753	3.030119177	0.012693427	0.109710812
CLEC14A	2.859107941	2.929822158	0.0090842	0.0893286
RP11-177N22.3	2.859032876	2.880204176	0.010299849	0.097684319
RP4-633H17.2	2.858864527	2.775775925	0.015240223	0.122275726
C5orf42	2.85866654	2.663572442	0.010453099	0.098019301
WNT1	2.858062157	2.375750213	0.027120138	0.174025207
LOC102723954	2.857788407	2.266211634	0.015150304	0.122275726
MGST3	2.856267669	4.431836569	0.003285554	0.049957427
FOLH1	2.855673688	1.671129368	0.034780972	0.205039552
RPUSD1	2.854981623	3.908813175	0.004734598	0.060415632
CXXC1P1	2.85460777	4.323406856	0.003443231	0.050710217
TEX22	2.854018795	2.949906178	0.0090842	0.0893286
NEURL1B	2.850031362	3.566110545	0.006383312	0.072829899
CAV2	2.849724127	3.222983991	0.006591493	0.074401841
LOC101927553	2.849191624	4.571525742	0.002761613	0.044680308
DUOXA1	2.848925077	3.432728039	0.005651771	0.067533779
GTF2H2 /// GTF2H2B /// GTF2H2C /// GTF2H2C_2	2.848034906	5.182665265	0.002331613	0.040876313
BRIP1	2.847253946	2.078187388	0.027322057	0.174558001
GMIP	2.847224605	3.763548729	0.005222803	0.064305083
UTS2B	2.847136145	2.766490129	0.015240223	0.122275726
DUOX1	2.847112509	3.600177349	0.005415811	0.065480303
PRKCH	2.846778592	3.207219854	0.00731957	0.079094454
FOXR2	2.846407354	3.531056046	0.00694101	0.0762667
T-18	2.845339748	1.920236455	0.041756881	0.227810645
EIF2S3	2.845128204	5.141033788	0.002622814	0.043412985
CTD-2130O13.	2.844871721	3.035475777	0.011327543	0.102585446

1				
SMIM5	2.843789864	3.30207667	0.009033387	0.0893286
ZNF354B	2.843535246	4.195068421	0.003838672	0.054161004
HIST1H2BO	2.843370867	3.794507689	0.004515652	0.058830288
MIR4313	2.843302145	3.263509926	0.009963019	0.096257688
FAM86C1	2.843240749	3.54450725	0.006383312	0.072829899
RAI2	2.842593339	4.120242252	0.004811303	0.061161862
LOC101060510	2.842383293	1.716970789	0.034780972	0.205039552
LOC101926963	2.8423797	2.841524111	0.011706775	0.104767834
FAM87A /// FAM87B	2.841953606	3.162506087	0.008141953	0.084939983
COBL	2.841824791	2.802201915	0.013339641	0.113500488
SRSF4	2.84102222	3.380467557	0.006790588	0.075213009
CLCN4	2.840717442	2.803947048	0.013339641	0.113500488
CLTCL1	2.840190969	3.945834715	0.004149868	0.05615708
GRM8	2.8394746	2.648556422	0.010453099	0.098019301
LINC00967	2.839082806	2.174889398	0.022314819	0.155835874
GKN1	2.837484441	4.327590765	0.003443231	0.050710217
LOC100289361	2.83729333	4.42723089	0.003446213	0.050710217
TAAR8	2.836077332	3.69051771	0.006537408	0.074299367
GRTP1	2.835834519	2.4442002	0.019434951	0.141967001
RP11-749H17.2	2.834774535	2.729532905	0.017459041	0.133224833
S100A12	2.834085831	2.357616618	0.027120138	0.174025207
PTGR1	2.831605721	5.018918488	0.002649176	0.043703961
AX746699	2.831572832	3.757252374	0.005222803	0.064305083
KB-1000E4.2	2.831015322	2.552309339	0.014149938	0.117459486
LOC100506538 /// NDUFAF6	2.830126422	2.655698042	0.010453099	0.098019301
LOC728084	2.829126152	3.345444562	0.007458047	0.079735439
NEK10	2.82763908	4.607893387	0.003305301	0.049999632
CCR8	2.825469734	4.220279881	0.003632326	0.051799007
UBL4A	2.8254616	3.73826981	0.00562384	0.067405742
PI4K2B /// SEPSECS-AS1	2.823941437	2.278211656	0.03224147	0.195096667
MIA2	2.822693663	3.843154255	0.005799521	0.068983464
PLEKHM3	2.8224887	3.566654364	0.006383312	0.072829899
ND4	2.819505523	8.734260383	0.001622023	0.03378064
ZNF484	2.818171162	4.375290136	0.003985437	0.054449116
ZNF211	2.817077417	2.541561664	0.016551802	0.128668079
ZZZ3	2.813803817	3.686189085	0.006537408	0.074299367
MED24 /// MIR6884	2.813345472	3.627491016	0.00499616	0.062599664
TFAP2A	2.812111084	3.529180003	0.00694101	0.0762667
XAGE2	2.811262468	4.712137116	0.003058065	0.047925327

FAM188A	2.810445125	3.126498371	0.009072677	0.0893286
CLEC3A	2.810048256	3.532745162	0.00694101	0.0762667
TAOK3	2.809785974	3.697046451	0.006537408	0.074299367
NAV2-AS4	2.809497143	3.216373045	0.00731957	0.079094454
SLC35A1	2.808397399	3.737755252	0.00562384	0.067405742
DTX1	2.807944719	4.48715187	0.003776467	0.05337952
PRR34	2.807418	2.070428628	0.027322057	0.174558001
NRF1	2.806310117	2.371306851	0.027120138	0.174025207
LOC102724112 /// WIBG	2.805469763	4.78327807	0.003075942	0.048173355
SPECC1	2.805047537	3.307384463	0.009033387	0.0893286
USE1	2.804813666	2.318010402	0.03224147	0.195096667
FLJ33360	2.804150271	3.378266484	0.006790588	0.075213009
CKAP2 /// IGLJ3 /// IGLV3-19 /// IGLV3-19	2.8041454	2.705496371	0.017459041	0.133224833
LOC101928191	2.803856391	4.076347713	0.004101696	0.055633396
BTN3A3	2.80292106	2.676916667	0.020057392	0.144802785
LOC100506114	2.802752806	3.04579013	0.011327543	0.102585446
TC2N	2.802395663	3.30520754	0.009033387	0.0893286
LOC100128644	2.802048672	4.334277036	0.004624411	0.059494341
PBDC1	2.800070965	2.465799794	0.019434951	0.141967001
ARMCX6	2.798575335	5.837846287	0.002242023	0.03966084
TRPM8	2.796994944	2.61551522	0.012140675	0.106947596
MASTL	2.796826614	2.3979494	0.022910824	0.156863073
PADI3	2.796826614	2.3979494	0.022910824	0.156863073
PHF5A	2.795579939	5.282084743	0.002921381	0.046339694
POLR2J /// POLR2J2 /// POLR2J3 /// POLR2J4 /// UPK3BL	2.794306045	2.49306296	0.019434951	0.141967001
PRAMEF12	2.794272088	4.242907488	0.004711457	0.060415632
BC043227	2.794199843	3.06054553	0.011327543	0.102585446
ANKUB1	2.792980978	2.971049936	0.014252316	0.117644205
C1orf127	2.792696564	4.251220749	0.004466789	0.058678986
ENO3	2.790924438	2.946438847	0.0090842	0.0893286
COX10-AS1	2.790189063	3.239178042	0.011004551	0.10122119
LINC00478	2.790026433	2.500196862	0.016551802	0.128668079
RP4-539M6.14	2.787011735	2.607789554	0.012140675	0.106947596
AZU1	2.785473432	3.619009719	0.008248451	0.084939983
TEAD4	2.784994644	2.882732407	0.011706775	0.104767834
OR10H3	2.783813848	2.662037731	0.020057392	0.144802785

AP006216.12 /// APOA1-AS	2.783256362	3.933708635	0.00634363	0.072694901
RRP12	2.782656854	3.904427402	0.005062312	0.062989321
MMP9	2.781521204	4.26081279	0.004466789	0.058678986
ADIRF	2.780691603	4.504531982	0.003607954	0.051799007
LOC441052	2.779020545	3.857125501	0.005799521	0.068983464
LOC100506100	2.778857377	4.69129937	0.003312922	0.050049628
SLCO5A1	2.778038984	2.51161696	0.016551802	0.128668079
CCNH	2.777713784	4.295076959	0.004020112	0.054589864
LINC00837	2.775554521	4.500128969	0.003607954	0.051799007
IGHA1 /// IGHV4-31	2.775098141	2.669112189	0.020057392	0.144802785
DNAJB7	2.773277869	2.861373057	0.011706775	0.104767834
RP11-340A13.1	2.772257928	3.912513164	0.004734598	0.060415632
FAM13B	2.77215408	4.326906863	0.004863127	0.061355847
FAM182A /// FAM182B	2.772140242	2.999245063	0.014252316	0.117644205
MAPK12	2.771636318	3.791543718	0.007150342	0.077730604
FASTKD2	2.771343672	2.595695044	0.014149938	0.117459486
TAS2R43 /// TAS2R45	2.770933655	2.652038706	0.02310992	0.156863073
LOC101928730	2.770923366	3.850316384	0.005799521	0.068983464
ZNF25	2.770511745	2.551120113	0.016551802	0.128668079
IL4I1	2.770195906	3.041230908	0.012693427	0.109710812
RASA2	2.769291971	3.570848573	0.006383312	0.072829899
C4orf17	2.768861739	3.897638906	0.005062312	0.062989321
TRIB3	2.764380431	4.751235338	0.003449469	0.050710217
OSCAR	2.763570049	3.139498389	0.009072677	0.0893286
HMGN3	2.763431801	5.863051776	0.002641188	0.043633458
CPVL	2.762470413	2.338428983	0.027120138	0.174025207
FAM83G	2.762015464	3.290363379	0.009033387	0.0893286
CMTR2	2.759258269	3.251514073	0.011004551	0.10122119
SERTAD4-AS1	2.757937432	2.641974947	0.02310992	0.156863073
TVP23C	2.756504778	3.862931463	0.005799521	0.068983464
HP09025	2.755957619	4.747355252	0.003449469	0.050710217
MIR21 /// VMP1	2.75539437	3.812138755	0.006663324	0.074789678
TRMT61B	2.754609438	2.822228275	0.013339641	0.113500488
TYSND1	2.753907316	4.833461827	0.003325012	0.050071404
ITK	2.75374465	2.666247035	0.020057392	0.144802785
LOC101927458 /// LPHN2	2.753645399	3.657205921	0.007626368	0.081251087
TRIP6	2.753608184	3.823265325	0.006663324	0.074789678
LOC286161	2.753594076	5.197351976	0.003252263	0.049579599

OR52A1	2.753110925	3.370827611	0.007458047	0.079735439
RGS9	2.752479453	3.048309718	0.011327543	0.102585446
DMKN	2.749965396	4.298948462	0.005384523	0.065438806
LOC653486 /// SCGB1C1	2.749841404	3.280434805	0.009963019	0.096257688
MYO18B	2.748940477	2.6070097	0.026707832	0.172890711
TMEM196	2.748896219	2.817734855	0.013339641	0.113500488
SLC26A1	2.748656304	2.920476887	0.018080005	0.136264188
AMPD3	2.74839715	5.042746203	0.003783879	0.053452133
INCENP	2.746871119	3.96121224	0.005949722	0.069745944
CALML5	2.745328794	3.971272354	0.005949722	0.069745944
ZNF33A	2.744034267	2.924443072	0.018080005	0.136264188
RPTOR	2.742675816	3.682812422	0.00705773	0.07736827
GUSBP4 /// LOC100653061	2.742383353	2.453614085	0.019434951	0.141967001
RECQL4	2.741696574	3.227955353	0.011004551	0.10122119
TMEM30A	2.741438632	3.71347521	0.006060814	0.070871401
LINC00458	2.741338462	2.543563712	0.016551802	0.128668079
ARHGAP35	2.740996962	4.256409917	0.004466789	0.058678986
RP11-218C14.8	2.740017151	3.254267862	0.011004551	0.10122119
HCRT	2.739723168	3.811641044	0.006663324	0.074789678
B4GALT4	2.739485626	3.854772551	0.005799521	0.068983464
CDCP1	2.739276388	2.768665907	0.015240223	0.122275726
CTD-2002J20.1	2.739117756	3.197668536	0.012173415	0.106947596
MRPL20	2.738150552	5.201944894	0.003252263	0.049579599
RP11-313C4.1	2.737860181	1.593175751	0.045799632	0.240968635
KCNN1	2.737108086	4.648392342	0.003899931	0.054412443
MYO16	2.736845569	3.305540338	0.009033387	0.0893286
MSANTD3	2.734519649	3.321169863	0.009033387	0.0893286
SYNGR3	2.734141662	4.028211527	0.006855999	0.075615556
LOC100506446	2.733610672	3.135195062	0.009072677	0.0893286
H1FOO	2.733312328	3.388290747	0.010730295	0.099399091
CLASP1	2.732655602	4.214527713	0.005248701	0.064421366
HMGN2P46	2.732425626	7.009884051	0.002414156	0.041860671
ATP1A2	2.732121494	3.842494968	0.006214146	0.072161502
LOC102725454	2.732090061	4.004697708	0.005243495	0.064421366
CDKAL1	2.732037094	2.77974564	0.015240223	0.122275726
BTBD9	2.731251059	4.269368409	0.005971452	0.069965756
TSPYL2	2.730943621	4.353668107	0.004399112	0.058628437
OR6A2	2.729842427	1.973051952	0.041756881	0.227810645
GRAMD2	2.729056392	3.41772186	0.010730295	0.099399091
TRIM23	2.728432881	2.357691026	0.027120138	0.174025207
OLFML1	2.727703014	2.873004726	0.011706775	0.104767834
PLD2	2.725795423	4.799823133	0.003713701	0.052555574

PPP2R5A	2.724732656	3.108211163	0.01012814	0.096916605
LOC101928631 /// ZNF77	2.724071248	4.06235156	0.006079527	0.071019522
PSMD7	2.723629324	4.937267748	0.003688765	0.052265667
IL4	2.723341516	2.257599732	0.03850259	0.217956466
LOC101927723	2.722844652	2.090471474	0.027322057	0.174558001
FGF13	2.722652344	5.324498304	0.003623799	0.051799007
LOC728061	2.722419377	2.529144113	0.016551802	0.128668079
TMPRSS13	2.722374222	4.490412823	0.004899885	0.061786419
STK10	2.722234556	3.202862032	0.012173415	0.106947596
EYA1	2.721849246	2.586213692	0.026707832	0.172890711
OR2W1	2.721325955	2.641203148	0.02310992	0.156863073
MYD88	2.721026813	4.141487736	0.004811303	0.061161862
OTC	2.720726763	3.292423184	0.009963019	0.096257688
CGN	2.720131607	3.481883288	0.008233828	0.084939983
IL5	2.719904844	3.393867528	0.010730295	0.099399091
IGSF1	2.719903175	4.282472747	0.005669237	0.067673641
ATP6V1E1	2.71942873	5.663029529	0.003223503	0.049354021
TENM4	2.719187614	4.625956742	0.004237983	0.057316464
TRIM56	2.717524611	2.717700825	0.017459041	0.133224833
ATG16L1	2.717524348	2.944687591	0.016035504	0.126496324
RBP3	2.715534067	3.080736211	0.011327543	0.102585446
MARS /// MIR6758	2.7154662	2.916962576	0.018080005	0.136264188
C14orf183	2.714388328	3.790490457	0.007150342	0.077730604
DVL1	2.714363279	4.135863918	0.004811303	0.061161862
DMGDH	2.711667341	2.520974017	0.016551802	0.128668079
AK055458	2.710902601	2.993866432	0.014252316	0.117644205
LOC100506107	2.7102544	3.614184133	0.008929719	0.0893286
ZNF318	2.710243022	3.628211297	0.008248451	0.084939983
RP11-219B4.3	2.71007299	2.212407121	0.03850259	0.217956466
KCNG4	2.710017493	3.941615811	0.00634363	0.072694901
MRPL19	2.708049538	2.498588036	0.019434951	0.141967001
KIF21A	2.707506543	3.290466548	0.009963019	0.096257688
LOC101927362	2.706727585	4.751378254	0.004484291	0.058687998
ANKRD19P	2.705955859	3.832830096	0.006663324	0.074789678
PRO1483	2.703740445	3.091386925	0.011327543	0.102585446
C5orf15	2.70319482	3.830527908	0.006663324	0.074789678
SYT5	2.702460141	3.295095923	0.009963019	0.096257688
MUC3	2.702418284	3.06010846	0.011327543	0.102585446
POLQ	2.702316615	3.039536269	0.012693427	0.109710812
MYOC	2.701059554	4.491735881	0.005126703	0.063723024
COX6CP1 /// COX6CP1	2.700988039	3.922712596	0.006767907	0.075213009

PKP1	2.700645323	2.964205604	0.016035504	0.126496324
ARHGAP33	2.700123672	4.433113921	0.004768213	0.060811535
RP3-334F4.1	2.698830059	2.703224494	0.020057392	0.144802785
SLC25A38	2.696069174	4.6893718	0.004365373	0.058400542
LOC101929180	2.69586675	3.236654648	0.011004551	0.10122119
ASPSCR1	2.695132305	3.162201806	0.014967264	0.121917808
CCDC14	2.694977307	3.786828752	0.007678842	0.081662004
GAD1	2.694093381	1.933849395	0.041756881	0.227810645
ABCC10	2.693749927	3.23494567	0.011004551	0.10122119
FIGLA	2.692022263	2.384402939	0.027120138	0.174025207
BLZF1	2.689619895	2.421696758	0.022910824	0.156863073
RAB33B	2.689369828	3.610977681	0.008929719	0.0893286
ACIN1	2.689190754	3.479569587	0.008983126	0.0893286
MECR	2.687436198	3.690852058	0.00705773	0.07736827
WDR5B	2.687204166	3.944951888	0.00634363	0.072694901
ADAM15	2.687069091	4.664769445	0.004730986	0.060415632
C17orf96	2.687018107	3.66384475	0.007626368	0.081251087
KIR2DL1	2.686872121	3.057851948	0.011327543	0.102585446
HSD17B7P2	2.686808706	3.999220706	0.007749009	0.082222092
GLT8D1	2.685582012	3.30726706	0.009033387	0.0893286
FIZ1	2.68447839	3.778106653	0.007678842	0.081662004
CEP72	2.684348537	1.612048075	0.045799632	0.240968635
SPOP	2.683411297	3.165353554	0.014967264	0.121917808
LINC00933	2.683313978	4.006975412	0.007286762	0.079094454
MAST3	2.683163675	3.93041046	0.006767907	0.075213009
HP08942	2.682667924	2.312774333	0.03224147	0.195096667
OR2AD1P /// OR2AD1P	2.682376947	3.395757948	0.010730295	0.099399091
SLC2A13	2.680420835	2.769138751	0.015240223	0.122275726
PHRF1	2.678706371	3.010429258	0.014252316	0.117644205
IFNE	2.678595496	3.392679826	0.011748854	0.104864769
DAAM2	2.678104765	3.809649654	0.007150342	0.077730604
AC009133.15	2.676425533	3.855976115	0.008826286	0.0893286
BTBD19	2.675555874	2.820404088	0.013339641	0.113500488
VAPB	2.674911257	2.43365307	0.022910824	0.156863073
CFH	2.674679977	2.605450122	0.026707832	0.172890711
ZNF815P /// ZNF890P	2.673267375	1.933541138	0.041756881	0.227810645
GPR45	2.671802105	3.932092891	0.006767907	0.075213009
HIST1H2AC	2.67161537	4.57830592	0.005020865	0.062639492
CNR2	2.671543104	2.490644902	0.019434951	0.141967001
LOC101927206	2.669987353	4.352986483	0.006072763	0.070975797
C12orf76	2.669157354	3.708396414	0.009555837	0.093380086
PKD2L1	2.668886003	3.962645216	0.00634363	0.072694901

LINC00998	2.66794228	3.972690045	0.008245364	0.084939983
ZNF74	2.66781174	3.16839025	0.014967264	0.121917808
LINC00328	2.667551603	3.19574718	0.01348744	0.113769095
PPP3CB-AS1	2.666126161	2.789392203	0.015240223	0.122275726
RP11-513N24.1	2.664016804	3.281271102	0.009963019	0.096257688
H2AFY2	2.662025936	4.395483482	0.005246892	0.064421366
TMEM132A	2.661869118	3.087565668	0.011327543	0.102585446
LCE2B	2.66081683	3.057178507	0.012693427	0.109710812
GPR183	2.660296448	3.500963299	0.008233828	0.084939983
RP11-701I24.3	2.660069344	2.820039258	0.013339641	0.113500488
OR5J2	2.660041598	2.56699788	0.030962986	0.190563917
TRIM35	2.658343651	3.343782522	0.012880261	0.110998929
SLC4A2	2.656671291	2.47946809	0.019434951	0.141967001
AXIN2	2.656043298	2.976884718	0.016035504	0.126496324
UPK2	2.653482972	4.60526124	0.00581046	0.069078607
OR4F16 /// OR4F21 /// OR4F29 /// OR4F3	2.653054893	3.306345855	0.009033387	0.0893286
LOC101927768 /// LOC101927794	2.652330134	3.635262086	0.008248451	0.084939983
GALK1	2.651981481	2.614971944	0.026707832	0.172890711
ABCA13	2.651255273	2.623246287	0.026707832	0.172890711
MYLK	2.65099747	4.040388847	0.006855999	0.075615556
LOC101929505	2.650918631	3.880983915	0.008251023	0.084939983
PSMD5	2.650813445	2.151568879	0.046195495	0.241429268
SCN1B	2.650722113	4.111858977	0.007338043	0.07921004
RP4-575N6.5	2.649727699	3.492336158	0.008233828	0.084939983
TDRD12	2.649551606	3.067994859	0.011327543	0.102585446
RP11-157P1.4	2.649008277	3.303080969	0.009963019	0.096257688
SYP	2.648206181	3.415368568	0.010730295	0.099399091
ACVR1	2.647023243	4.128073772	0.006931109	0.0762667
MILR1	2.646847091	2.500938643	0.019434951	0.141967001
SMR3B	2.645792336	4.048236615	0.006855999	0.075615556
TUBGCP2	2.644539616	3.478231177	0.008983126	0.0893286
SLC17A2	2.64325168	2.875188275	0.020429716	0.146813978
PAN3	2.643044614	5.496014011	0.004407365	0.058628437
SUOX	2.643031784	3.999680681	0.007749009	0.082222092
OTUD7A	2.642975221	3.959056068	0.008778694	0.0893286
MAMDC4	2.640994762	3.557253015	0.010496335	0.098019301
NLE1	2.639248845	3.696587343	0.010295292	0.097684319
SLC38A3	2.637685211	3.649851253	0.008248451	0.084939983
CXCR6	2.637196573	3.989591414	0.008245364	0.084939983

LINC00824	2.635147801	3.79810131	0.007150342	0.077730604
NKX3-1	2.634893927	8.034064806	0.003106569	0.048204405
GLTSCR1L	2.633940965	5.287638149	0.004246195	0.057347162
GPR62	2.633073798	3.93283192	0.006767907	0.075213009
RP4-539M6.21	2.631281941	4.076048961	0.008237392	0.084939983
WNT9A	2.630115249	2.516641436	0.036013059	0.209203458
HOXC-AS3	2.629822517	3.201470659	0.01348744	0.113769095
KIAA1755	2.629044049	4.335080044	0.006706733	0.075213009
RHBG	2.628047782	4.778659059	0.005135176	0.06376086
ECD	2.627894659	4.51966935	0.005975342	0.069976437
UBE3B	2.627021517	3.913258746	0.007225225	0.078472026
TNNI3	2.626593441	4.148851522	0.006549962	0.074370084
FBXW8	2.626488484	2.673097183	0.02310992	0.156863073
CASC10	2.626170841	3.186682055	0.01348744	0.113769095
CD72	2.625025107	2.792862865	0.015240223	0.122275726
RP11-111M22.4	2.624596469	2.484259414	0.019434951	0.141967001
ERICH4	2.623860276	2.095900241	0.027322057	0.174558001
AP000696.2	2.620792757	1.979004356	0.041756881	0.227810645
CDK5RAP1	2.620278102	3.821125091	0.010121155	0.096916605
SIRPB2	2.617993078	3.390897949	0.011748854	0.104864769
IL19	2.617858314	3.646717217	0.011980945	0.10657189
DENND1B	2.617739597	2.610897202	0.026707832	0.172890711
RP11-791M20.1	2.617116803	2.71543898	0.020057392	0.144802785
OR1A1	2.616458925	3.67146327	0.011101378	0.101436631
CCR4	2.61631539	3.420251607	0.010730295	0.099399091
A2ML1	2.615805294	3.079957064	0.018523963	0.137623322
HPYR1	2.614798484	3.611200142	0.008929719	0.0893286
KLC4	2.614730127	3.944510981	0.00935214	0.091732971
LINC00605	2.614033889	2.874471728	0.020429716	0.146813978
DDN	2.613999038	3.656558874	0.011101378	0.101436631
SPNS2	2.613904152	2.532178459	0.030962986	0.190563917
TRAV12-1 /// TRAV12-1 /// TRAV12-1 /// YME1L1	2.613699017	2.97972037	0.016035504	0.126496324
F13B	2.613332953	3.668901306	0.011101378	0.101436631
BC045779	2.613134993	2.261929272	0.03850259	0.217956466
PRDM16	2.612918071	2.341633648	0.03224147	0.195096667
BCL7B	2.61282558	3.616248911	0.008929719	0.0893286
DBH-AS1	2.612622433	2.999114085	0.014252316	0.117644205
SUPT4H1	2.612612261	4.466303724	0.007145155	0.077730604
RAB25	2.611895351	1.932616499	0.041756881	0.227810645
TMEM123	2.61110749	5.170313402	0.005359727	0.065317664

LOC102723779	2.609573518	4.207521834	0.007381306	0.079505562
AIP	2.605488262	3.662477513	0.011101378	0.101436631
CASQ1	2.60494361	2.823065516	0.023136836	0.156863073
NEUROD4	2.604597501	3.842649339	0.009448224	0.092482364
LRP3	2.60398929	3.117548211	0.016636829	0.128859999
RP11-108P20.1	2.603793541	3.070465994	0.018523963	0.137623322
MIR6741 /// PYCR2	2.602565276	2.809935657	0.0262636	0.17205368
SOX10	2.602488048	2.679729715	0.02310992	0.156863073
PDIA4	2.602300864	3.385968423	0.011748854	0.104864769
MGARP	2.6003779	2.653204497	0.02310992	0.156863073
TEN1 /// TEN1-CDK3	2.599597263	5.300752539	0.004804346	0.061161862
HRH4	2.599327169	2.601304075	0.026707832	0.172890711
CIRBP	2.597662007	7.53115267	0.003595066	0.051799007
YTHDF1	2.597029264	6.811735146	0.003740667	0.052905323
R3HDM2	2.596406291	5.665124726	0.004818955	0.061226004
LOC100131532	2.595581373	3.704801606	0.010295292	0.097684319
IFNAR1	2.595382065	3.0921503	0.018523963	0.137623322
ND3 /// SH3KBP1	2.594988536	9.511239429	0.00335904	0.050071404
KLHL8	2.594863514	3.529004959	0.011397017	0.102936841
RPL34-AS1	2.594577627	2.923478788	0.018080005	0.136264188
CBY3	2.594389643	4.409370074	0.006771721	0.075213009
CHRDL2	2.593690691	3.90022466	0.007718521	0.082009724
CEL	2.591807834	2.696094029	0.020057392	0.144802785
LOC101929988	2.591471566	3.45970335	0.01468201	0.120219512
ANKS4B	2.591252769	3.134917068	0.016636829	0.128859999
NKD2	2.587887177	3.82888515	0.009448224	0.092482364
LOC728743	2.587582551	3.104571802	0.018523963	0.137623322
MTPN	2.585360392	1.993549342	0.041756881	0.227810645
TAB1	2.584999768	2.273943251	0.03850259	0.217956466
CDRT15L2	2.584760617	2.510357834	0.036013059	0.209203458
LINC00896	2.584723147	3.837583745	0.009448224	0.092482364
SHPK /// TRPV1	2.583195034	3.233499653	0.012173415	0.106947596
RP4-710M16.1	2.58248496	4.074288495	0.008734314	0.089328038
CGB7 /// NTF4	2.582450238	2.955274762	0.016035504	0.126496324
LOC399884	2.582385277	3.509561339	0.012387782	0.108465809
STH	2.581811691	2.364726427	0.027120138	0.174025207
AGR2	2.581744729	4.850652949	0.006329959	0.072694901
CNBP	2.581575967	4.253560078	0.008654935	0.088631966
ADAMTS18	2.578094178	3.919537346	0.009969137	0.096257688
NCKAP1	2.577902659	3.540588437	0.011397017	0.102936841

UQCC2	2.57735918	3.12392904	0.016636829	0.128859999
KLRG2	2.577175664	2.951162738	0.018080005	0.136264188
PTBP2	2.576329039	3.395570605	0.011748854	0.104864769
PRR32	2.57616049	3.725616932	0.009555837	0.093380086
SCN2B	2.575521703	3.073377275	0.018523963	0.137623322
OOEP	2.574582394	3.226644417	0.012173415	0.106947596
RNU2-22P	2.574221477	1.985838371	0.041756881	0.227810645
ELAVL3	2.572993246	3.108358747	0.018523963	0.137623322
STARD7	2.572547182	8.180630041	0.003712116	0.052555574
SOD3	2.572421526	3.987298701	0.008245364	0.084939983
PPP1R27	2.572044735	4.875868799	0.005899638	0.069745944
RP11-285J16.1	2.571402582	2.966851367	0.016035504	0.126496324
BIRC2	2.570879532	5.199170692	0.005737513	0.06845386
ND6	2.570800228	10.01601638	0.00360475	0.051799007
XRCC1	2.569852053	4.783186267	0.00650632	0.074089415
SUCLG2	2.569388135	4.085601548	0.008237392	0.084939983
TMEM182	2.568902559	3.234735685	0.012173415	0.106947596
NFU1	2.568817604	3.288097687	0.015540812	0.123925577
IL1RL2	2.568669779	2.196821114	0.046195495	0.241429268
TMEM87A	2.56755222	3.396042327	0.011748854	0.104864769
LOC284865	2.566864512	3.565497371	0.010496335	0.098019301
ERICH1-AS1	2.566068357	4.05108337	0.009266069	0.09096469
FAM169B	2.565283345	3.252631488	0.018852638	0.139228598
RSU1P2	2.565070982	3.474383057	0.013478912	0.113769095
HRSP12	2.562268974	2.877365709	0.020429716	0.146813978
SERP1	2.561675555	6.94116204	0.004242516	0.057344754
TSTD1	2.56053237	6.635493958	0.004247576	0.057347162
PMEPA1	2.560236338	5.344584877	0.005606199	0.067405742
WFDC5	2.55751641	4.719116095	0.006449528	0.073532932
MMAB	2.557469066	3.300415969	0.015540812	0.123925577
CNIH1	2.557255933	2.595336506	0.026707832	0.172890711
ND2	2.556081359	7.618981834	0.004069221	0.055224805
SPC24	2.554403118	3.059908031	0.020661082	0.14811417
TMEM14A	2.55273532	4.574914431	0.006593931	0.074401841
PHF12	2.551937881	4.000371435	0.011099404	0.101436631
BC037214 /// RP11-58G13.1	2.550117396	4.218891805	0.007381306	0.079505562
SLC10A3	2.548578162	3.727051488	0.009555837	0.093380086
SLC29A4	2.548366414	2.845337956	0.023136836	0.156863073
FAM50B	2.547978545	4.2171166	0.007381306	0.079505562
PLAC1	2.547652212	3.873141395	0.01212097	0.106947596
MUC6	2.547269294	3.689922538	0.011101378	0.101436631
LNX1	2.546995782	3.063400644	0.020661082	0.14811417
INSL6	2.544861139	2.985714637	0.016035504	0.126496324

RAB26	2.544781042	4.863860769	0.006110434	0.071345088
TOP3B	2.543594545	3.331168828	0.014138806	0.117459486
ZNF408	2.541862272	4.031092553	0.009835439	0.095555885
LOC100240734	2.540279229	1.962474213	0.041756881	0.227810645
TLCD2	2.539692188	2.952173561	0.018080005	0.136264188
ATP6V0A2	2.538002687	4.088266377	0.008237392	0.084939983
NUTM2F /// NUTM2G	2.536739551	3.885541298	0.011349218	0.102663005
GLOD5	2.536577114	2.690080977	0.02310992	0.156863073
TDGF1 /// TDGF1P3	2.536519661	3.763967792	0.012496116	0.10929217
GPRC5D	2.536452657	3.728999546	0.013425963	0.113769095
RP11-134L10.1	2.535535205	3.324744477	0.014138806	0.117459486
ZNF625	2.535176294	3.195413135	0.01348744	0.113769095
GPR132	2.535126406	3.178429316	0.014967264	0.121917808
RP1-181J22.1	2.532834715	2.577043516	0.030962986	0.190563917
PSG6	2.532577423	2.525157347	0.036013059	0.209203458
NDUFC1	2.532276816	5.082718309	0.006143539	0.071695988
DUSP19	2.531042495	3.986460702	0.011800903	0.1051695
AOC2	2.530953015	4.56336505	0.006881669	0.075863051
LYPD5	2.53045529	2.169634599	0.046195495	0.241429268
TYK2	2.529585176	3.600735274	0.013991958	0.117016402
HAUS7 /// TREX2	2.529408719	2.029733569	0.033664757	0.201030113
LINC01049	2.529405599	3.991674176	0.011099404	0.101436631
ZNF668	2.528970295	2.933902731	0.018080005	0.136264188
PALM3	2.528785339	1.954607788	0.041756881	0.227810645
LINC00560	2.52828669	3.423358519	0.01601018	0.126496324
FLJ23519 /// RNH1	2.527742268	4.580511852	0.008176817	0.084939983
ZDHHC5	2.527720285	4.353715294	0.008207657	0.084939983
IER3IP1	2.527384977	3.533595011	0.012387782	0.108465809
SMPDL3B	2.526522018	2.646652654	0.02310992	0.156863073
PCDH15	2.525961016	2.199317264	0.046195495	0.241429268
LTB4R2	2.525780616	2.571777265	0.030962986	0.190563917
RP11-819C21.1	2.523877213	3.361092725	0.012880261	0.110998929
FUK	2.523836248	3.93718835	0.009969137	0.096257688
RP11-619L19.1	2.523536991	3.330561071	0.014138806	0.117459486
SLCO4C1	2.523482187	2.366494546	0.027120138	0.174025207
LOC286437	2.52330061	2.351762457	0.03224147	0.195096667
AX746492 /// TNPO1	2.523098027	2.888849792	0.020429716	0.146813978
TIGD6	2.522847114	2.867981961	0.023136836	0.156863073
STAC3	2.521871882	4.766114035	0.007009753	0.076950058

ZNF776	2.521129221	3.132953267	0.016636829	0.128859999
SLC5A10	2.519255424	4.299966563	0.010005701	0.096452169
ZNF821	2.519056977	3.199441262	0.01348744	0.113769095
TBR1	2.519045893	3.553365028	0.011397017	0.102936841
OPCML	2.516588237	3.39225405	0.017478245	0.133224833
HTR3A	2.516220882	2.418205961	0.027120138	0.174025207
PITPNB	2.514838775	4.860393028	0.007587237	0.080981083
hsa-let-7a-3 /// hsa-let-7b /// hsa-mir-4763 /// MIRLET7BHG /// RP4-695O20__B.10	2.514435213	3.482554525	0.013478912	0.113769095
LOC101926916	2.514186746	3.468564224	0.01468201	0.120219512
TOP1MT	2.514003712	3.55585154	0.011397017	0.102936841
SMR3A /// SMR3B	2.513707455	2.285006838	0.03850259	0.217956466
ABRA	2.513072336	2.253740727	0.03850259	0.217956466
LINC00086 /// LINC00087	2.512348416	4.135676781	0.009717042	0.094601222
GPER1	2.512227762	2.752041074	0.029884417	0.185922864
RP11-461A8.4	2.511314683	3.95706947	0.00935214	0.091732971
KIAA1161	2.51035969	2.805478631	0.0262636	0.17205368
NUDT9	2.509908891	3.034451751	0.02308602	0.156863073
H2AFB1 /// H2AFB2 /// H2AFB3	2.507276782	2.917162171	0.020429716	0.146813978
POM121 /// POM121C	2.506497242	4.265477572	0.008654935	0.088631966
LOC286272	2.506166745	3.716120935	0.014436237	0.118662028
ADHFE1	2.505105601	3.794774192	0.010849845	0.10041257
HCG22	2.504764973	4.77465872	0.008148408	0.084939983
SETD1A	2.504639998	4.093653301	0.010882272	0.100529431
DNASE1L2	2.504626997	4.768862244	0.008454524	0.086920649
KIAA1432	2.504044073	3.690875883	0.011101378	0.101436631
RAB42	2.503728519	4.056344812	0.009266069	0.09096469
INHBB	2.503246677	3.314445941	0.015540812	0.123925577
SUZ12P /// SUZ12P1	2.50168628	3.877908337	0.01212097	0.106947596
MOGAT2	2.498635676	4.042881624	0.009835439	0.095555885
SH3KBP1	2.49846304	4.170491966	0.008693112	0.088984135
LINC01139	2.498364162	4.033317939	0.01044545	0.098019301
SUPT6H	2.498337668	3.17480152	0.014967264	0.121917808

RP11-217B7.2	2.4982647	3.413697003	0.017478245	0.133224833
ARHGAP5-AS 1	2.497545466	4.373949521	0.009889844	0.096044733
EGFLAM	2.495866136	4.500488352	0.008556407	0.087852762
TNKS1BP1	2.495746414	3.197636476	0.020808432	0.148626237
ANKRD65	2.495550369	3.611126671	0.013991958	0.117016402
TMLHE	2.495294542	2.416823217	0.049219036	0.251742564
THPO	2.495168779	3.471695117	0.01468201	0.120219512
PSMA3	2.493554198	2.879775248	0.023136836	0.156863073
MCAT	2.492011537	4.819620248	0.007303888	0.079094454
RP11-214N9.1	2.491853809	2.596370925	0.030962986	0.190563917
LINC00528	2.491175517	3.715602629	0.014436237	0.118662028
BLMH	2.490563716	4.973550246	0.007610664	0.081194236
LY96	2.490119857	3.392902937	0.019102998	0.140635421
APOC2 /// APOC4 /// APOC4-APOC 2	2.489450649	4.181449076	0.011284046	0.102585446
COX6B1P3 /// COX6B1P3	2.488283055	3.541700723	0.016400808	0.128421803
LINC01271	2.487009993	4.493135836	0.008945235	0.0893286
FAM212A	2.48676398	4.224875677	0.009611529	0.093846234
ZNF324	2.483365225	2.380254775	0.027120138	0.174025207
TSPY26P	2.481496806	2.7399018	0.034088492	0.202428428
C1RL	2.480761414	3.886704496	0.01212097	0.106947596
OR10H1	2.480744943	2.321017324	0.03224147	0.195096667
TCRA /// TCRA /// TRAV10 /// TRAV10 /// YME1L1	2.47934823	3.717566013	0.014436237	0.118662028
GZMK	2.479291436	3.217221305	0.020808432	0.148626237
MCAM	2.477330204	3.805014112	0.010849845	0.10041257
C16orf71	2.476842013	3.793772883	0.011639558	0.10472482
NOSTRIN	2.476150154	4.015131072	0.011099404	0.101436631
FST	2.475774039	2.964305135	0.028984011	0.18181372
FLJ37786	2.47411965	2.507955394	0.036013059	0.209203458
MGC15885	2.473302455	4.567635542	0.008897012	0.0893286
FOCAD	2.47232835	2.91013036	0.020429716	0.146813978
PDP2	2.470949145	3.304081564	0.015540812	0.123925577
TCEB1P3 /// TCEB1P3	2.46807063	2.528994382	0.036013059	0.209203458
PCDH1	2.468006524	3.324221889	0.015540812	0.123925577
OR5P2	2.46781542	2.650131739	0.026707832	0.172890711
TTTY6 ///	2.467620187	4.035986444	0.01044545	0.098019301

TTTY6B				
C1QB	2.467617116	3.719526409	0.014436237	0.118662028
LOC101929076	2.467543905	4.081157091	0.01152472	0.103810856
KCTD6	2.467507474	3.59479833	0.015141462	0.122275726
IGFBP4	2.462907423	3.441417396	0.01601018	0.126496324
PROL1	2.461789345	3.281700965	0.017104955	0.131617949
ZNF137P	2.46146514	3.71456586	0.014436237	0.118662028
DPP10	2.461423615	2.757571759	0.029884417	0.185922864
CACNB3	2.460059912	4.404740365	0.009431722	0.092474966
ELANE	2.45969737	3.569852185	0.016400808	0.128421803
PCDH11X	2.459546725	4.101098129	0.010882272	0.100529431
BEX4	2.458141234	2.595590284	0.030962986	0.190563917
ADAM10	2.458023269	3.597479533	0.015141462	0.122275726
CARS-AS1	2.457671606	3.134639666	0.016636829	0.128859999
CALHM2	2.457394896	3.167603481	0.023000594	0.156863073
KCNC2	2.457192641	3.664888474	0.016730606	0.129373072
CHAF1B	2.456683259	4.310211088	0.010005701	0.096452169
RTN2	2.456667093	3.830861735	0.013852865	0.116184043
C5orf30	2.455903223	4.115509612	0.010882272	0.100529431
WISP1	2.455256051	3.18630118	0.023000594	0.156863073
DIEXF	2.455246366	3.49240778	0.013478912	0.113769095
LINC00858	2.454911384	3.010213208	0.025843036	0.170343605
MAGI2	2.45489507	3.920946976	0.014231827	0.117644205
LOC644656	2.454662138	2.836532062	0.0262636	0.17205368
ACADM	2.453588748	3.996723265	0.011800903	0.1051695
MTM1	2.45334502	3.172623611	0.023000594	0.156863073
SLC4A1	2.452934199	4.064755869	0.012211177	0.107159124
DCST1	2.451655846	4.004477577	0.011099404	0.101436631
LOC100506047	2.451601921	3.179397604	0.023000594	0.156863073
LOC101930114	2.451121459	2.590390829	0.030962986	0.190563917
NKX2-8	2.449528819	3.510730128	0.019298276	0.141673466
PTAR1	2.44804783	3.275968442	0.017104955	0.131617949
LRIG1	2.447024604	3.223707616	0.020808432	0.148626237
SERTM1	2.445308449	3.903275732	0.011349218	0.102663005
LQFBS-1	2.44486699	3.268405719	0.018852638	0.139228598
TMEM206	2.444645518	3.038254967	0.02308602	0.156863073
SLC19A2	2.444523653	2.9276257	0.020429716	0.146813978
RP11-445H22.4	2.442809953	3.29692991	0.017104955	0.131617949
IPP	2.442751865	3.085704597	0.020661082	0.14811417
SEMA3F	2.441717385	4.722165035	0.009818906	0.095474229
PUS7	2.44154968	2.350090687	0.03224147	0.195096667
EVI2B	2.441050416	3.38245722	0.019102998	0.140635421
LOC338963	2.441050416	3.38245722	0.019102998	0.140635421
FKBP1B	2.440438985	4.434084104	0.010718026	0.099399091

IDH2	2.439373106	3.99912335	0.011800903	0.1051695
GJA5	2.439192257	4.206078005	0.01069186	0.099399091
CELF3	2.439004697	4.110318991	0.010882272	0.100529431
TTC19	2.437927809	3.168740739	0.025461672	0.169158825
PABPC1P1 /// PABPC1P1	2.437521617	3.102133118	0.018523963	0.137623322
DPH7	2.437197607	4.100229618	0.01152472	0.103810856
LINC01136	2.436551029	4.091036899	0.01152472	0.103810856
COX2	2.435859259	8.126213794	0.005838069	0.069371732
LOC102723864	2.435566809	3.550586417	0.016400808	0.128421803
LOC101928054	2.434681541	2.715721344	0.034088492	0.202428428
SMIM10	2.434640695	4.214375635	0.010135147	0.096944163
RP11-248J18.3	2.433962937	4.260948576	0.01165463	0.104767834
ALDH3B2	2.433871139	2.62488052	0.026707832	0.172890711
CHST9	2.432858255	2.692303114	0.038983062	0.218826784
RP11-388M20.6	2.432097277	3.632987835	0.012941629	0.111020849
EXOC8	2.430802966	3.734707843	0.013425963	0.113769095
PP13	2.430152903	4.50607692	0.010568105	0.098518219
LOC100507201	2.427569115	2.77466078	0.029884417	0.185922864
GOLPH3	2.426963262	5.256069281	0.007867789	0.082883454
BC044765 /// RP11-96B5.4	2.425647794	3.327521701	0.022901344	0.156863073
AARS2	2.424867288	3.497593845	0.019298276	0.141673466
CHST14	2.424248439	3.650167764	0.018033255	0.136264188
LMO4	2.424002797	2.645196153	0.026707832	0.172890711
ICAM3	2.423757792	3.396676148	0.019102998	0.140635421
VGF	2.423736233	4.20545914	0.01069186	0.099399091
ESCO1	2.423633857	4.383029937	0.010373761	0.098019301
TBX3	2.421354955	4.219387011	0.012927774	0.111020849
ZNF143	2.421326815	3.951694669	0.013362619	0.113654832
TAS2R14	2.420922566	2.5997332	0.030962986	0.190563917
AMOTL2	2.420748699	2.890284622	0.023136836	0.156863073
TNR	2.420329156	4.036717797	0.013730126	0.115443137
AP000253.1	2.419369529	2.187455584	0.046195495	0.241429268
MYO18A /// TIAF1	2.419291911	3.202068707	0.023000594	0.156863073
SNHG10	2.418606992	3.977629072	0.012553876	0.109472446
STEAP1B	2.417556071	3.713010311	0.014436237	0.118662028
CA5B	2.416134257	2.837661501	0.0262636	0.17205368
GLTPD2	2.415865947	2.331708611	0.03224147	0.195096667
TRPV6	2.415777619	3.854395411	0.013852865	0.116184043
AKR1C1 /// AKR1C2 ///	2.415608416	2.490572015	0.042028125	0.227861691

LOC101930400				
PRAMEF1 /// PRAMEF2	2.415471934	3.722405456	0.014436237	0.118662028
NR0B2	2.413432844	3.827082126	0.014824518	0.121217393
COMMD10	2.412112255	2.401775385	0.049219036	0.251742564
OR2H4P /// OR2H4P	2.411281466	3.674290574	0.016730606	0.129373072
DFFB	2.410887721	2.653599322	0.044697393	0.237724963
CDKN2AIPNL	2.410480198	2.474215237	0.042028125	0.227861691
TMEM176B	2.41024513	3.171333914	0.025461672	0.169158825
BC045560	2.409931906	2.270530539	0.03850259	0.217956466
TSPAN12	2.408120423	4.07079811	0.012211177	0.107159124
RFPL1S	2.407951149	2.974187245	0.028984011	0.18181372
AP006547.3	2.407944652	4.441426364	0.010718026	0.099399091
C7orf62	2.40773173	4.041305189	0.013730126	0.115443137
LINC00473	2.405626225	3.611865768	0.013991958	0.117016402
MEOX2	2.405092291	2.222965034	0.046195495	0.241429268
NTN4	2.404490434	3.22158208	0.020808432	0.148626237
GNL2	2.403666988	3.487236393	0.020964854	0.148764869
LOC100996517 /// LOC102724364 /// SEC22B	2.403618228	6.008546471	0.007614292	0.08119607
TSGA10IP	2.403283861	4.778266726	0.010096418	0.096916605
AGR3	2.402893914	3.204914517	0.023000594	0.156863073
LOC101928475	2.401675168	3.78654231	0.017012514	0.131207477
EXOSC2	2.401669762	2.993388772	0.025843036	0.170343605
SNORD3A /// SNORD3B-1 /// SNORD3B-2 /// SNORD3C /// SNORD3D	2.401306385	5.295655594	0.009171421	0.090148547
LOC101927043	2.401158158	3.837577397	0.013852865	0.116184043
XPNPEP2	2.40100273	2.553501096	0.036013059	0.209203458
P2RY4	2.400618858	4.380560286	0.012907207	0.111020849
SLC22A18AS	2.400490613	4.218060793	0.013623884	0.114672976
PUSL1	2.400450072	5.27617061	0.008509788	0.087450545
S100A4	2.40023629	3.378166471	0.019102998	0.140635421
IRF2BPL	2.400139048	5.773272896	0.007866516	0.082883454
TXLNG	2.399725383	2.584130139	0.030962986	0.190563917
OXTR	2.399354313	4.248029692	0.012272237	0.107574403
TMPRSS4	2.397761592	4.231365485	0.012927774	0.111020849
ZNF740	2.395888802	3.309020062	0.025121187	0.167946786
CCDC117	2.394151562	3.806452807	0.015875293	0.126292715

GNG12	2.391125577	2.874275717	0.023136836	0.156863073
LOC389641	2.390717243	4.096569492	0.014874623	0.121542484
POM121C	2.390705642	4.440693585	0.010718026	0.099399091
CLEC11A	2.389383183	4.638766383	0.010729379	0.099399091
STIL	2.389185751	4.459315881	0.012610629	0.109511114
SNRPB	2.388139714	3.621384701	0.019453666	0.141971273
AC100830.4	2.388011057	2.225838072	0.046195495	0.241429268
SH3GL1	2.38676759	4.925905185	0.010852525	0.10041257
C4orf46 /// TOMM7	2.385500268	9.131621955	0.006619537	0.074654898
BLOC1S1	2.385372822	3.535557338	0.017781944	0.13505769
PAX9	2.382978307	2.832500967	0.0262636	0.17205368
ATXN1L	2.382776493	2.945926309	0.028984011	0.18181372
ARHGEF26-AS1	2.382456991	3.008162892	0.025843036	0.170343605
KCNK12	2.381517963	2.440555616	0.049219036	0.251742564
SNORA78	2.38068544	3.263195309	0.018852638	0.139228598
EIF2B5	2.380494165	4.767443863	0.010472754	0.098019301
SLC35B4	2.379125672	2.480745231	0.042028125	0.227861691
RP11-108B14.5	2.378911972	2.933853609	0.032569892	0.196087109
CLDN14	2.378846881	3.505619786	0.019298276	0.141673466
GPR22	2.378482121	2.951752456	0.028984011	0.18181372
LOC101928539	2.378195778	3.275908559	0.018852638	0.139228598
KIAA1191	2.377286047	4.685866283	0.011442893	0.103271778
SBK1	2.375992294	3.552515198	0.017781944	0.13505769
RP11-363E7.4	2.374325179	3.63346218	0.019453666	0.141971273
ENTPD6	2.373781961	9.494963734	0.006821872	0.075380721
PRKX /// PRKY	2.373039494	3.699486899	0.015534855	0.123925577
SLC25A43	2.372506826	2.484199652	0.042028125	0.227861691
NPHP4	2.371415847	3.348698111	0.022901344	0.156863073
ZNF417 /// ZNF587	2.371130053	4.037966096	0.013730126	0.115443137
RAPH1	2.371044476	6.860902964	0.007641468	0.081375054
KCNN4	2.370630138	3.653215954	0.018033255	0.136264188
EIF4A2 /// MIR1248 /// SNORA4 /// SNORA63 /// SNORA81 /// SNORD2	2.370489454	6.952194848	0.007460348	0.079735439
MS4A15	2.370487753	2.773183096	0.029884417	0.185922864
BC037833 /// RP11-492E3.2	2.370077931	3.947707618	0.014231827	0.117644205

LOC730961	2.370052572	3.368229457	0.020903485	0.148764869
SERPINE1	2.369449861	3.443083979	0.024818572	0.166629867
FZD3	2.367419697	4.287397211	0.013920106	0.116581512
TACC2	2.366333443	4.683846279	0.011442893	0.103271778
ASAP3	2.365960077	4.887115161	0.0106124	0.098891911
DNLZ	2.365511684	4.670594019	0.011895851	0.105935301
LMOD1	2.365407463	2.799550099	0.029884417	0.185922864
SOHLH2	2.365012721	3.818670634	0.014824518	0.121217393
IRF5	2.364871475	2.87356052	0.036672434	0.211314202
CRELD1	2.364187974	3.766982856	0.018244259	0.136997112
TXLNA	2.364000179	3.518688253	0.019298276	0.141673466
BCAR3	2.363956118	3.011434766	0.025843036	0.170343605
PET100	2.363461206	5.902463644	0.00842378	0.086642489
PDZD3	2.363406203	2.949348128	0.028984011	0.18181372
LOC101930405	2.36308468	2.294282727	0.03850259	0.217956466
TP53I13	2.36300632	3.278605525	0.027591054	0.175721803
PIM2	2.36125332	4.348429718	0.014192157	0.117644205
LOC100507530	2.360654641	3.491293244	0.020964854	0.148764869
C19orf81	2.36053294	2.825459088	0.0262636	0.17205368
CAMKMT	2.359680315	3.496755935	0.020964854	0.148764869
C18orf49 /// ZCCHC2	2.359504217	5.275820983	0.00991517	0.096245167
MACROD2	2.358303856	3.790569897	0.017012514	0.131207477
RP11-687F6.1	2.357793076	3.385667592	0.019102998	0.140635421
ANKRD39	2.35699542	2.473003454	0.042028125	0.227861691
HTR5A	2.35699542	2.473003454	0.042028125	0.227861691
MRPL35	2.356674456	3.955254129	0.017468545	0.133224833
MTUS1	2.356653245	3.911363851	0.015166636	0.122275726
B3GNT8	2.356299192	4.008565955	0.015470278	0.123825475
SACS-AS1	2.35564961	3.908213736	0.01617267	0.127354276
ZSCAN26	2.35554059	3.696455355	0.016730606	0.129373072
TP63	2.355414443	3.487175288	0.020964854	0.148764869
ZSWIM3	2.355301399	3.963223588	0.017468545	0.133224833
PILRA	2.354678009	2.80145192	0.029884417	0.185922864
ANKRD13B	2.354573798	6.413959747	0.008346794	0.08588825
FFAR1	2.354492707	3.063430277	0.02308602	0.156863073
NXNL2	2.353722807	2.968747011	0.028984011	0.18181372
LOC102724364 /// SEC22B	2.352621348	4.682310514	0.011895851	0.105935301
VPS33B	2.352140385	4.236145292	0.012927774	0.111020849
ATP6V0E1	2.352061167	6.445955789	0.008532724	0.087647904
LOC100130872	2.351294714	4.601704749	0.012614037	0.109511114
WDR73	2.350218832	4.346420674	0.014889395	0.121620886
TAPBPL	2.349080037	2.515718206	0.036013059	0.209203458

MAML1	2.348609732	5.171485121	0.010631344	0.099029152
POT1-AS1 /// RP11-3B12.1	2.347875006	5.345212029	0.010289807	0.097684319
ZNF418	2.347634996	3.657316967	0.018033255	0.136264188
ETFDH	2.347364522	2.212144968	0.046195495	0.241429268
CCL16	2.346131875	4.035069106	0.014570392	0.11955559
LOC100128239	2.343772078	4.100160251	0.014874623	0.121542484
DDX46	2.343432973	2.523493716	0.036013059	0.209203458
LOC643733	2.343257964	3.079514315	0.031346675	0.191719888
TNFSF10	2.342213192	3.257685256	0.027591054	0.175721803
FGF22	2.341108019	2.411590629	0.049219036	0.251742564
PGRMC1	2.340904486	2.781287245	0.029884417	0.185922864
AP1S3	2.339086468	2.840285118	0.0262636	0.17205368
LOC100653086	2.338713053	4.996475006	0.012027834	0.106947596
DLG5-AS1	2.338300569	3.075732936	0.031346675	0.191719888
KIT	2.336881725	3.745254966	0.019579449	0.142667623
CREG2	2.336656494	3.472466791	0.022798603	0.156863073
LOC101927513	2.33656672	3.459873171	0.022798603	0.156863073
IL27	2.336303289	4.825989876	0.01261285	0.109511114
CRYBA2	2.334099365	5.182975828	0.010338436	0.098010712
SNRNP35	2.333736565	3.554993794	0.017781944	0.13505769
LCN1	2.333370389	3.312184341	0.025121187	0.167946786
LARGE	2.333326513	2.629797074	0.044697393	0.237724963
LOC102725017	2.332983927	3.813040121	0.015875293	0.126292715
PRSS8	2.332341816	3.588808957	0.022697535	0.156863073
C3orf36	2.331834291	3.841488865	0.019682782	0.143198484
EVPL	2.331201877	3.102549515	0.031346675	0.191719888
TBC1D19	2.331201877	3.102549515	0.031346675	0.191719888
SOX8	2.330875424	2.841762528	0.0262636	0.17205368
URB1	2.330582854	3.532760461	0.019298276	0.141673466
PPP4R2	2.330372305	2.525811245	0.036013059	0.209203458
STX1A	2.330196906	4.723524211	0.012614382	0.109511114
CSAD	2.330174543	2.509978813	0.042028125	0.227861691
ATP6AP1	2.329442065	3.877844152	0.017256092	0.132477161
LOC153684	2.328469153	3.799530609	0.017012514	0.131207477
NMB	2.328282361	4.182738243	0.01514944	0.122275726
AX746968	2.328144978	4.553336505	0.014279166	0.117783062
FAM99B	2.326242362	2.499241425	0.042028125	0.227861691
C8orf44	2.325878036	2.211712446	0.046195495	0.241429268
CTDNEP1	2.325832041	3.066238944	0.034864293	0.205168327
GCLC	2.325808659	3.942604294	0.018577866	0.137849407
PPP1R3F	2.32553376	3.249619723	0.030342931	0.188127771
GCOM1 /// POLR2M	2.324921798	3.620808809	0.021003933	0.148764869

KIAA0513	2.324300303	3.901797888	0.01617267	0.127354276
TRNAU1AP	2.324173539	3.998048263	0.016434585	0.12856519
PCSK7	2.323365015	3.55857335	0.024549514	0.165201143
C1QC	2.322344277	3.498203144	0.020964854	0.148764869
TNFSF18	2.322249297	3.384479691	0.020903485	0.148764869
FIBIN	2.322024994	3.580694152	0.022697535	0.156863073
LOXL2	2.321821839	3.934149887	0.019768773	0.1436906
TNPO2	2.321740093	6.430258418	0.009056878	0.0893286
CALN1	2.320259484	4.020001806	0.015470278	0.123825475
SPATA41	2.319909615	3.140979372	0.028229227	0.178509016
LY6G6D /// LY6G6F	2.318298869	3.060514524	0.034864293	0.205168327
TBC1D8	2.318211604	3.683055548	0.016730606	0.129373072
RITA1	2.317456295	3.29292974	0.027591054	0.175721803
FZD1	2.317211142	3.086568431	0.031346675	0.191719888
AKAP8	2.315928531	4.522412528	0.013452725	0.113769095
MAP3K4	2.314785782	4.4279768	0.014442841	0.118674788
VCPKMT	2.313914291	3.597133341	0.022697535	0.156863073
CD300LG	2.313458353	3.886194931	0.017256092	0.132477161
RAP2C-AS1	2.310430361	3.5249017	0.019298276	0.141673466
LINC01365	2.310258688	4.556537755	0.014279166	0.117783062
LOC101928845	2.308783415	3.593104336	0.022697535	0.156863073
BC038194 /// RP11-550P17.5	2.308617799	3.870669556	0.018423658	0.137516572
LOC101927603	2.306812715	4.75652875	0.013322834	0.113500488
FAM135A	2.305061126	3.826649404	0.021041606	0.148764869
ACTG2	2.304984546	3.714814243	0.021027945	0.148764869
RPL23AP22 /// RPL23AP22	2.30460042	2.530433989	0.036013059	0.209203458
LOC100130741	2.303919702	3.483813212	0.022798603	0.156863073
TMEM68	2.301945712	2.973100245	0.028984011	0.18181372
SLC25A12	2.301892688	2.66050029	0.044697393	0.237724963
MRPS18C	2.300917206	2.787575328	0.029884417	0.185922864
PPP1R14D	2.30030406	4.482310922	0.01533299	0.122936042
GOLPH3L	2.300001709	2.503952585	0.042028125	0.227861691
AGFG2	2.29934809	2.855696994	0.041376294	0.227044057
LINC01116	2.298942678	3.140327823	0.028229227	0.178509016
GRIN2A	2.298613352	2.533771283	0.036013059	0.209203458
ZNF329	2.297661569	3.561591013	0.024549514	0.165201143
DMRT2	2.297431323	3.371777788	0.020903485	0.148764869
LOC101927972	2.296944972	2.943482543	0.032569892	0.196087109
KCND3	2.295719429	2.650723592	0.044697393	0.237724963
PAK1IP1	2.295390265	3.638092628	0.019453666	0.141971273
NR2F1 ///	2.29518304	3.878624853	0.017256092	0.132477161

NR2F2				
ITPK1	2.293669774	4.251285641	0.016205458	0.127354276
CAPN5	2.292048389	3.112022746	0.031346675	0.191719888
MPO	2.291719756	2.438517687	0.049219036	0.251742564
LAMP2	2.291621931	2.749788778	0.034088492	0.202428428
PRR15L	2.291284782	5.562934265	0.011669487	0.104767834
DCUN1D5	2.290876041	3.329277155	0.025121187	0.167946786
HIAT1	2.289669668	3.532202017	0.026576667	0.172890711
LINC00668	2.288961456	3.177793617	0.025461672	0.169158825
TRUB2	2.28709282	3.553330612	0.024549514	0.165201143
AR	2.286784506	4.77434516	0.012845211	0.110900293
SYTL3	2.286587776	2.905002786	0.036672434	0.211314202
LOC100505549	2.286082203	2.909053388	0.036672434	0.211314202
IGHA1 /// IGHG1 /// IGHM /// IGHV4-31	2.285317316	4.077594888	0.016666963	0.129050858
RP11-534L20.5	2.284945466	2.788608836	0.029884417	0.185922864
HOXB1	2.284909214	2.943133429	0.032569892	0.196087109
LOC101927877	2.284601376	4.192900912	0.01514944	0.122275726
KMT2D	2.282457441	3.030147895	0.038840397	0.218652915
CLCN1	2.28141941	5.207867333	0.012599102	0.109511114
LINC01348	2.281333231	2.64552372	0.044697393	0.237724963
SRCIN1	2.281098966	2.890531105	0.036672434	0.211314202
MAF	2.281091641	2.452647165	0.049219036	0.251742564
CASC7	2.280921016	3.921794097	0.021048068	0.148764869
C10orf88	2.280536353	3.899034365	0.02242318	0.156300628
FCGR2C	2.279029518	2.24483283	0.046195495	0.241429268
ZNF460	2.278473667	2.929661795	0.032569892	0.196087109
LOC101927653	2.277539821	4.4700394	0.016025581	0.126496324
C22orf15	2.277184119	3.336455254	0.022901344	0.156863073
MAPK1IP1L	2.275931262	5.543021963	0.011276178	0.102585446
HS3ST3A1	2.273156693	3.042538837	0.038840397	0.218652915
LOC202181	2.272898416	3.272498571	0.027591054	0.175721803
TNFRSF8	2.272509386	2.779445485	0.034088492	0.202428428
TCTN1	2.272462076	3.984704267	0.017468545	0.133224833
CCRN4L	2.27240826	4.393742912	0.016587537	0.128859999
MSH6	2.27181277	3.809950089	0.02250908	0.156583748
CAPN11	2.271147797	2.958517887	0.032569892	0.196087109
SERTAD2	2.270494563	3.80731498	0.02250908	0.156583748
PBX1	2.270108882	2.858378948	0.041376294	0.227044057
JADE1	2.269260586	3.113969578	0.031346675	0.191719888
RPTN	2.268566869	2.409947893	0.049219036	0.251742564
ICMT	2.268257889	4.500816604	0.017648839	0.134307266

LOC93444	2.267780026	3.675128786	0.024309658	0.163962815
AKAP2 /// PALM2 /// PALM2-AKAP2	2.26746233	2.635458905	0.044697393	0.237724963
EFCAB4B	2.266631683	3.954354945	0.018577866	0.137849407
SEMA6A	2.265759146	3.664104919	0.024309658	0.163962815
TMEM258	2.265267537	8.646071086	0.009708189	0.094593434
C1QTNF9B-AS1	2.265061596	5.141790705	0.013903006	0.116521378
FAR1	2.264226525	3.800777402	0.02250908	0.156583748
NONO	2.263899556	5.140842508	0.013903006	0.116521378
ISCU	2.263479202	6.983480843	0.010298929	0.097684319
SLC37A2	2.263166609	3.862493427	0.018423658	0.137516572
SLC15A2	2.26250437	3.858905182	0.019682782	0.143198484
PREPL	2.262029921	4.30951253	0.017229297	0.132477161
TMEM65	2.261258477	2.881723936	0.036672434	0.211314202
LOC101928378	2.260612295	3.994990253	0.021049482	0.148764869
ANKRD54	2.26026477	3.032840901	0.038840397	0.218652915
RP11-85A1.3	2.257071262	4.525199023	0.016201814	0.127354276
SMCO4	2.256884715	8.14846233	0.01007477	0.096916605
GUSBP1	2.256212926	3.902225469	0.02242318	0.156300628
TBXAS1	2.256182638	2.219944701	0.046195495	0.241429268
C21orf15	2.255920533	3.596296724	0.022697535	0.156863073
PAK4	2.255039188	4.726349668	0.015448794	0.123737833
NACC1	2.253403	4.134163929	0.018828856	0.139228598
GPC6	2.252983202	4.083613412	0.021047344	0.148764869
ACAD11 /// NPHP3 /// NPHP3-ACAD11	2.250857434	4.454650563	0.01675433	0.129428717
TMEM218	2.247869328	4.012560792	0.021049482	0.148764869
CTTN	2.246547307	4.869058356	0.01590741	0.126496324
LOC340184	2.245707079	3.744110158	0.019579449	0.142667623
SLC25A4	2.245266646	4.495156971	0.017648839	0.134307266
ORAOV1	2.244964206	2.749658786	0.034088492	0.202428428
MESDC2	2.244955137	4.045461994	0.018711703	0.138711054
DHTKD1	2.244860818	3.861766481	0.019682782	0.143198484
GLIPR1	2.244849242	3.723594469	0.021027945	0.148764869
CNKSR1	2.244554983	2.639730444	0.044697393	0.237724963
DACH2	2.244496355	3.64116793	0.026168335	0.172042207
MAS1	2.244110821	3.172964192	0.025461672	0.169158825
ELSPBP1	2.243675221	4.490920099	0.018427642	0.137516572
LOC101927164	2.243204249	3.067369597	0.034864293	0.205168327

BC032026 /// RP11-526I2.1	2.242762345	2.658971211	0.044697393	0.237724963
SOS1	2.241025333	3.415558082	0.027046234	0.174025207
LOC102725072 /// LOC102725315 /// LOC727983 /// POM121L10P /// POM121L1P /// POM121L4P /// POM121L8P /// POM121L9P	2.240431622	3.422446171	0.027046234	0.174025207
GYPB	2.239703379	3.330607124	0.025121187	0.167946786
BZRAP1	2.239427784	3.226196183	0.033413362	0.199731986
MESP1	2.239404549	5.292968236	0.014469079	0.11884881
ACTL6B	2.237354399	2.505102211	0.042028125	0.227861691
SLC2A14 /// SLC2A3	2.236481801	5.678978838	0.0135951	0.114553831
ARMCX1	2.236268401	4.461097089	0.01675433	0.129428717
P2RX6	2.236268401	4.461097089	0.01675433	0.129428717
LOC102723542	2.235344814	2.480978641	0.042028125	0.227861691
ZNF516	2.234738752	3.715686983	0.022600656	0.156863073
MADCAM1	2.233811078	4.028580262	0.019841152	0.144127499
CASKIN2	2.233506902	3.02078312	0.038840397	0.218652915
ASL	2.23306146	3.857021053	0.025494123	0.169230839
WBP5	2.232089942	3.662835231	0.026168335	0.172042207
PFKFB1	2.23062761	3.256181208	0.030342931	0.188127771
MLPH	2.230311283	6.772256995	0.011718767	0.104835213
TMPO-AS1	2.230105101	4.041698734	0.018711703	0.138711054
PPP2CB	2.227499758	5.302997489	0.014105303	0.117459486
CUL4A	2.226343329	3.995527274	0.02234293	0.155935865
TRIM69	2.224734372	3.714516036	0.022600656	0.156863073
RANBP3	2.224668121	3.558278139	0.026576667	0.172890711
INPPL1	2.22413058	4.122236383	0.018828856	0.139228598
PXMP4	2.223674471	3.666577784	0.026168335	0.172042207
LOC100128840	2.222796513	4.61508708	0.017873842	0.135508306
SNRNP70	2.221281527	3.212432016	0.033413362	0.199731986
PDC	2.221222868	4.297046664	0.018101124	0.136335879
HTR1B	2.22107274	3.56923433	0.024549514	0.165201143
C1QTNF9 /// C1QTNF9B	2.220467743	3.524349111	0.028797772	0.181420557
HOXA13	2.220209585	4.093810858	0.021047344	0.148764869
LOC100130219	2.219350874	4.417150676	0.019179603	0.141155146

COPS7A	2.219156569	2.682109988	0.044697393	0.237724963
AC016831.7	2.218875132	4.125579995	0.018828856	0.139228598
SACM1L	2.218797881	3.703249182	0.022600656	0.156863073
NTN1	2.216805	4.329773195	0.020040916	0.144802785
MIR7109 /// PISD	2.215876997	3.289543788	0.027591054	0.175721803
KCTD12	2.215435826	3.824955579	0.021041606	0.148764869
ZNF286A /// ZNF286B	2.214996785	4.357333909	0.018220645	0.136997112
RPUSD2	2.214962072	3.710205719	0.022600656	0.156863073
SLC6A1	2.214962072	3.710205719	0.022600656	0.156863073
TCF12	2.214951957	3.56814043	0.024549514	0.165201143
ENOX2	2.212945235	3.507233502	0.028797772	0.181420557
HOXC10	2.212370641	3.273650988	0.030342931	0.188127771
GAGE1	2.210197109	2.995426487	0.043342751	0.233159586
NOVA1	2.209773315	3.166179406	0.040683222	0.225325984
TCF19	2.209739797	3.686188528	0.024309658	0.163962815
NUP93	2.209560225	3.769040754	0.025810337	0.170343605
SLC6A20	2.207828192	3.309527556	0.027591054	0.175721803
SPEN	2.207592971	3.607214091	0.03039573	0.188206768
HNRNPA1 /// HNRNPA1P10	2.2050924	9.005023424	0.011492845	0.103642961
HADHA	2.204090305	3.430698111	0.027046234	0.174025207
CR2	2.203369809	3.105686733	0.031346675	0.191719888
LNX2	2.202840925	4.308855366	0.021028739	0.148764869
UCP3	2.20027125	3.244423773	0.033413362	0.199731986
RPS11	2.200037258	9.378541795	0.011576809	0.104240095
TPBGL	2.19950333	3.415243235	0.027046234	0.174025207
DPM3	2.199355078	6.001593282	0.013582216	0.114486336
RP11-764E7.1	2.198021009	3.109060466	0.031346675	0.191719888
CYBA	2.197682869	2.861191067	0.041376294	0.227044057
RPL18P10 /// RPL18P10	2.195403189	5.073694967	0.017503211	0.133371859
BC038731 /// CTD-2284J15.1	2.19516168	2.850631439	0.041376294	0.227044057
LOC100506548 /// RPL37	2.195006708	3.000048142	0.043342751	0.233159586
CCDC124	2.193908801	3.038241349	0.038840397	0.218652915
DUX1	2.19380764	3.091117449	0.034864293	0.205168327
ARHGEF37	2.193801161	4.871932833	0.016455039	0.128668079
SOCS2-AS1	2.193640264	3.526038745	0.028797772	0.181420557
LUZP2	2.192304392	4.694272539	0.017300635	0.132732368
LOC100288310	2.191713561	2.916404363	0.036672434	0.211314202
INTS5	2.191454334	5.021424948	0.015797819	0.125889542

NFIA	2.19131913	4.805541087	0.016909796	0.130543848
METTL20	2.189848679	2.999381401	0.043342751	0.233159586
RP11-521O16.2	2.189731911	4.399644753	0.0200758	0.144891153
CERS4	2.189726218	3.269707801	0.030342931	0.188127771
PEX19	2.187867096	3.40034998	0.029505815	0.184371149
AP2A1	2.18786441	4.440324061	0.021963259	0.154710909
CDC37P1 /// CDC37P1	2.187255629	2.447117353	0.049219036	0.251742564
C8orf82	2.187064179	3.417677546	0.027046234	0.174025207
SLC52A2	2.186795338	3.331833668	0.035233806	0.206824731
PLA2G4D	2.184787173	3.636423112	0.028191532	0.178509016
NDUFAB1	2.184769886	6.345913002	0.014098212	0.117459486
SH2D2A	2.184727976	3.989888766	0.02234293	0.155935865
KNDC1	2.184129059	4.304867263	0.022072778	0.155203144
TMCC2	2.183326005	3.545572589	0.026576667	0.172890711
LINC01105	2.182640527	2.853071243	0.041376294	0.227044057
RNF126P1	2.181742189	3.538904589	0.026576667	0.172890711
LRRC8D	2.18105281	4.361865383	0.023066449	0.156863073
OR5P3	2.180869835	3.559917072	0.026576667	0.172890711
MYCN	2.180725692	3.222931659	0.033413362	0.199731986
KRT35	2.180092464	4.385854689	0.021020343	0.148764869
C7orf73 /// LOC101930655 /// SLC13A4	2.179062602	2.864821489	0.041376294	0.227044057
MRPL45P2	2.179051259	3.19441498	0.036844159	0.211314202
EFR3A	2.17857492	5.113185384	0.017583623	0.133897719
GNGT1	2.177250434	3.65094231	0.028191532	0.178509016
LOC100505835	2.176520204	3.839687332	0.02720858	0.174361002
VAMP2	2.175873621	3.626125471	0.028191532	0.178509016
TPRN	2.175299452	6.000251903	0.014784627	0.121017578
FAM168B	2.175111947	4.029908589	0.02644669	0.172890711
AC025442.3	2.174798174	3.623584906	0.03039573	0.188206768
PPP1R14C	2.174260429	4.1915285	0.019955481	0.144802785
SQRDL	2.174168621	3.061553306	0.038840397	0.218652915
LOC151657	2.173898084	2.919893266	0.036672434	0.211314202
RRS1-AS1	2.172455541	3.782242452	0.024095046	0.162702163
NKD1	2.171196819	3.556066859	0.026576667	0.172890711
MN1	2.171017522	2.833001794	0.046781564	0.243463117
HOXB-AS3	2.170641788	3.703271796	0.03185775	0.194188444
WDR76	2.170608056	2.657060066	0.044697393	0.237724963
CTSK	2.170386271	3.42853488	0.027046234	0.174025207
ZNF395	2.169685744	4.973639807	0.01844333	0.137589936
GPR65	2.16816318	4.776344879	0.018812459	0.139228598
RP11-629E24.2	2.165849175	3.156765652	0.040683222	0.225325984

FGF5	2.165022567	3.183890784	0.036844159	0.211314202
SDF4	2.164803015	4.419016487	0.022964829	0.156863073
TMEM209	2.16231458	4.362519832	0.023066449	0.156863073
SLC16A6	2.157792847	3.807258615	0.031048896	0.190992583
BRI3BP /// THRIL	2.157457652	4.000397017	0.028034104	0.177897672
USB1	2.157144997	2.966808935	0.048450223	0.249548921
LINC00599 /// MIR124-1	2.157005227	4.01961083	0.02644669	0.172890711
ALMS1P	2.157000274	4.128230151	0.024734938	0.166353609
CS	2.156923284	4.578416026	0.020992821	0.148764869
CYSRT1	2.155640233	4.556005719	0.021867255	0.154265936
PNMA2	2.154831013	3.21021288	0.036844159	0.211314202
TAS2R3	2.153276656	3.281108181	0.04226773	0.228685747
ADCK1	2.150908191	2.90305388	0.036672434	0.211314202
CABP5	2.149007835	3.870615565	0.025494123	0.169230839
CHDC2	2.147571158	4.607666669	0.022702637	0.156863073
LOC101929752	2.146780228	3.128483279	0.044985511	0.238017935
METTL5	2.146648446	4.090941571	0.027608938	0.175721803
ROR1-AS1	2.1463997	3.851360189	0.02720858	0.174361002
TAP2	2.145729099	3.747662492	0.027666888	0.175852415
MTRF1	2.145164828	3.659483253	0.026168335	0.172042207
PLIN3	2.144418897	3.982314489	0.023728282	0.16050239
PLCB4	2.144021216	3.305766012	0.038568237	0.217956466
KRTAP4-8	2.143721654	3.831850767	0.029056273	0.18212112
LEAP2	2.14367548	2.993370399	0.043342751	0.233159586
ROM1	2.143162632	4.248478529	0.022133394	0.155536253
CTDSP1	2.142552882	4.951883744	0.019641357	0.143074344
MPV17	2.142297511	4.613788339	0.022702637	0.156863073
YPEL2	2.142100931	4.089832632	0.027608938	0.175721803
XKR7	2.140210429	3.258448479	0.033413362	0.199731986
NGFRAP1	2.139639151	7.695021972	0.014524215	0.11926
LOC101929897	2.139309235	5.427554214	0.017276654	0.132591689
ENPP2	2.139159003	3.351325523	0.035233806	0.206824731
RP11-391M1.4	2.138404152	2.973987087	0.048450223	0.249548921
ADRB2	2.133715046	4.847950092	0.020946711	0.148764869
LOC102723918	2.133331948	3.576279197	0.035422422	0.207568856
UBXN10-AS1	2.132347938	7.65823784	0.014942868	0.121917808
SHISA5	2.131453835	4.714043154	0.022556521	0.156863073
RP11-629O1.2	2.13135533	3.915657252	0.028512962	0.179915793
KIF4A	2.131025592	4.447678601	0.021963259	0.154710909
CLYBL	2.130812831	3.60512232	0.032799238	0.197114275
ACOX3	2.130082593	3.499417621	0.031233847	0.19167856
WDR60	2.129776092	4.439042759	0.021963259	0.154710909

AF131215.4	2.129079006	3.757862394	0.027666888	0.175852415
PDHB	2.128155863	3.514207434	0.031233847	0.19167856
MRPL32	2.127598031	4.262401493	0.02557964	0.1697026
TLR10	2.126456567	3.412605402	0.040082012	0.223447227
LOC100101148 /// LOC541473	2.126213457	5.703320738	0.017124225	0.131723084
SERPINF2	2.125735927	3.935764245	0.026804893	0.173423448
LOC153811 /// RNF130	2.125602627	4.059692029	0.024961318	0.167349109
FAM153A	2.125422138	4.247672354	0.02688736	0.17381339
KIF3C	2.124353985	3.966692867	0.025212922	0.168363261
B9D2	2.123066953	4.042772229	0.024961318	0.167349109
IARS2	2.121516562	2.814443132	0.046781564	0.243463117
UNK	2.121452208	3.57410215	0.035422422	0.207568856
EFTUD1	2.120173501	3.915184813	0.028512962	0.179915793
ZNF793	2.11758074	3.215534387	0.036844159	0.211314202
CHRM4	2.117386136	3.175550704	0.040683222	0.225325984
C18orf12	2.117249864	3.148285177	0.044985511	0.238017935
ZBTB12	2.117249864	3.148285177	0.044985511	0.238017935
ZDHHC14	2.11602808	4.03729059	0.02644669	0.172890711
KIAA1656	2.11528547	4.537413975	0.023744377	0.160565029
SGSM1	2.114949799	3.042523034	0.038840397	0.218652915
MTA1	2.114640585	4.817439969	0.022428461	0.156300628
INO80	2.114055855	3.039414559	0.038840397	0.218652915
SPTY2D1	2.113771558	3.576814035	0.035422422	0.207568856
LOC101927579 /// LOC101928042	2.113440197	4.404921629	0.025129106	0.167946786
HCLS1	2.112312967	5.052574521	0.022168436	0.155735915
LOC284661	2.11197423	3.933156903	0.028512962	0.179915793
ST6GALNAC6	2.110956278	4.735410871	0.021743958	0.153442195
RP11-38P22.2	2.108842141	4.341778376	0.025343964	0.168950169
TTC9C	2.108721887	3.8857415	0.032316502	0.195262157
IFNL2	2.10812233	3.546673537	0.038287949	0.217787043
RP11-384L8.1	2.106981841	3.099004703	0.049814168	0.253571923
FOXP4	2.106885276	3.591921434	0.032799238	0.197114275
KRTAP4-1	2.106710751	3.712993832	0.03185775	0.194188444
FCGR2A	2.105640354	3.866691071	0.034433792	0.204066257
CHRNA10	2.105456737	3.527170213	0.038287949	0.217787043
STAM2	2.104767113	3.564829119	0.035422422	0.207568856
HLA-DMB	2.10443671	4.319981673	0.026578631	0.172890711
MRPS26	2.103945781	3.743099555	0.029677854	0.185324864
RP11-793H13.11	2.102889639	4.916167753	0.022315336	0.155835874

TM9SF3	2.100532506	3.524138766	0.041421069	0.227044057
PAK6	2.099554422	3.9275607	0.028512962	0.179915793
TMEM63B	2.099158349	6.139518894	0.018221961	0.136997112
GSDMC	2.098841573	3.805941885	0.031048896	0.190992583
LOC284600	2.098385341	4.639362956	0.024431141	0.164687622
HTR1A	2.097954596	3.14763666	0.044985511	0.238017935
HOXD1	2.097822401	3.140739877	0.044985511	0.238017935
MIR7114 /// NSMF	2.096667076	4.23816573	0.028272149	0.17868424
RAB27B	2.096417937	5.250594852	0.021970063	0.154712445
LINC00628	2.096157734	3.821180051	0.029056273	0.18212112
PAGR1	2.095767564	4.40457268	0.025129106	0.167946786
MRPL33	2.094065176	7.678067379	0.016321624	0.128022567
C11orf95	2.093591949	3.540848793	0.038287949	0.217787043
CCDC12	2.092212623	3.635289848	0.039579885	0.221383488
LHX4-AS1	2.091763973	3.28523284	0.04226773	0.228685747
MESP2	2.091186136	4.330966949	0.025343964	0.168950169
CELSR3-AS1	2.090718546	3.044197636	0.038840397	0.218652915
OTP	2.090063352	3.214585465	0.036844159	0.211314202
COQ4	2.089759917	3.107289985	0.049814168	0.253571923
AJUBA	2.089321256	3.316921261	0.038568237	0.217956466
LRRC37A2	2.089039667	4.100169278	0.027608938	0.175721803
GLI4	2.088975091	2.866296827	0.041376294	0.227044057
KCNH3	2.088944767	3.343504722	0.035233806	0.206824731
XAGE-4	2.088527197	4.708334857	0.023404061	0.158537544
FUBP3	2.087776917	3.377708125	0.043643943	0.234031684
UNC80	2.087193244	3.381117122	0.043643943	0.234031684
MIEN1	2.086096923	5.659020811	0.02000023	0.144802785
PLA2G2A	2.085986278	4.249124216	0.02688736	0.17381339
TTF1	2.083830751	3.309807339	0.038568237	0.217956466
RXRG	2.082197049	4.955809545	0.022971639	0.156863073
ACAP3	2.081289555	3.607391925	0.032799238	0.197114275
SEC61G	2.081048858	6.305893654	0.018700212	0.138711054
LOC221946	2.080836301	3.349222779	0.035233806	0.206824731
RPS28	2.080743437	10.01722497	0.016373499	0.128386597
MICA	2.080003377	3.6738615	0.034222603	0.203019542
ZDHHC16	2.079732758	5.00404844	0.022900141	0.156863073
FBXO4	2.079172986	3.006517446	0.043342751	0.233159586
TJAP1	2.07885236	3.529694057	0.041421069	0.227044057
TECTB	2.078232004	3.718865821	0.03185775	0.194188444
DBR1	2.077888893	2.968214978	0.048450223	0.249548921
KCNJ3	2.076728728	3.08611297	0.049814168	0.253571923
DCAF4	2.07620208	3.839843955	0.036710999	0.211314202
PPP2CA	2.075437167	3.635446903	0.039579885	0.221383488

SSTR2	2.07505941	3.340990852	0.038568237	0.217956466
LOC101929538	2.073532726	4.436357361	0.027209579	0.174361002
HMX2	2.071043523	3.695021658	0.034222603	0.203019542
OR52K3P	2.069535334	3.268694244	0.046377407	0.241746369
SLC45A2	2.068061959	5.015516923	0.022214697	0.155835874
JADE2	2.067020874	3.563360028	0.035422422	0.207568856
KITLG	2.066672121	3.525677968	0.041421069	0.227044057
TRIB2	2.06632971	4.161814605	0.028704807	0.180980493
GRP	2.066275683	4.296284303	0.029260307	0.183204462
CYP27A1	2.065591492	4.603641162	0.027454964	0.175311774
LINGO1	2.063835876	5.609357523	0.021665243	0.152978629
MCCC2	2.063593156	4.674591872	0.026173968	0.172042207
LINC01314	2.06314764	4.237709415	0.028272149	0.17868424
CD247	2.062358763	3.641500549	0.039579885	0.221383488
BUD13	2.062016761	4.816958115	0.023214157	0.157296504
LOC441025	2.061610564	3.768704294	0.035521524	0.207696708
NCKAP5L	2.061122264	3.015346744	0.043342751	0.233159586
ANKFY1	2.061086513	2.985973465	0.048450223	0.249548921
PRAC1	2.060736915	7.430409534	0.018199534	0.136989253
CUX2	2.060547086	3.262029055	0.046377407	0.241746369
C6orf164	2.057315133	2.990628817	0.048450223	0.249548921
TLX1	2.056843628	3.18356879	0.040683222	0.225325984
PCLO	2.055968277	4.008188182	0.029731472	0.185561037
RAMP1	2.054864554	4.624659096	0.026401793	0.172862573
HNRNPA1 /// HNRNPA1L2 /// HNRNPA1P10 /// HNRNPA1P33	2.05429158	8.333595644	0.018167865	0.136794706
PSMB4	2.053299627	5.76627407	0.021941422	0.154696246
LOC101927965 /// LRAT	2.052367527	4.57330436	0.026648616	0.172890711
TSPAN13	2.051915983	4.955221278	0.023705603	0.160395166
AURKC	2.05135119	3.166528071	0.044985511	0.238017935
GNB5	2.051296297	3.28441265	0.04226773	0.228685747
HES1	2.05040098	4.047673014	0.032666687	0.196569113
NKRF	2.050145016	4.596469257	0.028556473	0.18012624
UBIAD1	2.048745131	4.063071885	0.030871457	0.19054973
METTL7B	2.048624248	3.501731298	0.044849939	0.238017935
PTPRO	2.045990298	4.79576269	0.024882046	0.167008292
SEC24B-AS1	2.045373022	4.255352982	0.032255894	0.195096667
NAIF1	2.045317283	4.044954115	0.032666687	0.196569113
RP4-714D9.5	2.044326693	3.423870984	0.040082012	0.223447227

GRTP1-AS1 /// LOC101930007 /// LOC101930262	2.043537108	3.920060598	0.037803841	0.216027204
GFOD1	2.043309088	4.275363854	0.030716392	0.18979208
BMX	2.042911345	4.089045499	0.035597747	0.207924536
MTFR1	2.041795738	4.138347252	0.030272862	0.188090472
LOC101927268 /// LOC102724851	2.041189896	3.926051715	0.037803841	0.216027204
MAP3K8	2.040926022	3.331174566	0.038568237	0.217956466
LOC101929280	2.040716114	5.4587142	0.023771798	0.160704197
KLHL13	2.039686647	3.853276573	0.036710999	0.211314202
AX747630	2.039552831	4.072474391	0.030871457	0.19054973
LTK	2.038634723	3.425079238	0.040082012	0.223447227
DGCR5 /// LOC100287576	2.037677186	3.440613282	0.040082012	0.223447227
RAB2B	2.036705514	4.896108269	0.024596016	0.165466668
NUP153	2.036485138	3.979576734	0.031547384	0.192696604
TXN2	2.035988861	5.328832537	0.024092045	0.162702163
ESPNL	2.034201744	3.639134005	0.039579885	0.221383488
ARMC4	2.033713603	4.289217069	0.029260307	0.183204462
AMIGO1	2.033645303	3.179407408	0.040683222	0.225325984
ISG15	2.032452166	4.780822682	0.026688556	0.172890711
RAD51AP1	2.032359203	3.530536434	0.041421069	0.227044057
HIGD1A	2.032179913	4.585700476	0.029708814	0.185468899
CLDN16	2.031856272	3.152237324	0.044985511	0.238017935
CD5	2.031598979	3.674301479	0.03679012	0.211314202
HIBADH	2.031492248	3.783156695	0.035521524	0.207696708
TMEM134	2.030948915	4.305080588	0.029260307	0.183204462
SYF2	2.029150153	4.475158867	0.029301521	0.183364765
TERF2IP	2.029004536	4.795253727	0.029339375	0.183552249
PRPS1	2.028882288	4.677686607	0.027179317	0.174357324
NUBPL	2.028232278	3.671757805	0.03679012	0.211314202
HOXD9	2.028017868	3.620303124	0.042613565	0.230344653
ARL5B	2.027071554	4.343243934	0.030197178	0.187719532
EPO	2.026804395	3.720246837	0.040745019	0.225325984
SLC5A4	2.023401391	4.213260627	0.031293785	0.191719888
GALNT11	2.022840647	7.528139973	0.020375673	0.146813978
GJA4	2.021951483	3.557685585	0.038287949	0.217787043
GTF2I	2.021839348	3.839677904	0.036710999	0.211314202
RP11-254F7.1	2.021118949	3.789400023	0.033199284	0.199110946
RABIF	2.021095797	5.82923403	0.02303688	0.156863073
NDOR1	2.020520266	3.898562706	0.040195758	0.223762737

LSM3	2.018265143	4.027759918	0.034582188	0.204790715
RP11-362K14.7	2.01733089	3.998133043	0.036627028	0.211314202
TLL2	2.01699632	3.899783594	0.040195758	0.223762737
TGIF2LY	2.016801167	3.41425706	0.043643943	0.234031684
C10orf2	2.016558502	3.50856866	0.044849939	0.238017935
MSMO1	2.01636323	4.26366612	0.032255894	0.195096667
HCG4	2.016139132	5.111781695	0.027485323	0.175457936
SMTN	2.015904857	3.166426053	0.044985511	0.238017935
ALKBH2	2.015171079	3.2887263	0.04226773	0.228685747
NDRG3	2.013860741	5.501151832	0.025750692	0.170343605
FAM151A	2.012702462	3.310298231	0.04226773	0.228685747
CCDC92	2.011804881	5.145308683	0.027281841	0.174558001
TBL1XR1	2.011132583	5.842648132	0.023795996	0.160821502
TRAF7	2.010912476	3.497558266	0.044849939	0.238017935
HMGN1 /// LOC101927733	2.008118043	7.150024288	0.021363863	0.150895932
DRD5	2.007423601	4.721928719	0.027927152	0.177266864
ZNF182	2.005840311	4.12848075	0.031939616	0.194435206
ACAD9	2.00480624	6.130818957	0.02318822	0.15716609
ANKHD1	2.004151549	4.242023531	0.033884188	0.202125599
NFE2L3	2.003348051	3.457482891	0.048605977	0.249912809
FAM96A	2.002914825	5.538507396	0.025098863	0.167946786
RAB6A /// RAB6C /// WTH3DI	2.001487749	4.15795178	0.036545084	0.211314202
SMAD9	2.001121527	2.979240102	0.048450223	0.249548921
TNFRSF1B	2.00029268	4.926168623	0.029347107	0.183552249
SMIM1	1.999107268	4.103272954	0.035597747	0.207924536
CTC-471C19.1	1.998488512	4.149146544	0.036545084	0.211314202
CCDC63	1.99810786	4.665156861	0.03238781	0.195592398
PPP3CC	1.996606528	3.572832652	0.049511261	0.252687059
LPAR1	1.996276104	3.727871189	0.040745019	0.225325984
LRRC4C	1.99620393	4.042731381	0.034582188	0.204790715
CCBL1	1.995571322	3.361970464	0.047571201	0.246480515
ANKRD18B	1.994350189	3.717725294	0.040745019	0.225325984
CENPBD1P1	1.9940978	4.492323629	0.034133397	0.20264386
SLC17A6	1.993674614	4.460812419	0.031930781	0.194435206
PLXNA1	1.991427119	3.783683316	0.044644611	0.237724963
ALG1 /// NAGPA-AS1	1.990983103	3.539455924	0.041421069	0.227044057
ADARB2-AS1	1.990874246	3.679811724	0.04686269	0.243561572
SAFB	1.990852634	4.147630257	0.038514034	0.217956466
DDX11 /// DDX12P ///	1.989794202	4.534547514	0.030126659	0.187330721

LOC642846				
PIP5KL1	1.989505727	3.5961389	0.045915158	0.241252271
C6orf25	1.987957761	3.507441932	0.044849939	0.238017935
NPTN-IT1	1.987954817	3.838212156	0.039161722	0.219672197
ELMO1	1.986618399	5.001002083	0.030544718	0.188979854
STARD3	1.986581965	3.763284245	0.038031097	0.217061837
AGO1	1.985882826	4.340102847	0.031640769	0.193166566
STRA13	1.985106244	5.436299886	0.026592709	0.172890711
LRWD1 /// MIR4467	1.984773455	5.235683149	0.026908356	0.173901267
C10orf91	1.983498823	4.410310774	0.031086254	0.191122295
FOXC2	1.982212101	3.655347814	0.039579885	0.221383488
STX18-AS1	1.981840996	5.248646889	0.028128747	0.178353721
CRIPT	1.981118171	4.285376137	0.036467928	0.211314202
ATP2B4	1.98111164	4.29221756	0.036467928	0.211314202
TNFSF8	1.980414613	3.647965116	0.039579885	0.221383488
CCT8	1.979756831	3.894705638	0.040195758	0.223762737
ZNF275	1.975762091	5.672003512	0.02561136	0.16986507
RABL3	1.975580754	3.383767879	0.047571201	0.246480515
TMEM104	1.973879985	3.40399861	0.043643943	0.234031684
VPS4A	1.973448945	6.421686374	0.025387192	0.169158825
AFMID	1.972178387	4.729455164	0.031900185	0.194396664
TSPYL6	1.971915264	3.896421864	0.040195758	0.223762737
LOC101927722	1.969650054	3.924913263	0.037803841	0.216027204
LINC01420	1.968840104	4.000751423	0.03881103	0.218652915
TBC1D4	1.96793864	5.846221734	0.027556219	0.175721803
PLEKHA5	1.966796445	5.667201159	0.028056695	0.177992945
SPRR2G	1.963816476	5.583646858	0.028561648	0.18012624
RPL39	1.962874436	11.12056469	0.022893076	0.156863073
PPP1CB	1.962612025	5.437873314	0.028914191	0.18181372
CYB5A	1.96201293	4.23551265	0.035607033	0.207924536
SLC39A4	1.96190066	3.556574644	0.049511261	0.252687059
SAP30BP	1.96158372	3.723313666	0.040745019	0.225325984
AK094733 /// RP11-114G22.1	1.961454492	3.639835082	0.042613565	0.230344653
TRIM27	1.961367723	3.799369081	0.044644611	0.237724963
RILPL1	1.960792335	3.98396772	0.041144837	0.227044057
RAB5A	1.958891044	5.261708278	0.030097892	0.187201398
PTK6	1.958593003	3.799982262	0.044644611	0.237724963
PIGZ	1.958447135	3.847614628	0.048474468	0.249564364
DIAPH2-AS1	1.956088914	4.090474759	0.037604801	0.215151483
ZNF271	1.955861286	3.767976459	0.047710877	0.246823148
PPP1R13B	1.954851047	4.783498621	0.03145059	0.192255337
TMEM132B	1.954711085	4.002983349	0.03881103	0.218652915

ABRACL	1.954520313	3.36829501	0.047571201	0.246480515
PRR13	1.954481256	6.06228705	0.027646707	0.175852415
PCGF2	1.954054902	4.622962585	0.032918447	0.197678977
BC017988 /// RP11-308N19.1	1.953215949	3.511420263	0.044849939	0.238017935
COMMD9	1.952062148	3.587000958	0.045915158	0.241252271
NUP188	1.950390369	3.563874004	0.049511261	0.252687059
IMPDH1	1.94860472	5.12856845	0.029623367	0.185033803
LOC100506172	1.948087187	3.992100366	0.03881103	0.218652915
UVSSA	1.947536332	5.042121522	0.031113934	0.191242423
ZNF146	1.947478846	3.8588017	0.045516199	0.240143095
SLC36A1	1.946893489	4.02261106	0.044445535	0.237568716
ARMC10	1.943222679	4.569065781	0.037023053	0.212184819
TTC40	1.943114634	3.467575814	0.048605977	0.249912809
DGCR6L	1.942420351	6.657211801	0.02654576	0.172890711
TAAR2	1.942128734	4.032751837	0.044445535	0.237568716
HIST1H3E	1.941395122	3.729389825	0.040745019	0.225325984
ZNF169	1.941374791	4.013824054	0.047033155	0.244285401
ACKR2	1.940968751	3.560042714	0.049511261	0.252687059
CDC42EP5	1.938673523	8.168449737	0.025248482	0.168552813
YEATS2	1.938314218	3.595304904	0.045915158	0.241252271
AAMP	1.935439753	5.206282217	0.032829426	0.197245235
RP4-740C4.7	1.934516898	4.430919742	0.03483308	0.205168327
LINC01208	1.93418979	4.708658102	0.034303769	0.203449671
COX1	1.932085283	10.38476789	0.025041067	0.167835874
OTUD5	1.930655401	4.060106343	0.039742006	0.221920419
SLC52A3	1.928783341	3.690283428	0.04686269	0.243561572
LOC541472	1.924617092	4.768334789	0.036820723	0.211314202
IRX2	1.924406011	4.354113051	0.037277647	0.213487685
CHAMP1	1.924229145	4.561561638	0.038518842	0.217956466
EPB41L4B	1.923330881	4.850909959	0.033832826	0.201879797
MRGBP	1.921944382	5.094823634	0.03311162	0.198737399
POLA1	1.921819983	4.506855378	0.039440343	0.221023985
LOC286189	1.919823447	3.974303506	0.043639988	0.234031684
FOSL1	1.918358979	3.485745347	0.048605977	0.249912809
RP11-143K11.1	1.918315308	3.580517347	0.049511261	0.252687059
FAM120C	1.918233658	4.436512308	0.040458543	0.224959076
TMEM234	1.917449353	4.098251433	0.045187007	0.23881511
PDK4	1.916484058	3.713635426	0.043682014	0.234031684
HERC2P7	1.914567489	5.639741523	0.03150472	0.19253613
FER1L4	1.912688167	4.502309498	0.039440343	0.221023985
NCBP2	1.911915879	4.676422325	0.036916356	0.211676601
C19orf52	1.911568872	4.673987867	0.038307341	0.217805768
MAP3K7	1.910790236	3.875014641	0.045516199	0.240143095

ATP6V0E2	1.909573155	5.477813232	0.034332167	0.203566699
QTRT1	1.908845588	4.298088074	0.044916029	0.238017935
SORD	1.907498266	6.307477778	0.030422857	0.188325098
SEMA4C	1.907263758	3.877982992	0.045516199	0.240143095
GHRL	1.907040779	4.583351489	0.037023053	0.212184819
KCNIP1	1.906696523	3.59019827	0.049511261	0.252687059
PDCD7	1.906006999	4.129924883	0.040604915	0.225325984
LOC100130232	1.905951186	4.766193797	0.038120537	0.217413852
SLC25A10	1.905525234	6.183516374	0.030662679	0.18960981
RPRD2	1.903276766	4.191336153	0.041406814	0.227044057
SLC29A3	1.903084696	4.91063364	0.036118839	0.209737603
ACPP	1.901836744	5.183231141	0.035512549	0.207696708
SLC30A9	1.900656566	4.094161252	0.045187007	0.23881511
DCP1A	1.900391755	4.193156422	0.041406814	0.227044057
KCND2	1.899964438	3.973385713	0.043639988	0.234031684
HIST1H2BK	1.899707519	5.874145427	0.032030963	0.194890282
PLXNA2	1.898975227	3.939790321	0.046308998	0.241746369
WIBG	1.898323032	4.247456835	0.042153843	0.228437848
XPNPEP1	1.897583516	4.326429864	0.040894132	0.226043517
DGKI	1.897507193	4.30064761	0.044916029	0.238017935
ZNF415	1.893981246	4.101906274	0.045187007	0.23881511
CYB561D2	1.893206744	5.28066052	0.035936042	0.209203458
HNRNPD	1.891656171	4.184308606	0.04357416	0.234031684
NAT8L	1.89093791	4.728037146	0.037532334	0.21489388
HBZ	1.888266738	5.317297298	0.035491252	0.207696708
ZG16B	1.886493813	4.767404736	0.038120537	0.217413852
CNDP2	1.885211092	5.608671095	0.034751209	0.205039552
SNHG7 /// SNORA17 /// SNORA43	1.884884414	4.382666947	0.041589141	0.227810645
SSTR5-AS1	1.883201936	4.355046684	0.045520058	0.240143095
RPA2	1.881087262	5.063240032	0.037076898	0.212441581
GRHL3	1.880353972	4.480091477	0.04285701	0.231341196
FDFT1	1.879807155	6.762568273	0.031204856	0.19167856
LINC00421	1.876247294	4.584938636	0.042302593	0.228821669
LOC101928921	1.875626597	4.461808464	0.04469104	0.237724963
TROAP	1.874966452	4.478431615	0.04285701	0.231341196
ACSS2	1.874691519	5.468075152	0.035854817	0.209203458
HIST1H4J	1.874452291	5.440555034	0.035471678	0.207696708
TBC1D10B	1.874085381	4.88833651	0.039735562	0.221920419
VPS37C	1.870066291	5.901084542	0.033714872	0.201278216
TP53BP2	1.867487329	4.272379407	0.047094745	0.244389167
NOC3L	1.866842071	4.65701576	0.041272511	0.227044057
LOC102724312	1.866035452	4.761750247	0.039472949	0.221153949

CDH22	1.866009319	4.647787056	0.041272511	0.227044057
LOC254896 /// TNFRSF10C	1.864378814	4.141873377	0.048308159	0.24947357
C11orf31	1.863170048	5.711527407	0.034781313	0.205039552
FNBP1L	1.86175022	5.79866402	0.035415597	0.207568856
AQP12A /// AQP12B	1.860866495	5.007978312	0.039955752	0.223060964
C16orf62	1.85966926	4.811637474	0.043317939	0.233159586
P2RX5-TAX1BP3 /// TAX1BP3	1.859562314	5.930666613	0.035396463	0.207568856
MARCKSL1	1.8591274	6.674759956	0.033977757	0.202384751
HNRNPUL2	1.856789993	4.998127436	0.041156722	0.227044057
WHAMMP2	1.855798019	5.655325318	0.036122865	0.209737603
LOC554206	1.850859101	4.314587095	0.04987958	0.25373995
MRPS24 /// URGCP-MRPS24	1.850480473	8.137103929	0.032175166	0.195096667
LOC102723722 /// NDUFA6-AS1	1.848763372	5.733088858	0.038066658	0.217212033
SLC25A24	1.844519361	4.285449983	0.047094745	0.244389167
LINC00341 /// SYNE3	1.843282986	4.319589566	0.04987958	0.25373995
MOB2	1.841786784	5.222516689	0.039287177	0.220323313
NASP	1.841322372	4.583076097	0.043984224	0.23542433
KLHL2	1.841054456	4.530829422	0.043436716	0.233611571
COX14	1.840667288	6.255848235	0.036258676	0.210422141
COX7A2L	1.839854241	7.144767562	0.034656397	0.205039552
SLC6A12	1.839630373	5.070071084	0.040842962	0.225813808
MTMR9LP	1.838937778	4.5745869	0.045742414	0.240968635
DNAJC16	1.835811343	4.480055261	0.04469104	0.237724963
ADPRHL1	1.834598322	4.655880468	0.046679966	0.243149836
IFT46	1.834354658	4.619431149	0.046224241	0.241525771
TCEA3	1.832720614	4.412053073	0.046085171	0.241429268
AK057657 /// RP11-473I1.9	1.83096735	5.308473906	0.04044431	0.224959076
MYO15A	1.827469668	5.366242315	0.0411559	0.227044057
DAB2IP	1.827374408	4.407202034	0.046085171	0.241429268
KPTN	1.826077635	4.528701855	0.045232199	0.239000185
NCR2	1.825314028	5.007381832	0.044084393	0.235906733
HS3ST4	1.824155959	4.452122525	0.048633759	0.249946239
RPL10A	1.823239829	9.743025903	0.033964108	0.202354763
PABPC1L2A /// PABPC1L2B	1.821620319	5.589921874	0.041074053	0.226931246

THUMPD1	1.821067155	4.898315914	0.044203848	0.236492098
LINC00629	1.820931829	4.511027987	0.047112672	0.244428199
NME1-NME2 /// NME2	1.817680187	5.279388736	0.041065989	0.226931246
STRN4	1.816752463	5.546051865	0.039642135	0.221626137
TRAF3	1.813766761	5.059828831	0.043220707	0.233090187
NOL12 /// TRIOBP	1.813649798	4.955057577	0.046389947	0.241746369
VAPA	1.813459829	5.681013035	0.041423073	0.227044057
LOC100128079	1.810711989	4.713725632	0.045458036	0.240139462
EEF2K /// LOC101930123	1.807001098	5.032417788	0.04708905	0.244389167
OR7E19P	1.806005493	4.585013592	0.049901074	0.253794332
PICK1	1.805986876	5.761463744	0.040745138	0.225325984
YWHAB	1.801486513	5.580327815	0.04189955	0.227861691
FAXDC2	1.790851344	5.923615559	0.042027023	0.227861691
RGL1	1.78809557	6.481995579	0.040380222	0.224683128
WDR82	1.785342457	5.951098878	0.043133988	0.232675922
TMEM161A	1.783900286	5.163947579	0.048324687	0.249504078
ARF4	1.773175384	6.721277772	0.042089274	0.228140567
C21orf33	1.772507646	4.872142814	0.048632027	0.249946239
TRAPPC6A	1.771957718	6.222368647	0.043025853	0.232145923
TULP3	1.771722612	5.30296372	0.047060118	0.244371416
SPCS2	1.767730307	5.290333606	0.048205315	0.249051961
ARPC1B	1.765608076	6.011277339	0.045940781	0.241332921
COX7C	1.763702831	7.508929114	0.041795407	0.227861691
EIF4A3	1.762422608	6.349630274	0.044512082	0.237724963
LOC101929441	1.760085949	5.40704636	0.048102555	0.248630414
BC047484	1.755216578	5.400937293	0.049186182	0.251742564
WDR83OS	1.732991685	5.829973915	0.049799809	0.253571923
AKR1A1	1.731072596	5.774217485	0.049290662	0.252054034
TRIM46	1.72234719	5.745303293	0.049985922	0.254115837
TMEFF2	1.708697938	8.010092435	0.046943708	0.243928687
RNPS1	1.701730831	6.512308286	0.049934783	0.253910807
ATP5G1	1.70055937	7.308044455	0.048946398	0.251442986
UQCRQ	1.690152865	9.260977736	0.048661403	0.250033613
Y16709	1.685822954	8.330792999	0.049702324	0.253571923

2.2 Supplementary Table 2

Table S2. The 1602 downregulated DEGs in GSE22331.

Gene ID	logFC	logCPM	PValue	FDR

SUZ12 /// SUZ12P1	-1.701072951	8.063814125	0.048137117	0.248754324
RNF148	-1.7137292	8.228100732	0.046803413	0.243522874
MT1F	-1.734819435	8.16028152	0.043977596	0.23542433
LOC101929681	-1.735583825	6.572190154	0.047151886	0.244577633
DDX20	-1.736008344	7.506969859	0.044632939	0.237724963
S100A10	-1.741210987	6.569498253	0.046080037	0.241429268
RIOK3	-1.749430497	6.713002493	0.044350747	0.237169986
TPP2	-1.750050624	5.999161004	0.046268451	0.241703014
PLEKHA1	-1.76135546	6.761911275	0.043632576	0.234031684
LOC100505685	-1.764916924	8.520979089	0.040249781	0.22401039
BBIP1	-1.76681984	5.300502938	0.047937952	0.247834147
LOC283214	-1.777386653	5.271843457	0.047660759	0.246823148
SARAF	-1.777832047	9.304212533	0.03859871	0.218076213
KLHL7	-1.780467802	6.680036287	0.042207446	0.228675584
LINC00943	-1.788484854	8.455765276	0.037932307	0.216708599
NUP210L	-1.791077016	5.41659617	0.04785492	0.247459339
SCG5	-1.794877713	5.839659283	0.042954752	0.231815537
NDUFA9	-1.800166657	9.785202941	0.036203747	0.210155281
PTPMT1	-1.802724903	7.2863104	0.037646202	0.215335907
GSPT2	-1.805200316	8.263629513	0.036511397	0.211314202
TPD52L3	-1.808788805	6.545455154	0.039596272	0.221422431
MRPS18B	-1.809474013	4.994776502	0.047814044	0.247302407
RIBC1	-1.809934002	7.15605545	0.037124575	0.21266289
ARMC5	-1.814095506	5.11641579	0.046106663	0.241429268
RP11-90C4.2	-1.816128951	8.756814436	0.035218216	0.206824731
CUL3	-1.819677641	8.517440278	0.034949856	0.205620337
TUBG1	-1.822507652	5.011925918	0.046389947	0.241746369
FAM230B /// FAM230C /// LOC729461	-1.823079421	8.537638491	0.034536579	0.204623785
PIGP	-1.825458567	5.164560078	0.043979911	0.23542433
STOML2	-1.83357717	6.679695951	0.035646301	0.208102112
PPP1R10	-1.833768314	5.030094788	0.043684188	0.234031684
EVA1C	-1.833970939	8.260388218	0.033803017	0.201753168
TMOD1 /// TSTD2	-1.844548308	5.291478141	0.040691475	0.225325984
ZDHHC13	-1.848517406	8.032982137	0.032943767	0.197780471
NIM1K	-1.848769281	4.387140035	0.04987958	0.25373995
LOC102723847	-1.849297843	9.22942772	0.031827546	0.194188444
LOC101928035	-1.849479639	5.291256195	0.040691475	0.225325984
LOC102723742	-1.857871618	5.844930435	0.03633325	0.210802842
FOPNL	-1.858679041	6.583256207	0.034596245	0.204822323
CRISP2	-1.863018293	12.82557171	0.030206265	0.187726342
FAM229B	-1.864125785	9.500754995	0.030572461	0.1891017

PSMC3IP	-1.86520135	4.638814111	0.043984224	0.23542433
CARKD	-1.866006053	5.660959978	0.036493571	0.211314202
DPCD	-1.872517894	6.737714042	0.03291812	0.197678977
TEX29	-1.87352375	4.93588979	0.039735562	0.221920419
DPH3	-1.873779838	5.604603269	0.036540557	0.211314202
TBX5-AS1	-1.875579014	6.113626304	0.033161012	0.198983011
AA06	-1.875922481	4.361700161	0.044916029	0.238017935
GALNT10	-1.879048465	4.015221874	0.049165447	0.251742564
PCNA	-1.879939826	6.594041978	0.032013742	0.194835968
PPIL1	-1.880666399	5.027306204	0.038309784	0.217805768
IL13RA2	-1.880982015	6.159138624	0.033928785	0.20219559
GCNT7	-1.881281754	4.310101886	0.044268966	0.236786555
LOC113230 /// MIR1199	-1.881545746	4.373750723	0.044916029	0.238017935
TPPP2	-1.884171628	7.569343249	0.030465292	0.188538101
SPOCK2	-1.888005216	4.42446537	0.045520058	0.240143095
CCNA1	-1.889396482	6.488244629	0.031425517	0.192152065
C16orf80	-1.889599685	7.65962742	0.02950943	0.184371149
CTD-2118P12.1	-1.890730429	4.852094229	0.040016131	0.223344965
CXCL8	-1.891208804	6.390910216	0.032325232	0.19526468
KNTC1	-1.891429354	4.449435295	0.041589141	0.227810645
GOLGA6L2	-1.896755268	10.24259416	0.027698006	0.176002583
WFIKKN1	-1.899583407	4.214434781	0.048308159	0.24947357
PRR9	-1.903439837	7.14191478	0.029284256	0.183305553
ASB9	-1.903862893	4.286116925	0.046505816	0.242296433
LINC01095	-1.904221566	7.371237581	0.028809608	0.181446466
LOC102723661	-1.907156906	8.320727626	0.027735429	0.176192726
TCEB3B	-1.908677392	6.244026795	0.03076257	0.190027424
DDX3Y	-1.916078902	8.553817583	0.026822732	0.173491083
DNAJB4	-1.921915667	4.685415479	0.037681099	0.21548305
CAMK1	-1.922618044	4.205411779	0.040604915	0.225325984
AK093205	-1.922796442	5.859244918	0.031938279	0.194435206
SH3PXD2B	-1.927925449	3.928920658	0.048474468	0.249564364
GGH	-1.928769746	5.159358088	0.035523811	0.207696708
BOLA2 /// LOC101060386 /// LOC101060596 /// LOC101060604 /// LOC102723773 /// LOC613037 /// NPIPA5 /// NPIPB4 /// NPIPB5 /// SLC7A5P1 ///	-1.930281967	4.099804299	0.047033155	0.244285401

SMG1 /// SMG1P1 /// SMG1P2 /// SMG1P3 /// SMG1P5 /// SMG1P7				
CCDC110	-1.933085335	4.195409085	0.042826199	0.231334346
UBE2N	-1.934385423	8.806116497	0.025337944	0.168950169
LINC00326	-1.937684412	7.965455562	0.025667705	0.170190722
PSG3	-1.937759511	4.171569058	0.045187007	0.23881511
POLD1	-1.939059779	3.853255235	0.047710877	0.246823148
FAM188B /// INMT /// INMT-FAM188B	-1.942150855	4.22921122	0.04587169	0.241252271
KB-431C1.4	-1.943516114	3.8566259	0.047710877	0.246823148
GRID1-AS1	-1.944117976	6.457892074	0.027051644	0.174025207
WDR66	-1.944526307	5.430147877	0.031209881	0.19167856
ZZEF1	-1.944751401	5.548135175	0.030838056	0.190443642
RYR3	-1.946905329	4.159974514	0.045187007	0.23881511
OR7D2	-1.947724924	4.368880314	0.042851358	0.231341196
NOXA1	-1.948112171	4.054172394	0.043639988	0.234031684
LOC101927424 /// LOC101929800 /// LOC440896	-1.951435611	7.08770471	0.025288932	0.168774885
LOC102725116	-1.952289199	6.479258479	0.02637151	0.172712434
KDELR1	-1.954215619	4.50489459	0.040458543	0.224959076
OR1E1	-1.954367242	3.58457282	0.048605977	0.249912809
ATXN3L	-1.95440082	6.693053185	0.025973651	0.171060559
ECM1	-1.955833733	3.681022833	0.049511261	0.252687059
UBE2Q2L	-1.958853815	3.573780036	0.048605977	0.249912809
IVL	-1.961089931	3.798833254	0.043682014	0.234031684
LINC00658	-1.961513008	8.280461499	0.023676233	0.160242599
LOC101928694	-1.965931715	3.688638924	0.045915158	0.241252271
ACVR2B-AS1	-1.969346436	4.008548244	0.046308998	0.241746369
SHROOM2	-1.969968873	3.876677985	0.044644611	0.237724963
GGPS1	-1.972507985	7.199194009	0.023936147	0.161722164
CKMT2-AS1	-1.972796891	3.890340305	0.041800833	0.227861691
SLC22A11	-1.973595936	4.217293985	0.038514034	0.217956466
LOC100505851	-1.975577218	3.863489085	0.047710877	0.246823148
DNAI2	-1.975781899	4.196007049	0.040604915	0.225325984
FLYWCH2	-1.977277909	4.350611982	0.038259949	0.217787043
RCAN2	-1.980056791	4.495030231	0.03483308	0.205168327
CCT2	-1.982855113	8.980297722	0.022065781	0.155203144
RP11-843B15.2	-1.985207227	3.841233511	0.047710877	0.246823148
PPP1R36	-1.98590999	8.423446518	0.022054069	0.155203144

MSRB1	-1.986436328	3.768553456	0.04686269	0.243561572
CST4	-1.987344156	4.307579373	0.035607033	0.207924536
MGC20647	-1.987463385	3.566532748	0.048605977	0.249912809
FEV	-1.987982499	4.043681054	0.043639988	0.234031684
DDX4	-1.988236748	9.010042685	0.021694853	0.153141671
SNX15	-1.988436625	4.684806969	0.03166765	0.193280443
C14orf119	-1.990705172	4.352608338	0.038259949	0.217787043
DNM1P41	-1.991706881	3.668015144	0.049511261	0.252687059
RAB4B	-1.9919336	4.267361398	0.039361495	0.220687408
RP11-774O3.1 /// RP11-774O3.2	-1.992017933	3.612928654	0.044849939	0.238017935
TMEM171	-1.992314392	3.961423896	0.042761799	0.231039603
PPIL3	-1.992392829	3.858604193	0.047710877	0.246823148
MAFG	-1.995227078	5.233885691	0.027821102	0.176689191
RP11-498E2.7	-1.995561241	3.941676223	0.045516199	0.240143095
SACS	-1.999727715	4.991166727	0.028437202	0.179679062
RP11-421F16.3	-2.000090856	3.95629184	0.042761799	0.231039603
STAC2	-2.007304956	3.693331797	0.045915158	0.241252271
TRAV8-3	-2.007444823	3.61040916	0.044849939	0.238017935
OR2N1P /// OR2N1P	-2.013182334	4.153487748	0.037604801	0.215151483
CYB561D1	-2.014480225	4.412181111	0.031640769	0.193166566
RSAD1	-2.015125254	3.911080069	0.039161722	0.219672197
AMZ2	-2.016541397	8.098052442	0.020418406	0.146813978
CD101	-2.017768728	3.986391153	0.040195758	0.223762737
LOC728819	-2.023268836	4.131710472	0.039742006	0.221920419
CCDC59	-2.023963271	5.300964677	0.027422557	0.175152448
ZFY	-2.024259141	6.455902914	0.021916696	0.154568306
BGLT3	-2.025012937	4.345225413	0.032255894	0.195096667
LOC732360 /// TDG	-2.026532932	3.667012605	0.049511261	0.252687059
LOC100507513	-2.027572905	5.453650342	0.02553821	0.1694756
C2CD4B	-2.029029809	3.762621645	0.04686269	0.243561572
RP11-669C19.1	-2.029302953	3.215885608	0.049814168	0.253571923
ZNF629	-2.030520901	4.769517537	0.027927152	0.177266864
AF086126 /// C16orf52	-2.031045492	3.3622326	0.046377407	0.241746369
ANKRD35	-2.031071087	3.839838264	0.038031097	0.217061837
RP11-690I21.2	-2.031135914	4.34996245	0.030716392	0.18979208
DBF4	-2.034886464	4.24610637	0.034690189	0.205039552
C3AR1	-2.035156488	4.058932497	0.041144837	0.227044057
MYRFL	-2.035856481	4.15132038	0.037604801	0.215151483
TAPT1-AS1	-2.036057378	3.915475462	0.039161722	0.219672197
PCDHB12	-2.037562245	4.142718381	0.039742006	0.221920419

MAFG-AS1	-2.038697667	5.355775182	0.025298085	0.168788018
LPAR6	-2.040788145	3.771589798	0.04686269	0.243561572
CCDC169 /// CCDC169-SOHLH2	-2.04340965	7.045429946	0.020320248	0.146565329
DBIL5P	-2.043580853	8.10141233	0.019022823	0.140353061
AX747544 /// JRK	-2.044137866	3.638235862	0.041421069	0.227044057
FAM228A	-2.047628872	5.270481196	0.024376628	0.164367322
CYP2R1	-2.047707187	5.96033686	0.02250364	0.156583748
HARS2	-2.050630929	3.949813801	0.034433792	0.204066257
RP11-324L17.1	-2.050670558	3.212613621	0.049814168	0.253571923
SHC1P1 /// SHC1P1	-2.052533008	3.509962705	0.043643943	0.234031684
CYP2D6 /// CYP2D7P /// LOC101929829	-2.052813284	4.793305478	0.025963035	0.171038594
NEU4	-2.054150218	4.041559183	0.033491172	0.200095271
GNRH2	-2.054642484	3.617394039	0.041421069	0.227044057
LOC101928460	-2.05745762	3.872268432	0.035521524	0.207696708
PAFAH1B3	-2.058030044	4.563018746	0.028080278	0.17809446
PDAP1	-2.058475214	3.989895528	0.040195758	0.223762737
RP11-173B14.4	-2.058613319	4.345310061	0.030716392	0.18979208
TMEM254-AS1	-2.060418574	3.799497871	0.043682014	0.234031684
POPDC2	-2.061469262	3.707397283	0.042613565	0.230344653
METTL21A	-2.062728349	4.798948783	0.025963035	0.171038594
SYCP2	-2.062880763	3.100342883	0.048450223	0.249548921
NSUN7	-2.065877991	6.043357808	0.021068416	0.148853903
MAP3K19	-2.066557073	6.360101711	0.020199432	0.145738656
EPS8L3	-2.066668949	3.842310026	0.038031097	0.217061837
LOC101927282	-2.070754187	3.976738157	0.032316502	0.195262157
LINC00917	-2.072909442	8.406520792	0.017332015	0.13292971
MAP3K14-AS1	-2.07311708	3.743479304	0.039579885	0.221383488
SBDS /// SBDSP1	-2.07383841	4.714472808	0.028229091	0.178509016
MOV10	-2.074146105	3.210674639	0.049814168	0.253571923
LOC100506885	-2.074355194	3.797270956	0.043682014	0.234031684
TRPM2	-2.074463166	4.466874519	0.031086254	0.191122295
FZD10-AS1	-2.07484141	3.259516016	0.044985511	0.238017935
PRH1 /// PRH1-PRR4 /// PRR4	-2.078890114	4.204234386	0.031939616	0.194435206
LOC100134040	-2.081192223	10.90815917	0.016297917	0.127879317
PIGA	-2.08271887	3.985964372	0.032316502	0.195262157
RSPO4	-2.082836438	3.653382686	0.038287949	0.217787043
LINC00347	-2.085728584	6.379715734	0.018993681	0.140182075

PIBF1	-2.088227958	3.279058251	0.040683222	0.225325984
LOC100507065	-2.088377258	3.643113663	0.038287949	0.217787043
CCDC71	-2.088468425	3.932207566	0.036710999	0.211314202
PSMB7	-2.089080472	3.62161171	0.041421069	0.227044057
LOC100131581	-2.090532423	3.933799292	0.036710999	0.211314202
ELN	-2.091061206	4.009409313	0.03557298	0.207924536
CDY1 /// CDY1B /// CDY2A /// CDY2B	-2.094136293	3.477247449	0.047571201	0.246480515
ALX1	-2.094137553	4.061609646	0.031547384	0.192696604
FDX1L	-2.094849781	3.375433415	0.046377407	0.241746369
HSD3B7	-2.095173797	3.864220903	0.035521524	0.207696708
NXNL1	-2.097260577	3.74845592	0.03679012	0.211314202
CAMK1G	-2.100951408	3.457525577	0.047571201	0.246480515
DDIT4	-2.101041139	4.479520659	0.025129106	0.167946786
LINC01226	-2.103150419	3.08743391	0.048450223	0.249548921
RSPH9	-2.105023094	3.365326425	0.046377407	0.241746369
PLCD1	-2.10704917	4.156104198	0.029188121	0.182898732
RRS1	-2.109283704	3.275921271	0.040683222	0.225325984
IRAK1	-2.113459384	3.617855552	0.041421069	0.227044057
HCN3	-2.113627135	4.396890745	0.026578631	0.172890711
TUSC8	-2.114583097	3.879188021	0.033199284	0.199110946
C1orf100	-2.118664981	5.410543193	0.020850677	0.148764869
KLF3-AS1	-2.120311677	3.205448076	0.049814168	0.253571923
SCRG1	-2.120891554	3.653347566	0.038287949	0.217787043
GAS7	-2.123838198	3.835341079	0.038031097	0.217061837
TM4SF4	-2.123878714	3.145976776	0.043342751	0.233159586
PHGR1	-2.127016531	4.031617424	0.033491172	0.200095271
MIR302B	-2.130399391	3.521252097	0.040082012	0.223447227
C11orf48 /// LOC102288414	-2.132271209	4.590873432	0.024751749	0.166419032
MAGEA8	-2.132392303	3.349746598	0.046377407	0.241746369
KRBA1	-2.133448964	4.025245982	0.026804893	0.173423448
EPS8	-2.13809785	3.316756554	0.036844159	0.211314202
CHMP7	-2.139532818	4.107710716	0.02644669	0.172890711
DUSP2	-2.141850618	3.63963242	0.038287949	0.217787043
LOC100506071	-2.141850618	3.63963242	0.038287949	0.217787043
NAV1	-2.142447035	3.310567377	0.036844159	0.211314202
RP11-102C16.3	-2.14695393	4.134947781	0.024961318	0.167349109
TMEM220	-2.147363133	4.829046501	0.022490448	0.156583748
MRPL45	-2.147538828	3.917162735	0.029056273	0.18212112
TRO	-2.148859115	3.257098648	0.044985511	0.238017935
MLNR	-2.149658924	3.344325905	0.046377407	0.241746369
RP11-218F4.1	-2.151115097	3.691257904	0.032799238	0.197114275

TRDV3	-2.152867116	3.219846854	0.049814168	0.253571923
PLXNB2	-2.153306225	3.419862522	0.038568237	0.217956466
IHH	-2.153484583	3.96459441	0.032316502	0.195262157
LOC100996782 /// MED18	-2.153714772	3.489356842	0.043643943	0.234031684
ZNF17	-2.153888959	3.852072107	0.035521524	0.207696708
OR2F1	-2.160793371	3.333999001	0.036844159	0.211314202
LRRC14	-2.162078501	3.770917293	0.034222603	0.203019542
RPL7L1 /// WAC-AS1	-2.162129132	5.520418531	0.017799779	0.135149453
HEG1	-2.162551192	3.175114179	0.038840397	0.218652915
LOC100505812	-2.163012984	3.470922856	0.035233806	0.206824731
ABO	-2.16454677	3.437285503	0.038568237	0.217956466
IQCF3 /// IQCF4	-2.165970425	8.083407028	0.013184216	0.11291418
POLE2	-2.166101484	3.198857138	0.049814168	0.253571923
AQP1	-2.166150751	3.596702895	0.031233847	0.19167856
OTUD3	-2.166737092	3.4477448	0.035233806	0.206824731
HLCS	-2.166909562	3.573460305	0.033908436	0.202125599
HERC2P5 /// HERC2P5 /// HERC2P8 /// HERC2P8	-2.168871527	3.582956059	0.033908436	0.202125599
KIF24	-2.169710235	4.007039893	0.028512962	0.179915793
GYPA	-2.170202685	3.226826439	0.049814168	0.253571923
DDRGK1	-2.174833923	5.442479153	0.016803902	0.12976899
GSTA3	-2.176019808	3.963935253	0.025494123	0.169230839
AC005785.2	-2.176505878	3.725393119	0.03039573	0.188206768
PPRC1	-2.176505878	3.725393119	0.03039573	0.188206768
SNHG17 /// SNORA71B	-2.176557827	5.645421117	0.016188398	0.127354276
SLC25A46	-2.176651559	6.578163436	0.014018419	0.11719598
PGR	-2.176802991	5.288798716	0.017339702	0.13294526
ZNF219	-2.179464514	4.238092325	0.022198381	0.155835874
FKBP1B /// MFSD2B	-2.182722175	3.786906567	0.03185775	0.194188444
LOC101926943	-2.183126218	3.252973392	0.044985511	0.238017935
TTLL9	-2.183480969	5.043547789	0.017875883	0.135508306
LOC284219	-2.183569598	4.052838587	0.025212922	0.168363261
FAM230B	-2.18457218	3.17038	0.038840397	0.218652915
ACTR10	-2.18483031	2.936451249	0.046781564	0.243463117
TRIP11	-2.184890064	3.34286169	0.033413362	0.199731986
ZMYND19	-2.18496236	4.132350905	0.024961318	0.167349109
TRAPPC12	-2.186272905	4.792951587	0.018747226	0.138930545
CD276	-2.18703312	4.148432178	0.023570697	0.159574296

KLRB1	-2.189939737	3.258153215	0.044985511	0.238017935
LOC102723809	-2.190593776	3.847538667	0.027666888	0.175852415
BRMS1	-2.19198024	3.449450097	0.035233806	0.206824731
C6orf10	-2.192499208	4.263383808	0.025839294	0.170343605
PPEF1	-2.19341678	7.068122723	0.012996651	0.111348408
LOC101928789 /// SP140	-2.194037115	3.486087678	0.032224685	0.195096667
USP18	-2.198386983	4.164515136	0.022268105	0.155835874
SLC7A3	-2.201516028	3.161479808	0.038840397	0.218652915
LOC285766	-2.208914682	3.004599409	0.041376294	0.227044057
CKAP2 /// IGLC1 /// IGLV3-1 /// IGLV3-1 /// IGLV@	-2.209400373	3.100113509	0.048450223	0.249548921
DOPEY2	-2.209799897	3.676908374	0.032799238	0.197114275
CLIP4	-2.212216689	5.774043541	0.013808847	0.116021972
CTD-2287O16.5	-2.213323312	3.383558709	0.04226773	0.228685747
NHEJ1 /// SLC23A3	-2.215145799	4.4415713	0.022016178	0.154990725
CXorf51A /// CXorf51B	-2.215417217	7.914555016	0.011470433	0.103480572
EIF3C	-2.216129057	2.985188207	0.041376294	0.227044057
SWT1	-2.217116155	5.751436424	0.014902337	0.121684286
LINC01001 /// LINC01347 /// LOC101060494 /// LOC101926894 /// LOC101929038 /// LOC101929819 /// LOC101930127 /// LOC101930567 /// LOC388572	-2.218060956	4.370664437	0.022072778	0.155203144
RIPPLY3	-2.220208524	3.281222196	0.040683222	0.225325984
RBM4	-2.2205265	3.923395719	0.02720858	0.174361002
TAP1	-2.221509561	4.582634971	0.0210022	0.148764869
STOML3	-2.22346657	3.761828689	0.026168335	0.172042207
AC003989.4	-2.223818446	3.702962288	0.03039573	0.188206768
LOC645188	-2.22384443	3.0878453	0.048450223	0.249548921
TMOD4 /// VPS72	-2.224229928	3.304487364	0.036844159	0.211314202
TTTY1 /// TTTY1B	-2.224378606	4.037938518	0.025212922	0.168363261
FMO1	-2.224381357	3.40142968	0.038568237	0.217956466
AF075036 /// OR7D2	-2.225513491	5.075247916	0.015797819	0.125889542

LRRC37A4P	-2.225804547	3.834253834	0.027666888	0.175852415
SSX2IP	-2.226441382	5.236459114	0.015820975	0.126031318
RHOXF1	-2.226618289	4.109453144	0.02644669	0.172890711
NDC80	-2.227494087	3.306984326	0.036844159	0.211314202
RP11-549J18.1	-2.227589434	3.203039727	0.034864293	0.205168327
CLIP1-AS1	-2.229109733	3.460116568	0.035233806	0.206824731
LOC100128108	-2.229529758	3.737284432	0.028191532	0.178509016
HOXC13	-2.229547316	3.267866665	0.040683222	0.225325984
LINC00269	-2.230158622	3.288826041	0.040683222	0.225325984
NPHS1	-2.23205888	3.906388719	0.02250908	0.156583748
LOC100288675	-2.232902571	3.270430411	0.040683222	0.225325984
RP11-506N2.1	-2.235456274	2.56137606	0.049219036	0.251742564
LANCL3	-2.235629151	2.948781609	0.046781564	0.243463117
ETNPPL	-2.236225758	3.518590776	0.029505815	0.184371149
ACOT9	-2.237451513	3.570606561	0.033908436	0.202125599
SCMH1	-2.239526589	4.142127763	0.023570697	0.159574296
ABHD8	-2.24136917	4.3118965	0.022133394	0.155536253
LOC79999	-2.247249577	3.34204197	0.033413362	0.199731986
NLGN3	-2.247249577	3.34204197	0.033413362	0.199731986
SLC3A2	-2.248657887	3.781464029	0.024309658	0.163962815
NAPSA	-2.249068868	4.61265788	0.018517835	0.137623322
ENC1	-2.24977481	5.080263239	0.015322563	0.122894386
MZT2B	-2.250635342	5.520733081	0.014067589	0.117459486
COL5A3	-2.252871867	3.285712619	0.040683222	0.225325984
C7orf33	-2.255695058	2.82721623	0.044697393	0.237724963
CYP11B2	-2.256766887	4.509870871	0.017521375	0.13346697
RHOH	-2.257392233	3.23654608	0.031346675	0.191719888
LOC100287877	-2.25747716	3.759067635	0.026168335	0.172042207
PAQR3	-2.257663672	8.546117374	0.009918677	0.096245167
RP11-292D4.3	-2.257820168	3.065962136	0.048450223	0.249548921
SPTBN2	-2.258251259	3.859619915	0.025810337	0.170343605
LOC284080	-2.258293138	3.350527421	0.033413362	0.199731986
ZNF766	-2.261419117	5.52517726	0.013755298	0.115613403
KLHL35	-2.262444036	3.844403199	0.025810337	0.170343605
LOC100996902	-2.262772471	4.686715693	0.017873842	0.135508306
ST7-AS1	-2.263000336	3.354147979	0.033413362	0.199731986
RP13-436F16.1	-2.263606633	4.149915213	0.022268105	0.155835874
UBASH3B	-2.264152699	2.827784736	0.044697393	0.237724963
CFHR2	-2.265721895	3.119626872	0.043342751	0.233159586
RP11-435O5.5	-2.266767417	3.85243948	0.025810337	0.170343605
HSD17B7	-2.270560503	4.109622446	0.019841152	0.144127499
SLC7A2	-2.270738581	4.311835515	0.017060924	0.1314945
ORC2	-2.271099982	3.225411131	0.031346675	0.191719888
NOP10	-2.271620078	4.365355416	0.018101124	0.136335879

AX746710	-2.271915594	3.511083986	0.029505815	0.184371149
GPRC6A	-2.273497084	3.05339794	0.036672434	0.211314202
KHDRBS3	-2.273534651	4.889869354	0.015224711	0.122275726
AHCTF1 /// AHCTF1P1	-2.279463441	3.603217387	0.031233847	0.19167856
NMI	-2.281370338	3.059376673	0.032569892	0.196087109
RPRM	-2.28231692	3.308320047	0.036844159	0.211314202
IAH1	-2.284957407	6.985343854	0.009930243	0.096257688
BRDT	-2.285624482	3.037696222	0.036672434	0.211314202
RP11-127B20.2	-2.285971309	3.012593718	0.036672434	0.211314202
ATP8B2	-2.286858975	3.540365137	0.027046234	0.174025207
KLHL30-AS1	-2.28833402	3.989490189	0.02242318	0.156300628
LBR	-2.288702565	4.309191003	0.017060924	0.1314945
CARD18	-2.28924043	4.464316178	0.016587537	0.128859999
SH3BGRL2	-2.292380928	3.295225357	0.036844159	0.211314202
TOR2A	-2.293938443	3.66385232	0.024549514	0.165201143
UBBP1 /// UBBP1 /// UBBP4 /// UBBP4	-2.295202392	4.293783153	0.017968629	0.136123523
YWHAEP7	-2.296817108	3.319485024	0.036844159	0.211314202
LYZL6	-2.297134961	3.848142133	0.025810337	0.170343605
RP5-1098D14.1	-2.298129576	3.021819035	0.036672434	0.211314202
RP11-225H22.4	-2.298261983	2.830055771	0.038983062	0.218826784
BRD2	-2.298640272	8.711490263	0.00875649	0.0893286
DNAJC27-AS1	-2.304038097	2.611818907	0.042028125	0.227861691
STAG3L3	-2.306631801	4.333647861	0.016205458	0.127354276
CCL27	-2.307931409	3.992106232	0.02242318	0.156300628
LOC285181	-2.308709989	4.092470627	0.021049482	0.148764869
UTP3	-2.310774802	2.581879714	0.049219036	0.251742564
UBL7-AS1	-2.312269707	2.840580726	0.038983062	0.218826784
USP9Y	-2.313790515	3.393353992	0.027591054	0.175721803
RPL29	-2.314665465	4.762684758	0.013609077	0.114630493
PDHX	-2.314675713	4.810884199	0.013820979	0.116082392
CCDC173	-2.31470878	3.847679603	0.019579449	0.142667623
RP11-235E17.4	-2.315819731	2.87235233	0.034088492	0.202428428
CCDC58	-2.319061837	3.45569846	0.022901344	0.156863073
HAVCR1	-2.319170565	3.011948935	0.036672434	0.211314202
AOAH	-2.319741211	3.512167946	0.027046234	0.174025207
C6orf99	-2.319753329	4.227865273	0.017820961	0.135222877
AGXT2	-2.321533196	2.847552412	0.038983062	0.218826784
VWA5A	-2.323779847	3.002335096	0.041376294	0.227044057
SMC5-AS1	-2.324851256	2.879165382	0.034088492	0.202428428
ABCF3	-2.324874152	3.966199226	0.018423658	0.137516572
DOCK9-AS2	-2.325319606	4.025116573	0.019768773	0.1436906

IWS1	-2.325458493	3.5437834	0.024818572	0.166629867
PALB2	-2.327494438	2.822305893	0.038983062	0.218826784
LOC151484	-2.328871511	3.07062456	0.032569892	0.196087109
GALR2	-2.329086479	4.278691936	0.018932185	0.139772124
RP5-1068B5.3	-2.329088523	3.555555161	0.024818572	0.166629867
LOC101930288 /// LOC101930531	-2.329345904	2.882559164	0.034088492	0.202428428
MORN2	-2.329378683	6.052222078	0.009625588	0.093944455
LINC00354	-2.329927388	4.295298863	0.017968629	0.136123523
SSUH2	-2.330918068	4.203323114	0.018828856	0.139228598
FUT9	-2.331215658	2.84500496	0.038983062	0.218826784
SLC25A5	-2.331479674	6.323949175	0.009536983	0.093312287
IGLV4-60 /// IGLV4-60	-2.331525103	3.097612064	0.028984011	0.18181372
RBM11	-2.332580446	2.995781221	0.041376294	0.227044057
BC041003 /// RP11-495P10.3	-2.334073996	3.12408781	0.043342751	0.233159586
LINC01364	-2.335302173	3.64682811	0.026576667	0.172890711
SH2D4A	-2.336538914	4.974004738	0.013464468	0.113769095
RPL27AP /// RPL27AP	-2.338237949	3.432018673	0.025121187	0.167946786
TFAP4	-2.33863975	3.936649167	0.019682782	0.143198484
APCS	-2.339003841	4.042331063	0.018577866	0.137849407
MYOCD	-2.339633794	3.128348977	0.043342751	0.233159586
ZFYVE28	-2.339710609	3.478090695	0.032224685	0.195096667
SNORA37	-2.340584401	3.332435647	0.033413362	0.199731986
LOC284112 /// MIR195 /// MIR497 /// MIR497HG	-2.341940408	3.473496767	0.022901344	0.156863073
LURAP1	-2.34209805	3.61821569	0.020964854	0.148764869
NLGN4X	-2.342158625	2.550911345	0.049219036	0.251742564
LINC00671	-2.342162677	3.118075119	0.028984011	0.18181372
SLC13A4	-2.34249718	4.082690621	0.016434585	0.12856519
CD2AP	-2.345565148	3.304379124	0.025461672	0.169158825
CCDC135	-2.346429274	2.979427384	0.041376294	0.227044057
FRMD7	-2.346429274	2.979427384	0.041376294	0.227044057
KDM1B	-2.346846416	2.572675199	0.049219036	0.251742564
RASGEF1B	-2.347116257	2.821962032	0.038983062	0.218826784
C1orf87	-2.348129319	3.206004198	0.031346675	0.191719888
LOC101929550	-2.348969864	4.037491769	0.018577866	0.137849407
MAK	-2.350316886	3.360859629	0.030342931	0.188127771
SERPINB3 /// SERPINB4	-2.350611379	4.348349703	0.015398876	0.123380082

MLLT1	-2.353131949	3.906473915	0.021041606	0.148764869
STARD5	-2.353654262	3.520209498	0.027046234	0.174025207
GLTSCR1	-2.353975253	4.391662911	0.016405322	0.128421803
GALNTL5	-2.354912027	10.01884219	0.007172767	0.077938314
AP1G2 /// JPH4	-2.358831419	2.988872364	0.041376294	0.227044057
RP11-740C1.2	-2.360917621	2.546217341	0.049219036	0.251742564
CDR2 /// LOC101060399	-2.360962011	4.215969396	0.017820961	0.135222877
SLC7A11	-2.361425916	2.642850506	0.042028125	0.227861691
PAXIP1OS	-2.361917866	3.545094131	0.024818572	0.166629867
RP11-439E19.10	-2.362367356	3.30658488	0.025461672	0.169158825
CRTC2	-2.363054575	4.34798385	0.015398876	0.123380082
PEAK1	-2.364112301	4.01712111	0.019768773	0.1436906
ZNF836	-2.368769027	3.333086215	0.033413362	0.199731986
NPVF	-2.368953052	3.126311332	0.025843036	0.170343605
FAM78B	-2.371101882	3.345544747	0.030342931	0.188127771
NEDD4	-2.373177074	3.726046583	0.021003933	0.148764869
MFSD5	-2.376297071	2.828683604	0.038983062	0.218826784
JUP	-2.376385497	4.449167196	0.013532169	0.114105426
ANAPC1 /// LOC730268	-2.376514586	4.374273513	0.013920106	0.116581512
BTNL2	-2.379752532	3.341619137	0.033413362	0.199731986
LINC01007	-2.380288529	3.445068526	0.022901344	0.156863073
FEZ1	-2.382146648	4.872750123	0.011121704	0.101582846
LOXHD1	-2.384290687	3.087628424	0.028984011	0.18181372
MYPN	-2.384533986	3.562820355	0.022798603	0.156863073
MBTPS2 /// YY2	-2.384977367	3.035778916	0.036672434	0.211314202
NEU2	-2.385991481	3.346471302	0.030342931	0.188127771
CCIN	-2.388442212	4.119316187	0.014570392	0.11955559
RGN	-2.388632679	2.639673321	0.042028125	0.227861691
GREB1L	-2.390918504	3.364493788	0.030342931	0.188127771
ORC3	-2.393956045	2.988222542	0.041376294	0.227044057
LA16c-381G6.1	-2.39482424	2.842705395	0.038983062	0.218826784
IL2RB	-2.395320376	3.896997535	0.015875293	0.126292715
GATC	-2.396610541	2.591272318	0.049219036	0.251742564
GSTT1	-2.396610699	3.861192129	0.018244259	0.136997112
NUMB	-2.396629302	2.962227374	0.041376294	0.227044057
PDZK1	-2.396872201	3.608689114	0.020964854	0.148764869
GPR112	-2.398557476	3.592042319	0.020964854	0.148764869
LINC00901	-2.398961465	6.70244086	0.00738708	0.079531293
OVCH1-AS1	-2.399795161	4.692912248	0.011627899	0.10466
AC018755.16	-2.401003274	3.698524421	0.022697535	0.156863073
ENPP3	-2.403065963	3.14012999	0.025843036	0.170343605
SNX14	-2.404447631	3.249543054	0.028229227	0.178509016

CTB-102L5.7	-2.40669093	2.912061439	0.029884417	0.185922864
AX746823	-2.406847919	4.031360436	0.014231827	0.117644205
RP11-536G4.2	-2.407550756	4.29813601	0.013623884	0.114672976
ZP1	-2.408040687	3.297925158	0.025461672	0.169158825
LOC730101	-2.410440897	3.507880498	0.019102998	0.140635421
U2AF1L4	-2.412009034	4.256888748	0.01514944	0.122275726
CLHC1	-2.412657203	2.584608108	0.049219036	0.251742564
CERCAM	-2.412888034	3.53851834	0.024818572	0.166629867
CHRM3-AS2	-2.413116496	2.856584264	0.038983062	0.218826784
IL10RA	-2.414210106	3.982955551	0.01617267	0.127354276
NDUFB2-AS1	-2.416025448	7.84525963	0.006254725	0.072596841
CDH16	-2.417222551	3.087001033	0.028984011	0.18181372
CEACAM8	-2.417653549	2.860031992	0.034088492	0.202428428
FPGT	-2.418107082	3.113661223	0.028984011	0.18181372
APOA2	-2.420278347	3.966889958	0.017256092	0.132477161
NGFR	-2.423335028	3.16825939	0.02308602	0.156863073
GDPD2	-2.423386554	3.976376566	0.017256092	0.132477161
DEFB4A /// DEFB4B	-2.425208994	4.555232723	0.012060365	0.106947596
RP11-1007O24.3	-2.425276155	4.459077541	0.012907207	0.111020849
FBXL16	-2.426215636	3.145433415	0.025843036	0.170343605
TACR3	-2.427164861	3.041191874	0.032569892	0.196087109
SETD6	-2.429391423	3.62536879	0.019298276	0.141673466
AKTIP	-2.429828985	7.603875367	0.006208423	0.072161502
BTN3A1	-2.431863025	3.8359558	0.019579449	0.142667623
FOS	-2.431863025	3.8359558	0.019579449	0.142667623
CLDN18	-2.432898692	2.946772145	0.029884417	0.185922864
ASB3 /// AX747197	-2.433438499	3.151021616	0.025843036	0.170343605
LINC00886	-2.433656778	2.776344475	0.044697393	0.237724963
RP11-214K3.19	-2.435584996	8.169461034	0.005903131	0.069745944
COL15A1	-2.436839612	2.935078338	0.029884417	0.185922864
OTUD6B	-2.437913202	2.993836115	0.036672434	0.211314202
ARHGEF34P /// OR2A4 /// OR2A7	-2.438482157	2.779998205	0.044697393	0.237724963
CR1L	-2.439557045	3.299851086	0.025461672	0.169158825
HTR3B	-2.441977353	2.996957053	0.036672434	0.211314202
CCDC170	-2.442117666	4.325997757	0.012272237	0.107574403
FCER2	-2.442763171	3.302352599	0.023000594	0.156863073
HNRNPA1P31 /// HNRNPA1P31	-2.442783629	3.053216016	0.032569892	0.196087109
AK021977	-2.442869973	2.361311964	0.046195495	0.241429268
RP11-123K19.2	-2.443016058	3.53350299	0.017478245	0.133224833
LINC01096	-2.444414504	4.271548153	0.011284046	0.102585446

KIAA1614	-2.446662043	3.056205564	0.032569892	0.196087109
TMEM50A	-2.446989016	2.683420337	0.036013059	0.209203458
MC1R	-2.448374453	3.537727267	0.017478245	0.133224833
FBXW4P1	-2.448723614	2.852447426	0.038983062	0.218826784
LOC101929910 /// LOC613037 /// NPIPA5 /// NPIPB11 /// NPIPB3 /// NPIPB4 /// NPIPB5 /// NPIPB8	-2.449464453	6.464696492	0.006543914	0.074337343
CCDC87	-2.450246838	3.754255257	0.018033255	0.136264188
DSCR3	-2.450419122	3.045395034	0.032569892	0.196087109
DUSP21	-2.452967484	5.527854053	0.007340382	0.07921004
LOC101927391	-2.453260667	2.855904709	0.034088492	0.202428428
CSNK1E /// CSNK1E /// LOC400927	-2.453520803	3.928768024	0.014824518	0.121217393
GAS5-AS1	-2.454101561	3.006277235	0.036672434	0.211314202
BC053951	-2.455068038	3.823054081	0.014436237	0.118662028
GUCA2A	-2.455135759	3.838892187	0.014436237	0.118662028
IGKV1-17 /// IGKV1-17	-2.455377281	4.325628071	0.012272237	0.107574403
COX7B2	-2.455944699	3.422406776	0.025121187	0.167946786
LINC01212	-2.45632297	2.371169167	0.046195495	0.241429268
EPHA6	-2.456406596	3.090749101	0.028984011	0.18181372
NPTX1	-2.456655246	4.258260664	0.011914274	0.106059156
FOLR1	-2.456842745	3.691391904	0.016400808	0.128421803
DGKK	-2.457025247	2.579653622	0.049219036	0.251742564
SRBD1	-2.458179019	3.037530813	0.032569892	0.196087109
ZSWIM2	-2.461738333	3.468573093	0.020903485	0.148764869
LOC100507291	-2.462029538	6.646451461	0.006193005	0.07202281
RETN	-2.462456219	2.730301189	0.030962986	0.190563917
CRYGEP	-2.464695881	4.168081817	0.012211177	0.107159124
DLL1	-2.46520079	3.26158534	0.028229227	0.178509016
DNAH8	-2.466411644	4.156903048	0.012945071	0.111020849
AX746670 /// RP11-171I2.2 /// TTN	-2.46655272	3.645753296	0.017781944	0.13505769
AQP9	-2.46711699	3.997330211	0.015166636	0.122275726
MCPH1	-2.468565658	3.252253037	0.028229227	0.178509016
VWC2	-2.469183734	3.153194894	0.02308602	0.156863073
ZC3H12D	-2.470320771	2.990058939	0.023136836	0.156863073

ASPDH	-2.47038922	4.068902323	0.012553876	0.109472446
POLM	-2.47054101	2.960916009	0.0262636	0.17205368
KAZALD1	-2.471560067	2.641151609	0.042028125	0.227861691
SH3BGRL	-2.471681934	3.278504122	0.025461672	0.169158825
LOC101928273	-2.472306192	3.206281473	0.020661082	0.14811417
LINC00572	-2.472530526	3.181354571	0.02308602	0.156863073
LOC101927133	-2.472850648	4.785337545	0.010198548	0.097510908
LCE3D	-2.474315036	4.363051326	0.011072497	0.101436631
CST9	-2.481310802	3.484001047	0.020903485	0.148764869
CLEC4G	-2.482412034	3.583873527	0.01468201	0.120219512
GPR111	-2.484261221	2.638215929	0.042028125	0.227861691
LOC100507537	-2.484261221	2.638215929	0.042028125	0.227861691
LOC101927900	-2.484308637	3.623106031	0.019298276	0.141673466
RP11-502N13.2	-2.484719068	5.938529606	0.006716853	0.075213009
REG1P	-2.487525044	3.141401026	0.025843036	0.170343605
ZPLD1	-2.487754035	2.602667991	0.042028125	0.227861691
FLJ43315 /// LOC101927088 /// LOC101927527 /// LOC101929518 /// LOC442028	-2.490978809	3.5518865	0.01601018	0.126496324
ANKRD29	-2.493371403	3.56357765	0.01601018	0.126496324
WDYHV1	-2.494078112	4.390014859	0.010005701	0.096452169
DENND4B	-2.49585385	3.839662349	0.013425963	0.113769095
DEFB122	-2.495945263	2.400319327	0.046195495	0.241429268
IFNL1	-2.496121263	2.950626282	0.0262636	0.17205368
HEATR4	-2.497807552	3.993429475	0.011349218	0.102663005
CPZ /// GPR78	-2.497889228	4.244080483	0.012585317	0.109472446
BTNL8	-2.49830582	3.904489872	0.015875293	0.126292715
F10	-2.49846253	4.280352853	0.01069186	0.099399091
LOC101929118	-2.499626649	3.858664141	0.013425963	0.113769095
MRPL54	-2.500948326	6.266923078	0.005668472	0.067673641
RP11-365H22.2	-2.501886886	3.458589805	0.020903485	0.148764869
ALG11 /// UTP14C	-2.501919582	2.76022861	0.026707832	0.172890711
ALMS1-IT1	-2.504179037	2.535281514	0.049219036	0.251742564
NR3C2	-2.50471027	3.460819849	0.020903485	0.148764869
OR7A17	-2.508097573	3.800398379	0.015534855	0.123925577
ANGPTL7	-2.509886913	4.391946438	0.010005701	0.096452169
KCNJ11	-2.512242568	2.868970421	0.034088492	0.202428428
HOXD4	-2.515045569	3.188907556	0.020661082	0.14811417
C6orf203	-2.516337488	3.240664916	0.018523963	0.137623322
RP11-65J3.14	-2.517090078	3.950002348	0.012953693	0.111020849
ADAM8	-2.517807581	3.757318314	0.012941629	0.111020849

RBSG3	-2.518136595	2.840657123	0.038983062	0.218826784
RP11-998D10.7	-2.518581349	3.267124293	0.025461672	0.169158825
TMPRSS5	-2.519037085	3.951569285	0.012953693	0.111020849
AC104537.2 /// TCEB1P28	-2.519669496	3.243274328	0.018523963	0.137623322
CTD-2561B21.11	-2.519673479	3.38535348	0.018852638	0.139228598
C17orf98	-2.520660586	4.344774037	0.011072497	0.101436631
OS9	-2.521524495	4.238854125	0.009717042	0.094601222
ZNF608	-2.523087558	2.68625196	0.036013059	0.209203458
LOC101928751	-2.526713611	3.171932863	0.02308602	0.156863073
LOC101929953 /// LRRC37A5P	-2.528351751	3.274789931	0.025461672	0.169158825
TBC1D27	-2.529451043	3.120371402	0.025843036	0.170343605
COL14A1	-2.530239474	3.473944186	0.020903485	0.148764869
DDX60	-2.530763332	2.595691813	0.042028125	0.227861691
DCUN1D1	-2.533019793	6.875666547	0.004651756	0.059747982
PDGFC	-2.533651511	3.385073281	0.018852638	0.139228598
MRM1	-2.533684249	4.355379216	0.011072497	0.101436631
CTPS2	-2.53620797	3.340911911	0.020808432	0.148626237
PPM1N	-2.536884361	3.646191357	0.017781944	0.13505769
MIRLET7BHG	-2.537405163	2.982478632	0.023136836	0.156863073
RP11-342L8.2	-2.538055636	2.475896105	0.03224147	0.195096667
LINC01431	-2.540751187	2.857995802	0.034088492	0.202428428
ATPAF2	-2.54126042	3.551881555	0.01601018	0.126496324
OR14J1	-2.542539712	2.755821611	0.026707832	0.172890711
ZNF350	-2.542979602	3.651056297	0.017781944	0.13505769
CACNA2D3	-2.543413189	5.391581575	0.005750748	0.068576936
ZIC4	-2.543825222	2.389887948	0.046195495	0.241429268
RP11-333O1.1	-2.54441572	4.352664864	0.008654935	0.088631966
RARRES3	-2.545031197	3.957379661	0.012953693	0.111020849
RND3	-2.547076073	4.829908928	0.007009753	0.076950058
ALDH1L2	-2.547325065	2.660456136	0.036013059	0.209203458
PCDH19	-2.550876576	3.931313251	0.013852865	0.116184043
CCDC3	-2.551455148	4.093351638	0.011099404	0.101436631
LOC727944	-2.551563679	3.619593558	0.013478912	0.113769095
TET2	-2.553410172	2.442905953	0.03850259	0.217956466
OXA1L	-2.554065117	4.60335319	0.007835671	0.082619204
LOC100506585	-2.5541571	4.954362644	0.006837595	0.07551894
PKN2	-2.555603547	3.781838184	0.011101378	0.101436631
DPEP1	-2.558336564	3.857284757	0.012496116	0.10929217
ASPN	-2.562292642	3.118326335	0.025843036	0.170343605
LOC101929683	-2.563294182	4.34485855	0.008654935	0.088631966
CDHR1	-2.565250774	2.496212023	0.03224147	0.195096667
LOC100506725	-2.565616688	3.004340843	0.023136836	0.156863073

SNHG1 /// SNORD22 /// SNORD25 /// SNORD26 /// SNORD27 /// SNORD28 /// SNORD29 /// SNORD30 /// SNORD31	-2.565875166	2.452191337	0.03850259	0.217956466
RP11-465I4.3	-2.56621264	3.228987393	0.018523963	0.137623322
WDR49	-2.56691953	2.582385304	0.042028125	0.227861691
AC074212.6	-2.567887346	3.230303366	0.018523963	0.137623322
RBM42	-2.568241054	2.498450739	0.03224147	0.195096667
CYP17A1	-2.56916002	4.320079309	0.009611529	0.093846234
SYCE1	-2.569357763	2.683580551	0.036013059	0.209203458
DEPDC7	-2.570552152	3.816806215	0.014436237	0.118662028
DLG3-AS1	-2.573757887	3.099032159	0.028984011	0.18181372
CHKA	-2.574203731	4.076660606	0.011800903	0.1051695
IL20	-2.575020325	3.128274784	0.025843036	0.170343605
HMGN5	-2.576183026	2.482320226	0.03224147	0.195096667
SPOPL	-2.576215977	2.934393188	0.0262636	0.17205368
STARD8	-2.576243391	3.517564126	0.017478245	0.133224833
NEUROD1	-2.576866938	3.043166793	0.020429716	0.146813978
LOC100288893	-2.577320242	3.263385138	0.016636829	0.128859999
GCKR	-2.57751084	4.552986137	0.007145155	0.077730604
GRK1	-2.577748425	2.818180013	0.02310992	0.156863073
FUNDC2	-2.579575045	9.395673489	0.003562065	0.051782199
PAQR4	-2.579935223	3.477450879	0.014138806	0.117459486
SRGAP2 /// SRGAP2B /// SRGAP2C	-2.581369518	3.786551106	0.011101378	0.101436631
MIR6878 /// TARS2	-2.581460193	4.493696402	0.008586806	0.088087882
HBG1 /// HBG2	-2.583113541	7.380364838	0.003884411	0.054412443
CLEC12B	-2.583567418	2.594953835	0.042028125	0.227861691
CCDC109B	-2.584523226	3.973990206	0.01212097	0.106947596
THUMPD2	-2.58562411	3.31990344	0.020808432	0.148626237
AC005306.3	-2.58915435	4.412096979	0.009055477	0.0893286
GBX2	-2.591726743	3.419644054	0.015540812	0.123925577
ASNA1	-2.592647463	4.181321396	0.008237392	0.084939983
LINC01181	-2.592801896	3.868503209	0.012496116	0.10929217
BAI1	-2.593529749	4.155047914	0.009266069	0.09096469
ANO5	-2.594168832	3.056654472	0.020429716	0.146813978
ZNF43	-2.596439665	3.490591518	0.012880261	0.110998929

LINC01085	-2.596846839	2.685175555	0.036013059	0.209203458
GZMM	-2.597266874	3.803741929	0.010295292	0.097684319
DRAM1	-2.597664033	3.732204553	0.012941629	0.111020849
LINC00857	-2.59942343	3.80548104	0.010295292	0.097684319
TMEM108-AS1	-2.599456674	3.119132564	0.025843036	0.170343605
NEUROG3	-2.599939646	3.734035224	0.012941629	0.111020849
LOC101927060	-2.601198286	3.972012231	0.01212097	0.106947596
RP11-305K5.1	-2.60154562	2.432782631	0.03850259	0.217956466
KIAA0196	-2.602058135	4.681189714	0.007522937	0.080367817
ACOT13	-2.602103894	4.175600567	0.008734314	0.089328038
SLC22A12	-2.603718391	3.581286369	0.01468201	0.120219512
LOC100996624	-2.604119336	6.517972871	0.003969062	0.054449116
AGTRAP	-2.604873142	3.719449326	0.013991958	0.117016402
EFNA4	-2.606895003	2.389104149	0.046195495	0.241429268
LINC00410	-2.607712255	3.79454076	0.011101378	0.101436631
SPARCL1	-2.609864857	3.098408111	0.016035504	0.126496324
NICN1	-2.611286162	5.374088718	0.005133003	0.06376086
PLGRKT	-2.611943141	3.628477535	0.012387782	0.108465809
LEO1	-2.613053936	3.525560051	0.017478245	0.133224833
SPAG17	-2.613451153	4.868213258	0.0060441	0.070746382
LOC101927129	-2.613554797	3.101298433	0.016035504	0.126496324
ZNF660	-2.613554797	3.101298433	0.016035504	0.126496324
KLHL23 /// PHOSPHO2 /// PHOSPHO2-KLHL23	-2.613938745	3.130487132	0.025843036	0.170343605
HPDL	-2.615261339	2.533778304	0.049219036	0.251742564
AFAP1L2	-2.616589405	3.044035734	0.020429716	0.146813978
TNNC1	-2.62250664	2.74323392	0.026707832	0.172890711
TBX6	-2.622789406	3.606830275	0.013478912	0.113769095
NMRK2	-2.626094515	4.097351519	0.008245364	0.084939983
GAS8	-2.628250809	4.313585509	0.007381306	0.079505562
HSBP1L1	-2.629200547	3.757615287	0.011980945	0.10657189
IRG1	-2.62984594	4.490206474	0.006771721	0.075213009
LRP6	-2.630628069	2.823391075	0.02310992	0.156863073
ZNF461	-2.631839265	3.144548963	0.02308602	0.156863073
PRKX	-2.632113973	2.860081473	0.020057392	0.144802785
UGDH	-2.63539287	3.147344055	0.02308602	0.156863073
BC089413 /// IGLVI-70 /// IGLVI-70	-2.635941715	3.744485597	0.012941629	0.111020849
RAB39A	-2.636894378	2.791742369	0.02310992	0.156863073
C8orf60	-2.637104817	3.478570622	0.012880261	0.110998929
DEPDC1B	-2.637712551	2.636030242	0.036013059	0.209203458

RP4-621B10.8	-2.637975998	3.311334386	0.014967264	0.121917808
KIF26A	-2.638152157	2.966275672	0.0262636	0.17205368
LRRTM1	-2.638484732	2.506611412	0.03224147	0.195096667
AF131215.8	-2.639698969	3.503073328	0.011748854	0.104864769
LINC01125	-2.640525466	3.387813204	0.017104955	0.131617949
SNORD114-3	-2.644895203	2.465234944	0.03224147	0.195096667
VILL	-2.645671528	3.183774451	0.020661082	0.14811417
FAM65B	-2.645807398	2.835112277	0.020057392	0.144802785
TTTY7 /// TTTY7B	-2.645913298	2.621428146	0.042028125	0.227861691
CCT6P1 /// CCT6P3	-2.646001466	3.155695206	0.02308602	0.156863073
PACRG-AS1	-2.648622476	3.809746656	0.010295292	0.097684319
XAGE3	-2.652267147	3.397168071	0.017104955	0.131617949
TNFSF15	-2.653792998	3.118203426	0.016035504	0.126496324
LOC152225	-2.656428197	2.980527134	0.023136836	0.156863073
RGL3	-2.656518997	3.953178751	0.008826286	0.0893286
LHX8	-2.658814145	2.65212005	0.036013059	0.209203458
PCYOX1L	-2.660684454	3.138326701	0.014252316	0.117644205
CTD-2534I21.8	-2.661485174	3.251304235	0.016636829	0.128859999
BC040624 /// FOXP1-AS1 /// FOXP1-AS1	-2.662286637	4.112367982	0.007749009	0.082222092
MAN2A1	-2.664677935	3.965255817	0.008826286	0.0893286
PLA2G7	-2.665065472	4.541125458	0.005617673	0.067405742
SLC26A11	-2.665864045	3.730747684	0.012941629	0.111020849
HNRNPA1P37 /// HNRNPA1P37	-2.666811127	3.408772637	0.015540812	0.123925577
MSS51	-2.667258803	3.842734794	0.008876868	0.0893286
LOC101928104	-2.667848302	3.56948014	0.01468201	0.120219512
LINC00152 /// LOC101930489 /// MIR4435-1HG	-2.66865072	3.115038955	0.016035504	0.126496324
GUCY2F	-2.67045564	3.17498515	0.020661082	0.14811417
GPR137B	-2.673915501	3.177718782	0.020661082	0.14811417
FLG2	-2.675467654	3.41568855	0.015540812	0.123925577
MPPE1	-2.675708978	4.258238352	0.00619277	0.07202281
NFXL1	-2.679964794	3.955861527	0.008826286	0.0893286
PSPN	-2.682476018	3.890209423	0.007678842	0.081662004
PGM5-AS1	-2.684434788	3.942884681	0.009448224	0.092482364
UBE2T	-2.685660178	3.583813815	0.013478912	0.113769095
TMEM169	-2.686458978	3.216024373	0.018523963	0.137623322
YPEL4	-2.691218797	4.275437365	0.007791452	0.082555619
TMX2	-2.691846615	3.248122042	0.016636829	0.128859999

ALOX15B	-2.692003221	3.379632868	0.011004551	0.10122119
CAPN7	-2.695619052	3.136305726	0.014252316	0.117644205
KIAA1586	-2.696659835	3.408217889	0.015540812	0.123925577
C9orf84	-2.701360885	3.70056284	0.009676606	0.094324829
GOLM1	-2.706253526	2.580533395	0.022910824	0.156863073
DQ588163	-2.708120487	3.315134927	0.01348744	0.113769095
DDX11-AS1	-2.711698675	3.087822111	0.016035504	0.126496324
PLCXD3	-2.712008064	2.492068223	0.03224147	0.195096667
RP11-307P5.2	-2.713364683	3.562248656	0.009812054	0.095447115
LINC00189	-2.713586437	2.469042846	0.03224147	0.195096667
PSMD6	-2.713982562	7.231006335	0.002623698	0.043412985
OSTC	-2.718970466	2.855281204	0.020057392	0.144802785
ZNF283	-2.71906914	3.093637508	0.016035504	0.126496324
LOC101929297	-2.719623309	2.473602102	0.03224147	0.195096667
LOC101928045	-2.720358756	4.005324832	0.007718521	0.082009724
HP	-2.720863281	4.474613311	0.005506886	0.066547206
FLJ44087	-2.722419166	4.899346462	0.004272068	0.057512561
NDUFA12	-2.722860773	3.244933737	0.016636829	0.128859999
CDH20	-2.722957625	3.379071802	0.011004551	0.10122119
RP6-91H8.2	-2.724407476	3.975555665	0.008251023	0.084939983
RP11-222K16.1	-2.725136958	3.549256918	0.010730295	0.099399091
PLCL1	-2.726119346	3.051448975	0.018080005	0.136264188
NDST3	-2.726514991	2.618283713	0.042028125	0.227861691
OBP2B	-2.727224355	2.745565281	0.026707832	0.172890711
PSMB10	-2.730193611	4.422391644	0.00638068	0.072829899
CD48	-2.730735243	3.359491979	0.012173415	0.106947596
FAM115C /// LOC101928422	-2.73254734	3.165534308	0.012693427	0.109710812
LOC101927244	-2.73254734	3.165534308	0.012693427	0.109710812
GLB1L2	-2.732704745	3.334770763	0.01348744	0.113769095
BC022047	-2.733620369	2.532275376	0.027120138	0.174025207
IL3RA	-2.73488483	3.137096932	0.014252316	0.117644205
ZMIZ1-AS1	-2.738018689	3.559656809	0.009812054	0.095447115
LOC284578	-2.73934579	2.536629542	0.027120138	0.174025207
BPHL	-2.74116316	4.206204159	0.007338043	0.07921004
SSX2 /// SSX2B /// SSX3	-2.741893056	3.753412434	0.008248451	0.084939983
IPO11 /// LRRC70	-2.741965888	3.142708174	0.014252316	0.117644205
LRRC56	-2.745130345	6.638295372	0.002489819	0.042380758
LOC102724888 /// LOC102725420	-2.746370306	3.176506781	0.012693427	0.109710812
SEMA4B	-2.746861094	4.598207374	0.004684563	0.06013648
ANKRD36	-2.748971722	2.367622523	0.046195495	0.241429268
OR51A10P ///	-2.750871863	3.349315262	0.012173415	0.106947596

OR51A10P				
OR8B8	-2.751525497	2.95311834	0.015240223	0.122275726
LINC00559	-2.754028366	4.300521038	0.005248701	0.064421366
IGFBP2	-2.756024583	3.153862079	0.014252316	0.117644205
LOC100132077	-2.756333478	3.326867768	0.01348744	0.113769095
LOC101929372	-2.756782204	2.808312852	0.02310992	0.156863073
PEX7	-2.757199183	4.15326862	0.006454321	0.073532932
HNRNPF	-2.7578398	4.562934363	0.005126703	0.063723024
GJD3	-2.757862628	3.328092522	0.01348744	0.113769095
WDR11	-2.757984965	4.06055555	0.00634363	0.072694901
ITGAV	-2.759024538	2.923236636	0.015240223	0.122275726
LPAL2	-2.759290746	3.482996326	0.012880261	0.110998929
GAS5	-2.75930058	2.401995637	0.03850259	0.217956466
LOC100506489	-2.760879497	2.11063319	0.041756881	0.227810645
LOC440149	-2.761011773	4.086974324	0.00558375	0.067405742
TUBGCP3	-2.763874886	2.877858238	0.017459041	0.133224833
LOC100132686	-2.764178758	2.690816619	0.030962986	0.190563917
GSX2	-2.765661832	3.09880488	0.016035504	0.126496324
CGRRF1	-2.766994405	3.851004817	0.008876868	0.0893286
DMRT3	-2.767659638	3.723967122	0.008929719	0.0893286
LOC100505920	-2.769580413	5.306390006	0.003257707	0.049598212
NOTUM	-2.76962004	3.441914591	0.009033387	0.0893286
LDHAL6A	-2.772001628	3.392603729	0.011004551	0.10122119
CELA1	-2.774330795	4.033208278	0.006767907	0.075213009
MRPS17	-2.77501666	2.822510176	0.020057392	0.144802785
CTD-2033C11.1	-2.776238656	3.041490619	0.018080005	0.136264188
LOC100293704 /// MIR8072	-2.783416703	3.6844645	0.009676606	0.094324829
LRRIQ1	-2.783787249	3.113178682	0.016035504	0.126496324
AANAT	-2.786694919	3.323841924	0.01348744	0.113769095
SCT	-2.787164521	3.178630818	0.012693427	0.109710812
PRRG2	-2.787452965	3.848862279	0.008876868	0.0893286
RGPD4-AS1	-2.787835989	4.230374725	0.004811303	0.061161862
GNPAT	-2.788376471	7.796612717	0.001924626	0.036328095
NKAPL	-2.792909562	3.314932973	0.01348744	0.113769095
LSM10	-2.793432407	4.305858364	0.005248701	0.064421366
HERC2P3 /// LOC101930458	-2.795886964	2.531524017	0.027120138	0.174025207
CSRNP2	-2.796907324	4.100686993	0.005243495	0.064421366
LOC285074 /// LOC730268	-2.797300427	3.695780437	0.009676606	0.094324829
ZNF852	-2.798374905	2.321448837	0.046195495	0.241429268
PRKG1-AS1	-2.79893009	3.059445761	0.018080005	0.136264188
LOC101927623	-2.800159858	3.654758961	0.00694101	0.0762667

SHANK2-AS3	-2.801339684	4.15158843	0.006454321	0.073532932
DQ576800	-2.80140804	4.021991787	0.006767907	0.075213009
LUZP4	-2.801687769	3.127403002	0.014252316	0.117644205
LINC00301	-2.803012678	6.070551601	0.002325647	0.040802159
LINC00884	-2.803482014	3.945171903	0.006214146	0.072161502
FKBP14	-2.80423822	2.884177721	0.017459041	0.133224833
USP44	-2.804499023	5.093737889	0.003232618	0.049354021
SNTN	-2.806335075	2.587822055	0.022910824	0.156863073
AZGP1	-2.806438979	3.254119807	0.01012814	0.096916605
RP11-305L7.3	-2.806438979	3.254119807	0.01012814	0.096916605
RASL10A	-2.80900228	3.570995639	0.008983126	0.0893286
PNMA6A	-2.809828026	3.396712509	0.009963019	0.096257688
ALOX5AP	-2.810301681	4.347274785	0.004466789	0.058678986
ERV3-1	-2.810386715	3.370284127	0.011004551	0.10122119
LINC00567	-2.813565649	3.230087389	0.01012814	0.096916605
C16orf3	-2.816496797	2.968432086	0.013339641	0.113500488
LOC101928278	-2.816992358	2.391591099	0.03850259	0.217956466
GMPR2	-2.817203668	2.596170213	0.022910824	0.156863073
FLT3	-2.818516188	2.89538427	0.017459041	0.133224833
RP11-95O2.1	-2.818601738	2.33661186	0.046195495	0.241429268
INTU	-2.819489806	3.816829777	0.006537408	0.074299367
RP1-28O17.1	-2.821913947	3.175294747	0.012693427	0.109710812
DEPDC1-AS1	-2.822043014	2.778075513	0.02310992	0.156863073
MGC57346	-2.823380364	3.237946691	0.01012814	0.096916605
HBD	-2.823893389	3.112558584	0.016035504	0.126496324
CKAP2L	-2.82400464	2.860807774	0.020057392	0.144802785
ZNF195	-2.824523364	2.974768199	0.013339641	0.113500488
RPL6	-2.825123938	4.54978159	0.00395413	0.054449116
OXSM	-2.826914323	4.780885968	0.00387635	0.054412443
PRORY	-2.829379552	4.061981629	0.005949722	0.069745944
ABCC1	-2.829717917	4.243188542	0.004543817	0.058830288
HYKK	-2.835465293	2.908711817	0.015240223	0.122275726
MAGEA4	-2.835973608	3.088856734	0.016035504	0.126496324
LOC101927709	-2.836060694	2.406002166	0.03850259	0.217956466
FW340027	-2.836480099	2.984217607	0.013339641	0.113500488
CCDC53	-2.836686797	7.748763328	0.001632779	0.033914455
UGT2B28	-2.837956707	4.272030583	0.004293343	0.057699773
BST1	-2.843251719	2.10384896	0.041756881	0.227810645
LOC101927286	-2.843871128	4.375286033	0.004020112	0.054589864
LAG3	-2.844293866	2.235965624	0.027322057	0.174558001
MOGAT1	-2.844869839	3.06178403	0.018080005	0.136264188
TRIM59	-2.847719298	2.839369792	0.020057392	0.144802785
TRIM51	-2.849709129	3.428942984	0.009033387	0.0893286
AK096335 ///	-2.849834245	2.799776947	0.02310992	0.156863073

AP000662.4				
NHP2P2 /// NHP2P2	-2.851153646	3.456523482	0.008202272	0.084939983
KRT77	-2.851925156	2.574461603	0.022910824	0.156863073
S100A6	-2.853249275	3.015572825	0.011706775	0.104767834
LY6E	-2.854200909	3.034177455	0.018080005	0.136264188
LOC101929586	-2.858293833	2.964599612	0.013339641	0.113500488
SLITRK4	-2.858460469	2.651583204	0.019434951	0.141967001
MNDA	-2.859083111	3.205036755	0.011327543	0.102585446
LOC643355	-2.860569237	3.237354665	0.01012814	0.096916605
MYRIP	-2.860569237	3.237354665	0.01012814	0.096916605
LOC101926967	-2.862289295	3.791094623	0.00705773	0.07736827
RP11-442O18.2	-2.862415119	3.207708819	0.011327543	0.102585446
LOC100131510	-2.864605341	3.639638169	0.007555632	0.080680408
LINC01272	-2.864938479	3.111938484	0.014252316	0.117644205
RP11-680C21.1	-2.866089339	3.730528616	0.008248451	0.084939983
LOC101929526	-2.868068701	2.814055143	0.020057392	0.144802785
B4GALNT2	-2.868615508	3.496633288	0.007458047	0.079735439
QRICH2	-2.868777585	2.934982914	0.015240223	0.122275726
ZBED2	-2.869129009	2.728422972	0.026707832	0.172890711
ABTB2	-2.869729383	3.348534788	0.012173415	0.106947596
SMYD4	-2.869808668	4.02665047	0.004734598	0.060415632
LGALS14	-2.870920967	2.773728279	0.02310992	0.156863073
ZCCHC13	-2.8711155	4.600242135	0.003296294	0.049999632
SLC41A2	-2.871428139	3.455417166	0.008202272	0.084939983
DYM	-2.873715147	4.22715863	0.004811303	0.061161862
RP3-400B16.4	-2.874776814	3.449270259	0.008202272	0.084939983
RP11-732A19.1	-2.876812369	3.154805119	0.012693427	0.109710812
TLR7	-2.8793493	2.82290374	0.020057392	0.144802785
MUM1L1	-2.879833337	2.904725269	0.015240223	0.122275726
ZNF595	-2.882857203	2.866981584	0.017459041	0.133224833
LOC101927348	-2.886744507	4.268403488	0.004293343	0.057699773
LOC101928111	-2.886920588	5.293853443	0.002299145	0.040411151
PANK4	-2.887167157	3.163100185	0.012693427	0.109710812
LINC00954	-2.889213806	2.951149403	0.013339641	0.113500488
SNAPIN	-2.889485271	4.193509382	0.003862936	0.054405334
ANAPC2	-2.890881641	4.25675796	0.004293343	0.057699773
KIF18B	-2.894066846	2.992955284	0.011706775	0.104767834
TH	-2.894588468	3.354266216	0.011004551	0.10122119
ZFY-AS1 /// ZFY-AS1	-2.89658563	2.917959011	0.015240223	0.122275726
CCDC26	-2.902683698	2.754621717	0.026707832	0.172890711
CDK20	-2.902903054	4.018370947	0.004734598	0.060415632
LOC100506691	-2.904570101	3.846557022	0.00562384	0.067405742

FAM124B	-2.904835135	3.473713254	0.008202272	0.084939983
RP11-68I3.11	-2.908272613	2.845645165	0.020057392	0.144802785
LMNB1	-2.908558899	3.275997691	0.009072677	0.0893286
ALDH3A1	-2.909326801	3.676289725	0.006383312	0.072829899
SCAF8	-2.91237515	4.114021323	0.004926933	0.062094158
LOC574538	-2.912585807	3.832549279	0.006060814	0.070871401
MEGF6	-2.913788032	2.622223301	0.019434951	0.141967001
SPATA31D1 /// SPATA31D3 /// SPATA31D4	-2.914747471	4.601247261	0.003296294	0.049999632
LOC100505658	-2.916462402	3.750046384	0.00499616	0.062599664
RHPN1-AS1	-2.917511605	4.184808603	0.003862936	0.054405334
RP4-612B15.3	-2.91788211	4.381916563	0.003816234	0.053876788
AMH /// MIR4321	-2.91807177	3.344061182	0.00731957	0.079094454
GXYLT1	-2.919321391	2.955634689	0.013339641	0.113500488
KIR2DS5	-2.920970398	3.4868649	0.007458047	0.079735439
LOC101928271	-2.921788234	2.525650387	0.027120138	0.174025207
SRL	-2.922036667	4.342355361	0.003256853	0.049598212
PARP16	-2.924027411	3.34888915	0.00731957	0.079094454
DKFZp451B082	-2.924623281	3.159731565	0.012693427	0.109710812
MICAL1	-2.926401584	2.727716237	0.014149938	0.117459486
CYP26A1	-2.927152571	3.321549542	0.00731957	0.079094454
GCA	-2.928629824	3.545588093	0.006191067	0.07202281
BC034444	-2.929466337	2.235149331	0.027322057	0.174558001
TMEM263	-2.931458671	4.553591458	0.00285306	0.045906903
PPP1R1B	-2.934530461	3.201159731	0.011327543	0.102585446
ZNF177 /// ZNF559-ZNF177	-2.936618076	3.026831932	0.010299849	0.097684319
RP5-1039K5.16	-2.937001188	3.267770714	0.009072677	0.0893286
LPCAT3 /// U47924.30	-2.942511739	4.878817652	0.002748344	0.04462882
PRICKLE3	-2.942717107	4.458277195	0.002959829	0.04685465
LOC101929497	-2.944176468	3.225377091	0.01012814	0.096916605
LOC101929552	-2.948415048	4.074274799	0.003889019	0.054412443
ATP5G2P1 /// ATP5G2P1	-2.94849929	3.178936275	0.011327543	0.102585446
MFAP2	-2.948840927	2.998532048	0.011706775	0.104767834
PA2G4P2 /// PA2G4P2	-2.949224746	3.42729364	0.009033387	0.0893286
DGCR9	-2.953732625	2.963292921	0.013339641	0.113500488
SLC52A1	-2.955402383	4.006616317	0.004734598	0.060415632
RPL7AP71 /// RPL7AP71	-2.958047279	3.31605096	0.00731957	0.079094454
VIT	-2.958398887	3.406092882	0.009963019	0.096257688

RP11-748H22.1	-2.962225368	3.466007331	0.008202272	0.084939983
CNGA1	-2.962290724	3.547153788	0.006191067	0.07202281
SGMS1	-2.962916494	2.502824792	0.027120138	0.174025207
AC004490.1 /// AX746733	-2.963782047	4.03234172	0.004431119	0.058645551
STX5	-2.967140289	3.323432509	0.00731957	0.079094454
ERCC6L	-2.969017747	3.355533918	0.006591493	0.074401841
LOC101929239	-2.969814503	3.654653039	0.00694101	0.0762667
BC040901 /// CTC-241N9.1 /// LOC100996419	-2.970101134	3.015502279	0.011706775	0.104767834
CYP2C18	-2.97052965	3.66740885	0.006383312	0.072829899
LOC401324	-2.970683459	3.230339239	0.01012814	0.096916605
LOC102724387	-2.970695078	2.126618435	0.041756881	0.227810645
THAP1	-2.970807627	3.386913707	0.00594568	0.069745944
SYNC	-2.973106935	2.668368319	0.016551802	0.128668079
CD7	-2.97552016	3.448755713	0.008202272	0.084939983
SAMD8	-2.976096896	3.058404239	0.010299849	0.097684319
GLP2R	-2.977160493	3.235578511	0.01012814	0.096916605
PITPNA-AS1	-2.977464211	3.967239563	0.003651944	0.051799007
RP11-692P14.1	-2.978613343	2.768672294	0.012140675	0.106947596
SLC43A2	-2.980032271	3.746131697	0.00499616	0.062599664
CYCSP19 /// RP11-3L10.1	-2.980582847	3.303082407	0.008141953	0.084939983
BOLA1	-2.980965068	3.889228291	0.004854398	0.061355847
CPN1	-2.985195127	3.274831092	0.009072677	0.0893286
MBLAC2	-2.9854678	2.906713656	0.015240223	0.122275726
LOC101926942	-2.985993572	3.785216046	0.004264421	0.057442534
PGK2	-2.987490984	9.613604341	0.000896972	0.024645223
PPIAP21 /// PPIAP21	-2.98927601	3.431291076	0.009033387	0.0893286
C1orf158	-2.989386588	3.996713493	0.005062312	0.062989321
TEP1	-2.989389079	2.345043332	0.022314819	0.155835874
ALLC	-2.989799603	4.888461969	0.002002572	0.037277678
LRRK1	-2.992240729	2.825058158	0.020057392	0.144802785
GNG13	-2.993703616	3.181053766	0.011327543	0.102585446
FXYD4	-2.994080429	3.345341959	0.006591493	0.074401841
VPREB3	-2.994829465	3.035290247	0.010299849	0.097684319
RP11-574H6.1	-2.996264414	2.872450609	0.017459041	0.133224833
RP11-554D14.1	-2.997453423	3.701704634	0.005876651	0.069745944
LOC101927346	-3.004777256	2.742048736	0.014149938	0.117459486
RP3-334F4.2	-3.004984831	3.866916584	0.005222803	0.064305083
NPIPA1 /// NPIPA5 ///	-3.006754196	4.456895743	0.002959829	0.04685465

NPIPB15 /// NPIPB3 /// NPIPB6 /// PDXDC2P				
ZNF367	-3.008055123	2.695710849	0.016551802	0.128668079
SLC9A1	-3.012957156	3.208182809	0.01012814	0.096916605
AK311120 /// LSAMP	-3.01374309	2.298558369	0.022314819	0.155835874
MYOZ1	-3.015418846	3.616897324	0.00472768	0.060415632
BC041484 /// RP11-90K6.1	-3.015475741	1.731649108	0.045799632	0.240968635
TSIX	-3.017082164	3.199959952	0.011327543	0.102585446
NGLY1	-3.017413991	7.466968013	0.000939013	0.024813611
NUS1 /// NUS1P3	-3.018968702	2.753220359	0.012140675	0.106947596
SPDYE2	-3.024337078	4.083015223	0.003646915	0.051799007
LOC101927247	-3.027837078	3.02250722	0.010299849	0.097684319
CTD-3193O13.1	-3.028829855	3.342356534	0.006591493	0.074401841
ANKS1A	-3.029971108	2.497608771	0.027120138	0.174025207
LINC01355	-3.030499109	3.359474204	0.006591493	0.074401841
BCAS2	-3.031497131	4.125837617	0.003213032	0.049354021
MGC4294	-3.033136593	4.127215829	0.003213032	0.049354021
RP1-272E8.1	-3.033552183	3.213306481	0.01012814	0.096916605
TCTE1	-3.033787352	4.198633303	0.003640076	0.051799007
RBMXL1	-3.034683069	2.379545083	0.018334706	0.136997112
AC007680.2	-3.035170225	3.708668565	0.005415811	0.065480303
KCNMB2	-3.03522551	3.848460564	0.003386477	0.050129249
DMTN	-3.035728133	4.251185674	0.003055493	0.047916984
RP1-179N16.6	-3.03942376	4.657830917	0.001986653	0.037010667
C11orf85	-3.039758944	3.736754753	0.00499616	0.062599664
RP11-472K22.1	-3.040087902	3.218608926	0.01012814	0.096916605
FAM170A	-3.041685508	2.563751623	0.022910824	0.156863073
APOD	-3.042964527	3.034653302	0.010299849	0.097684319
LOC101927798	-3.043311363	3.203678076	0.011327543	0.102585446
LOC101929529	-3.046369912	3.642471558	0.004331767	0.057983971
TCR-alpha /// TRAV13-1 /// TRAV13-1	-3.046693126	3.076962669	0.0090842	0.0893286
LOC100507221	-3.046866765	4.988219967	0.001784703	0.034880405
FAM205A	-3.047209086	4.613379359	0.002270058	0.040036192
LINC00923	-3.047455461	2.324132217	0.022314819	0.155835874
LOC100506016	-3.05095341	2.915967755	0.015240223	0.122275726
LOC101928516	-3.051335853	2.82613322	0.010453099	0.098019301
RAMP2	-3.053952333	3.082813637	0.0090842	0.0893286
RP11-314N13.3	-3.055037691	3.74944707	0.00499616	0.062599664

LOC100996634	-3.05936976	1.76249897	0.045799632	0.240968635
ERC2	-3.059662373	2.878564263	0.017459041	0.133224833
LOC101559451	-3.059733985	3.489076897	0.007458047	0.079735439
RFX7	-3.060888831	3.504495168	0.006790588	0.075213009
CYB5D2	-3.061012363	4.612084852	0.002270058	0.040036192
ZNF354C	-3.061550633	3.200700867	0.011327543	0.102585446
CECR6	-3.062165108	5.161966042	0.001561991	0.033028169
FCGR2B	-3.064912046	3.306901637	0.00731957	0.079094454
STEAP1	-3.065938658	3.239616038	0.009072677	0.0893286
MAGEA11	-3.066186839	2.403665252	0.018334706	0.136997112
RP4-614O4.12	-3.066186839	2.403665252	0.018334706	0.136997112
UFD1L	-3.066493169	5.183463864	0.001424028	0.031709257
RP11-303E16.7	-3.066657859	2.971512456	0.013339641	0.113500488
GALNT7	-3.066902929	2.691123632	0.016551802	0.128668079
M6PR /// PHC1	-3.068141766	4.192600918	0.002512059	0.042380758
MCM2	-3.071381019	3.810427189	0.003945405	0.054449116
PSMC3	-3.071499381	4.005973691	0.003181985	0.049049345
ESYT1	-3.074446991	3.765596379	0.004613585	0.05938758
KCTD11	-3.07551996	4.350044841	0.002066881	0.038262544
SKA1	-3.076884036	3.443751626	0.004860852	0.061355847
CPOX	-3.077023369	3.316794698	0.00731957	0.079094454
ALPI	-3.077168575	3.488808856	0.006790588	0.075213009
LOC100996286	-3.079888225	4.166601569	0.002669137	0.043909927
FAM98C	-3.081099418	2.537223684	0.022910824	0.156863073
TCERG1L	-3.086261013	2.98724344	0.011706775	0.104767834
DEXI	-3.08814263	3.999523224	0.003181985	0.049049345
COG7	-3.089598392	3.703965866	0.003355072	0.050071404
FPR1	-3.091698297	4.763966534	0.001810344	0.035264187
ZNF564	-3.094167895	4.084615458	0.003646915	0.051799007
DAG1	-3.09493542	4.015488041	0.002973425	0.046974924
VNN3	-3.097113044	3.07828882	0.0090842	0.0893286
SCRIB	-3.09741155	2.908697113	0.015240223	0.122275726
SLC39A11	-3.101305092	2.8183958	0.010453099	0.098019301
AX748267	-3.102847937	3.609397385	0.00472768	0.060415632
MIR6743 /// RIC8A	-3.103132954	4.975373101	0.001444353	0.031878391
GABBR1	-3.104646486	3.288545486	0.008141953	0.084939983
GNA13	-3.104894115	5.628292975	0.001140658	0.027483429
RP11-111J6.2	-3.106220949	2.36898982	0.018334706	0.136997112
GDPGP1	-3.106982606	3.27308108	0.008141953	0.084939983
TLR2	-3.107759768	4.016020193	0.002973425	0.046974924
FAS	-3.108924063	3.016037551	0.010299849	0.097684319
SIX6	-3.111683599	3.531933274	0.006191067	0.07202281
RPP38	-3.11211306	4.757226992	0.001810344	0.035264187

REEP1	-3.11252545	3.260013184	0.009072677	0.0893286
NDUFAF1	-3.114135761	4.429917852	0.00225414	0.039845192
MAGEC1	-3.114426013	3.672813814	0.003973636	0.054449116
PSD2	-3.116856378	4.179533197	0.002512059	0.042380758
KRTAP19-1	-3.118961135	2.378760868	0.018334706	0.136997112
VBP1	-3.118991551	3.173787401	0.006319303	0.072628703
TRAV34 /// TRAV34 /// V alpha immunoglobulin	-3.119317181	3.538248265	0.006191067	0.07202281
NECAB1	-3.119649809	2.567258875	0.022910824	0.156863073
GIMAP2	-3.123312539	2.682627068	0.016551802	0.128668079
XCL1 /// XCL2	-3.123366368	2.835961446	0.009030241	0.0893286
LIN28B	-3.123535249	3.51228187	0.003998158	0.054449116
EN1	-3.124786499	2.884647506	0.007826126	0.082555619
CCR6	-3.125289285	2.383620258	0.018334706	0.136997112
DPY19L1P1	-3.127217908	3.73530422	0.003087939	0.048200573
FCAMR	-3.127428085	3.656977234	0.003973636	0.054449116
LYZL1	-3.128166241	5.419119828	0.001138873	0.027468583
LINC00921 /// TIGD7 /// ZNF263	-3.128608875	3.290768363	0.008141953	0.084939983
C15orf49	-3.130573815	3.488004692	0.003998158	0.054449116
PCDHB15	-3.131482027	3.145576674	0.007116069	0.077573403
LOC729083	-3.133186961	2.891369676	0.007826126	0.082555619
JAK2	-3.138574404	3.22709016	0.005623714	0.067405742
EMC3-AS1	-3.138593705	4.000171419	0.003181985	0.049049345
HERC2 /// HERC2P10 /// LOC101929047 /// LOC101929832	-3.140251349	3.264845401	0.009072677	0.0893286
ATP8B4	-3.14173866	3.153916777	0.007116069	0.077573403
HIST1H1D	-3.14173866	3.153916777	0.007116069	0.077573403
HOMEZ	-3.142140446	2.642311495	0.016551802	0.128668079
FAM73A	-3.144966694	3.38908437	0.00594568	0.069745944
RNF139	-3.14839222	8.33059718	0.000539663	0.019150697
LOC284513	-3.150251384	2.95108226	0.013339641	0.113500488
CTXN3	-3.15270823	2.70584145	0.014149938	0.117459486
AVP	-3.154975248	3.679923646	0.003649256	0.051799007
TMEM74B	-3.156861242	4.156945716	0.002669137	0.043909927
LOC101928847	-3.158098791	2.911340107	0.007826126	0.082555619
LOC101928269	-3.162012759	3.208855079	0.005623714	0.067405742
GABRR1	-3.162285592	2.81771017	0.010453099	0.098019301
LOC101928399	-3.167002128	3.518156802	0.003633928	0.051799007
LINC01442	-3.167262139	4.311741172	0.002305564	0.040480046

AC009502.4	-3.167281807	3.456091698	0.004405204	0.058628437
AC011997.1 /// BC021693	-3.167747499	2.604928585	0.019434951	0.141967001
LOC100131860	-3.168825763	2.417159567	0.015150304	0.122275726
AC007401.2	-3.170190708	3.426272335	0.004860852	0.061355847
FAM83D	-3.170659293	3.359888348	0.006591493	0.074401841
ZBTB24	-3.171810074	3.337848855	0.006591493	0.074401841
LOC100130856 /// PSPN	-3.177921804	2.973372389	0.011706775	0.104767834
GPR150	-3.180690326	3.467185684	0.004405204	0.058628437
ZNF23	-3.182430428	2.674082346	0.016551802	0.128668079
C2orf50	-3.184481005	3.405078895	0.005371775	0.065317664
GPA33	-3.186982912	3.679428542	0.003649256	0.051799007
CPB2	-3.18859317	2.363290497	0.018334706	0.136997112
TMEM181	-3.190455906	3.626228418	0.004331767	0.057983971
NT5C1A	-3.193674736	3.37885952	0.00594568	0.069745944
LOC401220	-3.194977327	2.368203509	0.018334706	0.136997112
RP11-73M18.8	-3.195380882	4.090046919	0.002282946	0.040173011
CTD-2310F14.1	-3.197253737	2.685808321	0.014149938	0.117459486
LOC100129449	-3.197253737	2.685808321	0.014149938	0.117459486
RGS13	-3.198363387	2.648509203	0.016551802	0.128668079
C5orf34	-3.198685787	2.943989405	0.006803492	0.075213009
REG3G	-3.198989963	3.743424407	0.002845074	0.045841213
TBC1D9	-3.200532795	3.014149584	0.010299849	0.097684319
AK126278 /// CUL4A	-3.200801406	2.570754925	0.022910824	0.156863073
RP11-669M16.1	-3.201952256	3.719185105	0.003087939	0.048200573
OTOGL	-3.203798786	2.302109935	0.022314819	0.155835874
IAPP	-3.204078769	2.633524766	0.016551802	0.128668079
MIR1304 /// SNORA1 /// SNORA18 /// SNORA32 /// SNORA40 /// SNORA8 /// SNORD5 /// TAF1D	-3.20503799	3.54974688	0.003307485	0.049999632
LOC101926962	-3.207292379	3.041788036	0.0090842	0.0893286
KB-1568E2.1	-3.207661506	2.377976752	0.018334706	0.136997112
CREB5	-3.208736696	3.284577009	0.004481104	0.058678986
FAM215A	-3.208736696	3.284577009	0.004481104	0.058678986
RP11-366L5.1	-3.208736696	3.284577009	0.004481104	0.058678986
RP11-69I8.2	-3.209761562	2.751118601	0.012140675	0.106947596
HRK ///	-3.214293272	2.641586248	0.016551802	0.128668079

LOC283454				
RP11-663N22.1	-3.217158568	3.835944687	0.003386477	0.050129249
LIAS	-3.218334009	5.261370326	0.000815256	0.023074692
CSTL1	-3.218421247	3.705731953	0.003355072	0.050071404
ZBTB16	-3.220093091	3.863261209	0.003141633	0.048522849
SLC22A15	-3.221626244	3.735676017	0.003087939	0.048200573
LOC283075	-3.221822934	3.096908816	0.008030972	0.084337772
MYLK2	-3.222282563	3.33222808	0.006591493	0.074401841
TTTY7	-3.222282563	3.33222808	0.006591493	0.074401841
EGLN2 /// RAB4B /// RAB4B-EGLN2	-3.22958249	3.475458367	0.004405204	0.058628437
GUSBP1 /// GUSBP4 /// LOC100996497	-3.230922027	2.531378483	0.010510413	0.098019301
LOC145694	-3.232224882	3.477652889	0.003998158	0.054449116
PSMA7	-3.232344093	3.617560867	0.004331767	0.057983971
BTN1A1	-3.233190073	3.922736137	0.002521124	0.042380758
NCAPG2	-3.235167954	3.851117111	0.003386477	0.050129249
LINC01304	-3.238254904	5.816494756	0.000656206	0.02114621
KRTAP9-9	-3.242051001	2.979025937	0.005932006	0.069745944
RP3-497J21.1	-3.246580987	2.780490426	0.010453099	0.098019301
AK291611 /// TRGV7 /// TRGV7	-3.248318659	2.886673139	0.007826126	0.082555619
LINC01115	-3.250133915	3.459456002	0.004405204	0.058628437
RP11-131L23.2	-3.250338853	2.727962012	0.014149938	0.117459486
NRBP1	-3.250436323	6.027406237	0.000575621	0.019559727
KRT34 /// LOC100653049	-3.250626968	3.676539312	0.003649256	0.051799007
RP11-180N14.1	-3.251364977	1.900213485	0.026706022	0.172890711
HPCA	-3.251397175	4.400955505	0.001670126	0.033998787
PRR7	-3.251820415	3.588749349	0.002751	0.04462882
C1QTNF4	-3.263049168	3.36590838	0.00594568	0.069745944
RP11-1E4.1	-3.265051088	2.949771307	0.006803492	0.075213009
SLC44A3	-3.265378055	4.170106886	0.001664863	0.033998787
PRR18	-3.26864735	3.691658127	0.003355072	0.050071404
PNMAL1	-3.268747933	3.441021911	0.004860852	0.061355847
C15orf40	-3.269461663	2.425819297	0.015150304	0.122275726
SLC2A4	-3.274668313	3.338946716	0.003598289	0.051799007
CDNF	-3.277999673	3.64089875	0.003973636	0.054449116
LYSMD4	-3.279851017	3.415482928	0.004860852	0.061355847
SH3GL1P1	-3.280515336	3.343784416	0.003598289	0.051799007
LOC100289230	-3.282099737	3.188278657	0.005623714	0.067405742
VOPP1	-3.289160051	4.43342248	0.001505204	0.032500237
CCDC138	-3.2900704	3.388302001	0.005371775	0.065317664

CD1C	-3.292299338	3.494550027	0.003998158	0.054449116
GOLGA6L4 /// GOLGA6L5P /// GOLGA6L9 /// LOC102724093	-3.292430035	3.622735839	0.002513798	0.042380758
LINC01354	-3.297681132	2.517500213	0.010510413	0.098019301
OR8B2	-3.298575261	3.853548614	0.001917416	0.036328095
LOC613266	-3.301773526	3.433704496	0.004860852	0.061355847
ATP7B	-3.303442281	3.569375514	0.003014449	0.047508741
ABCB9	-3.304563352	4.41190508	0.001585188	0.033338612
LOC283454	-3.305540563	3.960009729	0.002186027	0.038849044
FHOD1	-3.307202495	3.438222459	0.004860852	0.061355847
VKORC1	-3.312461636	3.669833165	0.003649256	0.051799007
GAL3ST2	-3.318168812	3.761776117	0.002624011	0.043412985
HDX	-3.320242388	2.231885431	0.027322057	0.174558001
EXOC3	-3.321930448	3.949455449	0.002346676	0.041048479
SV2A	-3.326600585	3.094218869	0.008030972	0.084337772
RP11-799D4.4	-3.327682147	2.607585419	0.008819448	0.0893286
RP11-298D21.2	-3.329508719	2.952324685	0.005932006	0.069745944
LOC400748	-3.331038713	3.348038942	0.003598289	0.051799007
CTNNAL1	-3.335154394	4.808468666	0.000771814	0.022635483
FOXO4	-3.335649061	3.96112212	0.002186027	0.038849044
DCTN1-AS1	-3.335941307	3.190376678	0.005623714	0.067405742
SERPINA6	-3.336014333	3.055469536	0.004547753	0.058830288
LOC101929241	-3.336827087	3.352843915	0.003233264	0.049354021
HAPLN2	-3.338220929	3.14870043	0.006319303	0.072628703
AMPD1	-3.338787093	3.66167784	0.002106798	0.038575922
LINC01242	-3.339354159	3.465023389	0.004405204	0.058628437
TCEB3-AS1	-3.340519513	3.393578789	0.002910357	0.0463016
LOC441179	-3.340593648	3.940395862	0.002346676	0.041048479
THAP10	-3.342950223	4.062886671	0.001550881	0.032822782
ANKRD7	-3.343614482	5.098311177	0.000663119	0.021170904
C20orf195	-3.344027744	4.445946286	0.001358825	0.03063485
FAM13C	-3.346560006	2.804994826	0.010453099	0.098019301
HCG11	-3.347720392	2.413239143	0.015150304	0.122275726
INHA	-3.350694323	3.325635271	0.003598289	0.051799007
RP11-161D15.1	-3.351008713	3.898006781	0.001648214	0.033998787
CHN1	-3.352307883	3.845918706	0.001917416	0.036328095
LINC01013	-3.352651591	2.560779182	0.010510413	0.098019301
NME8	-3.352651591	2.560779182	0.010510413	0.098019301
TFDP3	-3.355397887	2.812109803	0.010453099	0.098019301
IL22RA2	-3.356823331	2.420335534	0.015150304	0.122275726
TMEM101	-3.358687948	3.209128122	0.005623714	0.067405742
LOC100506731	-3.359004143	2.696009243	0.014149938	0.117459486

ULBP1	-3.361263346	3.076130911	0.00399788	0.054449116
MAGEL2	-3.361493741	3.122835625	0.007116069	0.077573403
LOC102724938	-3.370981733	3.418959858	0.002624146	0.043412985
FAM196A	-3.371799218	3.13130549	0.007116069	0.077573403
CAGE1	-3.374718738	3.222366929	0.005015008	0.062599664
DSCR9	-3.379853666	4.887685652	0.000776497	0.022635483
RP11-727F15.11	-3.379871268	3.349877829	0.003233264	0.049354021
PLSCR1	-3.37991721	4.734422764	0.000703288	0.021318236
RHCE /// RHD	-3.380540907	3.426938522	0.002624146	0.043412985
CBLN2	-3.382971249	2.715177577	0.014149938	0.117459486
SERPING1	-3.383739979	2.996476105	0.005186874	0.063963283
ZNRF3	-3.385910951	2.836724638	0.009030241	0.0893286
LOC340581	-3.387763631	3.574483957	0.003014449	0.047508741
RP11-445L13__B.3	-3.38924701	2.895389095	0.007826126	0.082555619
SDF2	-3.390037159	3.434871235	0.002624146	0.043412985
GPR128	-3.391216285	2.521138615	0.010510413	0.098019301
RP5-1007H16.1	-3.394057068	3.0548381	0.004547753	0.058830288
TRAT1	-3.394511735	2.843676829	0.009030241	0.0893286
FAM153A /// FAM153B /// FAM153C /// LOC100507387 /// LOC101928349 /// LOC101930363	-3.395774762	2.786351586	0.010453099	0.098019301
FTX	-3.398695028	2.664049518	0.007434817	0.079735439
RP11-109D24.1	-3.399011989	3.404620028	0.002910357	0.0463016
ADAM3A	-3.399022006	5.777298351	0.000426163	0.017053238
LOC255177	-3.399904999	3.479882594	0.003998158	0.054449116
CCDC64B	-3.400068586	3.107982424	0.003523399	0.051251816
FAT4	-3.403636092	2.667994728	0.007434817	0.079735439
GEMIN5	-3.40564404	3.718108319	0.001774082	0.034862167
MIR3663HG	-3.407800382	2.534228024	0.010510413	0.098019301
NOG	-3.408844175	3.450600934	0.002369975	0.041241075
FAM64A	-3.40937173	3.251050773	0.004481104	0.058678986
DENND4A	-3.410396351	2.827643057	0.009030241	0.0893286
LOC102724537	-3.412481116	3.253628942	0.004481104	0.058678986
LOC554207	-3.413056548	2.386116875	0.018334706	0.136997112
EPHX4	-3.413614621	2.800734626	0.010453099	0.098019301
ILF3-AS1	-3.413735966	2.915281011	0.006803492	0.075213009
LOC100505664	-3.414477469	4.052960461	0.001550881	0.032822782
SMIM22	-3.414542294	5.531985875	0.00045317	0.017555101
HTATSF1P2	-3.416727315	1.91305069	0.026706022	0.172890711
TMEM130	-3.417632933	3.69719255	0.001932181	0.036328095

MSH4	-3.418358554	2.679762451	0.006295194	0.072628703
TMEM11	-3.418679855	3.258770813	0.004481104	0.058678986
DDC-AS1	-3.421265286	3.027163548	0.004547753	0.058830288
RCSD1	-3.42191353	4.241767461	0.001232269	0.028890692
GIPC2	-3.422115358	2.866030465	0.007826126	0.082555619
AQP11	-3.422420544	4.726651624	0.000703288	0.021318236
CCNJL	-3.423452649	2.977079227	0.005932006	0.069745944
IGDCC4	-3.425414766	2.395756356	0.015150304	0.122275726
BC045789	-3.429444403	2.315866363	0.022314819	0.155835874
TAAR9	-3.429743071	2.752725934	0.012140675	0.106947596
KLHL31	-3.429871127	3.0085128	0.005186874	0.063963283
AX747750 /// RP11-532F6.3	-3.433398436	2.480339151	0.012586629	0.109472446
LYZL1 /// LYZL2	-3.43453122	4.559292349	0.000916702	0.024755296
CXCL10	-3.439451308	3.232275254	0.005015008	0.062599664
FLJ31715	-3.449835345	3.901513151	0.001530156	0.032822782
MED22	-3.450171337	2.998932072	0.005186874	0.063963283
GAPDHP73 /// GAPDHP73	-3.45171491	3.594259173	0.002751	0.04462882
ASB15	-3.454477152	3.104497084	0.003523399	0.051251816
AL109706	-3.454614416	2.152240957	0.033664757	0.201030113
LOC100132167	-3.454644986	4.111593025	0.001272992	0.029320273
CDH13	-3.455854952	2.419561306	0.015150304	0.122275726
RP11-79P5.9	-3.457839231	3.057178786	0.004547753	0.058830288
FITM1	-3.461190555	3.734115112	0.001630752	0.033902317
ATP6V1G3	-3.461566349	2.839525266	0.009030241	0.0893286
LOC101930075 /// NPIPA1 /// NPIPA2 /// NPIPA3 /// NPIPA5 /// NPIPA7 /// NPIPA8 /// PKD1P1	-3.462157	5.429431967	0.000418001	0.016814533
AAED1	-3.462663255	4.98659049	0.000536948	0.019150697
NACAD	-3.463391002	5.050767658	0.000569837	0.019429053
RP11-1012E15.1	-3.463827946	4.320163728	0.000924518	0.024755296
LOC283665	-3.464020399	3.420143991	0.002624146	0.043412985
DCST2	-3.470996918	3.213247459	0.005015008	0.062599664
GHDC	-3.473198566	4.028290194	0.000996744	0.025675357
BC036830 /// OVAAL /// RP11-522D2.1	-3.474005465	3.745002563	0.001500646	0.03246148
DUSP27	-3.476627853	3.507502685	0.001942282	0.036328095

TNFSF4	-3.47737375	3.218540234	0.005015008	0.062599664
NMBR	-3.481276965	3.175011406	0.002756531	0.04462882
ZNF266	-3.481853282	2.914618152	0.006803492	0.075213009
CXCL6	-3.484803322	2.973279132	0.005932006	0.069745944
AC005537.2	-3.489446207	2.27371574	0.022314819	0.155835874
LHCGR	-3.489446207	2.27371574	0.022314819	0.155835874
WWC2-AS2	-3.491182958	3.032583197	0.004547753	0.058830288
ZNF861P /// ZNF861P	-3.4918281	3.520307062	0.00176232	0.034862167
RP11-440I14.2	-3.497634936	3.188573088	0.002756531	0.04462882
LOC100506790	-3.499031492	3.141294357	0.003112808	0.048204405
AC128677.4 /// CH17-132F21.1 /// IGKV1OR-1 /// IGKV1OR-1 /// IGKV1OR10-1 /// IGKV1OR10-1 /// IGKV1OR2-2	-3.499813403	3.887622779	0.001648214	0.033998787
AASDH	-3.502777004	3.946817378	0.001322163	0.0300216
RP11-10K16.1	-3.502962223	2.284172199	0.022314819	0.155835874
LOC100507516	-3.51029127	2.817818568	0.009030241	0.0893286
NTSR1	-3.514332343	3.539293601	0.00176232	0.034862167
PSRC1	-3.514883138	3.462860685	0.002143838	0.038830418
KLHL3	-3.516178023	3.749033933	0.001500646	0.03246148
AZGP1P1	-3.51751109	3.465072681	0.002143838	0.038830418
DYNC1LI1	-3.51784913	3.684440829	0.001932181	0.036328095
LXN	-3.51896966	2.824863484	0.009030241	0.0893286
IRS4	-3.524322192	3.257599266	0.004481104	0.058678986
RP11-471G13.5	-3.531125755	3.855353428	0.001776932	0.034862167
RPL26L1	-3.531508958	3.885538149	0.001648214	0.033998787
INHBE	-3.531752973	3.118150017	0.003523399	0.051251816
DUOX2	-3.534041949	3.311056249	0.003598289	0.051799007
OVGP1	-3.534744104	4.317856827	0.000924518	0.024755296
GYLTL1B	-3.534760708	4.680997775	0.000578654	0.019587534
TIGD3	-3.545966927	3.407573789	0.002624146	0.043412985
PRIMA1	-3.548625902	3.676375209	0.001932181	0.036328095
BD495725	-3.548829132	3.080101225	0.00399788	0.054449116
FAM223A /// FAM223B	-3.55549463	3.647103423	0.002106798	0.038575922
SLC38A11	-3.557866561	2.792181275	0.004569388	0.058850908
LDLRAD4-AS1	-3.561083006	3.540863398	0.001601365	0.033409649
ZNF432	-3.562306677	3.143473318	0.003112808	0.048204405
BC035400	-3.564013591	3.504293745	0.001942282	0.036328095
LRRTM2	-3.56504021	3.619186999	0.00229992	0.040411151

TMEM79	-3.566019682	3.545041975	0.001601365	0.033409649
LOC441454	-3.567720233	2.732673118	0.005352624	0.065287566
LOC339685	-3.568357302	4.084271369	0.000812836	0.02303396
GORAB	-3.570917752	1.774933781	0.034780972	0.205039552
BC062763	-3.570939523	3.549207947	0.001601365	0.033409649
RP4-545K15.5	-3.573081135	4.007262245	0.001068447	0.026816186
SLC20A2	-3.579829429	3.257013473	0.002176202	0.038849044
F13A1	-3.585962291	3.262139628	0.002176202	0.038849044
TIMP1	-3.587437981	4.585400187	0.000534505	0.019112011
ARNT2	-3.590486386	5.455307826	0.000285612	0.013582158
IL10	-3.595002321	3.644661897	0.002106798	0.038575922
AC092667.2	-3.602861224	2.690002985	0.006295194	0.072628703
DKFZP434C153 /// OBSCN	-3.60456646	4.062012539	0.000869392	0.024056245
ZFP36	-3.608160832	3.980703481	0.001146185	0.027503751
DAOA-AS1	-3.608483964	2.45604039	0.012586629	0.109472446
IGFL2	-3.613733977	3.864423007	0.001005703	0.025675357
LOC400756	-3.61601502	3.897649618	0.000862769	0.023929359
DUX1 /// DUX3 /// DUX5	-3.618326357	3.735823993	0.001500646	0.03246148
TAF1L	-3.626068728	3.516594658	0.00176232	0.034862167
DUSP11	-3.626343733	3.596234914	0.00132779	0.0300216
BMP10	-3.631044491	3.675385044	0.001932181	0.036328095
LOC101927720	-3.634377812	4.549922536	0.000588046	0.019876789
RAC3	-3.642125477	3.684847837	0.001932181	0.036328095
EEF1DP5 /// EEF1DP5	-3.64295992	3.309908353	0.001732606	0.034862167
PYGM	-3.6430948	4.345279366	0.00051512	0.018706971
RP11-255C15.4	-3.643245081	2.926403937	0.006803492	0.075213009
AK096592 /// YLPM1	-3.644390523	3.447882588	0.002369975	0.041241075
GTF2H2 /// GTF2H2B /// GTF2H2C	-3.648516306	3.108366345	0.003523399	0.051251816
STARD9	-3.649874302	3.762836118	0.001382397	0.031017767
GTPBP10	-3.650840043	4.181836406	0.000550733	0.019220167
RBMY1A1 /// RBMY1B /// RBMY1D /// RBMY1E /// RBMY1F /// RBMY1J	-3.651501808	3.655763965	0.00110686	0.027284747
FADD	-3.652874	3.833322514	0.001087349	0.027076494
LINC01346	-3.654360976	3.167732444	0.002756531	0.04462882

LOC102723845	-3.656216217	2.657931446	0.006295194	0.072628703
DSG1	-3.656780919	3.733521413	0.001500646	0.03246148
CPLX1	-3.658254104	3.661529548	0.001012527	0.025675357
BC041363	-3.661438851	3.003184837	0.005186874	0.063963283
ACOT12	-3.662208086	3.119754402	0.003523399	0.051251816
CA14	-3.66678851	3.910097557	0.000800201	0.022821694
LOC388210 /// LOC400499	-3.677943959	4.32916334	0.000545045	0.019220167
FAM47E	-3.679984154	2.422726806	0.015150304	0.122275726
CAPSL	-3.683664181	3.021572426	0.004547753	0.058830288
LRRC63	-3.683740325	2.827007953	0.003915642	0.054412443
ZIC2	-3.689317537	3.528782927	0.00176232	0.034862167
GPR87	-3.690222891	2.685409038	0.006295194	0.072628703
BBS5	-3.694653867	4.904272735	0.000341077	0.015002232
LOC101928770	-3.69529274	3.090482374	0.003523399	0.051251816
PRAMEF10	-3.70083635	4.089694361	0.000760495	0.022444169
PLS1	-3.702520628	3.153362615	0.003112808	0.048204405
ZSCAN16	-3.702520628	3.153362615	0.003112808	0.048204405
RP11-1109F11.3	-3.702780174	3.208207866	0.002446565	0.041860671
CD69	-3.703957073	2.531982021	0.010510413	0.098019301
KLHL23	-3.705697313	3.099142085	0.003523399	0.051251816
RP11-38C18.3	-3.707135687	4.962511339	0.000282856	0.013582158
BRE-AS1	-3.709040427	3.502698626	0.00176232	0.034862167
RP11-375I20.6	-3.709148896	3.102016558	0.003523399	0.051251816
CTB-43E15.1	-3.716607279	3.509132634	0.00176232	0.034862167
PTPRG-AS1	-3.718746769	2.708529563	0.005352624	0.065287566
ABCA11P	-3.719477101	3.467328758	0.002143838	0.038830418
CDC45	-3.725089687	2.929003217	0.002516721	0.042380758
LINC00561	-3.725991536	2.861626573	0.003367649	0.050071404
RP11-1072C15.4	-3.725991536	2.861626573	0.003367649	0.050071404
SLC7A13	-3.730069592	2.261561775	0.008203511	0.084939983
DAPK3	-3.73014939	2.865040773	0.003367649	0.050071404
LINC00588	-3.73324241	3.433302755	0.002369975	0.041241075
CTDP1	-3.736746971	4.307670196	0.000577	0.019559727
ITGAD	-3.738273979	3.939816942	0.000742826	0.022258248
PPY2	-3.744837893	3.072370576	0.00399788	0.054449116
LOC101929528	-3.746503034	3.444555501	0.002143838	0.038830418
LINC00895	-3.748356827	3.075300936	0.00399788	0.054449116
CRNN	-3.75390966	3.30380728	0.001732606	0.034862167
LOC101928545	-3.759642881	3.455717213	0.002143838	0.038830418
NEUROG2	-3.759874823	3.958537723	0.000690159	0.021170904
LAMB3	-3.761866738	3.257823206	0.001939802	0.036328095
OR2A20P /// OR2A9P	-3.76510879	3.65547896	0.001012527	0.025675357

LOC285902 /// ZNF273	-3.770465679	3.210268501	0.002446565	0.041860671
MED10	-3.77179169	3.902319128	0.000800201	0.022821694
HCP5	-3.775254338	3.468993183	0.002143838	0.038830418
TBCE	-3.775869194	3.581304562	0.00132779	0.0300216
CLIC1P1 /// CLIC1P1	-3.776501119	3.158277491	0.002756531	0.04462882
OR7C2	-3.782946251	3.768525019	0.000717575	0.02158421
PFN1	-3.789931097	3.614669874	0.001211515	0.028546542
TATDN1	-3.790285845	3.048067581	0.00399788	0.054449116
AVEN	-3.791689629	4.224912403	0.000457244	0.017638068
RERGL	-3.792019406	3.28323511	0.001939802	0.036328095
DEFB104A /// DEFB104B	-3.794551563	3.618629646	0.001211515	0.028546542
RP1-149C7.1	-3.801222523	2.421953909	0.015150304	0.122275726
USP39	-3.811109402	3.901870826	0.000800201	0.022821694
TCF24	-3.813024697	2.431334845	0.012586629	0.109472446
CTD-2619J13.17	-3.814687552	3.455175377	0.001013156	0.025675357
RP11-560G2.2	-3.816586093	3.834862218	0.001087349	0.027076494
PIGU	-3.81713389	4.327327353	0.00030226	0.013911006
LOC100506476	-3.821554755	3.308186383	0.001732606	0.034862167
AC006538.1	-3.824889491	4.109482374	0.000712016	0.021499589
RP11-286E11.2	-3.82569284	3.077599664	0.00399788	0.054449116
TMEM184C	-3.829167381	4.172838456	0.000550733	0.019220167
LY6H	-3.834250175	2.950948468	0.002516721	0.042380758
LOC100506272	-3.844390265	3.272544738	0.001939802	0.036328095
TTTY11	-3.844755262	3.327826935	0.001550588	0.032822782
ILF2	-3.846075397	4.244882813	0.000405112	0.01638019
HIST1H2BM	-3.846852696	3.705344388	0.0008504	0.023670138
EZH2	-3.849804248	3.032424254	0.001904897	0.036328095
ORAI3	-3.853246001	4.251183934	0.000405112	0.01638019
DAPK1-IT1 /// DAPK1-IT1	-3.853392755	3.163172736	0.002756531	0.04462882
STK17B	-3.860221866	3.6209593	0.001211515	0.028546542
TYRP1	-3.86553356	3.546075465	0.001601365	0.033409649
ZBTB34	-3.866482448	3.450177709	0.001013156	0.025675357
IL10RB	-3.868603415	2.979474025	0.00218621	0.038849044
RTN4IP1	-3.877225485	3.120739875	0.003112808	0.048204405
DYRK4	-3.878108007	5.434002149	0.000122575	0.009674353
NUPL2	-3.879441058	3.995889947	0.000556286	0.019220167
DLK1	-3.888397241	3.61166949	0.001211515	0.028546542
CXCL11	-3.888549148	3.469016365	0.000914836	0.024755296
LOC101928555	-3.89001557	2.59076494	0.008819448	0.0893286
PSMA8	-3.89301032	3.615636631	0.001211515	0.028546542

PARD3-AS1	-3.89476829	3.001257502	0.001904897	0.036328095
AV8S2 /// TRAV13-2 /// TRAV13-2	-3.895261397	3.198412926	0.002446565	0.041860671
BC047644	-3.896405501	3.259216209	0.001939802	0.036328095
CLCN6	-3.898294111	4.593086282	0.000191981	0.011215713
FDCSP	-3.899883279	2.856853772	0.003367649	0.050071404
RP11-288H12.4	-3.902647024	2.397437621	0.005404101	0.065480303
LOC100507462	-3.902942943	2.778575346	0.004569388	0.058850908
NPY5R	-3.906105936	3.32481693	0.001550588	0.032822782
SYDE2	-3.90643118	2.69559528	0.005352624	0.065287566
ANKRD20A12P /// LOC101059949 /// LOC101060632 /// LOC101927345	-3.907284047	3.145988328	0.003112808	0.048204405
TPRKB	-3.907497105	2.286962945	0.008203511	0.084939983
FLRT2 /// LOC100506718 /// LOC102724348	-3.910585555	3.14876529	0.002756531	0.04462882
NPHP3-AS1	-3.914056883	3.937832796	0.000690159	0.021170904
NLRP9	-3.915154084	3.440677888	0.001013156	0.025675357
SLC26A8	-3.915539334	4.094271135	0.000394426	0.01620832
DDX1	-3.915857015	3.635303582	0.00110686	0.027284747
LOC401176	-3.91660994	3.884286822	0.000442347	0.017384807
RP4-680D5.8	-3.925579147	2.619585831	0.007434817	0.079735439
RP11-493L12.3	-3.925861316	3.396765563	0.001248936	0.029078292
MBNL2 /// MBNL2	-3.929215814	2.800179775	0.003915642	0.054412443
P2RY6	-3.93774818	3.698781703	0.0008504	0.023670138
LOC100130458	-3.938635079	3.460749948	0.000914836	0.024755296
PLEKHG1	-3.94862618	3.360976099	0.001390333	0.031017767
IFNA17	-3.952753798	4.025309828	0.000518533	0.018706971
FBLN2	-3.954647415	3.878925227	0.000442347	0.017384807
LOC101927876	-3.968048081	3.377531208	0.001248936	0.029078292
ARF1	-3.974585969	4.749187832	0.000166697	0.010613982
KCNH1	-3.975157473	3.592651208	0.000617763	0.020256018
FCGR1B	-3.977967524	3.688930325	0.000927373	0.024755296
ACTR3P2 /// ACTR3P2	-3.987636461	3.213783316	0.002176202	0.038849044
SCAMPER	-3.989108326	3.604678459	0.000562075	0.019220167
LOC102723697	-3.994373135	3.153694398	0.00112921	0.027319677
RP11-496I2.2	-3.997102135	2.357781451	0.006635587	0.074656714
TPTE	-4.000036694	3.994601057	0.000556286	0.019220167
ZNF804A	-4.000116048	2.939994376	0.002516721	0.042380758

OR1A2	-4.001923445	3.515022146	0.000749483	0.022287152
RP1-178F10.1	-4.022779948	3.306464266	0.001732606	0.034862167
RPS10P7	-4.02434513	3.817217957	0.000560802	0.019220167
APELA	-4.02907567	3.11383173	0.001281795	0.029320273
RP1-142L7.8	-4.03633546	3.189160266	0.000997265	0.025675357
ST3GAL4-AS1	-4.041811645	3.789060664	0.000608184	0.020256018
PHGDH	-4.053015755	5.177189443	7.83E-05	0.008218811
MMP20	-4.055085436	2.725389069	0.005352624	0.065287566
DEFB108B	-4.055298564	3.205225724	0.000997265	0.025675357
DLEU2 /// MIR15A	-4.05596864	3.779176111	0.000660263	0.021170904
ASB12	-4.060063136	5.26449877	8.22E-05	0.008218811
LOC101927411	-4.060420279	4.347236701	0.000269841	0.013261729
RAG2	-4.061419123	3.457452175	0.000914836	0.024755296
GPR37	-4.062922869	2.82427943	0.003915642	0.054412443
ACSM2B	-4.074159249	3.350297383	0.001390333	0.031017767
DLGAP5	-4.078230752	3.414091224	0.001123917	0.027319677
CCPG1	-4.080749708	3.084466585	0.001458763	0.031878391
KIF11	-4.081938495	3.475088099	0.000914836	0.024755296
LOC101929007	-4.095029549	4.284934038	0.000185176	0.011018451
KRTAP4-3	-4.100246597	2.762588663	0.004569388	0.058850908
ZBTB6	-4.10056261	2.855503805	0.003367649	0.050071404
RP11-203B7.1	-4.104680534	3.313294844	0.001550588	0.032822782
LINC00924	-4.104683037	3.027493672	0.001904897	0.036328095
PLSCR4	-4.104687205	2.666904841	0.006295194	0.072628703
LPA /// PLG	-4.112520648	3.443531971	0.001013156	0.025675357
CNN3P1 /// CNN3P1	-4.115620373	3.385789845	0.001248936	0.029078292
SLC25A22	-4.118027612	4.337084336	0.000155342	0.010396859
RP11-245J9.5	-4.120314806	2.572541409	0.003029173	0.047599554
PRDM13	-4.129682035	3.048523919	0.001664638	0.033998787
SI	-4.134202316	3.824831107	0.000517642	0.018706971
LOC642757	-4.139111562	4.50470173	0.000149583	0.010097727
LOC100652999	-4.140035473	3.829932166	0.000517642	0.018706971
RP11-138I18.2	-4.146897461	4.086166489	0.000198579	0.011215713
POPDC3	-4.153271065	3.418112247	0.001123917	0.027319677
AF086294 /// RP11-485M7.3	-4.168600161	3.899898229	0.000409502	0.01650089
FAM46D	-4.16908701	2.912610074	0.002516721	0.042380758
SPTBN5	-4.176980069	3.815822263	0.000560802	0.019220167
ZNF383	-4.177414035	3.166233664	0.00112921	0.027319677
LOC441259 /// POLR2J /// POLR2J2 ///	-4.178136569	2.827102893	0.003367649	0.050071404

POLR2J3 /// UPK3BL				
FLJ31945	-4.187373901	3.447463167	0.001013156	0.025675357
ZNF157	-4.191677044	3.251740745	0.000783358	0.022635483
GOLGA6A /// GOLGA6B /// GOLGA6C /// GOLGA6D	-4.192110987	4.945164331	7.75E-05	0.008199045
RP11-489G11.3	-4.193609222	3.513436141	0.000749483	0.022287152
LOC642980	-4.196674194	4.091397046	0.000198579	0.011215713
RIBC2	-4.220067202	3.650270755	0.000467231	0.017818504
LOC101929480	-4.221212644	3.346768652	0.000554409	0.019220167
LOC102724487	-4.231207202	3.211999306	0.000882847	0.024285527
FAM43B	-4.236363846	3.608620032	0.000512118	0.018706971
LINC00403	-4.237608749	2.776497453	0.003915642	0.054412443
UGT2A3	-4.238260929	2.970590905	0.00218621	0.038849044
LOC101928140	-4.238932281	3.720401233	0.000357582	0.015133359
LOC101927424 /// LOC101929800 /// LOC440896 /// PGM5	-4.241116874	3.823904044	0.000234473	0.01235033
CTD-2021H9.3	-4.245487915	3.616553126	0.000512118	0.018706971
NVL	-4.246111953	3.778401238	0.000276657	0.013428147
STAM	-4.247314207	3.727723792	0.000327888	0.014451669
LOC728024	-4.247350369	4.354786194	0.000138542	0.010014253
C6orf57	-4.259872313	3.379994835	0.000496089	0.018410938
RSPO2	-4.267971445	2.995594207	0.001904897	0.036328095
TSPY1	-4.276891654	4.201402896	0.000125192	0.009674353
NSRP1	-4.289349177	3.710706566	0.000357582	0.015133359
ST8SIA1	-4.29783563	3.065355988	0.001458763	0.031878391
WNK4	-4.301977138	3.721750996	0.000357582	0.015133359
GPR61	-4.303420227	4.262895648	0.000196561	0.011215713
SPATA4	-4.310743716	4.714614238	6.33E-05	0.007934068
ZNF429	-4.313517713	3.982495239	0.000282223	0.013582158
BV03S1J2.2 /// IL23A /// TCRB /// TCRVB /// TRBC2 /// TRBC2 /// TRBJ2-7 /// TRBJ2-7 /// TRBV19	-4.317177912	4.158330535	0.000142344	0.010097727
LYG1	-4.318661065	3.892077451	0.000184391	0.011018451
LIMS3 /// LIMS3L	-4.320987888	3.563121004	0.000617763	0.020256018
RHOBTB1	-4.32262207	3.79368777	0.000254555	0.012805157

SMPX	-4.323576264	3.501681835	0.000749483	0.022287152
IQCF2	-4.324071428	5.441392639	2.62E-05	0.006472164
HECW1-IT1	-4.327326146	3.294226094	0.000696625	0.021170904
INTS2	-4.339676314	3.056180475	0.001664638	0.033998787
COQ10A	-4.341692828	4.052635848	0.000212712	0.011648108
FAM163A	-4.346107061	5.063603727	4.65E-05	0.007278211
RP11-348B17.1	-4.351238694	2.871206002	0.002906428	0.0463016
NPIPA5 /// NPIPB3 /// NPIPB6 /// NPIPB8	-4.355284231	4.670525818	7.59E-05	0.008199045
MSC	-4.355947536	3.82297684	0.000234473	0.01235033
LINC01142	-4.359241774	4.022973139	0.000244627	0.012493007
STT3B	-4.36090774	4.024450009	0.000244627	0.012493007
CTD-2555O16.4 /// MTHFD1	-4.370609302	3.170490056	0.000997265	0.025675357
TMEM27	-4.371054957	2.988741442	0.001904897	0.036328095
LINC00950	-4.371227063	3.888377872	0.000184391	0.011018451
HPSE2	-4.374643827	3.941494503	0.000146292	0.010097727
LOC101929413	-4.386195843	3.739484638	0.000327888	0.014451669
BC047651	-4.389655858	3.186759963	0.000997265	0.025675357
IDO2	-4.390509575	4.786899259	4.66E-05	0.007278211
ZFX /// ZFY	-4.391208958	3.099929124	0.001281795	0.029320273
LINC01019	-4.405943843	3.283633528	0.000696625	0.021170904
LINC00551	-4.414613416	3.025585197	0.001664638	0.033998787
CLRN3	-4.419133637	2.70044558	0.001782286	0.034862167
PTX3	-4.449024739	2.953424616	0.00218621	0.038849044
LOC101926959	-4.457175101	3.156176506	0.00112921	0.027319677
LOC100271840	-4.46725708	4.02360295	0.000108817	0.009228654
ERMN	-4.474812142	3.758775823	0.000301013	0.013911006
C11orf92	-4.479568661	3.175332229	0.000997265	0.025675357
RP11-359E8.5	-4.480020302	3.347475456	0.000554409	0.019220167
FAP	-4.509813636	4.06147814	8.79E-05	0.008464697
ZNF644	-4.51427027	3.565931733	0.00023965	0.012400588
MS4A12	-4.516373856	3.295566675	0.000620821	0.020256018
LOC100507033	-4.518746512	3.797495561	0.000254555	0.012805157
DNTTIP2	-4.522745267	3.015828282	0.001664638	0.033998787
RP11-138I17.1	-4.53479579	2.796741401	0.001280849	0.029320273
PDZD9	-4.543835519	5.154401476	1.91E-05	0.006472164
MGRN1	-4.545789549	4.517728246	6.90E-05	0.007990346
SYNM	-4.546981016	5.413542356	1.84E-05	0.006472164
RLN2	-4.54776738	3.233857699	0.000783358	0.022635483
TPT1P8	-4.561018929	3.417647534	0.000399481	0.01620832
MNS1	-4.564275507	3.573848924	0.000217408	0.011660639

LOC285768	-4.569120027	4.424352762	4.88E-05	0.00730635
C4orf22	-4.582787251	3.590057119	0.000217408	0.011660639
CRYAB	-4.584136964	4.223601059	0.000110389	0.009294849
COMP	-4.601741359	3.98641338	0.000117022	0.009478097
BORA	-4.613078443	3.819414692	0.000234473	0.01235033
SNX10	-4.625208523	3.473519674	0.00032401	0.014451669
DLGAP1-AS3	-4.63018798	3.39409661	0.000444759	0.017384807
LOC101927138	-4.632854794	3.396414105	0.000444759	0.017384807
LOC101930048 /// LOC101930053 /// VLDLR-AS1	-4.645336662	4.025284028	0.000101272	0.00891045
SGCZ	-4.64583315	2.890059645	0.000935753	0.024755296
LGSN	-4.654013678	3.414815475	0.000399481	0.01620832
KCTD9	-4.661868934	3.421653317	0.000399481	0.01620832
RP11-579O24.3	-4.677984868	3.744788257	0.000114341	0.009478097
RP4-730D4.1	-4.681871344	4.164917952	5.86E-05	0.007872709
CCNYL2	-4.684617923	3.882864696	0.000184391	0.011018451
KIAA0895	-4.726113862	3.787378223	9.63E-05	0.008697326
APOBEC3B	-4.748157629	3.407392597	0.000399481	0.01620832
C1orf50	-4.748220913	4.504573235	3.54E-05	0.006712069
SLC25A21-AS1	-4.758630823	3.669780136	0.000149393	0.010097727
DCAF4L1	-4.774271372	3.430172082	0.000359459	0.015133359
AX747191	-4.801231888	3.357735741	0.000496089	0.018410938
KRBOX1-AS1	-4.813520697	4.176089989	5.49E-05	0.007677442
ASZ1	-4.823328706	3.273752174	0.000696625	0.021170904
WFDC11	-4.838666651	4.357078121	2.64E-05	0.006472164
SH3GL2	-4.848700083	3.824843064	8.14E-05	0.008218811
ZNF165	-4.862843034	3.977083379	0.000117022	0.009478097
PLK2	-4.868326461	3.602558607	0.000197524	0.011215713
HAUS1	-4.873033029	3.986206569	0.000117022	0.009478097
ZSCAN31	-4.930143687	3.366664973	0.000496089	0.018410938
RNF149	-4.938638662	4.720324427	1.48E-05	0.006472164
CD27	-4.97700343	4.266684771	3.76E-05	0.006855942
KIAA1107	-4.983131249	3.178351396	0.000305554	0.013911006
PEX12	-5.016454905	3.898914291	5.90E-05	0.007872709
LINC01021	-5.041855272	3.351840839	0.000147935	0.010097727
RP11-330O11.3	-5.051118186	4.146412901	6.26E-05	0.007934068
PWRN2	-5.097031827	4.461044437	1.69E-05	0.006472164
LOC101929454	-5.115907183	3.158648798	0.000305554	0.013911006
AC087501.1	-5.133993487	4.152756225	2.14E-05	0.006472164
RBM34	-5.164995345	3.459827984	9.52E-05	0.008697326
LOC100131508	-5.236555502	3.636511061	0.000163744	0.010613982
DMRTC2	-5.237375322	4.617090489	9.15E-06	0.006321313
SERINC5	-5.288202579	3.682449939	0.00013648	0.009926277

LOC101060604 /// LOC101929910 /// LOC102725125 /// LOC613037 /// NPIPA5 /// NPIPB3 /// NPIPB4 /// NPIPB5 /// SLC7A5P1 /// SMG1P1 /// SMG1P3	-5.297730378	3.985569467	4.03E-05	0.006860454
RP11-171I2.1	-5.302248859	3.457076165	9.52E-05	0.008697326
MC4R	-5.354749931	4.280851257	1.28E-05	0.006472164
ANKRD55	-5.366864047	4.364638605	8.96E-06	0.006321313
COMMD1	-5.531760874	4.775595164	4.58E-06	0.005359422
CCDC79	-5.705726155	5.051950521	1.45E-06	0.005359422

2.3 Supplementary Table 3

Table S3. The 3061 upregulated DEGs in GSE92578.

Gene ID	log2Ratio(H-AsthS/H-sperm)	Up-Down-Regulation(H-AsthS/H-sperm)	p-value	q-value
A2ML1	1.381985233	Up	2.75E-09	9.70E-09
AAGAB	5.475089495	Up	4.49E-07	1.16E-06
AAR2	3.773299391	Up	2.92E-19	2.20E-18
ABAT	4.416051182	Up	0.000703575	0.000930449
ABCA12	2.15662803	Up	0.000404271	0.000570604
ABCB1	1.461207786	Up	3.02E-08	9.34E-08
ABCB9	4.56262039	Up	0.000329809	0.000475178
ABCC11	1.764310607	Up	1.37E-08	4.44E-08
ABCF1	5.15662803	Up	6.86E-06	1.44E-05
ABHD2	1.974365011	Up	1.04E-37	1.55E-36
ABHD5	4.834699935	Up	6.65E-05	0.000113504
ABI2	5.546194842	Up	2.27E-07	6.19E-07
ABLIM1	1.612055765	Up	1.46E-07	4.09E-07
ABLIM2	4.834699935	Up	6.65E-05	0.000113504
ABT1	1.299833913	Up	1.75E-07	4.85E-07
ACACA	5.802790687	Up	1.50E-08	4.84E-08
ACADVL	5.535139653	Up	2.53E-07	6.84E-07
ACAT2	1.497664948	Up	4.65E-06	1.01E-05
ACBD5	2.847797924	Up	3.71E-07	9.77E-07
ACE	8.572015609	Up	1.11E-188	8.81E-187
ACER1	3.213211558	Up	2.46E-10	9.77E-10
ACER2	1.507125277	Up	7.77E-05	0.000130462

ACKR1	2.179348107	Up	1.02E-05	2.08E-05
ACMSD	1.025642718	Up	6.72E-06	1.42E-05
ACOX1	1.277169562	Up	2.08E-05	3.97E-05
ACPT	1.963982952	Up	8.12E-05	0.000135608
ACSBG2	1.518041098	Up	2.54E-44	4.49E-43
ACSL1	1.214645851	Up	2.07E-18	1.49E-17
ACSL6	4.510515866	Up	0.000435181	0.000609805
ACTR3B	5.207652033	Up	4.58E-06	9.99E-06
ACTR5	3.197270015	Up	1.08E-13	5.75E-13
ACVR1B	2.336129034	Up	8.55E-06	1.77E-05
ADAM10	1.767548764	Up	0.000220677	0.000332005
ADAM23	4.419662436	Up	0.000691126	0.00091553
ADAM29	5.80048422	Up	1.54E-08	4.96E-08
ADAM8	1.20950759	Up	5.48E-09	1.87E-08
ADAMTS6	1.276805678	Up	1.24E-15	7.53E-15
ADAMTS9	1.016903266	Up	1.01E-05	2.06E-05
ADAMTSL3	7.705064655	Up	1.42E-25	1.43E-24
ADARB1	3.591189542	Up	1.78E-06	4.17E-06
adck5	3.358261891	Up	1.79E-05	3.46E-05
ADCY5	6.329755464	Up	1.32E-11	5.96E-11
ADD1	3.83741244	Up	7.87E-41	1.28E-39
ADGRB3	2.446134647	Up	1.20E-15	7.34E-15
ADGRG1	6.831088682	Up	1.66E-15	1.00E-14
ADGRL2	2.893593624	Up	2.95E-05	5.46E-05
ADIG	1.92780934	Up	1.41E-07	3.97E-07
ADIPOQ	5.402123693	Up	8.78E-07	2.17E-06
ADIPOR1	5.69268093	Up	5.06E-08	1.52E-07
ADIPOR2	2.529540054	Up	6.66E-10	2.52E-09
ADM5	1.095321807	Up	1.14E-40	1.85E-39
ADNP	7.361394781	Up	5.00E-21	4.11E-20
ADORA3	8.551896522	Up	3.75E-41	6.17E-40
ADPRH	4.589587437	Up	2.87E-07	7.68E-07
ADRA1A	1.576801923	Up	5.52E-05	9.61E-05
ADRM1	1.328547385	Up	5.85E-07	1.49E-06
ADTRP	1.263543234	Up	7.12E-07	1.78E-06
AES	4.834699935	Up	6.65E-05	0.000113504
AFAP1	1.761638039	Up	2.68E-49	5.25E-48
AFF4	1.120220335	Up	5.91E-34	7.92E-33
AFTPH	4.535139653	Up	0.000382164	0.000541831
AGAP1	1.291409029	Up	3.84E-22	3.33E-21
AGAP4	2.950835617	Up	8.56E-70	2.42E-68

AGBL3	1.001349805	Up	3.11E-13	1.60E-12
AGFG1	1.609922816	Up	1.20E-09	4.42E-09
AGO3	4.495981944	Up	5.05E-10	1.93E-09
AGO4	2.047693659	Up	1.02E-06	2.48E-06
AGTPBP1	5.788896246	Up	1.76E-08	5.61E-08
AHCTF1	1.142676605	Up	1.15E-28	1.30E-27
AHCYL1	2.13042296	Up	0.000504291	0.000695207
AHI1	2.134070598	Up	3.39E-07	8.97E-07
AIDA	1.884007575	Up	4.62E-06	1.01E-05
AK9	2.613398399	Up	3.13E-45	5.67E-44
AKAP10	2.213211558	Up	2.17E-07	5.93E-07
AKAP3	9.268911139	Up	4.45E-61	1.09E-59
AKAP4	1.325360362	Up	5.91E-14	3.21E-13
AKIP1	4.270639838	Up	2.27E-10	9.03E-10
AKIRIN1	5.004624937	Up	2.12E-05	4.03E-05
AKNA	9.724629724	Up	3.37E-78	1.07E-76
aknad1	1.112684682	Up	3.78E-34	5.10E-33
AKR1B10	4.419662436	Up	0.000691126	0.00091553
AKR1E2	5.612908512	Up	1.16E-07	3.31E-07
AKT1	1.511483144	Up	0.000207605	0.000315289
AKT3	3.317450824	Up	6.18E-14	3.36E-13
AKTIP	4.790756588	Up	8.77E-05	0.000145451
ALDH18A1	2.571665529	Up	0.000446586	0.000623907
ALDH1A2	4.816836425	Up	7.08E-15	4.10E-14
aldh3a1	1.469415471	Up	3.04E-05	5.60E-05
ALDH7A1	1.596089003	Up	1.51E-06	3.58E-06
ALDOA	4.939036595	Up	3.34E-05	6.11E-05
ALG3	4.190414892	Up	1.56E-30	1.88E-29
ALG9	8.358261891	Up	6.08E-37	8.86E-36
ALS2CR11	2.04087716	Up	0.000100085	0.000163606
AMACR	2.777214441	Up	0.000441061	0.000617367
AMD1	2.200205849	Up	6.80E-05	0.000115847
AMELX	4.419662436	Up	0.000691126	0.00091553
AMT	5.478556125	Up	4.35E-07	1.13E-06
AMY2B	2.782232515	Up	1.77E-09	6.39E-09
AMZ2	8.907180942	Up	4.31E-50	8.59E-49
ANAPC11	6.407286485	Up	3.88E-12	1.83E-11
ANAPC15	1.586789762	Up	0.000143515	0.000225421
ANGEL2	1.496684856	Up	0.000564464	0.00076582

ANGPT2	1.981541323	Up	0.000190985	0.000292294
ANK3	2.40510434	Up	2.07E-06	4.81E-06
ANKEF1	1.285241389	Up	1.04E-08	3.42E-08
ANKHD1	5.094725591	Up	1.10E-05	2.22E-05
ANKHD1-EIF4EBP3	1.982992255	Up	3.54E-30	4.23E-29
ANKLE2	1.818211812	Up	7.26E-17	4.79E-16
ankmy1	4.621296297	Up	0.000238755	0.000356613
ANKRD11	1.026696782	Up	0.000460739	0.000641841
ANKRD12	1.446501832	Up	2.77E-07	7.44E-07
ANKRD16	4.922162776	Up	3.75E-05	6.77E-05
ANKRD17	7.963982952	Up	1.31E-29	1.54E-28
ANKRD20A2	3.986071711	Up	1.37E-08	4.44E-08
ANKRD30A	1.341052601	Up	1.47E-08	4.75E-08
ANKRD42	3.732302762	Up	3.48E-12	1.65E-11
ANKRD62	1.180875576	Up	0.000533461	0.000728758
ANKS1B	3.981541323	Up	2.95E-26	3.05E-25
ANO2	5.227017358	Up	3.92E-06	8.66E-06
ANO6	1.229559553	Up	6.58E-05	0.000112787
ANP32E	2.911373037	Up	2.49E-05	4.67E-05
ANTXR1	2.077778515	Up	0.000427372	0.000599852
ANXA2	2.450275483	Up	1.03E-05	2.10E-05
ANXA2R	1.62057513	Up	5.42E-09	1.85E-08
AOC3	1.050741677	Up	9.14E-07	2.25E-06
AP1B1	2.765437273	Up	2.36E-28	2.65E-27
AP1M1	1.38178827	Up	2.17E-07	5.94E-07
AP2B1	1.029870888	Up	1.29E-10	5.27E-10
AP2M1	2.14811653	Up	4.55E-20	3.58E-19
AP3D1	3.076329752	Up	6.97E-24	6.51E-23
AP3M1	3.275947314	Up	1.21E-05	2.41E-05
AP3S1	2.027345013	Up	0.000119426	0.000191763
AP4B1	1.308631124	Up	6.62E-07	1.67E-06
APAF1	2.669614952	Up	6.60E-08	1.95E-07
APBB1	4.834699935	Up	6.65E-05	0.000113504
APBB2	7.119732185	Up	2.61E-18	1.87E-17

APBB3	2.264547094	Up	1.57E-23	1.45E-22
APCDD1	4.478556125	Up	9.00E-07	2.21E-06
APH1B	2.004624937	Up	5.93E-06	1.26E-05
API5	5.294131554	Up	2.25E-06	5.18E-06
APOBEC 3H	4.419662436	Up	0.000691126	0.00091553
APOE	4.509386555	Up	0.000437761	0.000613296
APTX	5.535139653	Up	2.53E-07	6.84E-07
AQP5	1.025789547	Up	1.19E-35	1.67E-34
ARCN1	1.597308321	Up	7.99E-14	4.31E-13
AREL1	2.349273108	Up	2.43E-06	5.57E-06
ARFGAP 1	5.121581083	Up	8.97E-06	1.85E-05
ARFGAP 3	1.558730521	Up	4.97E-33	6.48E-32
ARHGAP 11A	1.764605486	Up	2.34E-05	4.43E-05
ARHGAP 17	4.109322315	Up	9.76E-14	5.22E-13
ARHGAP 19	6.319482558	Up	1.55E-11	6.94E-11
ARHGAP 45	3.760339909	Up	2.18E-35	3.04E-34
ARHGAP 5	2.574633021	Up	4.39E-41	7.19E-40
ARHGDI A	4.423264673	Up	0.0006789	0.000905146
ARHGEF 12	5.788896246	Up	1.76E-08	5.61E-08
ARHGEF 26	6.478556125	Up	1.19E-12	5.87E-12
ARHGEF 40	5.075014265	Up	1.27E-05	2.53E-05
ARHGEF 6	2.097734341	Up	0.000659832	0.000881714
ARHGEF 7	5.40273204	Up	8.74E-07	2.16E-06
ARID1B	5.480862592	Up	4.25E-07	1.11E-06
ARID4A	2.619518653	Up	8.49E-09	2.83E-08
ARID4B	1.466128534	Up	1.57E-23	1.45E-22
ARL11	1.254238827	Up	6.48E-07	1.64E-06
ARL13B	5.266991273	Up	2.83E-06	6.41E-06
ARL2	2.149161702	Up	3.65E-13	1.87E-12
ARL2BP	2.022281941	Up	2.23E-12	1.07E-11

ARL4A	3.118408708	Up	4.11E-26	4.22E-25
ARL6IP4	2.059212408	Up	1.97E-11	8.74E-11
ARL8A	3.642054857	Up	0.000548719	0.000746049
ARMC1	1.986703029	Up	2.19E-05	4.15E-05
ARMC3	1.167438331	Up	1.33E-05	2.65E-05
ARMC4	4.439957198	Up	0.000624682	0.000839011
ARMC5	1.83141486	Up	4.00E-06	8.81E-06
ARMC8	3.741590531	Up	0.000303865	0.000442212
armc9	1.254978545	Up	6.32E-13	3.17E-12
ARNT	5.358261891	Up	1.30E-06	3.11E-06
ARNTL2	4.741590531	Up	0.000118566	0.000190485
ARRDC1	2.320126762	Up	9.05E-05	0.000149424
ARSA	6.227017358	Up	6.20E-11	2.61E-10
ARSB	1.519305569	Up	2.42E-05	4.56E-05
ARSG	4.419662436	Up	0.000691126	0.00091553
ASAP1	6.779558381	Up	4.71E-15	2.77E-14
ASB13	2.004624937	Up	5.10E-05	8.95E-05
ASB2	2.092917325	Up	1.58E-06	3.73E-06
ASCC1	4.929780427	Up	3.56E-05	6.47E-05
ASCC2	6.642054857	Up	6.64E-14	3.59E-13
ASCC3	5.389902569	Up	9.80E-07	2.40E-06
ASS1	1.100175636	Up	3.10E-29	3.58E-28
ASTN2	3.199077816	Up	0.000742471	0.000976297
ASXL1	4.840997959	Up	6.39E-05	0.000109697
ASXL2	1.773575342	Up	1.20E-06	2.90E-06
ATAD2	1.127481684	Up	2.68E-16	1.70E-15
ATAD2B	6.004624937	Up	1.30E-09	4.78E-09
ATAD5	1.444753417	Up	1.52E-12	7.43E-12
ATAT1	4.828374297	Up	6.92E-05	0.000117773
atf2	5.076540119	Up	1.26E-05	2.51E-05
ATF7IP	2.398225265	Up	1.43E-11	6.42E-11
ATG13	6.642054857	Up	6.64E-14	3.59E-13
ATG7	4.051930651	Up	2.71E-20	2.16E-19
ATMIN	1.80136034	Up	8.80E-36	1.24E-34
ATP11C	4.535139653	Up	0.000382164	0.000541831
ATP13A3	5.709988533	Up	4.20E-08	1.28E-07
ATP1A1	1.881178254	Up	1.70E-05	3.30E-05
ATP1A3	1.049049436	Up	3.73E-18	2.64E-17
ATP2A1	2.497664948	Up	0.00076148	0.000997826
ATP2B2	1.301017939	Up	3.93E-07	1.03E-06
ATP2C1	6.623907511	Up	9.28E-14	4.98E-13
ATP5A1	6.507125277	Up	7.35E-13	3.67E-12
ATP5D	4.922162776	Up	3.75E-05	6.77E-05
ATP5G3	2.188852	Up	0.000366649	0.00052358

ATP6AP1	1.249560449	Up	0.000406326	0.000573227
ATP6V0A1	1.030273927	Up	0.000232047	0.00034755
ATP6V0D1	2.922162776	Up	0.000537549	0.000733476
ATP6V0E2	2.526216198	Up	9.34E-36	1.32E-34
ATP6V1E2	1.20909545	Up	9.21E-05	0.000151774
ATP6V1G1	3.741590531	Up	0.000303865	0.000442212
ATP6V1H	3.10771843	Up	3.23E-06	7.23E-06
ATP8A2	1.140508365	Up	2.56E-05	4.80E-05
ATP9A	1.950177153	Up	0.000722703	0.000953274
ATRAID	5.13042296	Up	8.39E-06	1.74E-05
ATXN2	5.874715614	Up	6.49E-09	2.19E-08
ATXN2L	5.348822195	Up	1.41E-06	3.36E-06
AUNIP	1.711647379	Up	4.75E-09	1.63E-08
AURKAIP1	1.157017632	Up	0.000102622	0.000167403
AVEN	3.227017358	Up	5.58E-05	9.70E-05
AXDND1	1.482266444	Up	2.43E-49	4.76E-48
AZIN1	1.309154728	Up	8.19E-56	1.83E-54
AZIN2	6.220821092	Up	6.78E-11	2.85E-10
B3GALNT2	3.223456303	Up	5.05E-08	1.52E-07
B4GALT3	1.950177153	Up	0.000722703	0.000953274
B9D1	3.741590531	Up	0.000303865	0.000442212
BAALC	2.044153301	Up	5.85E-13	2.94E-12
BACE1	1.477318999	Up	5.65E-13	2.85E-12
BAD	3.939875127	Up	8.27E-05	0.000137983
BAG6	1.421333834	Up	4.02E-11	1.73E-10
BAIAP2	4.527350305	Up	0.000398282	0.000562965
BANP	1.54740672	Up	4.07E-56	9.12E-55
BAZ2A	4.736989546	Up	2.64E-23	2.41E-22
BAZ2B	1.4078254	Up	2.75E-08	8.56E-08
BBC3	4.646170966	Up	0.000207454	0.000315114
BBS12	4.633787241	Up	0.000222547	0.000334303
BBS7	4.750220836	Up	0.000112532	0.000182234
bbx	1.491328559	Up	0.000607025	0.000818124
BCAN	2.945902497	Up	1.21E-05	2.41E-05
BCKDK	2.492863962	Up	5.73E-09	1.95E-08
BCL11A	5.500320099	Up	3.54E-07	9.34E-07
BCL2L1	6.374479127	Up	6.56E-12	3.04E-11

BCL2L13	2.249385888	Up	5.91E-38	8.90E-37
BCL2L14	1.213211558	Up	3.49E-05	6.36E-05
BCL2L2	5.705064655	Up	4.43E-08	1.34E-07
BCLAF1	7.7202743	Up	8.54E-26	8.65E-25
BCR	1.022614561	Up	4.30E-13	2.19E-12
BDH1	1.330277911	Up	5.64E-12	2.63E-11
BDH2	4.49233994	Up	0.000478375	0.000662937
BDNF	4.668602012	Up	0.000182423	0.000279992
BDP1	1.072112459	Up	2.24E-06	5.17E-06
BEND2	4.922162776	Up	3.75E-05	6.77E-05
BEND7	1.869821076	Up	0.000554761	0.000753633
BEST1	4.424463422	Up	0.000674873	0.000900279
BEST3	2.24631987	Up	3.81E-10	1.48E-09
BICD2	1.190595749	Up	5.31E-13	2.68E-12
BLCAP	7.377267543	Up	3.22E-21	2.67E-20
BMP1	1.343713583	Up	1.27E-05	2.53E-05
BMS1	1.075756086	Up	8.71E-06	1.80E-05
BNC1	1.085293602	Up	7.63E-06	1.59E-05
BNIPL	1.93587877	Up	0.000293331	0.00042866
BOLA1	4.419662436	Up	0.000691126	0.00091553
BOLL	4.637926972	Up	0.000217396	0.000327473
BPIFA1	4.419662436	Up	0.000691126	0.00091553
BPIFA3	1.405499984	Up	6.56E-05	0.000112394
BPTF	5.796786185	Up	1.61E-08	5.16E-08
BRCA1	1.41141388	Up	4.30E-08	1.31E-07
BRD2	6.083007055	Up	4.66E-10	1.79E-09
BRD9	2.166930739	Up	1.70E-16	1.10E-15
BRDT	7.276648126	Up	4.94E-20	3.88E-19
BRE	1.37366616	Up	2.08E-11	9.21E-11
BRF1	5.242878013	Up	0.000512891	0.000703416
bri3	4.419662436	Up	0.000691126	0.00091553
BRINP3	1.642054857	Up	3.22E-12	1.53E-11
BRK1	1.118843368	Up	1.80E-06	4.20E-06
BRSK2	7.373858746	Up	3.54E-21	2.93E-20
BRWD1	1.064837475	Up	1.05E-20	8.55E-20
BSDC1	2.610280664	Up	7.94E-40	1.26E-38
BST1	4.741590531	Up	0.000118566	0.000190485
BTBD7	2.72063424	Up	1.73E-07	4.80E-07
BTBD9	2.405825109	Up	9.89E-10	3.68E-09
btn2a1	3.748637336	Up	1.43E-06	3.41E-06
BTN3A1	4.535139653	Up	0.000382164	0.000541831
BTN3A3	4.922162776	Up	3.75E-05	6.77E-05
btnl8	4.56262039	Up	0.000329809	0.000475178
BTRC	1.257231971	Up	2.14E-11	9.48E-11

BUB1B-P AK6	3.571665529	Up	6.68E-09	2.26E-08
C10orf126	1.263543234	Up	5.16E-05	9.04E-05
c10orf53	2.267659342	Up	0.000149652	0.000234247
C11orf44	1.438771259	Up	3.17E-06	7.11E-06
C11ORF4 9	5.044153301	Up	1.04E-09	3.87E-09
C11orf63	1.873425777	Up	9.44E-10	3.52E-09
C12orf40	5.082627449	Up	1.20E-05	2.40E-05
C12orf50	1.320126762	Up	1.44E-11	6.48E-11
c12orf66	1.241875317	Up	0.000192787	0.000294733
c14orf159	4.849055228	Up	6.06E-05	0.000104596
C14orf80	5.419662436	Up	7.50E-07	1.87E-06
c14orf93	4.082627449	Up	2.90E-05	5.37E-05
c15orf59	1.081614676	Up	0.000621968	0.000835698
c16orf45	3.893709035	Up	6.16E-18	4.32E-17
C16orf78	1.624776866	Up	1.01E-07	2.90E-07
C17orf105	2.173501849	Up	4.32E-11	1.85E-10
C17orf64	2.466968151	Up	1.96E-05	3.76E-05
C17orf78	2.10771843	Up	0.000173302	0.00026746
c17orf80	5.870323845	Up	6.84E-09	2.31E-08
c18orf54	4.563708806	Up	0.000327873	0.000472668
c18orf8	1.457475038	Up	6.24E-14	3.38E-13
C19orf38	3.890627203	Up	1.09E-19	8.39E-19
C1orf101	1.193781028	Up	0.000379196	0.000539882
C1ORF10 6	4.771508547	Up	2.79E-38	4.24E-37
c1orf116	4.672643177	Up	0.000178212	0.000274206
C1orf159	1.207811797	Up	3.92E-09	1.36E-08
C1orf189	2.451371296	Up	2.32E-13	1.21E-12
c1orf43	9.025462929	Up	1.85E-53	3.97E-52
C1QTNF1	1.27079905	Up	3.13E-06	7.04E-06
C1QTNF3	4.755945824	Up	0.000108683	0.000176504
C20orf203	4.419662436	Up	0.000691126	0.00091553
c21orf2	5.478556125	Up	4.35E-07	1.13E-06
C21orf58	1.701617643	Up	2.88E-77	9.03E-76
c21orf62	7.11082434	Up	3.24E-18	2.30E-17
C22orf23	5.741590531	Up	2.98E-08	9.22E-08
C22orf31	1.992241212	Up	4.42E-13	2.24E-12
C2CD5	2.056886242	Up	2.74E-06	6.23E-06
C2orf16	1.117338899	Up	5.21E-36	7.39E-35
c2orf49	2.214536608	Up	1.96E-15	1.18E-14
C2orf69	1.922162776	Up	0.000128602	0.000205005
c2orf70	2.834699935	Up	5.11E-05	8.96E-05

C3	1.319676162	Up	1.33E-14	7.57E-14
C3orf20	7.508962835	Up	7.10E-23	6.37E-22
C3orf35	5.358261891	Up	1.30E-06	3.11E-06
c3orf52	1.291596882	Up	3.11E-07	8.30E-07
c3orf80	3.543472211	Up	1.72E-09	6.22E-09
C4ORF17	2.922162776	Up	0.000537549	0.000733476
c4orf46	2.956544233	Up	0.000426749	0.000599035
c5orf15	4.419662436	Up	1.60E-06	3.77E-06
C5orf51	1.767585739	Up	5.82E-05	0.000100797
C6orf10	2.417881431	Up	8.46E-13	4.21E-12
C6orf136	3.408166797	Up	1.01E-07	2.90E-07
C6orf163	1.48451358	Up	1.80E-07	4.97E-07
C6orf201	3.007383387	Up	1.21E-45	2.22E-44
C6orf229	2.963982952	Up	5.70E-07	1.45E-06
C6orf25	5.561935039	Up	2.00E-53	4.29E-52
C6orf89	1.731521034	Up	4.48E-12	2.10E-11
C7orf49	1.418178351	Up	9.26E-34	1.24E-32
C7ORF50	4.834699935	Up	6.65E-05	0.000113504
C7orf73	1.368132135	Up	0.000135799	0.000215407
c8orf33	1.477761908	Up	4.00E-05	7.15E-05
C9orf131	2.97292053	Up	9.36E-24	8.70E-23
C9orf153	2.449409779	Up	8.98E-08	2.60E-07
C9orf43	2.642333218	Up	7.48E-45	1.35E-43
C9orf62	2.219363785	Up	7.93E-07	1.97E-06
CA12	6.28722948	Up	2.54E-11	1.11E-10
CABIN1	1.577352949	Up	2.87E-06	6.50E-06
CABS1	1.393525941	Up	3.70E-99	1.49E-97
CABYR	1.829072461	Up	3.59E-15	2.12E-14
CACFD1	5.299368202	Up	2.15E-06	4.98E-06
CACNA1B	7.26096469	Up	7.46E-20	5.80E-19
CACNA1C	2.712119402	Up	1.59E-191	1.29E-189
CACNA1G	5.991339246	Up	1.54E-09	5.62E-09
CACNA1H	1.501361499	Up	3.21E-57	7.34E-56
CACNA1S	1.337200276	Up	3.14E-06	7.06E-06
CADM1	6.15662803	Up	1.70E-10	6.83E-10
CADM3	5.082627449	Up	1.20E-05	2.40E-05
CADPS2	5.004624937	Up	2.12E-05	4.03E-05
CAGE1	5.667589949	Up	6.61E-08	1.95E-07
CALM2	3.07293095	Up	8.91E-05	0.000147358

CAMK2N1	3.741590531	Up	0.000303865	0.000442212
CAMK2N2	1.098454069	Up	1.52E-34	2.07E-33
CAMSAP2	1.085012442	Up	8.57E-05	0.000142477
CAMSAP3	7.249850141	Up	9.98E-20	7.71E-19
CAMTA2	1.067943743	Up	1.60E-40	2.57E-39
CANX	1.405211236	Up	2.58E-68	7.11E-67
CAP1	7.627685395	Up	1.79E-24	1.71E-23
CAPRIN1	1.892260751	Up	7.49E-08	2.19E-07
CAPZA3	1.792879759	Up	5.11E-25	5.01E-24
CARF	1.316568943	Up	6.92E-06	1.45E-05
CARHSP1	5.449409779	Up	5.71E-07	1.45E-06
CARMIL1	4.419662436	Up	0.000691126	0.00091553
CASC3	3.555230898	Up	4.61E-51	9.36E-50
CASP2	3.939620538	Up	3.58E-19	2.68E-18
CAST	4.508256359	Up	0.000440357	0.000616533
CATSPER2	1.265334289	Up	0.000136632	0.00021667
CATSPER3	2.571665529	Up	0.000446586	0.000623907
CBR4	3.074378015	Up	4.21E-05	7.50E-05
CBX5	1.305548297	Up	2.64E-05	4.94E-05
CCDC105	1.950177153	Up	3.46E-05	6.32E-05
CCDC110	5.09623075	Up	1.09E-05	2.20E-05
CCDC127	4.834699935	Up	6.65E-05	0.000113504
CCDC134	1.145040056	Up	1.12E-20	9.05E-20
CCDC138	1.885326008	Up	0.000481545	0.000666568
CCDC14	1.314390393	Up	5.19E-29	5.96E-28
CCDC144A	1.27173375	Up	2.45E-40	3.93E-39
CCDC151	3.034205927	Up	1.81E-08	5.77E-08
CCDC158	1.330395098	Up	2.23E-09	7.96E-09
CCDC168	1.152249735	Up	1.20E-134	6.68E-133
CCDC169	4.779558381	Up	9.40E-05	0.000154506
CCDC170	1.519198109	Up	0.000188837	0.000289188
CCDC171	2.709169053	Up	0.000151978	0.00023744
CCDC189	1.352578841	Up	4.32E-05	7.69E-05
CCDC191	1.062110431	Up	3.68E-05	6.68E-05
CCDC25	4.741590531	Up	0.000118566	0.000190485

CCDC36	1.294131554	Up	6.21E-05	0.000106814
CCDC40	1.415733169	Up	1.49E-07	4.17E-07
CCDC51	1.188579758	Up	4.06E-05	7.27E-05
CCDC62	1.440878922	Up	3.78E-10	1.47E-09
CCDC66	7.467404384	Up	2.44E-22	2.13E-21
CCDC7	1.128106693	Up	1.69E-05	3.28E-05
CCDC71L	3.542519184	Up	3.96E-06	8.73E-06
CCDC74B	3.17106312	Up	0.000207186	0.000314751
CCDC77	2.198882034	Up	5.22E-09	1.78E-08
CCDC82	1.247082981	Up	1.17E-10	4.79E-10
CCDC88A	1.237355296	Up	6.04E-08	1.79E-07
CCDC88C	1.018194128	Up	9.87E-05	0.000161453
CCDC93	1.278306587	Up	5.66E-05	9.83E-05
CCHCR1	1.70613625	Up	1.97E-27	2.13E-26
CCL18	3.227017358	Up	5.58E-05	9.70E-05
CCL5	1.378613644	Up	9.40E-11	3.89E-10
CCNA1	2.030571883	Up	7.15E-06	1.50E-05
CCNB1	2.642054857	Up	0.00026095	0.000386382
CCNB3	3.952394978	Up	2.19E-08	6.89E-08
ccnjl	2.006949792	Up	2.11E-07	5.77E-07
CCP110	4.642054857	Up	0.000212366	0.000320567
CCPG1	1.565178334	Up	2.92E-10	1.15E-09
CCSER1	4.082627449	Up	2.90E-05	5.37E-05
CCSER2	1.576997114	Up	3.14E-22	2.73E-21
CCT3	1.299686688	Up	1.84E-12	8.91E-12
CCT6A	5.806013559	Up	1.45E-08	4.68E-08
CCT7	2.10771843	Up	0.000173302	0.00026746
CCT8L2	1.915619931	Up	1.18E-06	2.85E-06
CCZ1	1.973295996	Up	2.59E-05	4.85E-05
CD101	7.834699935	Up	1.60E-27	1.73E-26
CD109	4.741590531	Up	0.000118566	0.000190485
CD180	3.177092133	Up	2.32E-25	2.31E-24
CD22	4.151981665	Up	3.21E-14	1.78E-13
CD24	1.100404351	Up	0.000130553	0.000207882
CD4	4.419662436	Up	0.000691126	0.00091553
CD46	1.691741981	Up	7.76E-17	5.11E-16
CD52	2.788896246	Up	4.99E-06	1.08E-05
CD59	5.592789869	Up	1.43E-07	4.01E-07
CD81	1.493803156	Up	0.000711119	0.000939831
CDC123	3.337200276	Up	1.97E-07	5.41E-07
CDC14B	1.703531894	Up	4.52E-14	2.48E-13

CDC20B	1.08113783	Up	2.60E-06	5.92E-06
CDC25A	2.773299391	Up	8.83E-05	0.000146179
CDC42BP A	1.167275274	Up	0.000178197	0.000274196
CDH11	1.15662803	Up	1.09E-05	2.21E-05
CDH26	1.798174059	Up	8.86E-05	0.000146689
CDH7	2.709169053	Up	0.000151978	0.00023744
CDHR3	1.431237586	Up	3.60E-24	3.41E-23
CDHR4	2.466968151	Up	1.96E-05	3.76E-05
CDHR5	1.743166386	Up	1.46E-20	1.18E-19
CDIP1	7.391129351	Up	2.18E-21	1.83E-20
CDIPT	1.462451709	Up	7.82E-08	2.28E-07
CDK11A	1.563051305	Up	1.08E-12	5.34E-12
CDK11B	2.329872802	Up	1.03E-05	2.11E-05
CDK14	6.082627449	Up	4.68E-10	1.80E-09
CDK17	1.332199595	Up	0.000478203	0.000662761
CDK18	4.626514006	Up	0.000231861	0.000347283
CDK20	5.240692295	Up	3.51E-06	7.81E-06
CDK5RA P3	5.082627449	Up	1.20E-05	2.40E-05
CDKAL1	3.17383732	Up	3.11E-05	5.73E-05
CDKL3	2.166613689	Up	1.46E-08	4.71E-08
CDKN2A IP	1.370752835	Up	2.86E-08	8.89E-08
CDKN3	1.537210012	Up	5.89E-11	2.49E-10
CDON	1.351686729	Up	0.000145021	0.000227607
CDR2	3.294131554	Up	3.16E-05	5.81E-05
CDV3	2.627997958	Up	7.20E-08	2.11E-07
CEACAM 16	3.004624937	Up	0.000305949	0.000444199
CECR1	4.529580128	Up	0.000393608	0.000556866
CELF2	2.411204164	Up	3.57E-11	1.54E-10
CELF6	5.561531152	Up	1.95E-07	5.37E-07
CENPC	5.294131554	Up	2.25E-06	5.18E-06
CENPN	5.338684639	Up	1.54E-06	3.64E-06
CENPU	1.004624937	Up	6.24E-05	0.00010737
CEP112	2.005497613	Up	2.57E-17	1.74E-16
CEP128	1.14212846	Up	8.82E-06	1.82E-05
CEP152	1.289594082	Up	5.70E-32	7.18E-31
CEP192	1.022636557	Up	2.36E-20	1.89E-19
CEP57L1	4.112684682	Up	2.30E-05	4.34E-05
CEP70	4.939875127	Up	3.32E-05	6.08E-05
CEP72	2.25320129	Up	1.04E-17	7.21E-17
CEP76	2.785790335	Up	1.09E-18	7.93E-18

CEP85	2.56262039	Up	1.17E-08	3.83E-08
CEP95	1.59722379	Up	9.64E-23	8.60E-22
CERS1	1.188353182	Up	4.80E-06	1.04E-05
CERS6	1.18833689	Up	7.65E-07	1.91E-06
CES1	5.763070258	Up	2.35E-08	7.37E-08
CES2	4.15662803	Up	1.62E-05	3.16E-05
CETN1	2.097734341	Up	0.000659832	0.000881714
CFAP20	1.451371296	Up	2.47E-07	6.69E-07
Cfap43	1.571665529	Up	0.00030787	0.000446589
CFAP97	1.552397681	Up	2.27E-22	1.99E-21
CFLAR	7.881060026	Up	2.97E-28	3.32E-27
chchd5	4.817141564	Up	3.04E-12	1.45E-11
CHD1	1.559028351	Up	4.65E-74	1.39E-72
CHEK1	1.137392456	Up	0.000126599	0.000202217
CHFR	3.062968923	Up	1.21E-11	5.47E-11
CHID1	4.419662436	Up	0.000691126	0.00091553
CHL1	3.478556125	Up	5.69E-06	1.22E-05
CHN1	1.436055562	Up	2.33E-05	4.40E-05
CHORDC1	1.880415653	Up	3.79E-17	2.54E-16
CHPF	3.051172596	Up	1.27E-16	8.25E-16
CHRDL2	4.40273204	Up	0.000751203	0.000986449
CHRM1	2.249737434	Up	3.99E-05	7.15E-05
CHRM2	1.950177153	Up	1.12E-36	1.62E-35
CHRNA10	1.391917445	Up	1.22E-07	3.46E-07
CHRND	3.436735949	Up	8.35E-17	5.49E-16
CHRNE	1.350001558	Up	1.06E-17	7.34E-17
CHST15	2.014600107	Up	7.34E-17	4.84E-16
CHSY1	2.834699935	Up	1.01E-08	3.35E-08
CHUK	1.834699935	Up	0.000318327	0.000460085
CIAPIN1	3.061768844	Up	2.94E-18	2.10E-17
CIRBP	1.948582672	Up	5.99E-33	7.79E-32
CIT	3.402384444	Up	2.53E-16	1.61E-15
CIZ1	6.375719088	Up	6.43E-12	2.98E-11
CKAP5	4.308742151	Up	3.07E-94	1.18E-92
CLASP1	1.636240017	Up	1.10E-07	3.13E-07
CLASRP	1.10235256	Up	2.93E-07	7.83E-07
CLCN2	1.434971826	Up	2.96E-08	9.18E-08
CLCN3	1.775555112	Up	0.000672401	0.000897226
CLCNKA	2.404976025	Up	3.13E-11	1.36E-10
CLCNKB	1.228712087	Up	2.65E-05	4.94E-05
CLDN10	5.473353052	Up	4.56E-07	1.18E-06
CLDN12	4.741590531	Up	0.000118566	0.000190485

CLDN5	2.06568462	Up	2.11E-08	6.66E-08
CLEC18B	2.711390293	Up	3.80E-05	6.85E-05
CLEC2D	5.564252706	Up	1.90E-07	5.24E-07
CLIP4	2.123131118	Up	6.05E-06	1.29E-05
CLK2	4.656291798	Up	3.34E-36	4.76E-35
CLK4	1.401571979	Up	1.06E-20	8.57E-20
CLOCK	5.15662803	Up	6.86E-06	1.44E-05
CLPB	2.886422448	Up	4.86E-115	2.29E-113
CLPX	1.200561063	Up	1.54E-05	3.02E-05
clstn3	1.216099838	Up	3.37E-05	6.16E-05
CLTA	1.072088834	Up	0.000115604	0.000186747
CLTCL1	8.024524494	Up	1.23E-30	1.49E-29
CLUAP1	1.5323939	Up	7.30E-13	3.64E-12
CMPK2	3.088257063	Up	1.88E-12	9.09E-12
CMSS1	4.419662436	Up	0.000691126	0.00091553
CMTM2	1.263543234	Up	5.16E-05	9.04E-05
CMTM7	3.240692295	Up	6.76E-07	1.70E-06
CMTR2	1.384358773	Up	6.36E-05	0.000109238
CNBD2	1.384158746	Up	1.30E-08	4.22E-08
CNGA2	2.419662436	Up	5.36E-06	1.15E-05
CNNM2	2.022326938	Up	1.37E-05	2.72E-05
CNOT1	1.03276754	Up	1.22E-06	2.94E-06
CNOT10	3.249737434	Up	6.04E-07	1.54E-06
CNOT4	1.78687141	Up	3.73E-09	1.30E-08
CNOT6	4.004624937	Up	1.05E-08	3.47E-08
CNOT8	6.780026712	Up	4.67E-15	2.74E-14
CNR1	3.057092357	Up	5.63E-06	1.21E-05
CNTFR	3.947677473	Up	4.34E-19	3.24E-18
CNTN1	2.497664948	Up	0.00076148	0.000997826
CNTROB	6.768744582	Up	5.84E-15	3.40E-14
COBL	4.642054857	Up	0.000212366	0.000320567
COCH	7.542197118	Up	2.60E-23	2.37E-22
COL1A2	3.153739751	Up	0.000100836	0.000164753
COL21A1	2.238437535	Up	6.15E-06	1.31E-05
COL4A3	1.236377543	Up	0.000265873	0.000391598
BP				
COL4A4	4.907663207	Up	4.13E-05	7.37E-05
COL6A2	2.961864365	Up	1.30E-10	5.31E-10
COL6A3	2.115066039	Up	0.000626165	0.000840693
COLCA1	3.217987117	Up	0.000634844	0.000851435
COLCA2	2.468887789	Up	1.02E-05	2.07E-05
COLEC11	4.535139653	Up	0.000382164	0.000541831
COLQ	5.081108022	Up	1.22E-05	2.43E-05
COMMD	1.056691797	Up	4.32E-06	9.46E-06

3				
COMMD 9	5.684699195	Up	5.51E-08	1.64E-07
COMT	2.097734341	Up	0.000659832	0.000881714
COPA	1.125931233	Up	2.06E-05	3.94E-05
COPRS	4.082627449	Up	2.90E-05	5.37E-05
COPS3	2.29722636	Up	5.30E-09	1.81E-08
COPS5	3.834699935	Up	0.000168506	0.000260719
COPS7A	2.175547012	Up	0.000343558	0.000493077
COPS7B	1.244090871	Up	1.05E-05	2.13E-05
COPZ1	5.834699935	Up	1.04E-08	3.43E-08
CORIN	2.011363366	Up	1.62E-14	9.15E-14
CORO2B	4.498044555	Up	0.000464429	0.000646509
CORO7	1.74513586	Up	2.05E-10	8.19E-10
cox11	4.612908512	Up	0.00025022	0.000372076
COX6B2	1.256597413	Up	3.63E-08	1.11E-07
CPA5	6.358261891	Up	8.48E-12	3.89E-11
CPEB1	2.156085562	Up	2.49E-07	6.76E-07
CPEB2	6.413151254	Up	8.81E-83	2.97E-81
cpeb3	1.554882771	Up	2.74E-47	5.15E-46
CPLX2	1.876495129	Up	4.98E-14	2.72E-13
CPLX3	2.294131554	Up	0.000567443	0.000768838
CPNE1	1.172494436	Up	4.41E-08	1.34E-07
CPNE9	3.358261891	Up	1.30E-09	4.78E-09
CPS1	1.337341193	Up	3.01E-06	6.78E-06
CPSF6	1.18833689	Up	1.87E-14	1.05E-13
CPSF7	5.535139653	Up	2.53E-07	6.84E-07
CPT1C	5.542335155	Up	2.36E-07	6.41E-07
cpt2	1.48870408	Up	1.42E-08	4.59E-08
CRAT	6.66581713	Up	4.27E-14	2.34E-13
CRBN	4.928936005	Up	3.58E-05	6.51E-05
CREB1	2.389095982	Up	1.49E-44	2.65E-43
creld1	5.642054857	Up	8.63E-08	2.50E-07
CRELD2	2.825511773	Up	2.68E-08	8.35E-08
CREM	5.441734281	Up	6.12E-07	1.55E-06
CRIP2	1.32313203	Up	1.39E-11	6.25E-11
CRISP1	4.419662436	Up	0.000691126	0.00091553
CRISP2	1.997170491	Up	8.12E-75	2.47E-73
CRLS1	1.640506735	Up	5.92E-05	0.000102285
CRTC3	3.073091237	Up	1.85E-12	8.98E-12
CRYBG3	1.019124506	Up	4.67E-05	8.25E-05
CSDE1	1.924875885	Up	1.79E-05	3.47E-05
CSE1L	1.001950802	Up	1.15E-20	9.33E-20
CSF1	3.381285764	Up	1.45E-05	2.85E-05

CSMD3	1.470543605	Up	1.15E-14	6.55E-14
CSNK1G1	1.147357487	Up	1.58E-08	5.07E-08
CSNK2A1	2.266749002	Up	0.000590927	0.000798415
CSNK2A2	2.055832095	Up	8.20E-58	1.90E-56
CSNK2A3	1.864128546	Up	6.65E-06	1.41E-05
CSPP1	1.2341445	Up	1.13E-12	5.59E-12
CSRP1	3.293796082	Up	5.96E-09	2.02E-08
CST8	5.649729673	Up	2.71E-11	1.19E-10
CTAGE1	1.13050069	Up	1.39E-21	1.17E-20
CTAGE6	1.384552134	Up	7.22E-06	1.51E-05
CTBP1	2.213211558	Up	2.17E-07	5.93E-07
CTBP2	4.971383513	Up	2.67E-05	4.99E-05
CTNNA1	1.348371142	Up	5.38E-06	1.16E-05
CTNNA2	1.295316358	Up	2.52E-19	1.91E-18
CTNNB1	5.180238245	Up	5.70E-06	1.22E-05
CTNNBIP1	5.473353052	Up	4.56E-07	1.18E-06
CTNND1	1.593691836	Up	0.000131764	0.000209551
CTNND2	1.663296005	Up	0.00020601	0.000313106
CTSB	5.135555806	Up	8.07E-06	1.68E-05
CTSF	1.110074767	Up	1.80E-05	3.48E-05
CTSS	2.996587618	Up	0.000323717	0.000467275
CTTN	4.550593306	Up	0.000351885	0.000504237
CTTNBP2	2.294131554	Up	0.000567443	0.000768838
CUL2	7.051736728	Up	1.31E-17	9.07E-17
CUL9	1.09660484	Up	2.35E-07	6.40E-07
CUX1	2.01655474	Up	9.71E-40	1.54E-38
CWF19L1	5.082627449	Up	1.20E-05	2.40E-05
CXCL16	1.061531775	Up	2.33E-06	5.35E-06
CXCR4	5.294131554	Up	2.25E-06	5.18E-06
CXorf66	2.10771843	Up	0.000173302	0.00026746
CXXC5	1.69748006	Up	0.000602521	0.000812728
CYB561	5.270328529	Up	2.75E-06	6.25E-06
CYB561A3	1.361089432	Up	1.86E-12	8.99E-12
CYB561D1	4.513898506	Up	0.000427534	0.000600017
CYB561D2	2.203933745	Up	6.54E-05	0.000112114
CYB5R2	3.127217143	Up	5.49E-25	5.37E-24

cyb5r3	4.673651702	Up	0.000177175	0.000272766
CYB5R4	1.041150813	Up	3.88E-07	1.02E-06
Cyc1	4.376957985	Up	2.39E-06	5.49E-06
cyfip2	5.589587437	Up	1.47E-07	4.13E-07
CYHR1	3.683584927	Up	5.28E-07	1.35E-06
CYP4F8	3.004624937	Up	0.000305949	0.000444199
cys1	1.389288787	Up	4.49E-07	1.16E-06
DAAM1	4.642054857	Up	0.000212366	0.000320567
DAB2	4.535139653	Up	0.000382164	0.000541831
DACT1	1.02373376	Up	1.28E-05	2.55E-05
DAG1	4.537357479	Up	0.000377682	0.000538023
DALRD3	1.87660627	Up	5.76E-06	1.23E-05
DAP3	3.408785847	Up	2.12E-06	4.91E-06
DAXX	1.049567447	Up	8.63E-05	0.000143334
DCAF1	9.12876923	Up	1.42E-56	3.21E-55
DCAF16	2.246530732	Up	7.15E-05	0.000121237
DCAF4L2	1.885326008	Up	0.000481545	0.000666568
DCDC2	4.082627449	Up	2.90E-05	5.37E-05
DCDC5	6.07692133	Up	5.05E-10	1.93E-09
DCP1A	2.760057279	Up	4.80E-06	1.04E-05
DCTN1	4.187317234	Up	1.26E-05	2.51E-05
DCTN2	7.00902645	Up	3.51E-17	2.36E-16
DCTN6	2.137262705	Up	1.44E-09	5.24E-09
DDAH2	1.600166816	Up	9.80E-08	2.82E-07
DDI2	2.419662436	Up	5.36E-06	1.15E-05
ddr1	1.589094675	Up	0.000263313	0.000389591
DDTL	1.828186628	Up	5.92E-07	1.51E-06
DDX11	6.389288787	Up	5.18E-12	2.42E-11
DDX19B	2.145801	Up	0.000279281	0.000409929
DDX21	5.984447055	Up	1.69E-09	6.11E-09
DDX39B	4.922162776	Up	3.75E-05	6.77E-05
DDX3Y	1.402807216	Up	4.60E-45	8.30E-44
DDX4	1.233475353	Up	3.94E-23	3.57E-22
DDX42	6.717342985	Up	1.60E-14	9.05E-14
DDX46	4.434452842	Up	3.24E-06	7.26E-06
DDX50	1.65412769	Up	8.88E-11	3.68E-10
DENND1A	1.982922771	Up	1.58E-28	1.79E-27
DENND2C	7.055803661	Up	1.19E-17	8.26E-17
DENND2D	1.200349407	Up	0.000142399	0.000223916
DENND4C	1.301017939	Up	8.53E-05	0.000141857

DENND5B	6.834699935	Up	1.54E-15	9.31E-15
DEPDC5	5.601825012	Up	1.30E-07	3.68E-07
DEPDC7	2.090825972	Up	3.26E-06	7.29E-06
DERL2	4.741590531	Up	0.000118566	0.000190485
DERL3	2.491965967	Up	4.19E-08	1.27E-07
DEUP1	2.71384554	Up	2.34E-16	1.50E-15
DFFB	2.137159937	Up	1.24E-08	4.07E-08
DGAT2	1.499297167	Up	1.09E-09	4.02E-09
DGCR6L	1.236031198	Up	9.96E-20	7.70E-19
DGCR8	1.20769299	Up	2.01E-16	1.29E-15
DGKH	4.401515089	Up	0.000755692	0.00099181
DGKI	3.419662436	Up	1.01E-05	2.06E-05
DHDDS	2.13427109	Up	9.07E-57	2.06E-55
DHFR	4.419662436	Up	0.000691126	0.00091553
DHFR2	2.15662803	Up	0.000404271	0.000570604
DHPS	3.922162776	Up	9.36E-05	0.000153908
DHRS4	4.612908512	Up	0.00025022	0.000372076
DHX29	1.08045635	Up	1.59E-11	7.11E-11
DHX36	1.166252449	Up	1.31E-35	1.83E-34
DHX8	1.240107699	Up	5.43E-16	3.39E-15
DHX9	1.140803063	Up	1.99E-19	1.51E-18
DIAPH2	1.593691836	Up	0.000131764	0.000209551
DICER1	1.355780041	Up	4.20E-40	6.70E-39
DIO2	7.197695747	Up	3.80E-19	2.84E-18
DIP2A	5.292491195	Up	2.32E-11	1.02E-10
DIP2B	1.003653098	Up	9.63E-15	5.53E-14
DIRAS3	4.419662436	Up	1.60E-06	3.77E-06
dis3	5.227704192	Up	3.90E-06	8.62E-06
DIS3L	1.788896246	Up	0.000497148	0.000686416
DIS3L2	1.008999185	Up	1.60E-06	3.77E-06
DIXDC1	4.535139653	Up	0.000382164	0.000541831
DKKL1	2.324284388	Up	1.89E-05	3.64E-05
DLG1	1.416698501	Up	1.30E-26	1.37E-25
DLK1	5.082627449	Up	1.20E-05	2.40E-05
DLL4	1.824636252	Up	3.78E-12	1.79E-11
DMBT1	1.138167587	Up	0.000213108	0.00032161
DMC1	5.101486476	Up	1.04E-05	2.12E-05
DMKN	3.758078654	Up	1.18E-05	2.37E-05
DMTF1	2.241142153	Up	2.06E-10	8.25E-10
DMTN	2.460053192	Up	5.27E-05	9.20E-05
DNAAF2	5.69268093	Up	5.06E-08	1.52E-07
DNAAF3	9.552376358	Up	2.99E-71	8.63E-70
dnah14	1.707723105	Up	0.00013112	0.000208663

DNAH6	2.055576124	Up	2.50E-34	3.38E-33
DNAH8	1.144027993	Up	1.79E-06	4.20E-06
DNAI1	2.051017842	Up	1.13E-09	4.16E-09
DNAJA1	1.301825946	Up	4.09E-09	1.42E-08
DNAJA3	6.824066049	Up	1.91E-15	1.15E-14
DNAJB12	1.994920743	Up	5.52E-13	2.78E-12
DNAJB2	1.157085956	Up	5.43E-10	2.07E-09
DNAJC22	1.410205157	Up	3.97E-07	1.04E-06
DNAJC4	1.713001894	Up	9.35E-07	2.29E-06
DNAJC5	1.227017358	Up	0.000174569	0.000269
DNAJC6	2.353628021	Up	1.06E-09	3.93E-09
DNAJC7	1.469456799	Up	5.55E-05	9.65E-05
DNASE1	1.063483866	Up	0.000685549	0.00091339
dnase2	5.082627449	Up	1.20E-05	2.40E-05
DNM2	7.157889836	Up	1.03E-18	7.51E-18
DNM3	4.666534962	Up	0.00024865	0.00036997
DNMT1	1.475400226	Up	2.87E-30	3.43E-29
DOCK1	1.020796227	Up	3.25E-07	8.63E-07
DOK7	5.600234682	Up	1.32E-07	3.74E-07
DONSON	3.284465436	Up	1.60E-191	1.29E-189
DPF3	5.176685682	Up	5.86E-06	1.25E-05
DPH3	2.054131934	Up	2.85E-11	1.24E-10
DPM1	1.258633703	Up	2.81E-05	5.23E-05
DPP3	1.254548668	Up	0.000663005	0.000885684
DPP6	2.265541625	Up	7.37E-08	2.16E-07
DPP8	5.811063571	Up	1.37E-08	4.44E-08
DPYD	5.15662803	Up	6.86E-06	1.44E-05
dqx1	3.294131554	Up	3.45E-07	9.12E-07
DRC7	5.139940901	Up	7.80E-06	1.62E-05
DROSHA	2.185197182	Up	3.51E-07	9.26E-07
dsn1	4.431635078	Up	0.000651219	0.000871824
DTNA	7.229248374	Up	1.70E-19	1.30E-18
DTWD1	1.697196411	Up	0.000137314	0.00021755
dus2	6.963982952	Up	9.66E-17	6.32E-16
DUSP15	1.369014667	Up	2.89E-20	2.29E-19
DUSP21	1.684162251	Up	3.95E-48	7.57E-47
DVL1	3.082627449	Up	0.000173799	0.000267992
DYNC2H1	2.088624429	Up	3.28E-23	2.97E-22
DYNC2LI1	4.676673054	Up	0.0001741	0.00026838
DYNLT1	5.15662803	Up	6.86E-06	1.44E-05
DYRK1A	2.064730541	Up	1.17E-06	2.84E-06
DZANK1	1.163208178	Up	2.14E-05	4.07E-05

DZIP1	7.920252062	Up	6.93E-29	7.92E-28
E4F1	5.019773573	Up	1.90E-05	3.67E-05
EBF1	2.137438333	Up	5.51E-05	9.60E-05
ecd	6.437287463	Up	2.37E-12	1.14E-11
ECSIT	3.699628907	Up	0.000391721	0.000554405
EDC4	1.004624937	Up	0.000639652	0.000857228
EDEM3	1.174928847	Up	6.22E-07	1.58E-06
EDNRA	4.90423026	Up	4.22E-05	7.53E-05
EDNRB	7.120102154	Up	2.59E-18	1.85E-17
EEF1D	1.341235789	Up	0.000699115	0.000925192
EEFSEC	1.535139653	Up	1.99E-11	8.82E-11
EFCAB1	1.692407563	Up	1.24E-05	2.47E-05
EFCAB11	4.404555543	Up	1.85E-06	4.31E-06
Efcab13	1.589970926	Up	5.35E-08	1.60E-07
EFCAB5	1.284784382	Up	4.24E-08	1.29E-07
EFCAB6	1.317003717	Up	2.28E-09	8.12E-09
EFHD1	3.834699935	Up	0.000168506	0.000260719
EFTUD2	2.111381405	Up	9.27E-10	3.46E-09
EGLN2	1.137054962	Up	7.87E-05	0.000131907
EGR3	4.561078774	Up	1.48E-13	7.80E-13
EHBP1	1.089616508	Up	2.79E-14	1.55E-13
EHD1	7.183307664	Up	1.31E-52	2.78E-51
EHMT2	7.228562275	Up	1.73E-19	1.32E-18
EIF1	1.741573116	Up	1.00E-18	7.33E-18
EIF2B4	5.082627449	Up	1.20E-05	2.40E-05
EIF3CL	6.132258216	Up	2.38E-10	9.46E-10
EIF3G	1.108002676	Up	0.000335769	0.000483052
EIF3J	3.61815659	Up	0.000628729	0.000843878
EIF3L	4.419662436	Up	0.000691126	0.00091553
EIF3M	4.741590531	Up	0.000118566	0.000190485
EIF4A1	1.520838468	Up	1.56E-10	6.30E-10
EIF4E2	1.159369289	Up	6.11E-06	1.30E-05
EIF4ENIF1	1.536022386	Up	3.92E-28	4.37E-27
EIF4G1	1.473363456	Up	7.70E-06	1.60E-05
EIF4G2	8.246888682	Up	1.04E-34	1.42E-33
EIF4G3	3.288086597	Up	9.43E-40	1.49E-38
EIF5AL1	2.250775185	Up	3.51E-05	6.39E-05
EIF5B	3.337200276	Up	1.97E-07	5.41E-07
ELAVL2	1.051022677	Up	9.16E-05	0.000151035
ELAVL4	7.222717221	Up	2.01E-19	1.53E-18
ELF1	6.719053642	Up	1.55E-14	8.77E-14
ELF2	5.834699935	Up	1.04E-08	3.43E-08
ELK1	4.834699935	Up	6.65E-05	0.000113504

ELK4	1.209170485	Up	6.63E-29	7.59E-28
ELMOD1	4.875592366	Up	5.10E-05	8.96E-05
ELOF1	2.535139653	Up	6.08E-50	1.21E-48
ELOVL5	5.213406926	Up	9.97E-15	5.72E-14
ELP2	1.460885099	Up	5.23E-06	1.13E-05
ELP5	1.442941448	Up	2.92E-06	6.60E-06
EMC10	5.278969438	Up	2.56E-06	5.84E-06
EMC6	5.642054857	Up	8.63E-08	2.50E-07
EMC8	1.752237775	Up	0.000324483	0.000468119
EML2	1.330896992	Up	1.63E-07	4.53E-07
EML3	1.390063551	Up	0.000138458	0.00021822
EML5	2.243978165	Up	4.53E-06	9.88E-06
EMSY	4.017001334	Up	2.67E-08	8.30E-08
ENAH	2.774076935	Up	7.31E-38	1.10E-36
ENAM	1.088193574	Up	1.10E-18	8.01E-18
ENC1	10.0132403	Up	2.64E-91	9.90E-90
ENGASE	2.120102154	Up	6.36E-10	2.41E-09
ENKUR	4.478556125	Up	0.000513605	0.000704242
ENOSF1	1.723199671	Up	1.57E-07	4.39E-07
ENTHD1	3.004624937	Up	0.000305949	0.000444199
entpd3	4.081108022	Up	2.93E-05	5.43E-05
EOGT	1.706543584	Up	0.000250782	0.000372874
EPB41	5.73180951	Up	2.02E-06	4.68E-06
EPB41L2	1.781900181	Up	3.96E-20	3.13E-19
EPB41L5	6.318193286	Up	1.58E-11	7.07E-11
EPM2AIP1	2.249737434	Up	4.87E-07	1.26E-06
EPOR	1.580127108	Up	1.53E-05	3.00E-05
EQTN	1.041922761	Up	0.000438565	0.000614246
ercc1	5.213211558	Up	4.38E-06	9.59E-06
ERCC6	2.598642596	Up	3.86E-05	6.94E-05
ERCC6L2	1.551699495	Up	0.000265559	0.000391187
ERG	5.963982952	Up	2.18E-09	7.80E-09
ERGIC1	1.342178968	Up	8.60E-05	0.000142832
ERGIC2	3.834699935	Up	0.000168506	0.000260719
ERI3	6.174549938	Up	1.32E-10	5.37E-10
ERICH3	1.95902807	Up	3.84E-12	1.81E-11
ERLEC1	4.922162776	Up	3.75E-05	6.77E-05
ERLIN1	1.058784322	Up	3.98E-09	1.38E-08
ERRFI1	2.659593306	Up	2.69E-07	7.26E-07
ERVW-1	1.562928515	Up	0.000758247	0.000994926
ESAM	6.493482669	Up	9.27E-13	4.59E-12
ESR2	3.501015778	Up	2.89E-12	1.38E-11
ESRRA	2.637938749	Up	2.51E-08	7.84E-08

ETFDH	4.535139653	Up	0.000382164	0.000541831
ETNK1	3.055429307	Up	1.32E-06	3.17E-06
ETNK2	6.231818344	Up	5.78E-11	2.45E-10
ETV1	1.506589192	Up	1.15E-09	4.26E-09
ETV4	1.415412963	Up	0.000166723	0.000258535
EVA1C	7.15662803	Up	1.06E-18	7.74E-18
EVL	1.722395714	Up	1.42E-05	2.81E-05
EVX1	1.723721243	Up	5.64E-52	1.17E-50
EXOC6	1.950177153	Up	1.74E-06	4.09E-06
EXOSC10	2.15662803	Up	7.66E-06	1.60E-05
EXPH5	4.477401507	Up	0.000516657	0.000707972
EYA1	4.823838943	Up	7.13E-05	0.000120932
EZH2	1.963752655	Up	3.08E-05	5.67E-05
F11R	1.69727624	Up	8.38E-28	9.20E-27
F2R	4.770159683	Up	9.96E-05	0.000162911
f2rl2	6.943224392	Up	1.53E-16	9.89E-16
FAAP20	4.510515866	Up	0.000435181	0.000609805
FABP3	4.15662803	Up	1.62E-05	3.16E-05
FAHD1	2.506391392	Up	2.66E-07	7.18E-07
FAM102A	2.46576595	Up	8.20E-05	0.00013685
FAM107A	6.589587437	Up	1.73E-13	9.07E-13
FAM109A	6.703584208	Up	2.08E-14	1.17E-13
FAM114A2	5.048047217	Up	1.55E-05	3.04E-05
FAM117B	1.316115396	Up	3.11E-08	9.60E-08
FAM135A	1.038681391	Up	0.000403064	0.000569165
fam135b	1.454668886	Up	3.29E-20	2.61E-19
FAM13A	7.370441876	Up	3.89E-21	3.22E-20
FAM155B	4.535139653	Up	0.000382164	0.000541831
FAM160B1	1.26288062	Up	2.69E-05	5.01E-05
FAM169A	2.26096469	Up	1.78E-06	4.18E-06
FAM169B	5.004624937	Up	2.12E-05	4.03E-05
FAM188B	4.478556125	Up	9.00E-07	2.21E-06
FAM189B	5.227017358	Up	3.92E-06	8.66E-06
FAM192A	1.681926583	Up	5.92E-05	0.000102376
FAM19A1	4.741590531	Up	0.000118566	0.000190485
FAM20A	2.381079974	Up	8.67E-06	1.79E-05
FAM20B	1.508111735	Up	0.000245127	0.000365407
FAM210A	2.15662803	Up	0.000404271	0.000570604
FAM212B	3.266143689	Up	2.47E-12	1.19E-11
FAM214B	1.447081425	Up	4.04E-46	7.42E-45
FAM217A	2.114807854	Up	1.70E-12	8.25E-12
FAM217B	5.611330358	Up	1.18E-07	3.36E-07

FAM219A	7.042609265	Up	1.28E-121	6.46E-120
FAM222B	4.462306773	Up	0.00055805	0.000757797
FAM234A	1.131681673	Up	3.93E-05	7.05E-05
FAM46A	1.885326008	Up	0.000481545	0.000666568
FAM47C	3.741590531	Up	0.000303865	0.000442212
FAM53A	1.144534113	Up	0.000728612	0.000960135
FAM60A	6.143220997	Up	2.05E-10	8.19E-10
FAM65A	1.070426995	Up	1.62E-28	1.83E-27
FAM65B	4.411222073	Up	0.000720529	0.000951236
FAM65C	5.91450469	Up	4.02E-09	1.39E-08
FAM71F1	1.086931633	Up	4.83E-65	1.27E-63
FAM71F2	2.014023635	Up	0.000171044	0.000264391
FAM72A	2.340830631	Up	2.02E-09	7.26E-09
FAM76A	2.883609536	Up	0.000106215	0.000172911
FAM84B	1.084059404	Up	1.53E-06	3.63E-06
FAM89B	3.578861031	Up	5.89E-14	3.20E-13
FAM92A	1.465234384	Up	8.14E-23	7.28E-22
FANCC	1.655771801	Up	9.12E-07	2.24E-06
FANCD2	2.304585911	Up	1.96E-07	5.40E-07
FANCD2 OS	5.099986793	Up	1.06E-05	2.15E-05
FANCL	1.600234682	Up	3.66E-05	6.64E-05
fancm	1.304007097	Up	2.21E-16	1.42E-15
FARP1	2.935437354	Up	7.03E-11	2.95E-10
FASLG	4.419662436	Up	0.000691126	0.00091553
FAXDC2	1.197644789	Up	3.92E-06	8.66E-06
FBLIM1	1.393822431	Up	2.68E-05	5.01E-05
FBXL13	2.135897348	Up	1.48E-09	5.40E-09
FBXL20	1.121547662	Up	4.08E-08	1.24E-07
FBXL5	1.958488044	Up	1.17E-12	5.74E-12
FBXO17	1.514744982	Up	7.41E-10	2.79E-09
FBXO22	2.141434356	Up	7.08E-39	1.09E-37
FBXO28	5.788896246	Up	1.76E-08	5.61E-08
FBXO3	1.412906446	Up	0.000223656	0.000335912
fbxo34	4.22259191	Up	1.02E-29	1.20E-28
FBXO9	5.294131554	Up	2.25E-06	5.18E-06
FBXW10	2.869514706	Up	6.79E-07	1.71E-06
FBXW11	1.697196411	Up	0.000137314	0.00021755
FBXW8	1.39763613	Up	2.49E-05	4.67E-05
FCAR	5.59811151	Up	1.35E-07	3.81E-07
FCGR2A	2.403394694	Up	1.03E-52	2.18E-51
FDFT1	2.615637266	Up	9.03E-07	2.22E-06
FERMT2	5.190139132	Up	5.27E-06	1.14E-05
FES	4.227017358	Up	9.05E-06	1.86E-05

FFAR3	2.571665529	Up	6.86E-07	1.73E-06
FGF11	1.3915488	Up	1.63E-08	5.24E-08
FGF14	5.478556125	Up	4.35E-07	1.13E-06
FGF17	6.004624937	Up	1.30E-09	4.78E-09
FGF7	3.456458792	Up	7.08E-06	1.48E-05
FGFR1	5.403947965	Up	8.64E-07	2.14E-06
FGFR1OP	1.026650319	Up	2.16E-10	8.64E-10
FGFR3	3.319516872	Up	0.000107914	0.000175345
FGGY	4.535139653	Up	0.000382164	0.000541831
FHOD3	4.941550732	Up	3.28E-05	6.01E-05
FIBP	4.642054857	Up	0.000212366	0.000320567
filip1l	3.244020702	Up	3.86E-80	1.25E-78
FIP1L1	1.911742584	Up	2.54E-19	1.92E-18
FKBP1A	2.605727365	Up	0.000405512	0.000572152
FKBP3	2.571665529	Up	0.000446586	0.000623907
FKBP7	7.865137178	Up	3.51E-52	7.33E-51
FLCN	1.599075866	Up	7.09E-05	0.000120412
FLT3	1.011022708	Up	1.71E-05	3.32E-05
FMNL3	8.366081396	Up	4.19E-37	6.13E-36
FMO5	5.855735415	Up	8.13E-09	2.72E-08
FNDC3A	2.221904899	Up	9.23E-08	2.66E-07
FNDC3B	1.356534457	Up	1.52E-19	1.16E-18
FNDC5	1.943577407	Up	5.26E-81	1.73E-79
FNIP1	6.326553031	Up	1.39E-11	6.25E-11
FNIP2	1.062110431	Up	3.68E-05	6.68E-05
FNTA	2.642054857	Up	0.00026095	0.000386382
FOCAD	1.419662436	Up	7.84E-10	2.95E-09
FOSB	8.195415361	Up	1.01E-33	1.34E-32
FOXJ3	8.049116204	Up	4.61E-31	5.66E-30
FOXK2	1.466902956	Up	1.49E-11	6.66E-11
FOXM1	7.123243068	Up	2.40E-18	1.72E-17
FOXN3	1.107931013	Up	3.81E-07	1.00E-06
FOXN4	3.004624937	Up	9.80E-06	2.00E-05
FOXO3	1.179670942	Up	6.22E-06	1.32E-05
FOXP1	4.704571341	Up	0.000147882	0.000231733
FPR2	1.719222718	Up	2.50E-05	4.69E-05
FRAS1	1.528961078	Up	5.51E-11	2.34E-10
FRG2C	1.960230817	Up	4.04E-06	8.90E-06
FRMD3	5.14212846	Up	7.67E-06	1.60E-05
FRMD4A	2.8265086	Up	2.08E-18	1.50E-17
FRMD8	5.088689201	Up	1.15E-05	2.31E-05
FRMPD2	5.36514529	Up	1.22E-06	2.94E-06
FRYL	1.320126762	Up	4.47E-06	9.77E-06
fsd1l	2.467917604	Up	6.01E-05	0.000103816

FSD2	6.615797317	Up	1.08E-13	5.74E-13
FSIP2	1.607289439	Up	1.26E-10	5.16E-10
FUT11	1.553994503	Up	2.36E-14	1.32E-13
FUT7	2.419662436	Up	0.000202995	0.000308875
FUT8	8.471324556	Up	2.39E-39	3.74E-38
FXR1	1.416404561	Up	2.63E-11	1.15E-10
FXYD6-FXYD2	4.408801443	Up	0.000729161	0.000960725
FYB	2.709169053	Up	0.000151978	0.00023744
FYTTD1	2.707856916	Up	0.000153635	0.000239815
FZD4	1.914618407	Up	2.74E-08	8.51E-08
FZD6	1.039182159	Up	0.000308614	0.000447609
FZR1	5.741590531	Up	2.98E-08	9.22E-08
G3BP1	2.885326008	Up	2.11E-10	8.42E-10
GABARAP	2.125290064	Up	3.19E-65	8.40E-64
GABARAPL1	1.525528275	Up	4.35E-80	1.41E-78
GABBR1	7.857067748	Up	7.14E-28	7.87E-27
GABRB3	4.834699935	Up	6.65E-05	0.000113504
GALNT5	1.118908364	Up	6.71E-08	1.98E-07
GAN	2.642054857	Up	2.62E-11	1.15E-10
GANAB	1.960453438	Up	8.49E-08	2.47E-07
GANC	1.941777158	Up	0.000538709	0.000734807
GAPDH	1.479255444	Up	0.000455585	0.000635124
GAPVD1	7.27581818	Up	5.05E-20	3.96E-19
GARS	4.741590531	Up	0.000118566	0.000190485
GART	5.589587437	Up	1.47E-07	4.13E-07
GAS2	1.36618537	Up	1.86E-05	3.59E-05
GAS2L3	2.823838943	Up	1.23E-08	4.02E-08
GAS7	1.718506918	Up	3.67E-14	2.03E-13
GATA2	7.15662803	Up	1.06E-18	7.74E-18
GATA4	2.138080222	Up	8.04E-13	4.00E-12
GBA	6.306537698	Up	1.89E-11	8.40E-11
GBAS	3.642054857	Up	2.82E-51	5.76E-50
GBF1	1.572254883	Up	5.12E-22	4.41E-21
gbp1	3.922162776	Up	9.36E-05	0.000153908
GBP5	5.365769428	Up	1.21E-06	2.93E-06
GBX2	6.15662803	Up	1.70E-10	6.83E-10
GCAT	4.769216437	Up	0.000100213	0.000163778
GCC1	1.112233911	Up	9.20E-05	0.000151689
GCK	5.358261891	Up	1.30E-06	3.11E-06
GCN1	1.294131554	Up	3.76E-06	8.33E-06
GCNT1	4.642054857	Up	0.000212366	0.000320567

GCNT7	1.627257855	Up	8.29E-09	2.77E-08
GDAP2	3.628061585	Up	2.94E-05	5.44E-05
GDI1	4.358261891	Up	2.84E-06	6.45E-06
GDPD1	4.092812932	Up	1.85E-05	3.57E-05
GDPD3	2.358261891	Up	4.05E-07	1.06E-06
GEN1	1.895582177	Up	8.27E-13	4.11E-12
GFPT1	5.443509178	Up	6.03E-07	1.53E-06
GFRA2	4.174244573	Up	1.46E-30	1.76E-29
Gfy	1.682696842	Up	0.000323884	0.000467362
GGA1	6.43491021	Up	2.47E-12	1.19E-11
GGA3	1.191791573	Up	1.85E-07	5.11E-07
GID8	2.249737434	Up	3.99E-05	7.15E-05
GINM1	1.870323845	Up	3.65E-05	6.63E-05
GINS3	4.082627449	Up	2.90E-05	5.37E-05
GIT2	2.294131554	Up	1.09E-06	2.65E-06
GJA10	5.69268093	Up	5.06E-08	1.52E-07
GK5	5.26297635	Up	2.92E-06	6.60E-06
GKAP1	1.254231265	Up	2.62E-06	5.97E-06
GLB1	2.521200462	Up	3.28E-15	1.94E-14
glb1l	3.188023226	Up	1.28E-06	3.08E-06
GLE1	1.038910602	Up	1.01E-12	5.02E-12
GLG1	1.51133346	Up	1.45E-14	8.22E-14
GLIPR1	3.834699935	Up	0.000168506	0.000260719
GLO1	5.004624937	Up	2.12E-05	4.03E-05
GLRX3	1.37311985	Up	5.59E-05	9.71E-05
GLT8D1	8.563640804	Up	2.02E-41	3.33E-40
GLYCTK	6.512208177	Up	6.73E-13	3.37E-12
GMPR2	2.102084877	Up	5.40E-29	6.19E-28
GNA12	1.002294877	Up	0.000242381	0.000361608
GNAI2	2.144431483	Up	1.11E-05	2.23E-05
GNAS	5.357634496	Up	1.30E-06	3.12E-06
GNB1	2.555545864	Up	1.67E-25	1.68E-24
GNE	4.545093127	Up	0.000362409	0.000517863
GNG3	1.762161388	Up	3.38E-16	2.14E-15
GNG5	2.15662803	Up	0.00010667	0.000173549
GNL2	1.593691836	Up	0.000131764	0.000209551
GNL3L	4.779558381	Up	9.40E-05	0.000154506
GOLGA3	2.490360147	Up	3.07E-16	1.95E-15
GOLGA8B	2.463889895	Up	3.36E-39	5.25E-38
GOLGB1	2.008406577	Up	4.61E-13	2.34E-12
GOSR2	1.920996019	Up	1.98E-26	2.06E-25
GOT2	1.012617727	Up	5.07E-08	1.52E-07
GP1BA	2.004624937	Up	0.000451229	0.000629432

GP6	1.566503824	Up	4.04E-08	1.23E-07
GPANK1	1.228750778	Up	3.61E-06	8.02E-06
GPATCH4	3.642054857	Up	1.10E-14	6.27E-14
GPBP1	2.261635555	Up	0.000727991	0.000959356
GPBP1L1	3.796423367	Up	6.76E-06	1.43E-05
GPR108	2.834699935	Up	8.76E-15	5.04E-14
GPR137	7.469873882	Up	2.27E-22	1.99E-21
GPR151	4.642054857	Up	0.000212366	0.000320567
GPR161	2.208941176	Up	2.27E-07	6.20E-07
GPR162	1.343340152	Up	2.29E-07	6.25E-07
GPR173	2.091936496	Up	0.000325718	0.000469762
GPR18	1.776929715	Up	6.54E-53	1.39E-51
GPR22	2.004624937	Up	1.01E-08	3.34E-08
GPR55	5.642054857	Up	8.63E-08	2.50E-07
GPR68	1.101486476	Up	0.000140796	0.000221629
GPR85	7.076540119	Up	7.34E-18	5.14E-17
GPR89B	5.69268093	Up	5.06E-08	1.52E-07
GPRC5B	6.875154057	Up	6.61E-16	4.10E-15
GPS2	1.343011941	Up	4.44E-14	2.43E-13
GPX2	1.858546677	Up	5.33E-07	1.36E-06
GRAMD1C	3.419662436	Up	1.01E-05	2.06E-05
GRAP2	5.576706146	Up	1.68E-07	4.66E-07
GRB10	2.08419073	Up	3.01E-12	1.43E-11
GRB2	5.082627449	Up	1.20E-05	2.40E-05
GREM1	4.834699935	Up	6.65E-05	0.000113504
GRHPR	1.318217172	Up	3.63E-18	2.57E-17
GRIA4	1.954994169	Up	3.11E-11	1.35E-10
GRIN1	2.486297188	Up	1.41E-15	8.54E-15
GRK4	4.535139653	Up	0.000382164	0.000541831
GRK6	3.28877169	Up	6.90E-17	4.56E-16
GRK7	5.227017358	Up	3.92E-06	8.66E-06
GRM1	1.405856636	Up	8.48E-08	2.46E-07
GRM4	6.69268093	Up	2.57E-14	1.43E-13
GRM7	2.535139653	Up	1.15E-06	2.79E-06
GS1-259H13.2	1.049990241	Up	0.000322459	0.000465583
GSAP	4.642054857	Up	0.000212366	0.000320567
GSDMB	2.830686873	Up	0.000464525	0.000646622
GSG1	1.0704599	Up	2.04E-13	1.07E-12
GSK3B	5.060437589	Up	1.42E-05	2.80E-05
GSTK1	1.158805457	Up	3.70E-10	1.44E-09
GSTM5	1.716130778	Up	7.17E-09	2.41E-08
GSTO1	4.004624937	Up	1.05E-08	3.47E-08

GSTO2	5.680691706	Up	5.75E-08	1.71E-07
GSTZ1	2.052273166	Up	3.96E-09	1.37E-08
GTF2F2	5.884766233	Up	5.76E-09	1.96E-08
GTF2H4	3.044153301	Up	1.91E-07	5.25E-07
GTF2I	7.031818315	Up	2.08E-17	1.42E-16
GTF2IRD2	1.438367179	Up	7.24E-19	5.33E-18
gtf2ird2b	1.635309154	Up	1.33E-13	7.06E-13
GTSF1L	2.298537197	Up	3.43E-24	3.25E-23
GUCY1A3	2.389592672	Up	2.13E-07	5.83E-07
GUK1	6.824520155	Up	1.89E-15	1.14E-14
GUSB	4.417255938	Up	0.0006994	0.000925408
GZF1	1.315535218	Up	1.42E-10	5.78E-10
H3F3B	1.050887984	Up	1.35E-227	1.29E-225
H6PD	1.137162637	Up	4.78E-09	1.64E-08
HABP2	2.337200276	Up	1.47E-05	2.90E-05
HADHB	2.320126762	Up	9.05E-05	0.000149424
HAGH	1.389143051	Up	8.85E-18	6.17E-17
HARS	4.928091088	Up	3.60E-05	6.54E-05
HAUS3	5.180238245	Up	5.70E-06	1.22E-05
HDAC11	1.488516626	Up	1.87E-21	1.57E-20
HDAC5	2.576571937	Up	1.70E-18	1.23E-17
HDGF	1.079951147	Up	4.97E-07	1.28E-06
HDGFRP2	2.956945841	Up	0.000162914	0.000253191
HDLBP	6.380977067	Up	5.91E-12	2.75E-11
HECW1	3.158584646	Up	6.18E-25	6.03E-24
HECW2	5.082627449	Up	1.20E-05	2.40E-05
HELZ	1.939837067	Up	9.50E-16	5.84E-15
HEMGN	1.016903266	Up	2.94E-12	1.40E-11
HERPUD1	2.212101366	Up	0.000248975	0.000370388
HEXA	1.834699935	Up	0.000758784	0.000995362
HEXIM2	5.127481684	Up	8.58E-06	1.77E-05
HGSNAT	2.642054857	Up	0.00026095	0.000386382
HIC1	5.15662803	Up	6.86E-06	1.44E-05
HINT1	5.294131554	Up	2.25E-06	5.18E-06
HIPK3	3.938186951	Up	9.75E-26	9.85E-25
hist1h2ba	4.741590531	Up	0.000118566	0.000190485
Hist1h2bj	1.305837909	Up	4.15E-06	9.12E-06
Hist1h2bk	1.399344135	Up	5.08E-05	8.92E-05
HIST3H3	2.834699935	Up	5.11E-05	8.96E-05
HIVEP2	1.419662436	Up	1.11E-05	2.23E-05

HIVEP3	1.453609768	Up	0.000412519	0.00058116
HJURP	6.217021342	Up	7.17E-11	3.00E-10
HK1	7.298387777	Up	2.77E-20	2.21E-19
HKR1	2.048824741	Up	1.64E-13	8.64E-13
HLA-DOB	6.082627449	Up	4.68E-10	1.80E-09
HMGB3	4.741590531	Up	0.000118566	0.000190485
HMGCL	1.3856006	Up	3.83E-06	8.47E-06
HMGCR	1.649024412	Up	2.38E-33	3.13E-32
HMGN2	1.465814456	Up	1.83E-06	4.28E-06
HMGXB4	1.219694155	Up	2.68E-06	6.10E-06
HMMR	5.349453434	Up	1.40E-06	3.34E-06
HMOX2	5.234554609	Up	3.69E-06	8.19E-06
HN1L	2.072830659	Up	3.22E-12	1.53E-11
HNF1B	5.478556125	Up	4.35E-07	1.13E-06
HNF4A	6.326553031	Up	1.39E-11	6.25E-11
HNRNPA2B1	1.130445891	Up	9.88E-08	2.84E-07
HNRNPAB	5.69268093	Up	5.06E-08	1.52E-07
HNRNPCL2	1.573239902	Up	7.64E-08	2.23E-07
HNRNPD	4.535139653	Up	5.08E-07	1.30E-06
HNRNPF	1.36888214	Up	1.56E-06	3.69E-06
HNRNPH1	1.135545429	Up	5.49E-34	7.36E-33
HNRNPL	1.054234748	Up	1.69E-08	5.42E-08
HNRNPUL1	1.029745837	Up	1.33E-07	3.76E-07
HOXA1	3.589587437	Up	1.81E-06	4.24E-06
HOXC4	4.929975224	Up	5.55E-26	5.66E-25
HOXC6	3.791287674	Up	1.04E-06	2.54E-06
HOXD9	3.082627449	Up	0.000173799	0.000267992
HPD	2.848325881	Up	4.15E-06	9.12E-06
HPS1	1.502157237	Up	6.89E-05	0.00011723
HPS4	4.563708806	Up	0.000327873	0.000472668
hps5	1.445292579	Up	7.33E-13	3.66E-12
HRAS	6.190139132	Up	1.06E-10	4.35E-10
HS2ST1	4.222216372	Up	4.87E-07	1.26E-06
hsd11b1l	1.651392722	Up	1.94E-07	5.33E-07
HSD17B4	4.834699935	Up	6.65E-05	0.000113504
hsf5	1.04620404	Up	5.94E-15	3.46E-14
HSP90AA1	4.289786844	Up	1.39E-60	3.39E-59

HSP90AB 1	1.298910071	Up	4.42E-29	5.09E-28
HSPA5	2.197270015	Up	1.75E-05	3.40E-05
HSPA8	1.844285984	Up	9.02E-06	1.86E-05
HSPD1	4.834699935	Up	1.69E-08	5.43E-08
HTR2A	1.355011246	Up	4.56E-07	1.18E-06
HYAL1	5.140670456	Up	7.76E-06	1.61E-05
hyal3	2.320221867	Up	3.63E-16	2.29E-15
HYDIN	1.793209726	Up	3.51E-08	1.08E-07
ICA1	5.60288426	Up	1.29E-07	3.65E-07
ICA1L	7.132991666	Up	1.89E-18	1.36E-17
ICOSLG	2.223086306	Up	1.28E-05	2.54E-05
ID1	2.193622238	Up	0.000499964	0.00069004
ID3	1.167275274	Up	0.000178197	0.000274196
IFNL4	3.038777459	Up	0.000125748	0.000201033
IFT122	1.33042528	Up	3.79E-05	6.85E-05
IFT20	1.400453659	Up	3.38E-05	6.18E-05
IFT46	1.119429412	Up	3.62E-07	9.55E-07
IFT52	5.879094055	Up	6.16E-09	2.09E-08
IGDCC4	1.465466126	Up	1.10E-07	3.14E-07
IGF1R	1.367045142	Up	6.20E-12	2.88E-11
IGF2BP1	1.243339663	Up	0.000214735	0.000323831
IGF2BP2	5.29084897	Up	2.31E-06	5.32E-06
IGFBP2	4.589587437	Up	2.87E-07	7.68E-07
IGFL2	5.80555359	Up	1.45E-08	4.70E-08
IGSF1	5.082627449	Up	1.20E-05	2.40E-05
IGSF10	3.014740281	Up	5.88E-07	1.50E-06
igsf11	2.662772933	Up	1.48E-17	1.02E-16
IK	1.241875317	Up	0.000192787	0.000294733
IKZF3	5.204864216	Up	4.69E-06	1.02E-05
IL16	4.711462426	Up	0.000141977	0.000223293
IL17RC	2.367524813	Up	0.00018123	0.000278364
IL19	1.497609748	Up	1.42E-30	1.72E-29
IL1A	1.874228299	Up	8.59E-05	0.000142662
IL4I1	6.541230488	Up	4.07E-13	2.07E-12
IL4R	4.836502178	Up	6.57E-05	0.00011265
IL6R	2.6834099	Up	4.12E-29	4.75E-28
il6st	5.888680103	Up	5.49E-09	1.88E-08
ILDR2	1.679725275	Up	7.69E-17	5.06E-16
ILF2	1.703455402	Up	3.03E-08	9.36E-08
ILK	1.351352572	Up	6.26E-09	2.12E-08
ILKAP	1.660670535	Up	0.000209433	0.000317582
IMPDH1	4.424463422	Up	0.000674873	0.000900279
IMPG1	3.419662436	Up	1.01E-05	2.06E-05

ING1	6.172767733	Up	1.35E-10	5.51E-10
INHA	2.097734341	Up	0.000659832	0.000881714
INHBC	1.263543234	Up	7.12E-07	1.78E-06
INO80C	4.358261891	Up	2.84E-06	6.45E-06
INO80E	5.114913931	Up	9.44E-06	1.93E-05
INPP1	1.857459838	Up	5.28E-13	2.67E-12
INPP4B	1.093434204	Up	7.93E-05	0.000132821
INPP5B	5.53180651	Up	2.61E-07	7.05E-07
INPP5F	2.809840948	Up	6.40E-05	0.000109852
INPP5K	1.829253676	Up	2.47E-16	1.58E-15
INSIG1	5.15662803	Up	6.86E-06	1.44E-05
INSM1	2.642054857	Up	0.00026095	0.000386382
INTS12	1.144334754	Up	5.21E-05	9.11E-05
INTS4	2.660855484	Up	1.61E-12	7.85E-12
INTS6	8.226330197	Up	2.59E-34	3.50E-33
INTS9	2.2815863	Up	0.000123326	0.000197507
intu	1.592129632	Up	1.22E-06	2.93E-06
IP6K2	5.373858746	Up	1.13E-06	2.74E-06
IPO8	4.642054857	Up	0.000212366	0.000320567
IPPK	1.922162776	Up	2.74E-06	6.22E-06
IQCA1	1.28690401	Up	0.000569892	0.00077168
IQCF3	5.004624937	Up	2.12E-05	4.03E-05
IQCH	4.553883376	Up	0.00034572	0.000495888
IQCK	5.41424216	Up	7.87E-07	1.96E-06
IQGAP2	1.617967176	Up	8.77E-32	1.10E-30
IRF9	1.950177153	Up	0.000722703	0.000953274
ISCU	4.484315394	Up	0.000498617	0.000688336
ISG20	4.789826716	Up	8.82E-05	0.000146179
ISPD	4.834699935	Up	6.65E-05	0.000113504
ISY1	4.741590531	Up	0.000118566	0.000190485
ISY1-RAB43	4.613959656	Up	0.000248757	0.000370114
ITCH	2.880104133	Up	2.26E-42	3.82E-41
ITGAV	4.741590531	Up	0.000118566	0.000190485
ITGAX	5.33804867	Up	1.55E-06	3.66E-06
ITGB1BP1	3.027117297	Up	2.78E-07	7.46E-07
ITSN1	5.140305725	Up	2.56E-10	1.01E-09
JADE1	6.785168349	Up	4.21E-15	2.48E-14
JAK2	1.007461589	Up	4.78E-11	2.04E-10
JARID2	1.320920251	Up	1.68E-07	4.67E-07
JKAMP	4.419662436	Up	0.000691126	0.00091553
JMJD4	3.535139653	Up	7.29E-16	4.51E-15
jmjd7-pla	5.512239251	Up	3.63E-16	2.29E-15

2g4b				
josd2	1.986933032	Up	2.10E-08	6.62E-08
JRKL	1.871123343	Up	0.000236823	0.00035387
JUP	5.160949636	Up	6.63E-06	1.40E-05
KANK1	7.0268941	Up	2.33E-17	1.58E-16
KANSL1	1.133479166	Up	6.97E-12	3.22E-11
L				
KANSL2	1.748970061	Up	1.63E-05	3.19E-05
KARS	3.337200276	Up	1.97E-07	5.41E-07
KAT2A	2.675015056	Up	0.000399768	0.000564974
KAT6B	4.642054857	Up	0.000212366	0.000320567
KATNAL	8.396789685	Up	9.56E-38	1.43E-36
1				
KAZN	5.522320567	Up	2.86E-07	7.68E-07
KCNA10	2.69268093	Up	8.77E-10	3.28E-09
KCNAB2	5.234554609	Up	3.69E-06	8.19E-06
KCNC1	1.608431323	Up	3.31E-06	7.39E-06
KCNC4	4.944896113	Up	3.21E-05	5.89E-05
KCND3	6.357320696	Up	8.60E-12	3.94E-11
KCNH5	1.615827715	Up	5.65E-07	1.44E-06
KCNH6	2.297395044	Up	4.25E-05	7.56E-05
kcnip2	3.082627449	Up	0.000341743	0.000490632
KCNIP4	1.093434204	Up	0.00041565	0.00058529
KCNJ6	2.571665529	Up	0.000446586	0.000623907
KCNMA1	2.036104573	Up	2.78E-05	5.17E-05
KCNMB2	3.864447279	Up	4.09E-11	1.76E-10
KCNMB3	4.642054857	Up	0.000212366	0.000320567
KCNQ3	1.315325776	Up	4.54E-08	1.37E-07
KCNS1	4.741590531	Up	0.000118566	0.000190485
KCNS2	1.180474772	Up	0.000341156	0.00048987
KCNT2	1.834699935	Up	0.000318327	0.000460085
KCNV1	4.419662436	Up	0.000691126	0.00091553
KCTD14	5.358261891	Up	1.30E-06	3.11E-06
KCTD17	4.545093127	Up	0.000362409	0.000517863
KCTD19	2.453609768	Up	3.85E-18	2.73E-17
KCTD3	2.15662803	Up	0.00010667	0.000173549
KDELR2	4.535139653	Up	0.000382164	0.000541831
KDM2B	1.35882955	Up	7.09E-22	6.08E-21
KDM3A	8.535694429	Up	8.78E-41	1.43E-39
KDM4C	1.672088978	Up	4.41E-12	2.07E-11
KDM7A	2.571665529	Up	0.000446586	0.000623907
KIAA023	2.225757312	Up	5.75E-16	3.58E-15
2				
KIAA031	3.637019267	Up	1.01E-05	2.05E-05

9				
KIAA0408	3.065234043	Up	2.33E-21	1.95E-20
KIAA0430	5.891283464	Up	5.33E-09	1.82E-08
KIAA0825	1.623007723	Up	2.72E-18	1.94E-17
KIAA0907	2.244646241	Up	2.94E-08	9.13E-08
KIAA1024	2.56262039	Up	1.17E-08	3.83E-08
KIAA1107	1.396578823	Up	5.90E-09	2.00E-08
KIAA1143	1.915451466	Up	8.63E-06	1.78E-05
KIAA1217	1.205609767	Up	4.27E-09	1.48E-08
KIAA1257	2.049712826	Up	3.35E-08	1.03E-07
KIAA1324	4.832895438	Up	6.73E-05	0.000114708
KIAA1324L	5.358261891	Up	1.30E-06	3.11E-06
KIAA1429	4.786101227	Up	9.03E-05	0.0001492
KIAA1958	6.56262039	Up	2.80E-13	1.44E-12
KIAA2012	1.963982952	Up	8.12E-05	0.000135608
KIF14	6.552787519	Up	3.32E-13	1.71E-12
KIF16B	4.905089263	Up	4.20E-05	7.49E-05
KIF17	8.189522314	Up	1.30E-33	1.73E-32
KIF18B	4.537357479	Up	0.000377682	0.000538023
KIF1B	1.35254824	Up	0.000542648	0.000739628
KIF21B	1.259199241	Up	7.37E-28	8.12E-27
KIF23	5.004624937	Up	2.12E-05	4.03E-05
KIF27	7.846150847	Up	1.06E-27	1.16E-26
KIF9	5.163823531	Up	6.48E-06	1.37E-05
KIFAP3	1.190280083	Up	5.24E-05	9.16E-05
KIN	2.148104557	Up	2.66E-05	4.96E-05
KIRREL2	4.922162776	Up	3.75E-05	6.77E-05
KIT	1.933321612	Up	1.60E-05	3.13E-05
KLF15	4.535139653	Up	0.000382164	0.000541831
KLF3	2.893593624	Up	2.95E-05	5.46E-05

KLHDC4	3.843928721	Up	1.58E-08	5.07E-08
KLHL12	4.816552588	Up	7.46E-05	0.000125676
KLHL2	3.921313883	Up	9.41E-05	0.000154664
KLHL20	1.709169053	Up	1.32E-08	4.30E-08
KLHL3	2.162767167	Up	2.57E-09	9.10E-09
KLHL41	1.755265468	Up	7.00E-06	1.47E-05
KLHL42	3.082627449	Up	0.000173799	0.000267992
KLHL5	5.15662803	Up	6.86E-06	1.44E-05
KLHL8	5.15662803	Up	6.86E-06	1.44E-05
KLK11	4.510515866	Up	0.000435181	0.000609805
KLK7	5.478556125	Up	4.35E-07	1.13E-06
KNTC1	1.639228813	Up	5.87E-21	4.81E-20
KRT18	5.749742727	Up	2.72E-08	8.47E-08
KRT38	5.082627449	Up	1.20E-05	2.40E-05
KRT6B	2.791221299	Up	4.86E-06	1.05E-05
KRT74	4.419662436	Up	0.000691126	0.00091553
KRT85	1.571836252	Up	1.84E-05	3.56E-05
KRTAP17-1	3.10771843	Up	3.23E-06	7.23E-06
Krtap5-5	4.419662436	Up	0.000691126	0.00091553
KRTDAP	1.773299391	Up	2.48E-09	8.80E-09
KTN1	5.174549938	Up	5.96E-06	1.27E-05
KXD1	5.507125277	Up	3.31E-07	8.78E-07
L1TD1	5.708513134	Up	4.27E-08	1.30E-07
L3HYPDH	1.922162776	Up	0.000128602	0.000205005
L3MBTL3	1.943821658	Up	4.30E-16	2.70E-15
L3MBTL4	2.384897018	Up	1.47E-06	3.49E-06
LACE1	1.986703029	Up	1.93E-09	6.95E-09
LAMA2	1.90564041	Up	2.38E-05	4.49E-05
LAMA4	4.834699935	Up	6.65E-05	0.000113504
LAMC1	2.492662296	Up	3.88E-18	2.75E-17
LANCL1	1.113207764	Up	5.38E-08	1.61E-07
LATS1	1.636581615	Up	0.000108505	0.00017624
LCMT1	5.044153301	Up	1.60E-05	3.12E-05
LCN2	4.642054857	Up	0.000212366	0.000320567
LCP2	3.922162776	Up	9.36E-05	0.000153908
LDAH	1.314692876	Up	0.0002803	0.000411061
LDB1	2.75413198	Up	2.66E-23	2.43E-22
LDB3	6.10416061	Up	3.12E-11	1.36E-10
LDLRAD4	9.955689425	Up	1.55E-88	5.62E-87

LEPROT L1	3.075014265	Up	3.35E-09	1.17E-08
LHFP	3.922162776	Up	9.36E-05	0.000153908
LHX6	1.49061019	Up	2.41E-08	7.57E-08
LIAS	2.505002105	Up	7.90E-07	1.97E-06
LIG4	5.478556125	Up	8.92E-13	4.43E-12
LILRA2	2.854471156	Up	3.37E-05	6.16E-05
LILRA4	3.057092357	Up	5.63E-06	1.21E-05
LIMCH1	5.004624937	Up	2.12E-05	4.03E-05
LIN52	2.128715853	Up	5.07E-18	3.57E-17
LINGO1	7.608036984	Up	3.35E-24	3.18E-23
LITAF	3.922162776	Up	9.36E-05	0.000153908
LMAN2L	1.114807854	Up	0.000303496	0.000442212
LMBR1L	1.355056824	Up	1.10E-06	2.66E-06
LMNTD1	1.914725421	Up	5.23E-06	1.13E-05
LMO1	2.261555366	Up	2.87E-08	8.91E-08
LMO7	4.789826716	Up	8.82E-05	0.000146179
LMX1A	4.834699935	Up	6.65E-05	0.000113504
LNPK	6.879094055	Up	6.08E-16	3.78E-15
LOC1009 96634	1.449409779	Up	0.000168454	0.000260719
LOC2862 38	1.094295257	Up	1.51E-05	2.97E-05
LOC4009 27-CSNK 1E	4.428083341	Up	6.89E-14	3.73E-13
LOC4411 55	1.054979765	Up	6.55E-07	1.65E-06
LOC7301 59	2.097974854	Up	4.78E-118	2.35E-116
LOC8169 1	4.674659523	Up	0.000176144	0.000271236
LOXL3	1.922162776	Up	2.74E-06	6.22E-06
LPAR1	6.358261891	Up	8.48E-12	3.89E-11
LPAR3	3.082627449	Up	0.000173799	0.000267992
LPAR5	5.69268093	Up	5.06E-08	1.52E-07
lpar6	6.637151676	Up	7.27E-14	3.93E-13
LPCAT3	5.082627449	Up	1.20E-05	2.40E-05
LPIN1	1.418325402	Up	9.68E-27	1.02E-25
LPXN	5.760982327	Up	2.25E-31	2.79E-30
LRMP	5.69268093	Up	5.06E-08	1.52E-07
LRP12	5.008626867	Up	2.06E-05	3.94E-05
LRP2BP	1.788896246	Up	0.000497148	0.000686416
lrp6	2.551229708	Up	1.31E-06	3.14E-06

LRP8	8.116398185	Up	2.92E-32	3.72E-31
LRRC27	1.815642627	Up	5.71E-21	4.68E-20
LRRC37A	1.853097403	Up	3.32E-225	3.14E-223
LRRC37A2	1.052505914	Up	4.39E-223	4.12E-221
LRRC43	3.036856417	Up	6.99E-06	1.47E-05
LRRC49	2.575873952	Up	1.96E-32	2.50E-31
LRRC52	1.659128371	Up	4.51E-08	1.37E-07
LRRC61	5.082627449	Up	1.20E-05	2.40E-05
LRRC70	1.898830273	Up	9.98E-06	2.04E-05
LRRC8A	5.691660677	Up	1.32E-58	3.12E-57
LRRC8C	1.973704442	Up	5.43E-58	1.26E-56
LRRCC1	2.057092357	Up	0.000280305	0.000411061
LRRFIP2	3.078533035	Up	1.94E-05	3.73E-05
LRRIQ4	1.01594025	Up	5.51E-12	2.57E-11
LRRK1	2.031097148	Up	4.42E-07	1.15E-06
LRTM2	6.507125277	Up	7.35E-13	3.67E-12
LRTOMT	5.170268941	Up	6.16E-06	1.31E-05
LSAMP	4.419662436	Up	0.000691126	0.00091553
LSM4	3.052007067	Up	0.000195313	0.000298324
LSP1	2.250736185	Up	3.34E-08	1.03E-07
LTA4H	5.358261891	Up	1.30E-06	3.11E-06
LTBP3	5.700123913	Up	2.32E-05	4.38E-05
LTN1	1.294555938	Up	7.34E-47	1.37E-45
LUC7L2	7.449409779	Up	4.12E-22	3.57E-21
LUZP6	3.69268093	Up	0.000408265	0.000575695
Ly6k	1.520849735	Up	0.0002313	0.000346537
LYPD4	3.847144702	Up	7.62E-08	2.23E-07
LYRM1	1.520158817	Up	4.53E-05	8.02E-05
LZTFL1	4.922162776	Up	3.75E-05	6.77E-05
LZTS2	3.672120247	Up	1.80E-71	5.21E-70
m1ap	4.535139653	Up	0.000382164	0.000541831
MACF1	1.094187455	Up	8.66E-07	2.14E-06
MAD2L2	1.026231393	Up	3.36E-15	1.99E-14
MADD	3.296942569	Up	8.48E-77	2.64E-75
MAEA	4.419662436	Up	0.000691126	0.00091553
MAFF	5.082627449	Up	1.20E-05	2.40E-05
Maged4	4.496905435	Up	0.000467185	0.000649645
MAGED4B	4.496905435	Up	0.000467185	0.000649645
MAGI1	6.421164461	Up	3.09E-12	1.47E-11
MAGI3	2.282807979	Up	6.23E-08	1.84E-07
MAGOHB	5.227017358	Up	3.92E-06	8.66E-06

MAK	1.279570117	Up	7.04E-06	1.48E-05
MALL	4.454112946	Up	0.000581716	0.000786624
MALRD1	1.461331615	Up	1.25E-19	9.60E-19
MAN2C1	7.171518878	Up	7.33E-19	5.40E-18
MANEAL	4.521760624	Up	0.000410215	0.000578277
MAOA	6.113056463	Up	3.10E-10	1.22E-09
MAP1S	1.404808361	Up	1.79E-20	1.44E-19
MAP2K2	1.138938796	Up	1.30E-31	1.62E-30
MAP2K4	3.834699935	Up	0.000168506	0.000260719
map2k5	3.081395604	Up	0.00030786	0.000446589
MAP3K19	5.949483382	Up	2.62E-09	9.25E-09
MAP3K3	7.294131554	Up	3.11E-20	2.47E-19
MAP3K7CL	4.157349197	Up	1.61E-05	3.15E-05
MAP3K9	1.878446487	Up	2.56E-06	5.85E-06
MAP4K1	4.535139653	Up	0.000382164	0.000541831
map4k4	1.647089276	Up	1.23E-24	1.18E-23
MAP6	2.270131915	Up	2.25E-28	2.52E-27
MAP7	1.308257672	Up	0.000636165	0.000853127
MAPK10	8.079396753	Up	1.35E-31	1.68E-30
mapk13	1.048147812	Up	2.15E-05	4.09E-05
MAPK3	2.354781728	Up	1.06E-48	2.06E-47
MAPKAP1	4.546194842	Up	0.000360279	0.000515121
MAPKAPK2	1.068096507	Up	4.70E-10	1.80E-09
MAPKAPK3	4.827468366	Up	6.96E-05	0.000118372
MAPRE3	2.517281494	Up	8.97E-11	3.71E-10
MARK3	6.912156588	Up	3.00E-16	1.90E-15
MARS	1.939036595	Up	1.48E-05	2.92E-05
MAST4	1.786291411	Up	1.36E-09	4.98E-09
MASTL	1.14452129	Up	1.98E-14	1.11E-13
MAT2B	4.741590531	Up	0.000118566	0.000190485
MAVS	1.790812291	Up	4.78E-14	2.61E-13
MAX	4.730002557	Up	0.000127125	0.000202954
MAZ	6.639218205	Up	7.00E-14	3.79E-13
MB	4.082627449	Up	2.90E-05	5.37E-05
MBD1	5.443509178	Up	6.03E-07	1.53E-06
MBD3	1.737523665	Up	0.000221406	0.000332964
MCC	1.244983904	Up	0.000676511	0.000902325
MCCC1	4.741590531	Up	0.000118566	0.000190485
MCM2	1.752237775	Up	0.000324483	0.000468119

MCM9	2.294131554	Up	0.000567443	0.000768838
MCRIP2	3.758582245	Up	3.34E-11	1.45E-10
MCRS1	5.922162776	Up	3.66E-09	1.28E-08
ME2	2.632461726	Up	3.62E-05	6.58E-05
MEAF6	3.55525637	Up	0.00019139	0.000292873
MECOM	4.354807837	Up	3.80E-11	1.64E-10
MECR	3.937865735	Up	7.93E-06	1.65E-05
MED24	4.535139653	Up	0.000382164	0.000541831
MED9	1.682696842	Up	1.07E-05	2.16E-05
MEDAG	1.449409779	Up	0.000168454	0.000260719
MEF2B	1.958721418	Up	0.000108885	0.000176794
MEIOB	3.811979859	Up	9.32E-14	5.00E-13
MEIOC	1.560271028	Up	8.97E-37	1.30E-35
MEMO1	5.505993308	Up	3.35E-07	8.87E-07
METAP2	6.358261891	Up	8.48E-12	3.89E-11
METRNL	1.834699935	Up	0.000318327	0.000460085
METTL1	1.549907128	Up	4.50E-11	1.93E-10
METTL10	2.640504406	Up	2.59E-05	4.84E-05
METTL13	3.443292431	Up	1.09E-20	8.87E-20
METTL21B	1.021182774	Up	2.61E-19	1.97E-18
METTL23	5.975069606	Up	1.90E-09	6.84E-09
METTL26	4.432826895	Up	0.000647359	0.000866895
mettl2a	2.720695306	Up	0.000118378	0.000190485
METTL6	2.590391269	Up	3.05E-08	9.44E-08
MEX3D	1.224491483	Up	0.000332522	0.000478647
MFAP3L	5.543990571	Up	2.32E-07	6.31E-07
MFF	4.990934733	Up	2.33E-05	4.41E-05
MFN2	1.629880143	Up	6.82E-06	1.44E-05
MFSD11	2.419662436	Up	1.45E-10	5.87E-10
mgat2	3.082627449	Up	0.000173799	0.000267992
MGAT3	7.257941931	Up	8.08E-20	6.27E-19
MGAT5	1.62106135	Up	2.23E-47	4.20E-46
MGLL	4.535139653	Up	0.000382164	0.000541831
mia2	6.19225194	Up	1.02E-10	4.22E-10
MIA3	1.285361344	Up	0.000343601	0.000493123
MIB1	1.050428626	Up	5.48E-07	1.40E-06
MICAL2	1.042580961	Up	0.000126297	0.000201807
MICU1	5.504860449	Up	3.39E-07	8.97E-07
MIEF2	5.589587437	Up	1.47E-07	4.13E-07
MIER1	5.43699052	Up	6.40E-07	1.62E-06
MIER3	1.487597822	Up	1.84E-08	5.85E-08
MIF4GD	1.372156035	Up	1.62E-09	5.90E-09
MIGA1	3.000688522	Up	1.06E-06	2.57E-06

MINK1	8.90696655	Up	4.37E-50	8.70E-49
MINOS1	4.51277184	Up	0.000430068	0.000603327
MIR1-1HG	4.535139653	Up	0.000382164	0.000541831
MITD1	1.560524972	Up	0.000197557	0.000301187
MKI67	2.014748328	Up	5.82E-14	3.17E-13
MKKS	5.660485563	Up	7.12E-08	2.09E-07
MKL1	6.195766424	Up	9.74E-11	4.02E-10
MKLN1	1.178654336	Up	0.000198282	0.000302197
MKNK1	5.996990549	Up	1.44E-09	5.25E-09
MKS1	1.145040056	Up	6.23E-07	1.58E-06
MLKL	4.15662803	Up	1.62E-05	3.16E-05
MLLT10	5.535139653	Up	2.53E-07	6.84E-07
MLLT11	1.444753417	Up	7.71E-09	2.59E-08
MLST8	1.263116464	Up	3.02E-12	1.44E-11
MMS19	6.513898506	Up	6.54E-13	3.28E-12
MOB2	5.227017358	Up	3.92E-06	8.66E-06
MOCS3	4.66251896	Up	0.00018893	0.0002893
MON1A	2.452707998	Up	3.01E-08	9.31E-08
MON1B	5.260190971	Up	8.34E-35	1.14E-33
MORC1	1.027345013	Up	5.93E-05	0.000102405
MORC2	4.053772586	Up	3.92E-43	6.75E-42
MORF4L1	4.193862043	Up	4.66E-19	3.47E-18
MORN1	4.834699935	Up	6.65E-05	0.000113504
MORN5	4.535139653	Up	0.000382164	0.000541831
MOV10	1.166543499	Up	0.000103821	0.000169245
MOV10L1	2.058727139	Up	0.000407244	0.000574467
MPDZ	7.274156854	Up	5.28E-20	4.13E-19
MPHOSPH9	2.207743346	Up	2.72E-37	4.01E-36
MPI	1.3091859	Up	2.36E-05	4.45E-05
MPP2	6.574008006	Up	2.28E-13	1.19E-12
MPP7	5.294131554	Up	2.25E-06	5.18E-06
MPZL2	1.304571563	Up	0.00011675	0.000188335
MPZL3	1.796870035	Up	2.33E-12	1.12E-11
MRAS	7.680189986	Up	3.25E-25	3.22E-24
MRC1	3.249737434	Up	6.04E-07	1.54E-06
MRGBP	2.338590825	Up	1.76E-43	3.04E-42
MRGPRE	2.384897018	Up	1.47E-06	3.49E-06
MRGPRF	3.497664948	Up	2.07E-08	6.54E-08
MROH1	4.673651702	Up	0.000177175	0.000272766
MROH8	2.127227114	Up	4.32E-05	7.69E-05

MRPL24	5.478556125	Up	4.35E-07	1.13E-06
MRPL30	4.845479774	Up	6.21E-05	0.000106814
MRPL34	1.487481881	Up	1.78E-06	4.17E-06
MRPL43	1.404009172	Up	0.000751334	0.000986593
MRPS12	8.051251805	Up	4.23E-31	5.20E-30
MRPS18A	3.834699935	Up	0.000168506	0.000260719
MRPS31	2.312747232	Up	4.00E-06	8.81E-06
MS4A6E	3.974251288	Up	1.34E-29	1.57E-28
MSANTD1	1.071739132	Up	2.43E-05	4.57E-05
MSL2	5.220821092	Up	4.12E-06	9.06E-06
mst1l	1.050422901	Up	0.000167753	0.000260003
MSTO1	1.108811048	Up	3.38E-13	1.73E-12
MT1E	1.660670535	Up	0.000209433	0.000317582
MTCH2	4.963982952	Up	2.82E-05	5.23E-05
MTERF2	2.097734341	Up	0.000659832	0.000881714
MTFR2	3.272105247	Up	9.83E-13	4.87E-12
MTG1	4.820200365	Up	2.03E-08	6.42E-08
mtg2	1.756048583	Up	0.000519644	0.000711544
MTMR2	1.025841423	Up	1.66E-06	3.90E-06
mto1	5.806013559	Up	1.45E-08	4.68E-08
MTPAP	1.76486031	Up	3.37E-06	7.51E-06
MTPN	3.69268093	Up	0.000408265	0.000575695
MTR	8.294787175	Up	1.18E-35	1.66E-34
MTRF1L	5.15662803	Up	6.86E-06	1.44E-05
MTRNR2L2	1.322003383	Up	7.56E-21	6.16E-20
MTRNR2L3	2.056405195	Up	8.70E-06	1.80E-05
MTTP	1.618971244	Up	4.43E-06	9.68E-06
MTX3	4.568054276	Up	0.000320244	0.000462688
MUC1	1.243477001	Up	3.17E-18	2.26E-17
MUC17	5.358261891	Up	1.30E-06	3.11E-06
MUSK	5.004624937	Up	2.12E-05	4.03E-05
MYBL1	2.760293969	Up	0.000164349	0.000255068
MYEF2	1.421005477	Up	4.03E-08	1.23E-07
MYH11	2.871791959	Up	6.54E-69	1.83E-67
MYH14	8.542473179	Up	6.16E-41	1.00E-39
MYH15	1.443509178	Up	2.85E-05	5.30E-05
MYH7	2.709169053	Up	6.59E-05	0.000112873
MYL6B	2.191661025	Up	4.34E-10	1.68E-09
MYLK	6.047658298	Up	7.45E-10	2.81E-09
MYLK3	2.607993021	Up	0.000339692	0.000488216

MYO1B	4.209739367	Up	1.20E-18	8.73E-18
MYO1C	6.56262039	Up	2.80E-13	1.44E-12
MYO1D	3.555970439	Up	3.29E-12	1.56E-11
MYO1E	1.15662803	Up	3.27E-08	1.01E-07
MYO3A	2.482028317	Up	2.87E-08	8.89E-08
MYO7A	1.097734341	Up	0.000241659	0.000360629
MYO9A	1.151241484	Up	8.31E-55	1.82E-53
MYOC	1.419662436	Up	0.000246377	0.000367036
MYOM1	7.004624937	Up	3.88E-17	2.60E-16
MYRF	5.963982952	Up	2.18E-09	7.80E-09
MYRIP	4.535139653	Up	0.000382164	0.000541831
MZF1	3.012627959	Up	8.43E-13	4.19E-12
MZT2B	1.620155376	Up	0.000328647	0.000473723
N4BP2	2.420864181	Up	0.000200902	0.000305999
N4BP2L2	4.582087335	Up	0.00029667	0.000433264
NAA60	6.568325434	Up	2.53E-13	1.31E-12
NAALAD 2	1.883026049	Up	8.26E-54	1.78E-52
NADK	4.082627449	Up	3.36E-09	1.17E-08
NAIP	1.406994435	Up	2.84E-06	6.44E-06
NANOG	4.913651277	Up	3.97E-05	7.11E-05
NAP1L1	4.475089495	Up	0.000522817	0.000715515
NAPA	2.381267057	Up	5.34E-05	9.32E-05
NARF	6.622602495	Up	9.50E-14	5.09E-13
NARS	2.097734341	Up	0.000659832	0.000881714
NAT10	5.050378532	Up	1.52E-05	2.99E-05
NAT6	1.968857501	Up	0.000165422	0.000256644
NAT9	5.175974119	Up	5.89E-06	1.26E-05
NAV2	2.160865023	Up	5.87E-06	1.25E-05
NAXD	2.799120399	Up	2.31E-07	6.29E-07
NBPF12	1.515371809	Up	2.78E-07	7.47E-07
NCAPG	3.439561993	Up	1.18E-14	6.71E-14
NCAPH	4.419662436	Up	0.000691126	0.00091553
NCDN	6.579130165	Up	2.08E-13	1.09E-12
NCEH1	7.105415743	Up	3.69E-18	2.62E-17
NCKAP5	2.228181291	Up	5.83E-05	0.000100936
NCL	1.5347556	Up	3.56E-66	9.54E-65
NCOA1	3.17488344	Up	5.63E-06	1.21E-05
NCOA3	2.834699935	Up	1.34E-19	1.03E-18
NCOR1	4.573581762	Up	5.38E-69	1.50E-67
NDE1	2.211790884	Up	0.000222685	0.000334499
NDFIP2	2.201022149	Up	3.47E-20	2.74E-19
NDRG1	3.453380341	Up	1.71E-14	9.68E-14
NDRG3	3.116370428	Up	2.29E-29	2.66E-28

NDST1	5.788896246	Up	1.76E-08	5.61E-08
NDST3	2.989315546	Up	0.000340583	0.00048916
NDUFA13	1.074706474	Up	2.01E-12	9.72E-12
NDUFS1	1.592129632	Up	1.22E-06	2.93E-06
NDUFS8	1.35254824	Up	1.03E-08	3.41E-08
NDUFV3	4.629635598	Up	0.000227822	0.000341747
NECAB3	2.963241016	Up	8.22E-07	2.04E-06
NECAP2	3.179731272	Up	2.01E-25	2.01E-24
NECTIN2	1.08995699	Up	0.000215435	0.000324742
NEDD1	2.070561062	Up	0.000265515	0.000391137
NEK1	5.997796074	Up	1.42E-09	5.20E-09
NEK5	1.277959897	Up	2.16E-11	9.55E-11
NELFCD	1.440420996	Up	7.62E-06	1.59E-05
neurl4	1.251916733	Up	7.58E-07	1.89E-06
NF1	1.190527906	Up	2.25E-19	1.70E-18
NF2	6.028865804	Up	9.53E-10	3.55E-09
NFASC	2.18440471	Up	7.29E-05	0.000123065
NFAT5	1.864835007	Up	2.33E-28	2.61E-27
NFATC1	4.429248485	Up	0.000659009	0.000881184
NFATC2	6.15662803	Up	1.70E-10	6.83E-10
NFATC3	2.192086968	Up	1.60E-17	1.10E-16
NFE2L2	5.788896246	Up	1.76E-08	5.61E-08
NFIB	1.079812433	Up	0.000637631	0.000854884
nfic	2.157842704	Up	2.51E-13	1.30E-12
NFIL3	1.972673469	Up	3.55E-11	1.53E-10
NFIX	2.516431885	Up	5.31E-24	5.00E-23
NFKBIB	5.600234682	Up	1.32E-07	3.74E-07
NFRKB	1.525824243	Up	3.49E-09	1.22E-08
NFX1	1.727560971	Up	4.67E-09	1.61E-08
NFYA	6.485464494	Up	1.06E-12	5.25E-12
NFYC	4.920464489	Up	3.79E-05	6.84E-05
NGLY1	1.713366598	Up	6.94E-10	2.63E-09
NIN	4.578861031	Up	0.000301949	0.000440431
NIPA2	1.821875895	Up	5.78E-05	0.000100165
NIPBL	1.22043919	Up	3.23E-13	1.66E-12
NIPSNAP1	1.834699935	Up	0.000318327	0.000460085
NLGN3	5.589587437	Up	1.47E-07	4.13E-07
NLRP11	4.879094055	Up	9.67E-09	3.20E-08
NLRP2	2.069691406	Up	2.01E-19	1.53E-18
NMD3	4.15662803	Up	1.62E-05	3.16E-05
NME4	5.613434179	Up	1.16E-07	3.29E-07
NOC2L	1.876955144	Up	8.32E-05	0.000138731
NOD1	1.676002189	Up	8.92E-05	0.000147481

NOL3	3.011692655	Up	0.000567385	0.000768838
NOL4	1.573932555	Up	2.80E-09	9.86E-09
NOL4L	2.027975424	Up	1.58E-19	1.20E-18
NOL7	1.722225206	Up	1.61E-07	4.47E-07
NOL8	5.589587437	Up	1.47E-07	4.13E-07
nomo3	4.831088682	Up	6.80E-05	0.000115911
NOP2	1.888554837	Up	1.10E-13	5.89E-13
NOS3	5.571845855	Up	1.76E-07	4.88E-07
NOVA1	1.642054857	Up	3.75E-05	6.78E-05
NOXRED 1	3.294131554	Up	3.16E-05	5.81E-05
NPAS3	1.31767902	Up	3.09E-05	5.68E-05
NPC1L1	1.219702133	Up	1.50E-13	7.90E-13
NPEPL1	1.741590531	Up	3.82E-05	6.89E-05
NPFFR2	3.082247742	Up	1.11E-07	3.15E-07
NPRL3	5.401515089	Up	8.83E-07	2.18E-06
NPSR1	5.004624937	Up	2.12E-05	4.03E-05
NQO1	3.505615787	Up	1.84E-08	5.86E-08
NR1D2	3.180474772	Up	8.86E-12	4.06E-11
NR1H3	3.203933745	Up	1.06E-06	2.57E-06
NR1I3	4.642054857	Up	0.000212366	0.000320567
NR2C2	1.147272119	Up	8.53E-12	3.91E-11
NR3C2	1.370752835	Up	1.54E-06	3.65E-06
NR4A1	6.841446768	Up	1.34E-15	8.13E-15
NR6A1	3.807949982	Up	3.06E-88	1.10E-86
NRG2	5.589587437	Up	1.47E-07	4.13E-07
NRM	1.953001972	Up	8.92E-39	1.37E-37
NRXN1	1.834699935	Up	3.56E-07	9.40E-07
NRXN2	1.203327564	Up	5.30E-05	9.26E-05
NSD1	1.130603156	Up	4.73E-21	3.90E-20
NSF	1.741590531	Up	3.16E-10	1.24E-09
NSUN2	3.15662803	Up	9.86E-05	0.000161311
NT5C1B	5.937358067	Up	3.04E-09	1.07E-08
NT5C2	4.971383513	Up	2.67E-05	4.99E-05
NTAN1	3.203933745	Up	1.06E-06	2.57E-06
NTM	3.642054857	Up	0.000548719	0.000746049
NTMT1	3.364330565	Up	7.46E-09	2.50E-08
NTNG1	4.419662436	Up	0.000691126	0.00091553
NUB1	4.741590531	Up	0.000118566	0.000190485
NUCB2	1.183605096	Up	3.34E-05	6.10E-05
NUDT4	2.509004594	Up	1.89E-13	9.91E-13
NUDT6	2.709169053	Up	0.000151978	0.00023744
NUFIP2	1.794965112	Up	1.33E-58	3.14E-57
NUP153	9.338088427	Up	2.00E-63	5.16E-62

NUP155	2.254025529	Up	1.21E-37	1.80E-36
NUP160	1.084678188	Up	4.60E-44	8.08E-43
NUP210L	1.834699935	Up	1.22E-23	1.13E-22
NUP214	1.251129431	Up	2.41E-28	2.70E-27
NUP35	2.13804233	Up	0.00047314	0.000657092
NUP43	1.641668128	Up	1.06E-05	2.16E-05
NUP98	1.41340561	Up	8.53E-44	1.49E-42
NUPR2	2.124206552	Up	2.47E-58	5.79E-57
NUSAP1	4.015031752	Up	2.25E-14	1.26E-13
NUTM1	4.834699935	Up	6.65E-05	0.000113504
NUTM2A	1.196991905	Up	0.000152636	0.000238374
NWD1	6.312700991	Up	1.72E-11	7.66E-11
NWD2	5.004624937	Up	2.12E-05	4.03E-05
NXPE1	2.709169053	Up	0.000151978	0.00023744
OAZ3	1.333082617	Up	6.14E-140	3.58E-138
OBSCN	4.717342985	Up	0.000137106	0.000217385
ODC1	1.544901986	Up	7.71E-06	1.61E-05
ODF1	1.369036363	Up	6.83E-65	1.79E-63
ODF2	3.286175952	Up	1.55E-11	6.95E-11
ODF2L	1.152211291	Up	1.72E-05	3.34E-05
OGFOD2	6.346610684	Up	1.02E-11	4.64E-11
OGFOD3	6.397247659	Up	4.56E-12	2.14E-11
OLFM1	2.079992759	Up	7.06E-11	2.96E-10
OLFM2	2.525787376	Up	1.00E-05	2.05E-05
olfml2a	2.899329994	Up	5.98E-08	1.77E-07
OPN4	1.497664948	Up	3.21E-05	5.90E-05
OPRL1	2.360848258	Up	6.70E-06	1.41E-05
OPRM1	2.343829212	Up	2.52E-07	6.83E-07
OPTN	6.004624937	Up	1.30E-09	4.78E-09
OR10J3	1.816552588	Up	1.94E-15	1.17E-14
OR10S1	4.419662436	Up	0.000691126	0.00091553
OR11G2	1.394127344	Up	9.39E-05	0.000154366
OR14J1	1.08995699	Up	0.000215435	0.000324742
OR1L1	2.571665529	Up	0.000446586	0.000623907
OR1N2	2.10771843	Up	0.000173302	0.00026746
OR2A4	1.067090065	Up	1.34E-13	7.12E-13
OR2H2	1.397109741	Up	3.69E-51	7.51E-50
OR2J3	5.004624937	Up	2.12E-05	4.03E-05
OR2V2	4.741590531	Up	0.000118566	0.000190485
OR4C15	3.741590531	Up	0.000303865	0.000442212
OR4C16	4.419662436	Up	0.000691126	0.00091553
OR4K14	3.834699935	Up	0.000168506	0.000260719
OR4K15	3.639992391	Up	0.000555251	0.000754205
OR4N4	7.699752675	Up	1.70E-25	1.70E-24

OR51T1	3.741590531	Up	0.000303865	0.000442212
OR56A4	2.253320402	Up	1.23E-09	4.52E-09
OR6M1	4.834699935	Up	6.65E-05	0.000113504
OR6P1	3.642054857	Up	0.000548719	0.000746049
OR7E24	2.01230696	Up	1.74E-06	4.09E-06
ORAI2	1.843671067	Up	5.76E-07	1.47E-06
ORC1	1.721106509	Up	4.27E-11	1.83E-10
ORM2	5.642054857	Up	8.63E-08	2.50E-07
OS9	4.411222073	Up	0.000720529	0.000951236
OSBP2	1.578116726	Up	6.59E-06	1.39E-05
OSBPL10	7.401819423	Up	1.61E-21	1.36E-20
OSBPL11	1.529845354	Up	3.97E-06	8.74E-06
OSBPL2	5.574008006	Up	1.72E-07	4.78E-07
OSBPL9	8.239756281	Up	1.42E-34	1.94E-33
OSCP1	5.478556125	Up	4.35E-07	1.13E-06
OTOA	4.534029461	Up	0.000384426	0.0005448
OTUD4	4.811063571	Up	7.73E-05	0.00012975
OTUD6B	4.741590531	Up	0.000118566	0.000190485
OVOL2	4.723199671	Up	0.000132404	0.000210532
OXGR1	2.249737434	Up	3.99E-05	7.15E-05
P2RX5	5.535139653	Up	2.53E-07	6.84E-07
P2RY2	4.543990571	Up	0.000364552	0.000520756
P2RY6	5.082627449	Up	1.20E-05	2.40E-05
P3H1	1.497664948	Up	2.66E-19	2.01E-18
P3H2	4.419662436	Up	0.000691126	0.00091553
P4HA3	1.642054857	Up	6.68E-15	3.88E-14
P4HTM	7.548945453	Up	2.11E-23	1.93E-22
PABPC1	1.294131554	Up	2.34E-07	6.37E-07
PACRG	3.458215677	Up	6.96E-06	1.46E-05
PAFAH1B2	4.419662436	Up	0.000691126	0.00091553
PAFAH1B3	4.227017358	Up	9.05E-06	1.86E-05
PAH	1.197270015	Up	0.000654174	0.000875299
PAIP1	4.828374297	Up	6.92E-05	0.000117773
PAIP2	3.967195107	Up	2.26E-06	5.19E-06
PAK4	4.535139653	Up	0.000382164	0.000541831
PAK5	1.535139653	Up	0.000460943	0.000641962
PALM2	4.419662436	Up	0.000691126	0.00091553
PANX3	2.642054857	Up	0.00026095	0.000386382
PAOX	4.727090961	Up	0.000129361	0.000206103
PAPD5	6.416954844	Up	3.31E-12	1.57E-11
PAPOLG	4.7055578	Up	0.000147023	0.000230486
PAPPA2	1.792910822	Up	2.28E-08	7.17E-08

PAQR5	5.004624937	Up	1.82E-09	6.56E-09
PARD3	1.159960411	Up	1.02E-08	3.37E-08
PARD3B	6.055416827	Up	6.73E-10	2.55E-09
PARK2	4.839201327	Up	6.46E-05	0.000110902
PARN	2.485464494	Up	7.03E-09	2.37E-08
parp2	2.922162776	Up	0.000537549	0.000733476
PARP4	2.162746491	Up	7.25E-15	4.19E-14
PARP6	1.465543025	Up	2.91E-43	5.03E-42
PASK	2.314907873	Up	1.13E-05	2.27E-05
PATZ1	5.81381069	Up	1.32E-08	4.31E-08
PAX2	4.690689632	Up	0.000160449	0.000249599
PAX3	1.025780551	Up	0.000274457	0.000403307
PAX5	5.227017358	Up	3.92E-06	8.66E-06
PBXIP1	2.846632799	Up	3.13E-45	5.67E-44
PC	1.50427986	Up	2.79E-10	1.10E-09
PCBP2	1.571829277	Up	6.22E-10	2.36E-09
PCBP4	2.289062241	Up	3.99E-30	4.76E-29
PCDH1	7.309946982	Up	2.03E-20	1.63E-19
PCDH10	1.377990706	Up	3.44E-06	7.67E-06
PCDH11Y	2.315604621	Up	2.57E-09	9.11E-09
PCDH15	7.821907091	Up	2.53E-27	2.72E-26
PCDH19	4.625624513	Up	2.44E-06	5.59E-06
PCDH7	4.689692952	Up	0.000161387	0.000251005
PCDHA12	5.248158623	Up	3.30E-06	7.38E-06
PCDHA9	1.312992317	Up	1.86E-07	5.14E-07
PCDHAC1	1.210283342	Up	1.52E-07	4.24E-07
PCDHB11	1.925608431	Up	1.74E-09	6.31E-09
PCDHB12	1.03675824	Up	2.68E-20	2.13E-19
PCDHB13	1.266704285	Up	9.69E-11	4.00E-10
PCDHB6	1.843281664	Up	3.92E-06	8.66E-06
PCDHB8	1.332964175	Up	1.15E-52	2.44E-51
PCDHGA2	1.440084728	Up	8.86E-06	1.83E-05
PCDHGA4	1.891283464	Up	0.000178606	0.000274719
PCDHGA9	4.419662436	Up	0.000691126	0.00091553
PCDHGB1	5.814657151	Up	6.46E-15	3.75E-14
PCDHGB2	2.745548544	Up	1.77E-09	6.40E-09
PCF11	1.116626855	Up	7.67E-40	1.22E-38

PCGF3	4.741590531	Up	0.000118566	0.000190485
PCK2	4.814725235	Up	7.55E-05	0.000126989
PCLO	6.307187707	Up	1.87E-11	8.32E-11
PCM1	7.796554743	Up	6.19E-27	6.59E-26
PCNX1	1.042000597	Up	1.52E-34	2.07E-33
PCYOX1L	1.232035433	Up	8.23E-07	2.04E-06
PCYT2	8.274987756	Up	2.92E-35	4.05E-34
PDCD6IP	7.752847609	Up	2.82E-26	2.92E-25
PDE10A	1.709169053	Up	1.06E-07	3.02E-07
PDE1A	2.097232621	Up	4.45E-05	7.89E-05
PDE3A	2.453609768	Up	5.35E-07	1.37E-06
PDE4C	1.114278389	Up	1.03E-06	2.50E-06
PDE4D	5.215290869	Up	4.31E-06	9.44E-06
PDE7A	1.753815606	Up	5.27E-12	2.46E-11
PDE8A	5.589587437	Up	1.47E-07	4.13E-07
PDE8B	5.419662436	Up	7.50E-07	1.87E-06
PDK1	7.358261891	Up	5.45E-21	4.48E-20
PDLIM5	2.195050671	Up	9.63E-18	6.69E-17
PDP1	2.041551506	Up	1.06E-05	2.16E-05
PDPK1	6.943224392	Up	1.53E-16	9.89E-16
PDS5B	1.483792773	Up	5.54E-06	1.19E-05
PDXK	1.238243309	Up	3.46E-24	3.28E-23
PDZD9	3.036333796	Up	6.62E-09	2.24E-08
PDZK1	1.077248513	Up	1.07E-37	1.60E-36
PEG3	1.771789291	Up	2.17E-33	2.86E-32
pelp1	1.675103154	Up	1.84E-139	1.07E-137
PEPD	4.967276778	Up	2.75E-05	5.12E-05
PER3	3.893593624	Up	2.32E-11	1.02E-10
PEX1	1.095824751	Up	1.10E-07	3.15E-07
PEX11B	1.017544408	Up	7.37E-14	3.98E-13
PEX26	1.258340293	Up	7.62E-05	0.000128079
PFKM	2.439561993	Up	7.50E-11	3.13E-10
PFN2	2.515259998	Up	2.30E-13	1.20E-12
PGA3	5.265654207	Up	2.86E-06	6.48E-06
PGAM5	1.179510765	Up	3.45E-05	6.29E-05
PGAP2	4.839201327	Up	6.46E-05	0.000110902
PGBD2	4.741590531	Up	0.000118566	0.000190485
PGBD4	5.294131554	Up	2.25E-06	5.18E-06
PGBD5	3.358261891	Up	1.79E-05	3.46E-05
PHACTR1	1.647066246	Up	1.40E-09	5.13E-09
PHACTR4	1.583213252	Up	6.65E-08	1.96E-07

PHC3	4.704356805	Up	4.85E-146	2.94E-144
PHF10	1.974338712	Up	2.04E-31	2.54E-30
PHF14	2.895611985	Up	0.000639888	0.000857439
PHF21B	1.332074909	Up	3.52E-07	9.29E-07
PHF3	1.552641881	Up	0.000732612	0.000964809
PHF7	3.144162158	Up	8.44E-69	2.35E-67
PHKB	1.306877087	Up	6.10E-09	2.07E-08
PHLPP2	1.075460408	Up	1.19E-05	2.39E-05
PHRF1	6.520640084	Up	5.82E-13	2.93E-12
PHYKPL	1.61152999	Up	8.90E-07	2.19E-06
PI4KB	2.559692888	Up	3.05E-11	1.33E-10
PIAS2	7.143403006	Up	1.47E-18	1.06E-17
PIAS4	1.66251896	Up	5.55E-07	1.42E-06
PICALM	5.726119122	Up	3.53E-08	1.08E-07
PIGO	3.36500122	Up	0.000520835	0.000713086
PIGS	3.057092357	Up	5.63E-06	1.21E-05
PIGT	1.533535235	Up	1.69E-13	8.89E-13
PIK3C2A	1.943134245	Up	7.63E-20	5.93E-19
PIK3CG	6.495195067	Up	9.01E-13	4.47E-12
PIK3R4	1.047003539	Up	4.96E-05	8.73E-05
PIKFYVE	4.666577176	Up	0.000184567	0.000283063
PILRA	5.101486476	Up	1.04E-05	2.12E-05
PIP4K2A	2.097734341	Up	1.46E-06	3.47E-06
PIP4K2C	1.834699935	Up	1.54E-07	4.30E-07
PIP5K1A	1.542857984	Up	3.53E-18	2.50E-17
PIP5K1B	3.004624937	Up	0.000305949	0.000444199
PIP5K1C	7.887267986	Up	2.36E-28	2.65E-27
PITPNB	3.082627449	Up	7.89E-11	3.29E-10
PITPNM1	1.065801071	Up	8.21E-16	5.06E-15
PITPNM2	5.69268093	Up	5.06E-08	1.52E-07
PITRM1	4.434309212	Up	1.77E-06	4.15E-06
PJA1	1.288836178	Up	2.02E-22	1.77E-21
PJA2	1.874228299	Up	8.59E-05	0.000142662
PKD1	1.285845953	Up	2.66E-101	1.09E-99
PKLR	1.642054857	Up	1.11E-11	5.04E-11
PKM	1.423920549	Up	4.35E-114	2.04E-112
PKP4	2.977718244	Up	8.90E-08	2.57E-07
PLA2G10	5.004624937	Up	2.12E-05	4.03E-05
PLA2G2D	4.95902807	Up	2.91E-05	5.39E-05
PLAC4	4.642054857	Up	0.000212366	0.000320567
PLAT	4.642054857	Up	0.000212366	0.000320567
PLCB4	3.922162776	Up	9.36E-05	0.000153908
PLCD1	6.419662436	Up	3.17E-12	1.50E-11
PLCH1	4.842792356	Up	6.31E-05	0.000108521

PLCL1	2.51277184	Up	1.17E-05	2.34E-05
PLD1	6.227017358	Up	6.20E-11	2.61E-10
plekhb2	4.991743645	Up	2.32E-05	4.38E-05
PLEKHG5	2.071109959	Up	2.97E-15	1.77E-14
PLEKHM3	1.349093434	Up	0.000565025	0.000766478
PLK1	1.150727429	Up	6.93E-07	1.74E-06
PLOD3	2.893593624	Up	2.95E-05	5.46E-05
PMM2	1.792570359	Up	8.34E-06	1.73E-05
PMS1	6.294131554	Up	2.28E-11	1.01E-10
PNPLA6	3.325812998	Up	9.60E-12	4.38E-11
PNPLA8	4.834699935	Up	6.65E-05	0.000113504
POC1B	3.642054857	Up	0.000548719	0.000746049
PODXL2	1.563274259	Up	4.79E-10	1.83E-09
POGLUT1	4.535139653	Up	0.000382164	0.000541831
POGZ	5.629115802	Up	9.86E-08	2.83E-07
POLB	1.15662803	Up	4.54E-07	1.18E-06
POLD2	1.717117252	Up	2.66E-09	9.39E-09
POLD3	1.132380484	Up	0.000655816	0.000877175
poldip2	1.260257437	Up	6.97E-22	5.98E-21
POLDIP3	6.122689286	Up	2.72E-10	1.07E-09
POLH	1.038242965	Up	8.21E-05	0.000137052
POLL	7.5631647	Up	1.36E-23	1.26E-22
POLR1B	5.642054857	Up	8.63E-08	2.50E-07
POLR2B	4.922162776	Up	3.75E-05	6.77E-05
POLR2H	6.664042129	Up	4.41E-14	2.42E-13
POLR2J	3.142272737	Up	0.000101898	0.000166309
POLR2K	3.004624937	Up	0.000305949	0.000444199
polr2m	4.832895438	Up	6.73E-05	0.000114708
POLR3E	6.674407634	Up	3.63E-14	2.00E-13
POLR3H	4.651299642	Up	0.000201475	0.000306819
POMGNT1	3.772729043	Up	0.000122797	0.000196695
POT1	4.535139653	Up	0.000382164	0.000541831
POU3F1	2.271217162	Up	3.15E-05	5.79E-05
POU5F1	2.458710188	Up	1.50E-10	6.07E-10
PPARA	1.910534044	Up	0.000137562	0.000217912
PPARD	5.123796638	Up	8.82E-06	1.82E-05
PPARG	1.422235232	Up	0.000384526	0.000544925
PPEF1	2.729404992	Up	9.47E-18	6.59E-17
PPFIA2	1.345674219	Up	0.00010428	0.000169938
PPFIBP1	2.373096128	Up	1.15E-08	3.78E-08

PPHLN1	5.368263283	Up	1.19E-06	2.87E-06
PPIL6	4.530693748	Up	0.000391292	0.000553923
PPIP5K1	1.823920096	Up	0.000336497	0.000484004
PPM1B	5.685199349	Up	5.48E-08	1.64E-07
PPME1	6.433422435	Up	2.53E-12	1.21E-11
PPP1CC	4.814725235	Up	7.55E-05	0.000126989
PPP1R14B	2.419662436	Up	5.36E-06	1.15E-05
PPP2R1B	1.35998767	Up	4.32E-06	9.45E-06
PPP2R2B	1.591438598	Up	4.14E-05	7.40E-05
PPP2R5C	1.588480764	Up	2.30E-11	1.01E-10
PPP2R5E	2.950177153	Up	1.70E-05	3.31E-05
PPP3CC	3.755484972	Up	5.19E-05	9.09E-05
PPP4C	2.00276106	Up	4.47E-08	1.35E-07
PPP4R2	5.25625988	Up	3.09E-06	6.95E-06
PPP4R3B	4.004624937	Up	1.05E-08	3.47E-08
PPP6C	2.953225784	Up	0.000436476	0.000611515
PPP6R3	1.570839322	Up	6.18E-39	9.56E-38
pprc1	1.390586327	Up	2.49E-12	1.20E-11
PPT2	4.834699935	Up	6.65E-05	0.000113504
PPTC7	1.621296297	Up	1.25E-06	3.01E-06
ppwd1	2.400717773	Up	7.58E-22	6.49E-21
PRAME	1.634494866	Up	9.20E-06	1.89E-05
PRAMEF26	4.754993235	Up	0.000109316	0.000177425
PRC1	2.478921496	Up	7.01E-35	9.63E-34
PRDM10	4.637926972	Up	0.000217396	0.000327473
PRDM16	4.741590531	Up	0.000118566	0.000190485
PRDM2	2.575114119	Up	1.67E-71	4.86E-70
PRDM5	2.709169053	Up	0.000151978	0.00023744
PRDM7	2.174125803	Up	3.37E-05	6.17E-05
PRDX6	1.685944833	Up	4.10E-08	1.25E-07
PREPL	4.962333215	Up	2.85E-05	5.28E-05
PRH1	2.941969329	Up	7.61E-07	1.90E-06
PRICKLE1	3.892750706	Up	5.62E-94	2.15E-92
prkaa1	1.04280813	Up	0.00068336	0.000910631
PRKAR1B	5.097734341	Up	1.07E-05	2.18E-05
PRKCD	6.227017358	Up	6.20E-11	2.61E-10
prkcg	1.162401585	Up	2.47E-08	7.73E-08
PRKCH	1.743552047	Up	7.81E-17	5.14E-16
PRKCI	2.478556125	Up	0.000120687	0.000193534
PRKD3	1.091039688	Up	4.69E-12	2.20E-11

PRKDC	2.109062347	Up	1.13E-115	5.39E-114
PRKG1	6.717098439	Up	1.61E-14	9.09E-14
PRKRA	2.634478022	Up	0.000276884	0.000406642
PRKRIP1	2.419662436	Up	3.27E-05	5.99E-05
PROCA1	1.193937594	Up	4.25E-08	1.29E-07
PROM1	5.128217566	Up	8.53E-06	1.77E-05
prop1	3.642054857	Up	0.000548719	0.000746049
PRPF4B	1.783466247	Up	7.73E-05	0.00012975
PRR11	1.18833689	Up	0.000738093	0.000971181
PRR18	2.294131554	Up	2.43E-05	4.57E-05
PRR3	6.564252706	Up	2.72E-13	1.40E-12
PRR4	5.15662803	Up	6.86E-06	1.44E-05
PRR5-AR HGAP8	5.419662436	Up	7.50E-07	1.87E-06
PRR5L	2.740490916	Up	3.99E-09	1.38E-08
PRRC1	2.193781028	Up	8.03E-15	4.63E-14
PRRC2A	9.930940701	Up	2.28E-87	8.15E-86
PRRT2	3.478556125	Up	5.69E-06	1.22E-05
PRRX2	1.475157548	Up	7.36E-05	0.000124085
PRSS37	1.568718245	Up	5.03E-71	1.45E-69
PRSS46	2.027345013	Up	0.000119426	0.000191763
PRSS54	6.361707696	Up	8.03E-12	3.69E-11
PRTG	4.765437273	Up	7.92E-15	4.57E-14
PRUNE1	5.817009065	Up	1.28E-08	4.16E-08
PSAP	2.309135978	Up	0.00050462	0.000695617
PSD2	3.922162776	Up	9.36E-05	0.000153908
PSEN1	1.036113086	Up	7.58E-06	1.58E-05
PSENEN	3.741590531	Up	0.000303865	0.000442212
PSIP1	4.595985209	Up	0.000274853	0.000403849
PSMA4	2.444454897	Up	2.50E-05	4.70E-05
PSMC3IP	4.922162776	Up	3.75E-05	6.77E-05
PSMD2	1.550906969	Up	1.36E-09	5.00E-09
PSMD6	2.321826402	Up	4.42E-09	1.52E-08
PSMD8	1.484202688	Up	4.53E-12	2.13E-11
PSME3	6.773691374	Up	5.30E-15	3.10E-14
PTCH1	3.463406672	Up	6.55E-05	0.000112244
PTCHD1	3.642054857	Up	0.000548719	0.000746049
ptdss2	1.230125843	Up	9.55E-39	1.47E-37
PTER	6.118251358	Up	2.89E-10	1.14E-09
ptges3l	1.101335504	Up	6.88E-06	1.45E-05
PTGR2	3.004624937	Up	0.000305949	0.000444199
PTH2R	1.732820321	Up	8.96E-05	0.000148162
PTK2	4.727090961	Up	0.000129361	0.000206103
PTK2B	5.082627449	Up	1.20E-05	2.40E-05

PTMA	1.233837639	Up	1.32E-13	7.01E-13
PTOV1	5.504293686	Up	3.40E-07	9.01E-07
PTPA	5.007827369	Up	2.07E-05	3.96E-05
PTPMT1	8.840324484	Up	2.80E-48	5.38E-47
PTPN1	1.995313189	Up	5.50E-43	9.43E-42
PTPN20	1.827874816	Up	4.52E-14	2.48E-13
PTPN4	3.082627449	Up	1.10E-07	3.14E-07
PTPN6	2.480900065	Up	1.47E-11	6.58E-11
PTPRA	1.26536284	Up	8.17E-93	3.09E-91
PTPRB	2.200807966	Up	6.20E-07	1.57E-06
PTPRJ	1.831486805	Up	7.27E-08	2.13E-07
PTPRK	4.15662803	Up	1.62E-05	3.16E-05
PTPRM	8.765437273	Up	2.53E-46	4.66E-45
PTPRN2	1.407885268	Up	6.33E-09	2.14E-08
PTTG1IP	1.214660768	Up	1.15E-22	1.02E-21
PUM1	10.12066253	Up	1.85E-64	4.83E-63
PUM2	1.580098297	Up	5.06E-10	1.93E-09
PVR	3.968099061	Up	6.78E-05	0.000115465
PXK	1.967276778	Up	0.000624871	0.000839212
PXT1	3.607289439	Up	3.80E-09	1.32E-08
PXYLP1	6.004624937	Up	1.30E-09	4.78E-09
PYCR2	3.741590531	Up	0.000303865	0.000442212
QARS	5.087176149	Up	1.16E-05	2.34E-05
QKI	1.804049298	Up	2.60E-19	1.96E-18
QPCTL	1.445491878	Up	0.000581172	0.000785984
RAB11FIP1	1.736813999	Up	1.79E-05	3.47E-05
RAB11FIP2	2.273990616	Up	1.47E-06	3.50E-06
RAB15	4.834699935	Up	6.65E-05	0.000113504
RAB1A	1.482113944	Up	1.63E-09	5.91E-09
RAB21	1.165865704	Up	0.000545623	0.000743383
rab22a	1.516253833	Up	0.000100928	0.000164848
RAB27A	3.113428148	Up	0.000137766	0.000218116
RAB2A	6.541782927	Up	4.03E-13	2.05E-12
RAB34	4.620250487	Up	0.000240158	0.000358561
RAB40C	5.419662436	Up	7.50E-07	1.87E-06
RAB5B	6.223578274	Up	6.52E-11	2.74E-10
RAB6A	5.431038799	Up	6.76E-07	1.70E-06
rab6c	3.065563552	Up	0.000307225	0.00044586
rab9b	2.709169053	Up	0.000151978	0.00023744
RABGAP1L	2.311496457	Up	9.38E-05	0.000154212
RABGEF	1.147143566	Up	3.15E-08	9.71E-08

1				
RABGGT A	2.267659342	Up	0.000149652	0.000234247
RABL2A	6.574548038	Up	2.26E-13	1.18E-12
RABL2B	4.91450469	Up	3.94E-05	7.08E-05
RACGAP 1	3.312747232	Up	3.99E-15	2.35E-14
RAD17	2.538219046	Up	0.000736527	0.00096944
RAD23B	1.29271784	Up	1.60E-13	8.44E-13
RAD51D	2.294699209	Up	5.67E-06	1.22E-05
RAD52	2.820697076	Up	1.37E-14	7.79E-14
RAD9A	4.676002189	Up	3.83E-20	3.02E-19
RAG1	1.992241212	Up	3.04E-07	8.11E-07
RAI14	1.787176468	Up	6.79E-11	2.85E-10
RALB	1.019124506	Up	0.000421816	0.000592868
RALGAP A1	2.363760451	Up	4.68E-06	1.02E-05
RALGAP A2	1.545182257	Up	8.45E-10	3.16E-09
RALGAP B	8.434612777	Up	1.50E-38	2.29E-37
RALGPS2	1.610484778	Up	6.69E-10	2.53E-09
RAN	4.922162776	Up	3.75E-05	6.77E-05
RANBP17	2.177587649	Up	1.01E-24	9.81E-24
RANBP2	1.226556048	Up	8.13E-146	4.91E-144
RANBP3	5.044153301	Up	1.60E-05	3.12E-05
RANBP6	1.003407695	Up	1.49E-08	4.82E-08
RANGRF	3.075014265	Up	0.000183925	0.000282148
RAPGEF2	3.066542901	Up	8.70E-08	2.52E-07
RAPGEF3	7.105228878	Up	3.71E-18	2.63E-17
RAPGEF6	1.653514029	Up	1.03E-32	1.32E-31
RASAL2	1.632762857	Up	5.92E-26	6.04E-25
RASGEF1 A	4.419662436	Up	0.000691126	0.00091553
RASSF8	1.796883541	Up	0.000222164	0.000333808
RB1	1.447676812	Up	0.000107883	0.0001753
RBCK1	1.45282002	Up	1.08E-17	7.50E-17
RBL2	1.372069746	Up	2.53E-45	4.60E-44
rbm12	6.963982952	Up	9.66E-17	6.32E-16
RBM23	5.523439803	Up	2.83E-07	7.60E-07
RBM39	2.310044272	Up	3.74E-08	1.15E-07
RBM6	2.129430018	Up	4.20E-22	3.63E-21
RBM7	1.867225839	Up	5.00E-06	1.08E-05
RBPMS	4.576706146	Up	0.000305521	0.000444052

RCBTB2	4.814725235	Up	7.55E-05	0.000126989
RCOR3	6.107096444	Up	3.36E-10	1.31E-09
RECQL5	5.182050627	Up	5.89E-22	5.07E-21
REXO4	1.2235842	Up	1.83E-10	7.34E-10
RFC2	2.610803923	Up	4.21E-08	1.28E-07
RFESD	2.057092357	Up	8.61E-06	1.78E-05
RFWD3	3.571665529	Up	6.68E-09	2.26E-08
RFX2	1.433337373	Up	4.08E-08	1.24E-07
RFX3	3.053123454	Up	1.75E-13	9.20E-13
RFX4	1.409837961	Up	5.42E-72	1.59E-70
RGL1	1.428872559	Up	1.56E-05	3.06E-05
RGL4	1.057092357	Up	0.000231819	0.000347244
RGPD2	1.723814454	Up	4.21E-123	2.16E-121
RGPD3	2.221754378	Up	5.01E-82	1.67E-80
RGPD4	2.055279515	Up	3.84E-22	3.33E-21
RGPD5	10.96074614	Up	1.27E-150	8.02E-149
RGS12	1.122053837	Up	2.88E-12	1.38E-11
RGS2	3.642054857	Up	0.000548719	0.000746049
RGS22	1.562184049	Up	3.28E-50	6.56E-49
RGS3	3.808482414	Up	5.34E-05	9.32E-05
RGS4	2.305887396	Up	0.000517668	0.000709193
RGS6	5.911088004	Up	4.19E-09	1.45E-08
RGS9BP	5.865918666	Up	7.21E-09	2.42E-08
RHBDD1	2.999661472	Up	4.82E-111	2.21E-109
rhbdd2	1.483454924	Up	5.87E-21	4.81E-20
RHBG	2.073082087	Up	5.47E-12	2.55E-11
RHOBTB1	8.67018194	Up	6.05E-44	1.06E-42
RHOC	5.741590531	Up	2.98E-08	9.22E-08
RICTOR	4.834699935	Up	6.65E-05	0.000113504
rilpl2	2.571665529	Up	0.000446586	0.000623907
RIMS2	5.15662803	Up	6.86E-06	1.44E-05
ring1	2.189542653	Up	1.78E-16	1.15E-15
RIPK2	3.922162776	Up	9.36E-05	0.000153908
RLIM	1.503870528	Up	0.000126504	0.000202095
RMDN2	2.291068041	Up	2.51E-05	4.71E-05
RMI2	1.208158331	Up	0.000420819	0.000591581
RMND5B	4.58530644	Up	0.000291484	0.000426187
RNASE1	4.922162776	Up	3.75E-05	6.77E-05
RNASE11	1.279484778	Up	1.32E-06	3.15E-06
RNASEH1	1.904908937	Up	6.12E-06	1.30E-05
RNASEH2B	4.419662436	Up	0.000691126	0.00091553

RNASEK	1.419662436	Up	1.21E-09	4.44E-09
RNF111	1.687317567	Up	1.44E-47	2.73E-46
RNF139	1.446134647	Up	2.62E-36	3.74E-35
RNF14	7.330075316	Up	1.18E-20	9.56E-20
RNF17	2.314551095	Up	9.19E-14	4.93E-13
RNF183	2.893593624	Up	2.95E-05	5.46E-05
RNF187	1.096512706	Up	0.000136078	0.000215834
RNF19B	8.066793758	Up	1.09E-14	6.21E-14
RNF214	2.059443339	Up	3.71E-15	2.19E-14
RNF216	1.066828156	Up	5.45E-29	6.25E-28
RNF38	4.95203285	Up	1.19E-30	1.44E-29
RNF39	4.584968261	Up	6.00E-21	4.91E-20
RNF41	1.709578851	Up	0.000323452	0.000466939
RNF6	3.243767102	Up	7.11E-15	4.11E-14
RNF7	1.400308352	Up	8.09E-11	3.37E-10
RNFT2	1.876040731	Up	8.29E-18	5.79E-17
RNH1	1.28238943	Up	0.000222263	0.000333933
RNMT	5.589587437	Up	1.47E-07	4.13E-07
RNPS1	2.551253863	Up	2.04E-05	3.91E-05
ROBO1	5.419662436	Up	7.50E-07	1.87E-06
ROGDI	1.726554812	Up	1.24E-08	4.04E-08
RPL13	1.739648073	Up	1.18E-25	1.19E-24
RPL14	3.922162776	Up	9.36E-05	0.000153908
RPL15	1.558675168	Up	0.000325091	0.000468939
RPL17	3.199736971	Up	7.62E-09	2.56E-08
RPL32	1.419532458	Up	2.49E-08	7.78E-08
RPL37	1.951079119	Up	0.000175549	0.000270367
RPL39	1.584721682	Up	7.90E-07	1.97E-06
RPL7A	1.210365046	Up	2.86E-05	5.31E-05
RPL8	3.419662436	Up	2.93E-12	1.40E-11
RPP38	4.437584344	Up	0.00063215	0.000848081
rps15a	4.642054857	Up	0.000212366	0.000320567
RPS25	1.478556125	Up	0.000114673	0.000185353
RPS27A	4.241372651	Up	8.01E-06	1.66E-05
RPS4X	3.834699935	Up	0.000168506	0.000260719
RPS6KA5	4.575959474	Up	5.88E-09	2.00E-08
RPS6KC1	5.491196306	Up	3.86E-07	1.01E-06
RPS8	4.004624937	Up	5.21E-05	9.11E-05
RPTOR	1.505863471	Up	8.27E-06	1.71E-05
RRP12	1.349273108	Up	8.04E-05	0.000134463
RSPH14	1.038233329	Up	0.000313269	0.000453896
RSPRY1	5.377577035	Up	1.09E-06	2.66E-06
RSRC1	1.584721682	Up	0.000480226	0.000665124
RSU1	1.705966621	Up	1.26E-13	6.67E-13

RTEL1	4.966454026	Up	2.77E-05	5.15E-05
RTFDC1	6.811979859	Up	2.45E-15	1.46E-14
RTN3	4.450587009	Up	0.000592168	0.000799993
RTN4	5.739285537	Up	1.07E-36	1.55E-35
RTN4RL2	3.741590531	Up	3.25E-07	8.64E-07
RTTN	1.992241212	Up	3.04E-07	8.11E-07
RUBCNL	2.02555337	Up	6.21E-14	3.37E-13
RUFY1	5.242732401	Up	3.45E-06	7.69E-06
RUFY2	3.758748331	Up	2.41E-27	2.59E-26
RUNDC3A	2.913957854	Up	2.08E-08	6.56E-08
RUNDC3B	4.642054857	Up	0.000212366	0.000320567
RUNX1	1.712071444	Up	0.000343638	0.00049316
RXFP2	5.358261891	Up	1.30E-06	3.11E-06
RXRB	2.606350251	Up	5.70E-50	1.13E-48
RYBP	5.004624937	Up	2.12E-05	4.03E-05
RYK	5.108215825	Up	9.93E-06	2.03E-05
RYR2	1.734781127	Up	7.83E-84	2.67E-82
RYR3	2.406017291	Up	1.80E-48	3.48E-47
S100PBP	1.167275274	Up	0.000178197	0.000274196
S1PR3	3.834699935	Up	0.000168506	0.000260719
SACS	4.419662436	Up	0.000691126	0.00091553
SAMD11	1.486776632	Up	0.000279762	0.000410566
SAMD4B	4.033608945	Up	5.85E-22	5.03E-21
SAMD8	1.419662436	Up	2.67E-05	4.98E-05
SAP130	3.768422024	Up	1.97E-97	7.79E-96
SAP30BP	6.120102154	Up	2.82E-10	1.11E-09
SARS2	1.834033885	Up	9.28E-23	8.28E-22
SAV1	2.127108943	Up	2.65E-43	4.58E-42
SAXO1	1.086333613	Up	1.57E-05	3.08E-05
SAYSD1	1.694979043	Up	5.10E-06	1.10E-05
scaf8	1.054268893	Up	2.23E-10	8.87E-10
SCAMP3	3.834699935	Up	0.000168506	0.000260719
SCAMP5	3.834699935	Up	0.000168506	0.000260719
SCMH1	5.741590531	Up	2.98E-08	9.22E-08
SCN10A	5.963982952	Up	2.18E-09	7.80E-09
SCN3A	1.64311139	Up	9.27E-126	4.85E-124
SCN5A	1.118006736	Up	6.14E-07	1.56E-06
SCNM1	1.55563671	Up	3.09E-10	1.21E-09
SCP2	1.97866571	Up	6.19E-05	0.00010666
SCP2D1	1.19459588	Up	4.86E-05	8.55E-05
SCX	4.082627449	Up	2.90E-05	5.37E-05
SDAD1	1.120595299	Up	0.000168381	0.000260719

SDCCAG 3	2.237621913	Up	0.000591356	0.000798945
SEC14L1	2.98132343	Up	1.66E-16	1.07E-15
SEC23A	1.664774934	Up	5.65E-09	1.93E-08
SEC24B	2.691541211	Up	0.000454825	0.000634084
SEC61A2	5.082627449	Up	1.20E-05	2.40E-05
SECISBP 2L	6.238649299	Up	5.23E-11	2.22E-10
SEH1L	1.035826018	Up	0.000180277	0.000277029
SELENOI	2.972203459	Up	1.97E-08	6.24E-08
SELPLG	6.994167664	Up	4.92E-17	3.28E-16
SEMA4A	5.004624937	Up	2.12E-05	4.03E-05
SEMA4G	1.28350966	Up	1.12E-08	3.66E-08
SERINC3	2.351275461	Up	3.78E-14	2.09E-13
SERINC5	1.514202376	Up	8.03E-05	0.000134383
serpina1	4.941550732	Up	3.28E-05	6.01E-05
SERPINB 6	1.682818711	Up	1.03E-27	1.12E-26
SERTAD3	1.218507478	Up	2.30E-33	3.03E-32
SETBP1	1.05781718	Up	0.000334942	0.000481941
SETD3	1.632779372	Up	4.37E-21	3.61E-20
SETD9	6.701360686	Up	2.18E-14	1.22E-13
setmar	1.697196411	Up	0.000137314	0.00021755
SETX	1.322539969	Up	2.13E-21	1.78E-20
SF1	1.341115472	Up	3.17E-24	3.01E-23
SF3B2	1.259197764	Up	0.000568511	0.000769977
sfmbt1	1.02056648	Up	0.000100861	0.000164769
SFTPC	5.332950803	Up	1.62E-06	3.81E-06
SGIP1	4.642054857	Up	0.000212366	0.000320567
SGK3	5.194361658	Up	5.10E-06	1.10E-05
SGMS2	5.294131554	Up	2.25E-06	5.18E-06
SGO2	2.282158912	Up	1.39E-08	4.52E-08
SGPL1	5.004624937	Up	2.12E-05	4.03E-05
SGSM1	5.456458792	Up	5.34E-07	1.37E-06
SH2D4A	2.049712826	Up	3.35E-08	1.03E-07
SH3BGR	2.212341574	Up	5.99E-11	2.53E-10
SH3GL2	1.528596807	Up	3.38E-05	6.17E-05
SH3GLB1	5.453525888	Up	5.49E-07	1.40E-06
SH3YL1	1.950177153	Up	0.000722703	0.000953274
SHC1	1.314934061	Up	2.42E-07	6.58E-07
SHF	5.923011171	Up	3.63E-09	1.26E-08
SHKBP1	2.004624937	Up	8.38E-08	2.44E-07
SIDT1	1.571665529	Up	3.34E-07	8.84E-07
siglec10	3.137565116	Up	2.35E-08	7.39E-08

SIGLEC5	2.406296318	Up	1.63E-14	9.20E-14
SIGMAR1	3.741590531	Up	3.25E-07	8.64E-07
SIL1	1.801533071	Up	3.77E-05	6.81E-05
SIN3A	1.954033507	Up	0.000373815	0.000532999
SIN3B	6.900789126	Up	3.83E-16	2.41E-15
SIPA1	1.697196411	Up	0.000137314	0.00021755
SIRPG	4.535139653	Up	0.000382164	0.000541831
SIRT1	4.85972873	Up	5.66E-05	9.82E-05
SIRT5	2.815709482	Up	2.99E-05	5.53E-05
SKA1	1.012055401	Up	7.38E-05	0.000124435
SKAP2	7.021755025	Up	2.63E-17	1.78E-16
SKOR2	4.384760625	Up	7.68E-08	2.24E-07
SKP1	1.876612058	Up	2.05E-13	1.07E-12
SLA	2.766382992	Up	4.58E-07	1.19E-06
SLBP	2.348961442	Up	6.62E-17	4.38E-16
SLC10A6	2.213211558	Up	2.17E-07	5.93E-07
SLC12A5	3.419662436	Up	1.01E-05	2.06E-05
SLC12A6	5.067360692	Up	1.35E-05	2.67E-05
SLC13A5	5.589587437	Up	1.47E-07	4.13E-07
SLC15A2	4.380557687	Up	0.000110307	0.000178901
SLC16A3	5.147945787	Up	7.33E-06	1.53E-05
SLC16A4	4.928936005	Up	3.58E-05	6.51E-05
SLC20A2	2.971684286	Up	1.45E-22	1.28E-21
SLC22A12	5.741590531	Up	2.98E-08	9.22E-08
SLC22A15	1.453609768	Up	0.000412519	0.00058116
SLC22A16	2.349273108	Up	2.43E-06	5.57E-06
SLC22A18	1.716298245	Up	1.72E-05	3.34E-05
SLC23A3	2.168537775	Up	6.38E-09	2.16E-08
SLC24A2	4.535139653	Up	0.000382164	0.000541831
SLC24A4	6.387753188	Up	5.31E-12	2.48E-11
SLC25A1	3.829344609	Up	2.05E-21	1.71E-20
SLC25A10	5.584770421	Up	1.55E-07	4.32E-07
SLC25A30	6.63093427	Up	8.15E-14	4.39E-13
SLC25A35	1.360084873	Up	1.02E-05	2.07E-05
SLC25A37	2.680497699	Up	1.45E-08	4.68E-08

SLC25A38	5.589587437	Up	1.47E-07	4.13E-07
SLC25A40	1.169884127	Up	5.51E-09	1.88E-08
SLC25A46	2.885439068	Up	1.66E-15	1.00E-14
SLC25A52	4.289533842	Up	5.28E-06	1.14E-05
SLC26A8	6.227017358	Up	6.20E-11	2.61E-10
SLC2A11	4.801868543	Up	8.18E-05	0.000136618
slc2a14	7.517132776	Up	5.56E-23	5.00E-22
SLC2A8	4.771102313	Up	9.91E-05	0.000162049
SLC2A9	5.358261891	Up	1.30E-06	3.11E-06
SLC30A3	1.493663017	Up	1.66E-13	8.70E-13
SLC30A6	5.462306773	Up	5.06E-07	1.30E-06
SLC30A9	4.741590531	Up	0.000118566	0.000190485
SLC34A2	2.97988599	Up	1.43E-10	5.82E-10
SLC35A3	4.293854831	Up	2.18E-12	1.05E-11
SLC35A4	1.18069579	Up	3.89E-14	2.14E-13
SLC35A5	1.153710547	Up	1.47E-12	7.20E-12
SLC35C2	4.731940361	Up	0.000125656	0.000200899
SLC35F3	1.419662436	Up	0.000385837	0.000546571
SLC35G2	3.376957985	Up	1.50E-05	2.96E-05
SLC36A1	1.442392939	Up	1.38E-05	2.73E-05
SLC36A3	9.310433366	Up	1.78E-62	4.49E-61
SLC37A3	2.419662436	Up	3.27E-05	5.99E-05
SLC38A10	4.023583094	Up	7.61E-07	1.90E-06
SLC38A3	1.294131554	Up	6.21E-05	0.000106814
SLC39A10	2.305106186	Up	5.39E-09	1.84E-08
SLC39A3	1.360768747	Up	1.38E-05	2.72E-05
SLC39A7	5.296752254	Up	2.20E-06	5.09E-06
SLC43A1	1.025929542	Up	0.000130488	0.000207795
SLC43A3	5.004624937	Up	2.12E-05	4.03E-05
SLC44A5	3.797583911	Up	2.79E-23	2.55E-22
SLC45A4	1.031963645	Up	0.000253356	0.000376537
SLC4A3	1.020970083	Up	5.71E-05	9.89E-05
SLC4A7	2.455769944	Up	0.000186215	0.000285421
SLC4A8	1.523751429	Up	5.26E-14	2.87E-13
SLC5A1	4.419662436	Up	0.000691126	0.00091553
SLC5A9	1.464331485	Up	4.04E-12	1.91E-11
SLC6A12	1.228614143	Up	4.86E-07	1.26E-06
SLC6A20	1.017833714	Up	6.52E-06	1.38E-05

SLC6A8	3.160257375	Up	7.57E-79	2.43E-77
SLC6A9	4.848162195	Up	6.10E-05	0.000105183
SLC7A13	3.227017358	Up	1.21E-08	3.94E-08
SLC7A6	4.616846359	Up	2.21E-13	1.15E-12
SLC7A7	4.114813545	Up	8.94E-75	2.71E-73
SLC7A9	3.419662436	Up	1.01E-05	2.06E-05
SLC8A1	2.203933745	Up	6.54E-05	0.000112114
SLC9A4	1.01152126	Up	1.74E-07	4.84E-07
SLC9B1	6.004624937	Up	1.30E-09	4.78E-09
SLCO1A2	5.205561675	Up	4.66E-06	1.01E-05
SLF2	3.88280531	Up	1.56E-14	8.85E-14
SLTM	3.2815863	Up	5.66E-41	9.23E-40
SMAD1	1.7394639	Up	2.03E-41	3.36E-40
SMAD2	1.080754204	Up	0.000148071	0.000232004
SMAD5	1.109706983	Up	4.11E-09	1.42E-08
SMAD6	1.709169053	Up	5.85E-05	0.000101136
SMARCA2	6.988099963	Up	5.64E-17	3.74E-16
SMARCA4	1.557782816	Up	1.92E-08	6.10E-08
SMARCB1	1.334151923	Up	7.49E-07	1.87E-06
SMARCC1	1.410121669	Up	9.44E-104	3.95E-102
SMARCE1	2.358261891	Up	0.000340113	0.000488628
SMC2	5.534584664	Up	2.54E-07	6.88E-07
SMCHD1	1.559304417	Up	6.04E-35	8.32E-34
SMG7	3.801665156	Up	3.42E-09	1.19E-08
SMIM12	4.448231588	Up	0.000599242	0.000808927
SMIM14	1.979203449	Up	9.54E-13	4.73E-12
SMIM2	1.793915216	Up	2.66E-06	6.05E-06
SMPD1	8.984345453	Up	2.90E-52	6.07E-51
SMURF1	5.788896246	Up	1.76E-08	5.61E-08
SNAP91	1.233061334	Up	0.000168907	0.000261293
SNRK	1.059992247	Up	6.04E-08	1.79E-07
SNRNP40	3.741590531	Up	0.000303865	0.000442212
SNRPD3	1.283458052	Up	8.55E-07	2.11E-06
SNTG1	3.037117657	Up	0.000242686	0.000362051
SNTN	4.634823288	Up	0.000221248	0.000332739
SNUPN	4.535139653	Up	0.000382164	0.000541831
SNW1	2.019124506	Up	1.61E-06	3.81E-06
SNX13	1.635213386	Up	1.00E-28	1.14E-27
SNX14	4.834699935	Up	6.65E-05	0.000113504

SNX3	5.642054857	Up	8.63E-08	2.50E-07
SNX30	2.419662436	Up	2.51E-08	7.83E-08
SNX5	2.709169053	Up	0.000151978	0.00023744
SNX9	4.922162776	Up	3.75E-05	6.77E-05
SOCS5	2.642054857	Up	2.48E-09	8.81E-09
SOD2	5.247481461	Up	3.32E-06	7.42E-06
SOGA3	1.465544169	Up	7.19E-05	0.000121785
SON	2.423328753	Up	1.80E-07	4.97E-07
sorbs1	3.420413644	Up	2.31E-08	7.26E-08
SORT1	3.017392233	Up	1.97E-20	1.58E-19
SOX30	2.126806472	Up	0.000219373	0.000330236
SOX5	2.449998514	Up	0.000155782	0.000242863
SOX6	1.123955085	Up	6.40E-07	1.62E-06
SP1	4.966454026	Up	2.77E-05	5.15E-05
SP140	3.218749742	Up	5.98E-05	0.00010322
SP3	4.459268781	Up	1.15E-27	1.25E-26
SP7	6.542059067	Up	4.01E-13	2.05E-12
SPACA3	5.009425923	Up	2.05E-05	3.92E-05
SPACA9	1.103777675	Up	0.000341212	0.000489934
SPAG16	2.126256457	Up	3.27E-17	2.20E-16
SPAG9	1.543552607	Up	9.18E-10	3.43E-09
SPATA12	1.108129512	Up	5.42E-26	5.53E-25
SPATA13	4.575627495	Up	0.000307323	0.000445973
SPATA22	1.660670535	Up	0.000209433	0.000317582
SPATA33	3.834699935	Up	8.67E-09	2.89E-08
SPATA6	2.857896135	Up	1.92E-38	2.93E-37
SPATA6L	1.436150559	Up	5.53E-07	1.41E-06
SPATA7	5.210434474	Up	4.48E-06	9.79E-06
SPATC1	1.750057109	Up	1.70E-17	1.16E-16
SPATC1L	2.132995212	Up	8.43E-10	3.16E-09
SPATS2	2.775942036	Up	6.54E-11	2.75E-10
SPATS2L	1.629944209	Up	1.77E-07	4.89E-07
SPDL1	1.320126762	Up	0.000179559	0.000276
SPECC1	3.247943197	Up	3.74E-11	1.61E-10
SPECC1L	1.341211303	Up	1.43E-11	6.42E-11
SPEF2	1.802599092	Up	1.32E-05	2.63E-05
SPERT	5.375719088	Up	1.11E-06	2.70E-06
SPESP1	1.009197666	Up	0.000330968	0.000476598
SPG11	3.621296297	Up	1.92E-14	1.08E-13
SPHAR	5.066593097	Up	1.35E-05	2.68E-05
SPHK2	5.066593097	Up	1.35E-05	2.68E-05
SPIDR	1.794057951	Up	1.58E-07	4.40E-07
SPIRE1	7.003021051	Up	4.03E-17	2.69E-16
SPNS1	4.834699935	Up	6.65E-05	0.000113504

SPON2	6.082627449	Up	4.68E-10	1.80E-09
spop	1.946368164	Up	0.00036258	0.000518074
SPPL3	1.43743307	Up	3.44E-17	2.31E-16
SPRED1	1.001235246	Up	1.84E-06	4.29E-06
SPTSSB	4.834699935	Up	6.65E-05	0.000113504
SQRDL	1.287230176	Up	2.61E-28	2.92E-27
SQSTM1	2.192487447	Up	9.74E-06	1.99E-05
SRBD1	3.642054857	Up	0.000548719	0.000746049
SREBF1	5.478556125	Up	4.35E-07	1.13E-06
SRR	7.452497961	Up	3.77E-22	3.27E-21
SRRM1	2.884255275	Up	1.81E-292	2.08E-290
SRRT	6.335793704	Up	2.44E-18	1.74E-17
SRSF10	1.34156744	Up	4.48E-14	2.46E-13
SRSF11	5.879094055	Up	6.16E-09	2.09E-08
SS18	1.170284988	Up	6.85E-05	0.000116594
SSBP3	1.045759973	Up	4.01E-16	2.52E-15
SSR3	2.314602977	Up	1.58E-06	3.73E-06
SSU72	1.100980001	Up	7.22E-09	2.43E-08
SSUH2	2.834699935	Up	5.11E-05	8.96E-05
ST3GAL3	5.69268093	Up	5.06E-08	1.52E-07
ST6GAL NAC6	3.184914273	Up	4.30E-10	1.66E-09
STAB1	1.062727891	Up	3.58E-08	1.10E-07
STAC3	4.933153181	Up	3.48E-05	6.34E-05
STAG1	2.057092357	Up	8.61E-06	1.78E-05
STAG3	2.233848593	Up	1.94E-60	4.70E-59
STAMBP	2.259075299	Up	9.90E-09	3.27E-08
STARD13	2.846213923	Up	1.51E-20	1.21E-19
STAT2	1.89342883	Up	2.82E-08	8.74E-08
STAT4	3.04175181	Up	3.01E-09	1.06E-08
STAT6	5.61763264	Up	1.11E-07	3.16E-07
STAU1	5.249844774	Up	1.58E-31	1.97E-30
STEAP1B	4.407589604	Up	0.000733516	0.000965825
STIL	7.15662803	Up	1.06E-18	7.74E-18
STIP1	7.993965818	Up	4.11E-30	4.89E-29
STK32A	4.862384812	Up	5.56E-05	9.67E-05
STK33	5.664549495	Up	6.82E-08	2.01E-07
STK38	1.725514464	Up	4.10E-07	1.07E-06
STK40	5.790291727	Up	1.73E-08	5.53E-08
STOML2	7.669486985	Up	4.62E-25	4.53E-24
stpg1	2.140582189	Up	8.32E-10	3.12E-09
STRA6	4.26786071	Up	1.93E-10	7.74E-10
STRBP	1.328856152	Up	3.67E-10	1.43E-09
STRN4	8.053868456	Up	3.81E-31	4.69E-30

STUM	4.922162776	Up	3.75E-05	6.77E-05
STX5	1.399929377	Up	9.52E-10	3.54E-09
STX6	2.074320161	Up	6.53E-07	1.65E-06
STXBP5	1.493183706	Up	9.22E-09	3.06E-08
STYX	1.617768803	Up	1.13E-09	4.16E-09
SUCLG2	3.372617184	Up	1.57E-05	3.07E-05
SUCO	2.402689357	Up	1.13E-05	2.27E-05
SULT1A1	4.922162776	Up	3.75E-05	6.77E-05
SUMF1	6.181656825	Up	1.19E-10	4.88E-10
SUN1	5.924706465	Up	3.55E-09	1.24E-08
SUN3	3.286240768	Up	3.82E-07	1.00E-06
SUPT3H	5.69268093	Up	5.06E-08	1.52E-07
SUPT5H	1.419662436	Up	0.000605305	0.000816033
SUPV3L1	6.082627449	Up	4.68E-10	1.80E-09
SURF4	4.535139653	Up	0.000382164	0.000541831
SUSD1	2.282158912	Up	1.02E-15	6.25E-15
SUZ12	1.425147975	Up	0.000569476	0.000771182
SV2B	1.271220987	Up	2.35E-09	8.36E-09
SV2C	3.569848042	Up	8.02E-05	0.000134222
SVEP1	1.252232248	Up	9.38E-07	2.30E-06
SYCP2	5.185550741	Up	1.28E-10	5.24E-10
SYF2	4.834699935	Up	6.65E-05	0.000113504
SYNCRIP	4.978384975	Up	9.40E-12	4.30E-11
SYNE1	2.827015187	Up	4.47E-11	1.91E-10
SYNE2	3.483997078	Up	1.74E-08	5.57E-08
SYNPO	3.976055784	Up	1.30E-05	2.58E-05
SYNRG	1.414611735	Up	4.43E-05	7.86E-05
SYPL1	4.535139653	Up	0.000382164	0.000541831
SYT11	1.326553031	Up	0.000749683	0.000984751
SYTL3	4.535139653	Up	0.000382164	0.000541831
SZRD1	2.001181695	Up	1.13E-17	7.80E-17
TAB2	1.113919653	Up	0.000128035	0.00020428
TACR1	3.417318495	Up	0.000108754	0.000176612
TADA2A	2.61480002	Up	5.15E-11	2.19E-10
TADA3	1.892640067	Up	0.000198855	0.000303017
TAF1	4.420864181	Up	0.000687026	0.00091496
TAF1C	4.535417068	Up	1.20E-12	5.91E-12
TAGAP	4.224954891	Up	9.21E-06	1.89E-05
TAGLN2	5.227017358	Up	3.92E-06	8.66E-06
TANGO2	4.407589604	Up	0.000733516	0.000965825
TAOK2	2.028538803	Up	2.51E-16	1.60E-15
TAOK3	5.535139653	Up	2.53E-07	6.84E-07
TAPBP	1.432309812	Up	5.64E-07	1.44E-06
TARSL2	3.337200276	Up	1.97E-07	5.41E-07

tas1r2	2.057092357	Up	0.000280305	0.000411061
TAS2R4	1.478556125	Up	2.31E-06	5.31E-06
TASP1	1.699770355	Up	1.63E-05	3.19E-05
TATDN3	1.655642418	Up	1.47E-29	1.72E-28
TAX1BP1	1.036616864	Up	1.94E-05	3.73E-05
TBC1D1	3.440736057	Up	6.41E-27	6.81E-26
TBC1D12	2.057092357	Up	8.61E-06	1.78E-05
TBC1D13	5.004624937	Up	2.12E-05	4.03E-05
TBC1D14	3.188224875	Up	1.38E-13	7.29E-13
TBC1D17	5.004624937	Up	2.12E-05	4.03E-05
TBC1D21	3.057092357	Up	5.63E-06	1.21E-05
TBC1D24	5.09623075	Up	1.09E-05	2.20E-05
TBC1D25	1.407194958	Up	6.24E-08	1.85E-07
TBC1D2B	5.741590531	Up	2.98E-08	9.22E-08
TBC1D31	2.10771843	Up	0.000173302	0.00026746
TBC1D4	5.642054857	Up	8.63E-08	2.50E-07
TBCEL	1.039058434	Up	1.82E-07	5.04E-07
TBCK	2.178600803	Up	2.94E-29	3.40E-28
TBX5	2.250882495	Up	8.17E-05	0.000136383
TC2N	3.004624937	Up	9.80E-06	2.00E-05
TCAF2	2.053123454	Up	2.47E-06	5.65E-06
TCEA2	1.768062742	Up	8.84E-12	4.05E-11
TCEB2	1.320219374	Up	1.30E-06	3.11E-06
TCERG1L	3.004624937	Up	1.14E-08	3.74E-08
TCF4	3.594716113	Up	0.000560616	0.000761069
TCF7L2	5.652323193	Up	7.75E-08	2.26E-07
TCFL5	5.478556125	Up	4.35E-07	1.13E-06
TCOF1	6.815315571	Up	8.19E-15	4.72E-14
TCP10	3.765676149	Up	0.00028108	0.000412129
TCP11	1.749864398	Up	7.22E-62	1.80E-60
TDRD1	1.109706983	Up	1.58E-06	3.74E-06
TDRD10	1.943207379	Up	1.47E-06	3.49E-06
TDRD7	7.214598099	Up	2.47E-19	1.87E-18
TDRKH	2.058182994	Up	1.11E-10	4.58E-10
TECPR1	4.829279659	Up	6.88E-05	0.00011716
TEF	1.806685559	Up	6.89E-06	1.45E-05
TEK	1.335821309	Up	7.96E-05	0.000133336
TEKT3	1.289894561	Up	1.44E-10	5.86E-10
TENM2	2.536592186	Up	3.78E-16	2.38E-15
TENM3	1.544109807	Up	5.80E-10	2.21E-09
TEPP	4.922162776	Up	3.75E-05	6.77E-05
TERF2	2.148268962	Up	6.60E-12	3.06E-11
TESC	5.642054857	Up	8.63E-08	2.50E-07

TET2	1.885326008	Up	0.000481545	0.000666568
TEX15	2.298100456	Up	3.28E-23	2.98E-22
TEX2	3.818455954	Up	6.11E-101	2.50E-99
TEX264	1.020321776	Up	9.89E-08	2.84E-07
TEX29	2.407589604	Up	3.72E-05	6.74E-05
TEX35	5.478556125	Up	4.35E-07	1.13E-06
TEX36	1.650987982	Up	1.03E-07	2.96E-07
TEX37	1.144027993	Up	0.00073337	0.00096572
TEX38	4.927245677	Up	3.62E-05	6.58E-05
TFAP2A	4.623385646	Up	0.000235973	0.00035278
TFCP2L1	1.679725275	Up	3.76E-09	1.31E-08
TFDP2	4.599173486	Up	0.000270054	0.000397316
TFG	5.808311211	Up	1.41E-08	4.57E-08
TFRC	4.834699935	Up	6.65E-05	0.000113504
TGFB2	4.963982952	Up	3.17E-09	1.11E-08
TGFBR3	1.966670487	Up	1.37E-31	1.71E-30
TGIF1	6.200322491	Up	9.13E-11	3.78E-10
TGOLN2	1.885326008	Up	1.48E-09	5.40E-09
THADA	1.095952136	Up	0.000101868	0.000166284
THAP5	3.833346774	Up	1.05E-07	3.01E-07
THAP6	2.834699935	Up	2.91E-06	6.58E-06
THG1L	1.645595215	Up	6.24E-07	1.58E-06
thoc6	1.912702447	Up	5.47E-05	9.52E-05
THTPA	2.098308091	Up	6.59E-08	1.94E-07
THUMPD3	5.511080191	Up	3.19E-07	8.49E-07
THYN1	4.419662436	Up	0.000691126	0.00091553
TICRR	1.89126927	Up	7.27E-13	3.63E-12
TINF2	4.750220836	Up	0.000112532	0.000182234
TIPARP	4.588518378	Up	0.00028639	0.000419284
TJP1	1.282158912	Up	0.000150051	0.000234805
TJP2	4.889548412	Up	4.66E-05	8.22E-05
TKT	7.012617727	Up	3.24E-17	2.18E-16
TKTL1	4.834699935	Up	6.65E-05	0.000113504
TLE3	1.900297656	Up	3.55E-08	1.09E-07
TLR1	2.69268093	Up	1.46E-05	2.88E-05
TLR3	1.036333796	Up	0.000605959	0.000816839
TLR7	1.255406185	Up	0.000585833	0.000791977
TM2D2	4.419662436	Up	0.000691126	0.00091553
TM9SF1	2.864321985	Up	2.62E-07	7.06E-07
TMC2	1.801533071	Up	0.000429665	0.000602858
TMC5	4.642054857	Up	0.000212366	0.000320567
TMCC1	1.612056783	Up	1.89E-09	6.80E-09
TMED10	1.376343232	Up	2.75E-11	1.20E-10

TMED4	2.043673993	Up	1.09E-05	2.21E-05
TMEM116	1.729542491	Up	0.000229229	0.0003436
TMEM130	5.521760624	Up	2.88E-07	7.71E-07
TMEM136	4.934836606	Up	3.44E-05	6.27E-05
TMEM139	9.207086194	Up	4.74E-59	1.12E-57
TMEM14B	4.605528981	Up	0.000260709	0.000386299
TMEM161B	1.176495644	Up	0.000414427	0.000583754
TMEM165	1.806685559	Up	6.89E-06	1.45E-05
TMEM171	4.294131554	Up	5.07E-06	1.10E-05
TMEM18	2.057092357	Up	0.000280305	0.000411061
TMEM183A	6.050378532	Up	7.19E-10	2.71E-09
TMEM189-UBE2V1	8.267909789	Up	4.02E-35	5.57E-34
TMEM198	4.024129181	Up	7.95E-09	2.66E-08
TMEM205	6.564252706	Up	2.72E-13	1.40E-12
TMEM206	3.571665529	Up	6.68E-09	2.26E-08
TMEM210	3.044153301	Up	1.91E-07	5.25E-07
TMEM214	1.475157548	Up	7.36E-05	0.000124085
TMEM216	2.14212846	Up	2.39E-12	1.15E-11
TMEM217	5.613959656	Up	1.15E-07	3.28E-07
TMEM219	6.693924097	Up	2.51E-14	1.40E-13
TMEM231	8.501243515	Up	5.22E-40	8.32E-39
TMEM234	1.832340662	Up	1.17E-55	2.59E-54
tmem245	1.570738475	Up	1.67E-27	1.81E-26

TMEM267	4.922162776	Up	5.53E-09	1.89E-08
TMEM30A	6.120102154	Up	2.82E-10	1.11E-09
TMEM38B	1.739293744	Up	0.000313805	0.000454613
TMEM39B	1.898830273	Up	9.98E-06	2.04E-05
TMEM44	5.879094055	Up	6.16E-09	2.09E-08
TMEM51	1.137262705	Up	0.000128525	0.000205002
TMEM74B	4.462306773	Up	0.00055805	0.000757797
TMEM79	3.642054857	Up	0.000548719	0.000746049
TMEM80	4.535139653	Up	0.000382164	0.000541831
Tmem91	3.035044332	Up	0.000124942	0.000199875
TMEM92	2.589587437	Up	4.22E-05	7.52E-05
TMPO	6.248835468	Up	4.50E-11	1.92E-10
tmprss6	2.131068557	Up	1.14E-11	5.19E-11
TMSB10	2.967205253	Up	7.79E-08	2.27E-07
TMSB4X	3.155906502	Up	9.91E-05	0.000162157
TNFAIP3	2.004624937	Up	0.000451229	0.000629432
TNFRSF18	5.352605489	Up	1.36E-06	3.25E-06
TNFSF13	4.606585514	Up	0.000259184	0.000384247
TNFSF14	2.42659515	Up	0.000521511	0.000713878
TNFSF15	4.799098567	Up	8.33E-05	0.000138806
TNIK	5.69268093	Up	5.06E-08	1.52E-07
TNIP1	4.741590531	Up	0.000118566	0.000190485
TNS1	6.227017358	Up	6.20E-11	2.61E-10
TOB1	2.254078185	Up	6.66E-35	9.16E-34
TOPORS	1.877020782	Up	9.20E-55	2.01E-53
TOR1AIP2	2.778685779	Up	9.62E-13	4.77E-12
TOX4	1.255847855	Up	8.39E-31	1.02E-29
TP53	1.479529768	Up	2.64E-05	4.94E-05
TP53BP1	8.724051788	Up	2.83E-45	5.13E-44
TP53I11	6.356692892	Up	8.69E-12	3.98E-11
TP53TG3D	6.067744336	Up	5.71E-10	2.18E-09
TPBG	3.358261891	Up	1.79E-05	3.46E-05
TPD52L3	2.506114633	Up	5.03E-09	1.72E-08
TPI1	1.155861661	Up	4.77E-121	2.40E-119
TPK1	3.004624937	Up	0.000305949	0.000444199
TPM3	6.114171231	Up	3.05E-10	1.20E-09

TPP2	2.795396974	Up	4.63E-06	1.01E-05
TPPP3	1.732062132	Up	1.89E-24	1.81E-23
TPRA1	1.047749233	Up	3.00E-08	9.28E-08
TPST1	2.238957596	Up	6.94E-09	2.34E-08
TPTE	1.332563596	Up	1.11E-19	8.54E-19
TPTE2	1.620599175	Up	0.000174992	0.000269576
TRA2A	2.065279865	Up	3.58E-19	2.68E-18
TRAF3	3.000939034	Up	2.45E-08	7.68E-08
TRAF5	2.981308612	Up	2.48E-11	1.09E-10
TRAF7	1.233249312	Up	0.000299654	0.000437317
trak1	3.439027761	Up	1.83E-05	3.53E-05
TRANK1	1.834699935	Up	2.43E-05	4.57E-05
TRAPPC2	4.677678767	Up	0.000173087	0.000267288
TRIM3	1.507775635	Up	7.35E-05	0.000124082
TRIM35	1.916229821	Up	9.49E-19	6.95E-18
TRIM36	6.701113416	Up	2.19E-14	1.22E-13
TRIM45	6.448820804	Up	1.96E-12	9.50E-12
TRIM51	3.834699935	Up	0.000168506	0.000260719
TRIM54	4.044153301	Up	5.94E-09	2.02E-08
trim59	1.514459186	Up	2.42E-08	7.58E-08
TRIM61	7.044153301	Up	1.57E-17	1.08E-16
TRIM7	4.894747319	Up	4.50E-05	7.97E-05
TRIM71	1.21020907	Up	4.58E-06	9.99E-06
TRIO	2.15662803	Up	0.000404271	0.000570604
TRIT1	6.082627449	Up	4.68E-10	1.80E-09
TRMT10B	3.348061381	Up	2.32E-20	1.86E-19
TRMT11	3.741590531	Up	0.000303865	0.000442212
TRMT112	2.981028069	Up	5.85E-20	4.58E-19
TROVE2	5.61815659	Up	1.10E-07	3.15E-07
TRPC7	2.320126762	Up	9.05E-05	0.000149424
TRPM6	6.405162866	Up	4.01E-12	1.89E-11
TRPM7	4.800646768	Up	2.71E-06	6.17E-06
TRPV4	4.722225206	Up	0.000133176	0.00021169
TRRAP	1.057092357	Up	3.64E-07	9.60E-07
TSACC	4.839874544	Up	1.02E-21	8.70E-21
TSC1	1.030012441	Up	2.84E-07	7.63E-07
TSC22D1	1.251005231	Up	7.76E-08	2.26E-07
TSHR	1.419662436	Up	2.67E-05	4.98E-05
TSN	5.15662803	Up	6.86E-06	1.44E-05
TSNAX	4.511644294	Up	0.000432617	0.000606476
TSNAXIP1	1.633360508	Up	8.69E-07	2.15E-06
TSPAN16	1.12137602	Up	5.33E-06	1.15E-05

TSPAN6	4.790756588	Up	8.77E-05	0.000145451
TSPAN9	4.425904501	Up	8.29E-33	1.07E-31
TSPOAP1	1.904402102	Up	2.23E-09	7.96E-09
TSPYL5	1.148066017	Up	1.23E-38	1.88E-37
TSSC4	5.004624937	Up	2.12E-05	4.03E-05
TSSK4	1.609298764	Up	8.65E-07	2.14E-06
TSTD2	1.459536693	Up	1.29E-15	7.89E-15
TTC16	1.087806781	Up	1.77E-15	1.07E-14
TTC17	4.741590531	Up	0.000118566	0.000190485
TTC28	1.141654267	Up	2.89E-18	2.06E-17
TTC29	5.24680398	Up	3.34E-06	7.46E-06
TTC4	1.443509178	Up	2.85E-05	5.30E-05
TTC7A	2.744349216	Up	1.50E-28	1.70E-27
TTC7B	2.741590531	Up	8.54E-06	1.77E-05
TTF2	1.341659924	Up	0.000195516	0.000298563
TTI1	2.267659342	Up	0.000149652	0.000234247
TTL	1.462731158	Up	8.11E-06	1.68E-05
TTLL1	1.571665529	Up	0.00030787	0.000446589
TTLL2	2.258726218	Up	2.54E-20	2.03E-19
ttll5	1.172237867	Up	1.38E-56	3.13E-55
TTLL6	1.592482535	Up	2.24E-29	2.61E-28
TTN	1.814213479	Up	1.05E-12	5.18E-12
TTPAL	5.823838943	Up	1.18E-08	3.86E-08
TTYH2	3.820200365	Up	0.000185158	0.000283921
TUBB2A	4.480862592	Up	0.000507556	0.000699334
TUBB8	4.004624937	Up	5.21E-05	9.11E-05
TUSC3	1.419662436	Up	3.35E-07	8.86E-07
TWSG1	4.419662436	Up	0.000691126	0.00091553
TXNL4B	4.627555288	Up	0.000230507	0.00034542
TXNRD1	1.967276778	Up	0.000624871	0.000839212
TXNRD2	2.164565849	Up	2.74E-06	6.23E-06
TYMS	1.555560736	Up	1.58E-06	3.74E-06
TYW1B	2.728668032	Up	1.18E-11	5.33E-11
UBA5	6.185550741	Up	1.13E-10	4.63E-10
UBA52	2.803663896	Up	3.58E-61	8.78E-60
UBALD1	1.145487472	Up	3.67E-07	9.67E-07
UBAP1	4.915357599	Up	3.92E-05	7.04E-05
UBAP2	1.464780487	Up	7.80E-69	2.17E-67
UBAP2L	7.482014445	Up	1.59E-22	1.40E-21
UBB	5.655899913	Up	7.47E-08	2.19E-07
UBE2D2	1.283629764	Up	0.000535419	0.000731182
UBE2F	1.185097689	Up	1.78E-05	3.45E-05
UBE2H	5.004624937	Up	2.12E-05	4.03E-05
UBE2I	2.929784428	Up	4.47E-07	1.16E-06

UBE2J1	2.571665529	Up	6.86E-07	1.73E-06
UBE2K	5.403947965	Up	8.64E-07	2.14E-06
UBE2L3	3.806071389	Up	1.12E-11	5.08E-11
UBE2V1	7.324949146	Up	1.36E-20	1.10E-19
UBE3B	1.000122675	Up	5.93E-07	1.51E-06
UBE3C	1.15662803	Up	0.000474006	0.000657755
UBE4A	2.934235609	Up	7.23E-11	3.02E-10
UBL7	2.15662803	Up	7.66E-06	1.60E-05
UBR1	1.915619931	Up	1.18E-06	2.85E-06
UBR3	2.419662436	Up	8.99E-20	6.97E-19
UBTFL1	1.593691836	Up	0.000131764	0.000209551
UBXN10	1.191234596	Up	2.42E-22	2.11E-21
UBXN11	4.55169083	Up	4.28E-07	1.11E-06
UBXN7	2.249140169	Up	2.72E-94	1.04E-92
UCK2	1.381443114	Up	2.50E-10	9.91E-10
UCP3	1.844709212	Up	0.000314444	0.000455508
UGGT2	1.202270696	Up	2.57E-15	1.53E-14
UGP2	2.51277184	Up	1.17E-05	2.34E-05
UHMK1	6.667589949	Up	4.13E-14	2.27E-13
ULBP2	1.759939841	Up	0.000138336	0.000218116
ULK1	1.419662436	Up	1.24E-06	2.98E-06
UMODL1	4.082627449	Up	2.90E-05	5.37E-05
UNKL	4.669613365	Up	0.000181361	0.000278517
Uqcrb	1.288891703	Up	3.69E-12	1.74E-11
Uqcrc2	2.025841423	Up	1.31E-05	2.61E-05
Uqcrh	5.082627449	Up	1.20E-05	2.40E-05
USF1	2.236798379	Up	1.07E-05	2.18E-05
USP14	1.767585739	Up	5.82E-05	0.000100797
USP15	3.355597043	Up	3.74E-37	5.48E-36
USP16	4.734842196	Up	0.000123485	0.00019774
USP17L1	1.724385178	Up	9.20E-16	5.66E-15
USP17L15	6.597580228	Up	1.50E-13	7.90E-13
USP17L17	3.485542941	Up	9.38E-51	1.90E-49
USP19	5.144391358	Up	5.10E-08	1.53E-07
USP30	2.11713974	Up	1.29E-05	2.56E-05
USP39	4.477401507	Up	0.000516657	0.000707972
USP4	7.024524494	Up	2.46E-17	1.67E-16
USP44	2.236271367	Up	4.67E-24	4.40E-23
USP46	5.406376745	Up	8.45E-07	2.09E-06
USP47	6.26096469	Up	3.76E-11	1.62E-10
USP6NL	2.312546174	Up	2.63E-16	1.68E-15
USP7	1.363594349	Up	1.10E-24	1.06E-23

UTP14C	4.922162776	Up	5.53E-09	1.89E-08
UTRN	1.604580152	Up	3.50E-21	2.90E-20
VAMP3	2.549639223	Up	6.78E-05	0.000115486
VAV2	7.19225194	Up	4.36E-19	3.25E-18
VCY1B	2.000850447	Up	7.80E-21	6.36E-20
VDAC2	4.358261891	Up	2.84E-06	6.45E-06
VEPH1	1.35014639	Up	6.60E-05	0.00011303
vkorc1	4.741590531	Up	0.000118566	0.000190485
VPS13A	4.339176907	Up	4.92E-81	1.62E-79
VPS13B	3.035040925	Up	5.00E-48	9.54E-47
VPS13C	3.097734341	Up	2.72E-17	1.84E-16
VPS25	4.741590531	Up	0.000118566	0.000190485
VPS26A	5.642054857	Up	8.63E-08	2.50E-07
VPS28	5.233871029	Up	3.71E-06	8.23E-06
VPS39	1.508471703	Up	5.85E-06	1.25E-05
VPS52	1.862325841	Up	4.97E-07	1.28E-06
VPS53	1.002028159	Up	5.57E-07	1.42E-06
VRK2	5.642054857	Up	8.63E-08	2.50E-07
VTCN1	1.794057951	Up	0.000209318	0.000317528
VWA5A	2.733875566	Up	9.31E-06	1.91E-05
VWCE	1.950177153	Up	0.000722703	0.000953274
WAC	1.954733131	Up	3.36E-09	1.17E-08
WAPL	1.486776632	Up	0.000279762	0.000410566
WARS	4.698946193	Up	4.83E-21	3.98E-20
wasf1	6.988099963	Up	5.64E-17	3.74E-16
WASF2	3.194347696	Up	1.09E-06	2.66E-06
WDR11	2.213211558	Up	0.000246513	0.000367036
WDR19	2.497664948	Up	0.00076148	0.000997826
WDR20	1.96440289	Up	2.39E-07	6.50E-07
WDR26	1.318206233	Up	1.06E-21	9.02E-21
WDR31	5.010224536	Up	2.04E-05	3.89E-05
WDR33	1.609549358	Up	1.50E-06	3.57E-06
WDR38	3.057976632	Up	6.26E-05	0.000107724
WDR62	1.145732716	Up	2.47E-08	7.74E-08
WDR66	1.914427128	Up	7.90E-11	3.29E-10
WDR7	1.073887599	Up	5.51E-13	2.78E-12
WDR70	2.486776632	Up	3.22E-07	8.55E-07
WDR74	1.338013886	Up	2.49E-09	8.83E-09
WDR93	5.963982952	Up	2.18E-09	7.80E-09
WDYHV1	1.478556125	Up	0.000114673	0.000185353
WFDC5	1.164848537	Up	1.75E-09	6.31E-09
WHRN	4.028918452	Up	1.03E-19	7.95E-19
whsc1	1.615344085	Up	6.02E-06	1.28E-05
WIPI1	2.514461509	Up	1.06E-11	4.83E-11

WRAP53	5.553883376	Up	2.10E-07	5.77E-07
WRN	2.811979859	Up	6.33E-11	2.67E-10
WTIP	3.49698683	Up	0.000616164	0.000829321
WWP1	4.294131554	Up	5.07E-06	1.10E-05
WWP2	1.765650919	Up	4.41E-06	9.64E-06
XIAP	1.370624392	Up	2.34E-05	4.42E-05
XKR4	3.642054857	Up	0.000548719	0.000746049
XPNPEP3	1.446885998	Up	2.87E-51	5.86E-50
XPO5	1.302848771	Up	6.35E-06	1.35E-05
XPR1	6.231818344	Up	5.78E-11	2.45E-10
XRCC2	1.68921172	Up	4.60E-06	1.00E-05
XRCC6	7.810605209	Up	3.77E-27	4.04E-26
XRN1	5.750220836	Up	2.71E-08	8.43E-08
XRN2	1.391875256	Up	6.57E-05	0.000112669
XRRA1	1.308571797	Up	1.02E-17	7.07E-17
YIF1B	1.135464115	Up	4.57E-08	1.38E-07
YIPF1	1.834699935	Up	0.000318327	0.000460085
YIPF6	4.419662436	Up	0.000691126	0.00091553
YJEFN3	1.210257031	Up	2.40E-07	6.52E-07
YKT6	2.320126762	Up	3.09E-08	9.54E-08
YME1L1	3.107096444	Up	7.69E-08	2.24E-07
YPEL2	2.303017517	Up	8.42E-05	0.000140218
YPEL5	1.61105428	Up	7.89E-38	1.18E-36
YRDC	1.308631124	Up	6.62E-07	1.67E-06
YWHAQ	1.309784818	Up	4.46E-09	1.54E-08
YY1AP1	6.140670456	Up	2.12E-10	8.47E-10
ZAN	1.051930651	Up	5.58E-05	9.70E-05
ZBP1	4.835601338	Up	6.61E-05	0.000113239
ZBTB1	4.375926545	Up	2.10E-57	4.82E-56
ZBTB2	1.642054857	Up	0.000135374	0.000214849
ZBTB21	1.239502212	Up	1.32E-06	3.15E-06
zbtb3	1.716582041	Up	0.000465115	0.000647341
ZBTB43	3.713915572	Up	4.51E-07	1.17E-06
ZBTB44	1.488428212	Up	3.14E-25	3.11E-24
ZBTB7B	5.358261891	Up	1.30E-06	3.11E-06
ZBTB8A	1.132672715	Up	2.92E-06	6.60E-06
ZBTB9	1.25130473	Up	3.97E-17	2.66E-16
ZC2HC1A	4.982820566	Up	2.47E-05	4.64E-05
ZC2HC1C	1.419662436	Up	0.000246377	0.000367036
ZC3H10	1.907129546	Up	3.45E-39	5.38E-38
ZC3H11A	2.358849564	Up	9.70E-07	2.37E-06
ZC3H14	2.285890385	Up	0.000614472	0.000827323
ZC3H7A	1.794362349	Up	2.54E-08	7.93E-08

ZC3HAV1	2.993010308	Up	0.000408117	0.000575587
ZCCHC11	1.923011171	Up	1.41E-30	1.71E-29
ZDHHC14	3.589587437	Up	1.81E-06	4.24E-06
ZDHHC16	2.354009572	Up	1.18E-10	4.82E-10
ZDHHC20	4.641023993	Up	0.000213612	0.000322283
ZDHHC3	3.320620921	Up	4.44E-34	5.97E-33
ZDHHC6	1.212004787	Up	4.50E-13	2.28E-12
ZEB1	4.875592366	Up	5.10E-05	8.96E-05
ZFAND4	5.868563387	Up	6.98E-09	2.35E-08
ZFP30	2.047693659	Up	4.73E-12	2.22E-11
ZFP42	5.680189986	Up	5.78E-08	1.72E-07
zfp64	4.642054857	Up	0.000212366	0.000320567
ZFX	4.419662436	Up	0.000691126	0.00091553
zfyve1	5.63275045	Up	9.50E-08	2.74E-07
ZG16B	1.242784674	Up	7.26E-05	0.000122664
ZGPAT	4.834699935	Up	6.65E-05	0.000113504
ZGRF1	1.597660738	Up	9.93E-10	3.69E-09
ZIK1	2.950177153	Up	1.70E-05	3.31E-05
ZKSCAN3	3.141261424	Up	2.32E-10	9.21E-10
ZKSCAN7	5.222200342	Up	4.08E-06	8.97E-06
ZKSCAN8	6.410314813	Up	3.69E-12	1.74E-11
ZMYM2	1.468483235	Up	1.05E-19	8.14E-19
ZMYM6	5.125271786	Up	8.73E-06	1.80E-05
ZMYND11	5.013414576	Up	1.99E-05	3.82E-05
ZMYND8	7.258278106	Up	8.01E-20	6.21E-19
ZNF10	1.732820321	Up	3.03E-08	9.37E-08
ZNF106	4.551969728	Up	3.95E-08	1.21E-07
ZNF124	1.23735665	Up	2.50E-06	5.70E-06
ZNF133	7.686698772	Up	2.62E-25	2.61E-24
ZNF141	4.589587437	Up	2.87E-07	7.68E-07
ZNF146	1.315526695	Up	0.000108353	0.000175999
ZNF148	1.245332255	Up	5.89E-07	1.50E-06
ZNF154	1.392064877	Up	9.68E-05	0.0001587
ZNF160	1.532810567	Up	7.22E-16	4.47E-15
ZNF177	3.642054857	Up	0.000548719	0.000746049
ZNF184	1.419662436	Up	6.47E-05	0.000111039
ZNF197	5.651299642	Up	7.84E-08	2.29E-07

ZNF20	1.972546081	Up	4.17E-08	1.27E-07
ZNF200	2.329566951	Up	2.90E-09	1.02E-08
ZNF207	7.245617623	Up	1.11E-19	8.58E-19
znf208	1.727784731	Up	1.07E-05	2.17E-05
ZNF213	1.572275454	Up	3.04E-43	5.24E-42
ZNF219	3.870174737	Up	4.39E-09	1.51E-08
ZNF222	1.361946938	Up	0.000331794	0.000477631
ZNF226	2.39235509	Up	0.000256191	0.00038017
ZNF23	4.417255938	Up	0.0006994	0.000925408
ZNF232	1.208158331	Up	0.000420819	0.000591581
ZNF236	8.419662436	Up	3.13E-38	4.75E-37
ZNF248	2.007418152	Up	0.000167435	0.000259537
ZNF250	5.811063571	Up	1.37E-08	4.44E-08
ZNF251	2.294131554	Up	2.43E-05	4.57E-05
ZNF257	5.589587437	Up	1.47E-07	4.13E-07
ZNF274	6.158070004	Up	1.66E-10	6.71E-10
ZNF280B	1.418176398	Up	2.23E-28	2.51E-27
ZNF286A	4.208807894	Up	4.29E-11	1.84E-10
ZNF286B	1.168358785	Up	5.04E-06	1.09E-05
ZNF3	4.814725235	Up	7.55E-05	0.000126989
ZNF302	7.510656968	Up	6.75E-23	6.06E-22
ZNF304	5.15662803	Up	6.86E-06	1.44E-05
ZNF331	5.658958632	Up	7.23E-08	2.12E-07
ZNF33B	1.871168102	Up	6.51E-11	2.74E-10
ZNF346	2.871556008	Up	2.39E-05	4.51E-05
ZNF362	3.461139072	Up	6.76E-06	1.43E-05
ZNF382	4.419662436	Up	0.000691126	0.00091553
ZNF383	2.379020451	Up	8.90E-06	1.83E-05
ZNF384	2.455911769	Up	2.01E-13	1.05E-12
ZNF385B	2.196187587	Up	2.91E-07	7.79E-07
ZNF385D	1.517509759	Up	1.40E-05	2.77E-05
ZNF407	1.558634725	Up	1.15E-12	5.66E-12
ZNF415	3.026262586	Up	7.88E-09	2.64E-08
ZNF416	1.06220377	Up	2.88E-16	1.83E-15
ZNF426	1.341032037	Up	5.82E-16	3.63E-15
ZNF433	5.950731929	Up	2.58E-09	9.12E-09
ZNF439	1.603766373	Up	0.000213207	0.000321748
ZNF44	1.981541323	Up	1.32E-07	3.73E-07
ZNF440	2.05371846	Up	6.49E-10	2.46E-09
ZNF451	6.002619801	Up	1.34E-09	4.90E-09
ZNF461	4.13042296	Up	2.00E-05	3.83E-05
ZNF473	1.027913025	Up	3.64E-14	2.01E-13
ZNF479	2.572386697	Up	6.79E-07	1.71E-06
ZNF490	1.238993388	Up	1.12E-38	1.72E-37

ZNF506	1.315724001	Up	1.01E-14	5.79E-14
ZNF510	1.750419404	Up	0.000254937	0.00037864
ZNF521	6.69268093	Up	2.57E-14	1.43E-13
ZNF528	3.72889407	Up	1.82E-11	8.10E-11
ZNF534	4.419662436	Up	0.000691126	0.00091553
ZNF552	3.740643061	Up	1.58E-33	2.09E-32
ZNF555	1.363327017	Up	5.15E-43	8.84E-42
ZNF57	1.672864252	Up	8.67E-09	2.89E-08
ZNF574	2.26096469	Up	1.43E-11	6.42E-11
ZNF585A	2.083561685	Up	5.29E-08	1.58E-07
ZNF587B	1.774759473	Up	2.70E-15	1.61E-14
ZNF600	2.294131554	Up	2.64E-13	1.37E-12
ZNF607	3.518496988	Up	9.29E-67	2.51E-65
ZNF608	1.25859961	Up	5.72E-28	6.33E-27
ZNF615	5.69268093	Up	5.06E-08	1.52E-07
ZNF638	1.750615988	Up	1.99E-08	6.32E-08
ZNF653	1.256932936	Up	8.00E-05	0.000133923
ZNF655	6.542059067	Up	4.01E-13	2.05E-12
ZNF678	4.419662436	Up	0.000691126	0.00091553
ZNF705B	4.645143039	Up	0.000208671	0.000316656
ZNF705E	4.745192767	Up	2.35E-09	8.37E-09
ZNF732	2.709169053	Up	8.47E-08	2.46E-07
ZNF772	1.834193282	Up	1.58E-06	3.73E-06
ZNF778	2.71131752	Up	8.60E-46	1.58E-44
ZNF780A	3.740631611	Up	3.75E-10	1.46E-09
ZNF783	1.242637477	Up	5.31E-05	9.27E-05
ZNF789	2.4591908	Up	3.22E-06	7.21E-06
ZNF791	2.237578003	Up	1.06E-05	2.16E-05
ZNF8	1.989978161	Up	2.56E-06	5.84E-06
ZNF800	3.127481684	Up	1.28E-09	4.71E-09
ZNF808	1.798174059	Up	8.86E-05	0.000146689
ZNF816	4.2921629	Up	5.16E-06	1.11E-05
ZNF83	5.950731929	Up	2.58E-09	9.12E-09
ZNF84	1.156865023	Up	2.59E-08	8.08E-08
ZNF845	5.294131554	Up	2.25E-06	5.18E-06
ZNF850	2.381747664	Up	8.03E-06	1.67E-05
ZNF853	5.572386697	Up	1.75E-07	4.85E-07
znf91	4.542887171	Up	0.000366708	0.000523648
ZNF93	5.082627449	Up	1.20E-05	2.40E-05
ZNRD1	3.24887017	Up	3.65E-07	9.61E-07
ZPR1	2.778148021	Up	4.57E-17	3.05E-16
ZSCAN32	4.120102154	Up	1.90E-09	6.84E-09
zswim5	1.869267828	Up	3.70E-05	6.71E-05
ZSWIM8	6.141764097	Up	2.09E-10	8.35E-10

ZWILCH	4.922162776	Up		3.75E-05	6.77E-05
ZXDC	3.552556706	Up		0.000394557	0.000558077

2.4 Supplementary Table 4

Table S4. The 1198 downregulated DEGs in GSE92578.

GeneID	length	log2Ratio(H-AsthS/H-sperm)	Up-Down-Regulation (H-AsthS/H-sperm)	p-value	q-value
AATK	5089	-1.612759042	Down	1.86E-06	4.33E-06
ABL2	11568	-5.162592472	Down	2.07E-05	3.96E-05
ABR	4775	-4.980875494	Down	6.90E-05	0.000117345
ABTB2	4903	-1.450702284	Down	7.26E-10	2.74E-09
ACAP2	7170	-1.522852069	Down	0.000156508	0.000243934
ACBD7	3370	-1.666629387	Down	0.000304105	0.00044243
ACD	2066	-6.918316194	Down	1.05E-14	5.99E-14
ACIN1	4817	-3.376979812	Down	3.17E-09	1.11E-08
ACKR4	2505	-6.047453982	Down	6.14E-09	2.08E-08
ACOT11	2015	-2.663951242	Down	1.38E-06	3.30E-06
ACSS1	3676	-1.64246211	Down	6.87E-05	0.000117001
ACY1	1673	-7.007021646	Down	1.81E-15	1.09E-14
ADAD1	2002	-2.358694789	Down	1.54E-07	4.29E-07
ADAM11	4423	-1.583210942	Down	4.08E-24	3.85E-23
ADAM12	8028	-3.310236346	Down	3.27E-05	5.99E-05
ADAM18	2399	-5.624731683	Down	5.04E-07	1.30E-06
ADAMTS10	4271	-2.217731503	Down	5.08E-11	2.16E-10
ADAMTS13	4690	-1.0587999	Down	9.26E-06	1.90E-05
ADAP1	1947	-3.48722816	Down	1.69E-05	3.29E-05
ADAR	6536	-5.950850617	Down	1.85E-08	5.91E-08
ADD2	3697	-1.14464723	Down	1.26E-08	4.12E-08
ADGRE1	2722	-5.145325365	Down	2.34E-05	4.41E-05
ADGRG5	3340	-3.975712625	Down	0.000148725	0.000233005
ADORA2A	2635	-1.670797547	Down	0.000245512	0.000365906
AEN	3116	-1.207120241	Down	3.54E-05	6.44E-05
AGAP2	4860	-1.405193311	Down	8.00E-07	1.99E-06
AGBL5	3237	-1.388452177	Down	5.83E-13	2.93E-12
AGRN	7326	-1.649094576	Down	1.19E-10	4.89E-10
AGTRAP	1127	-4.48722816	Down	3.24E-06	7.26E-06
AHCY	2371	-4.957114136	Down	7.99E-05	0.000133847
AHSP	464	-4.07219066	Down	8.45E-05	0.000140663
AK8	2113	-6.294583082	Down	2.70E-10	1.06E-09
AKT1S1	1813	-1.6480162	Down	9.09E-07	2.24E-06
AKT2	5170	-3.476021031	Down	8.76E-05	0.000145336

ALDH3B1	2485	-4.07219066	Down	8.45E-05	0.000140663
ALDH5A1	5131	-3.50645014	Down	1.87E-15	1.13E-14
ALPL	2322	-1.672260054	Down	3.44E-06	7.67E-06
ALX4	5466	-1.07676339	Down	6.10E-05	0.00010527
AMELY	802	-2.165300065	Down	7.06E-05	0.000119932
AMER2	9939	-5.497291843	Down	1.56E-06	3.69E-06
ANAPC4	2672	-4.750262566	Down	0.000265439	0.00039105
ANGPTL6	1857	-5.252762906	Down	1.08E-05	2.19E-05
ANK1	8157	-2.318914682	Down	9.65E-12	4.41E-11
ANKRD28	6383	-6.219280233	Down	7.33E-10	2.76E-09
ANKRD34C	5424	-3.865739783	Down	0.00031738	0.00045908
ANKRD35	3093	-2.031107671	Down	1.30E-47	2.47E-46
ANP32A	2466	-1.786788442	Down	2.85E-05	5.28E-05
AOX1	4933	-4.972654987	Down	7.26E-05	0.000122618
apcdd1l	3426	-2.750262566	Down	0.000255065	0.00037864
APLP1	2450	-1.229675674	Down	1.46E-22	1.29E-21
APOOL	6467	-1.548641035	Down	1.65E-09	5.98E-09
APP	3240	-4.996163822	Down	6.26E-05	0.000107742
APPBP2	6499	-5.557617488	Down	9.23E-07	2.26E-06
AQP3	1871	-2.428334471	Down	8.06E-05	0.000134849
AQP7	1242	-1.400689169	Down	6.43E-06	1.36E-05
ARAP1	5137	-1.934992488	Down	9.39E-45	1.69E-43
ARF1	1985	-2.920160996	Down	4.02E-25	3.96E-24
ARHGAP27	3650	-4.865739783	Down	0.000138382	0.000218116
ARHGAP30	3792	-5.688862021	Down	2.76E-07	7.43E-07
ARHGAP32	9443	-7.893356616	Down	1.51E-25	1.51E-24
ARHGAP39	4669	-1.466955765	Down	3.37E-09	1.18E-08
ARHGEF11	6889	-2.052044087	Down	1.14E-12	5.63E-12
ARHGEF2	4179	-9.467207746	Down	7.19E-61	1.76E-59
ARHGEF4	3656	-1.748630865	Down	6.10E-09	2.07E-08
ARID1A	8585	-1.029780915	Down	1.95E-09	7.02E-09

ARID3A	2810	-1.112832645	Down	1.48E-06	3.51E-06
ARID3B	4274	-3.954693975	Down	8.58E-19	6.29E-18
ARIH2	4090	-1.759945119	Down	1.07E-09	3.98E-09
ARL6IP6	2826	-3.252762906	Down	0.000116723	0.000188298
ARL8B	2985	-2.02328106	Down	0.000107318	0.000174466
ARMC10	2457	-5.688862021	Down	2.76E-07	7.43E-07
ARPC4	1971	-5.685722313	Down	2.85E-07	7.64E-07
ARPP19	5525	-2.322039247	Down	7.10E-11	2.97E-10
ARRDC4	4067	-3.48722816	Down	1.69E-05	3.29E-05
asic1	3662	-5.865739783	Down	4.67E-08	1.41E-07
ASTN1	4221	-5.165300065	Down	2.03E-05	3.89E-05
ATAD3A	2502	-2.495874138	Down	7.03E-07	1.76E-06
ATF5	2041	-1.781599944	Down	2.01E-21	1.69E-20
atf7ip2	3665	-4.865739783	Down	0.000138382	0.000218116
ATG101	1437	-2.272215269	Down	7.06E-09	2.38E-08
ATP2C2	3476	-6.745143951	Down	2.54E-13	1.32E-12
ATP6V0C	1021	-2.865739783	Down	7.15E-06	1.50E-05
ATP8B1	5938	-4.865739783	Down	0.000138382	0.000218116
ATP9B	4479	-5.865739783	Down	4.67E-08	1.41E-07
ATPAF1	1829	-1.170697359	Down	0.000414371	0.000583694
ATXN7L1	5423	-5.656511821	Down	3.75E-07	9.86E-07
B3GAT3	2078	-1.37408379	Down	0.000636458	0.000853468
B3GNT4	1504	-1.56969032	Down	5.13E-05	9.00E-05
BAGE5	1755	-1.163046191	Down	8.72E-17	5.72E-16
BAHD1	4711	-5.07219066	Down	3.83E-05	6.89E-05
BAIAP3	4681	-6.956072103	Down	5.01E-15	2.94E-14
bcam	3431	-4.165300065	Down	4.38E-05	7.78E-05
BCAR1	2926	-5.809156255	Down	8.41E-08	2.44E-07
BCL3	1864	-1.32220612	Down	5.76E-34	7.71E-33
BCL6	3083	-5.380201975	Down	4.09E-06	8.99E-06
BCL6B	3525	-1.802729985	Down	0.000426237	0.000598392
BCL7B	1711	-1.524381158	Down	5.67E-06	1.21E-05
BCL9L	7739	-1.078885313	Down	8.94E-09	2.97E-08
BCOR	6423	-6.37475343	Down	8.86E-11	3.67E-10
BDKRB2	4197	-2.750262566	Down	0.000255065	0.00037864
BHMG1	2754	-1.60139918	Down	3.27E-05	5.99E-05
BICDL1	3081	-2.165300065	Down	1.87E-14	1.05E-13
BIN1	2408	-1.175143898	Down	0.000622264	0.00083607
BIN2	2255	-5.165300065	Down	2.03E-05	3.89E-05
BPIFA2	1129	-4.165300065	Down	4.38E-05	7.78E-05
BRD4	5198	-1.359451532	Down	7.82E-129	4.17E-127
BRSK1	3081	-1.70371998	Down	5.74E-07	1.46E-06

BZW1	3383	-7.800183702	Down	3.03E-24	2.88E-23
c10orf2	3621	-1.532196628	Down	5.51E-17	3.66E-16
C12orf80	1292	-1.798691962	Down	0.000165089	0.000256154
C19orf45	1679	-1.144260846	Down	6.10E-08	1.81E-07
C1orf141	2203	-3.972654987	Down	0.000163599	0.000253976
c1orf35	1431	-2.0810336	Down	2.21E-11	9.75E-11
C1ORF94	3004	-4.749013402	Down	2.98E-21	2.48E-20
C20orf14<br>1	617	-1.533687471	Down	8.23E-05	0.00013723
c21orf91	5430	-4.674995907	Down	0.000396213	0.000560239
C2orf81	2071	-5.918048656	Down	2.66E-08	8.29E-08
C2orf88	3962	-3.165300065	Down	6.10E-06	1.30E-05
c4a	5288	-1.189605777	Down	3.23E-39	5.05E-38
C4BPA	2243	-4.865739783	Down	0.000138382	0.000218116
C6orf120	4246	-2.167642967	Down	6.10E-10	2.32E-09
C6orf47	2475	-1.114074741	Down	2.40E-05	4.53E-05
C6orf99	972	-4.865739783	Down	0.000138382	0.000218116
C8G	877	-4.865739783	Down	0.000138382	0.000218116
C9orf116	676	-4.865739783	Down	0.000138382	0.000218116
c9orf172	2931	-1.098558395	Down	5.77E-82	1.93E-80
CABLES<br>1	5005	-7.075553028	Down	4.38E-16	2.75E-15
CACNA1<br>I	10003	-6.165300065	Down	1.46E-09	5.33E-09
CACNG2	4523	-1.226613614	Down	3.22E-06	7.21E-06
calb1	2535	-2.667800405	Down	0.000470282	0.000653455
CALCOC<br>O1	2790	-4.991085692	Down	6.47E-05	0.000111011
CALD1	4282	-5.177868738	Down	1.86E-05	3.59E-05
CALML3	1380	-1.228626	Down	1.56E-10	6.29E-10
CAPN15	4744	-1.44454957	Down	3.28E-17	2.20E-16
CAPN3	3298	-4.64427187	Down	0.00046417	0.00064619
CARD10	3899	-2.027381822	Down	8.67E-131	4.69E-129
CASKIN<br>2	4499	-2.504903356	Down	2.50E-12	1.20E-11
casp8	1123	-2.498971493	Down	0.000179472	0.000275924
CASZ1	7962	-2.067480864	Down	0.000248651	0.00036997
CAV3	1335	-3.165300065	Down	6.10E-06	1.30E-05
CBARP	2564	-1.10781457	Down	3.08E-07	8.21E-07
CBFA2T<br>3	4038	-1.619315176	Down	0.000216633	0.000326413
CC2D2A	5240	-1.049822847	Down	0.000177745	0.000273596
CCDC10<br>3	1748	-1.165300065	Down	0.000321903	0.000464825

CCDC106	1992	-1.605872656	Down	0.000154844	0.000241557
CCDC114	3220	-1.532082396	Down	4.34E-09	1.50E-08
CCDC129	4018	-1.660064757	Down	0.000466494	0.000648745
CCDC157	4878	-5.47345704	Down	1.91E-06	4.44E-06
CCDC43	2138	-1.086281755	Down	2.22E-09	7.91E-09
CCDC85C	5671	-1.064371156	Down	0.000206222	0.000313395
CCDC92	2151	-5.837159533	Down	6.30E-08	1.86E-07
CCKBR	1931	-2.755289383	Down	3.50E-05	6.39E-05
CCR7	2134	-1.549963915	Down	4.12E-05	7.37E-05
CCS	1032	-3.335225066	Down	6.14E-05	0.00010573
CCT4	2349	-4.972654987	Down	7.26E-05	0.000122618
CD163L1	4644	-1.286162923	Down	4.90E-29	5.62E-28
CD164L2	1563	-3.438318559	Down	2.59E-07	7.00E-07
CD276	2747	-5.743635014	Down	1.62E-07	4.52E-07
CD44	5732	-4.685093551	Down	0.000375882	0.00053572
CD55	2796	-5.12414274	Down	2.70E-05	5.04E-05
CD74	1681	-5.688862021	Down	2.76E-07	7.43E-07
cdc25b	3573	-6.294583082	Down	2.70E-10	1.06E-09
CDC27	5850	-2.201150749	Down	5.18E-12	2.42E-11
CDC42EP1	2162	-1.012143277	Down	1.21E-11	5.49E-11
CDC42SE1	3047	-5.043044315	Down	4.64E-05	8.20E-05
CDCA2	3794	-3.07219066	Down	0.00042043	0.000591129
CDH13	3904	-4.624731683	Down	0.000512547	0.000702989
CDK13	6975	-1.540764639	Down	0.000426556	0.000598784
CDK19	6016	-4.707558115	Down	0.000333923	0.000480522
CDK9	2472	-1.196427114	Down	8.57E-08	2.49E-07
CDRT1	3210	-4.961274759	Down	7.79E-05	0.000130728
CEACAM21	1439	-6.165300065	Down	1.46E-09	5.33E-09
CEACAM5	3504	-2.891404666	Down	8.15E-05	0.000136091
CECR2	9657	-5.282995108	Down	8.66E-06	1.79E-05
CECR6	3961	-2.467129187	Down	2.26E-07	6.16E-07
CELF3	5596	-1.732437535	Down	5.70E-05	9.89E-05
cemip	7112	-3.750262566	Down	0.000617248	0.000829661
CENPS	1243	-1.146138566	Down	2.33E-09	8.29E-09
CEP120	4630	-2.413227578	Down	0.00047376	0.000657454

CEP170B	6705	-8.485513941	Down	1.49E-35	2.09E-34
CEP57	3222	-4.865739783	Down	0.000138382	0.000218116
CEP97	7516	-1.804978563	Down	1.81E-05	3.50E-05
CERCAM	2213	-1.902265659	Down	0.000155262	0.000242096
CFAP65	3270	-1.285647347	Down	3.16E-09	1.11E-08
CHD1L	2907	-3.688862021	Down	2.45E-06	5.60E-06
CHD3	7325	-7.316671841	Down	1.90E-18	1.37E-17
CHKA	2717	-4.515089935	Down	2.52E-06	5.76E-06
CHST12	2133	-1.75111096	Down	1.13E-20	9.12E-20
CIC	5472	-1.094057497	Down	2.08E-06	4.83E-06
CIDEB	2242	-1.119129883	Down	2.91E-13	1.50E-12
CISD3	2636	-1.381563781	Down	3.04E-53	6.49E-52
CKAP2L	4789	-6.809156255	Down	8.10E-14	4.37E-13
CLCN6	5632	-2.157033225	Down	1.02E-92	3.87E-91
CLCN7	4154	-2.784229633	Down	7.63E-19	5.61E-18
CLDN20	1209	-3.280777282	Down	1.73E-06	4.06E-06
CLDN7	1924	-5.470541031	Down	1.95E-06	4.55E-06
CLDND1	2179	-4.972654987	Down	7.26E-05	0.000122618
CLEC18A	2215	-4.804532228	Down	0.000196557	0.000299965
CLN5	2800	-1.387692486	Down	0.000357854	0.000512021
CLPTM1	2631	-1.281273662	Down	2.03E-14	1.14E-13
CMTM5	954	-3.972654987	Down	0.000163599	0.000253976
CNN1	1633	-4.741822203	Down	0.00027789	0.000407996
CNN3	1974	-4.972654987	Down	7.26E-05	0.000122618
CNPY2	1221	-2.810782993	Down	7.12E-18	4.99E-17
CNTNAP1	5454	-1.502335052	Down	2.31E-12	1.11E-11
COL11A1	7291	-1.385244717	Down	3.38E-11	1.46E-10
COL11A2	6167	-1.416835529	Down	5.77E-87	2.05E-85
COL16A1	5584	-1.063792483	Down	8.59E-10	3.22E-09
COL19A1	8725	-4.554866877	Down	0.00072366	0.000954336
COL22A1	6359	-1.192351389	Down	1.54E-35	2.16E-34
COL25A1	2667	-1.141812345	Down	4.80E-07	1.24E-06
COL2A1	5087	-1.085944041	Down	1.33E-05	2.64E-05
COL5A3	6174	-1.183881491	Down	2.54E-58	5.96E-57
COL7A1	9169	-1.301504448	Down	3.39E-108	1.50E-106

COL9A1	3050	-1.508755749	Down	3.18E-19	2.39E-18
COLGALT1	3707	-1.173911023	Down	1.28E-07	3.63E-07
COQ8B	2443	-3.165300065	Down	3.09E-11	1.34E-10
COQ9	1689	-2.399713793	Down	4.50E-06	9.81E-06
CPNE2	2238	-1.02193589	Down	0.000404394	0.000570722
CPNE5	2583	-5.094144102	Down	3.31E-05	6.06E-05
CPNE6	2235	-6.02328106	Down	8.14E-09	2.72E-08
CPSF4	1661	-6.017798364	Down	8.67E-09	2.89E-08
CPT1B	2578	-4.918048656	Down	0.000101454	0.000165639
CRACR2A	2289	-4.734548095	Down	2.95E-07	7.89E-07
CREBBP	10082	-9.007398936	Down	2.15E-47	4.06E-46
CREBZF	7575	-1.72920095	Down	9.60E-08	2.76E-07
cryaa	1144	-1.595729615	Down	1.90E-05	3.66E-05
CSDC2	2530	-1.086867522	Down	6.19E-05	0.000106618
csnk1e	2642	-2.640359043	Down	5.38E-12	2.51E-11
CSNK2B	1123	-5.335225066	Down	5.82E-06	1.24E-05
CSTB	940	-5.48722816	Down	1.70E-06	3.99E-06
CTDP1	3590	-2.712034896	Down	6.32E-07	1.60E-06
CTH	2044	-5.413227578	Down	3.13E-06	7.04E-06
CTPS1	3237	-5.094144102	Down	3.31E-05	6.06E-05
CTSW	1296	-3.557617488	Down	8.89E-06	1.83E-05
CUEDC2	1196	-5.557617488	Down	9.23E-07	2.26E-06
CUL3	6592	-1.84952683	Down	9.67E-16	5.94E-15
CUL4A	4043	-3.902265659	Down	2.37E-10	9.40E-10
CUL4B	5182	-1.171842911	Down	7.09E-13	3.54E-12
CWC27	1986	-1.949571374	Down	9.29E-05	0.000153005
CXCL12	524	-3.335225066	Down	6.14E-05	0.00010573
CXCL9	2708	-6.02328106	Down	8.14E-09	2.72E-08
CXXC1	2966	-3.111903226	Down	8.52E-45	1.53E-43
CYP26B1	4556	-2.520780719	Down	1.74E-14	9.84E-14
CYTH2	4604	-4.28600912	Down	1.66E-97	6.57E-96
DAAM2	6247	-8.403704804	Down	5.64E-34	7.57E-33
DAB2IP	5471	-1.7828416	Down	2.72E-08	8.47E-08
DACT3	2574	-1.013337036	Down	0.00027065	0.000398166
DAZAP2	2166	-3.947862525	Down	9.86E-05	0.000161311
DCAF11	3656	-5.971108414	Down	1.48E-08	4.77E-08
DCAF12	3671	-1.543811688	Down	1.19E-09	4.38E-09
DCAF6	3524	-8.024896968	Down	1.67E-27	1.80E-26
DCANP1	3135	-4.522852069	Down	3.67E-21	3.04E-20
DCLK3	5344	-3.750262566	Down	0.000617248	0.000829661
DCP1B	2116	-3.02328106	Down	1.13E-06	2.75E-06
DDX23	3267	-3.165300065	Down	1.76E-07	4.88E-07

DDX24	2948	-1.298622871	Down	6.82E-05	0.000116135
DDX3X	4645	-5.398728009	Down	3.52E-06	7.84E-06
DDX54	4378	-4.0514321	Down	0.000114374	0.000184979
DEAF1	2735	-4.982923322	Down	6.81E-05	0.000115971
DEDD2	2010	-5.07219066	Down	3.83E-05	6.89E-05
DEF6	2296	-5.217542046	Down	1.40E-05	2.77E-05
depdc1b	2318	-4.750262566	Down	0.000265439	0.00039105
DEXI	1501	-1.038800534	Down	0.000336975	0.000484675
DGKD	6294	-6.998190079	Down	2.16E-15	1.30E-14
DGKQ	4643	-1.052825336	Down	0.000139357	0.000219573
DGKZ	4086	-1.272484147	Down	8.95E-06	1.84E-05
DHRS1	1480	-3.624731683	Down	9.41E-11	3.89E-10
dhx37	4551	-1.371109602	Down	7.06E-13	3.53E-12
DHX40	3667	-6.659715675	Down	1.09E-12	5.40E-12
DKK3	2578	-3.128774189	Down	9.74E-09	3.22E-08
DLL3	2383	-3.78979093	Down	1.60E-09	5.81E-09
DMBX1	2884	-4.865739783	Down	0.000138382	0.000218116
DMPK	2722	-4.813765508	Down	0.000186577	0.000285937
DMRT3	2180	-1.414213362	Down	4.09E-14	2.25E-13
DNAJB13	1875	-3.750262566	Down	0.000617248	0.000829661
DNAJB4	2982	-1.099267458	Down	2.54E-32	3.23E-31
DNAJB5	2446	-2.366933926	Down	0.000143456	0.000225337
DOCK8	7237	-1.285594299	Down	2.06E-07	5.66E-07
dpf1	2329	-5.637787836	Down	4.47E-07	1.16E-06
DPT	1728	-3.335225066	Down	6.14E-05	0.00010573
DPYSL5	5126	-6.284656242	Down	3.08E-10	1.21E-09
DRD3	1557	-1.853356059	Down	0.000258042	0.000382606
DTX2	2122	-1.202145793	Down	0.000241333	0.000360191
DYNC1I2	4333	-4.584838956	Down	0.000625251	0.000839624
DYSF	6679	-1.721693413	Down	5.11E-06	1.10E-05
E2F8	3522	-5.688862021	Down	2.76E-07	7.43E-07
EARS2	1744	-4.409810892	Down	2.06E-05	3.94E-05
EBNA1BP2	1504	-2.07219066	Down	0.000216103	0.000325659
ECE1	5087	-3.252762906	Down	0.000116723	0.000188298
ECI2	1393	-6.360647663	Down	1.08E-10	4.45E-10
ECT2L	4343	-2.714332708	Down	4.04E-05	7.22E-05
EDC3	4069	-1.748070019	Down	4.24E-05	7.56E-05
EEF2KMT	2304	-5.624731683	Down	5.04E-07	1.30E-06
EFCAB2	1240	-3.239976751	Down	0.000128524	0.000205002
EID1	2091	-5.335225066	Down	5.82E-06	1.24E-05
EIF3C	3063	-6.37475343	Down	8.86E-11	3.67E-10

ELFN1	3742	-3.10389952	Down	1.14E-05	2.30E-05
ELN	3726	-4.802214645	Down	0.000199136	0.000303393
ELOVL1	1626	-4.57808159	Down	0.00064635	0.000865808
EME2	1140	-2.055117147	Down	8.07E-09	2.70E-08
ENSA	2730	-2.197914429	Down	9.86E-06	2.01E-05
ENTPD1	12589	-5.214059377	Down	1.44E-05	2.84E-05
ENTPD6	2731	-1.093826411	Down	6.82E-05	0.000116207
EPB41L3	3912	-7.117400619	Down	1.79E-16	1.16E-15
EPHA2	3964	-1.904656772	Down	0.000243449	0.000363066
EPHA5	8352	-2.164608953	Down	0.0006027	0.000812944
EPHB2	10943	-6.413227578	Down	5.10E-11	2.17E-10
EPN1	2406	-4.177371856	Down	7.21E-55	1.58E-53
ERBIN	7063	-6.068820439	Down	4.77E-09	1.64E-08
ERC1	9266	-6.874590701	Down	2.41E-14	1.35E-13
ERF	2716	-3.501590458	Down	1.76E-43	3.04E-42
ERFE	2902	-3.438318559	Down	2.59E-07	7.00E-07
ERICH4	946	-1.48722816	Down	1.69E-05	3.29E-05
ERMARD	1925	-2.001801333	Down	0.000354982	0.000508177
ESPL1	6623	-2.019449198	Down	8.43E-07	2.09E-06
ESPNL	3610	-4.64427187	Down	0.00046417	0.00064619
ESR1	6356	-4.865739783	Down	0.000138382	0.000218116
ESRP1	3684	-4.335225066	Down	1.19E-05	2.38E-05
ESYT1	4247	-1.029436674	Down	0.000207572	0.00031525
ETF1	3872	-5.425827615	Down	6.39E-19	4.72E-18
ETV2	1495	-3.494283863	Down	5.18E-32	6.54E-31
EWSR1	2678	-5.72295522	Down	1.99E-07	5.47E-07
EXD3	2883	-1.387692486	Down	0.000115564	0.00018669
EXOC1	3582	-5.557617488	Down	9.23E-07	2.26E-06
EYA3	5888	-2.48452901	Down	0.000164212	0.000254874
FABP5	731	-1.205942049	Down	2.77E-05	5.16E-05
FADS6	1883	-3.07219066	Down	0.00042043	0.000591129
FAM120A	5152	-2.259030657	Down	1.42E-19	1.09E-18
Fam129c	2222	-2.848045268	Down	1.78E-09	6.43E-09
FAM131C	1701	-2.224193754	Down	6.67E-08	1.97E-07
FAM13B	5591	-7.72891583	Down	2.72E-23	2.48E-22
fam149a	2405	-5.252762906	Down	1.08E-05	2.19E-05
FAM163B	1131	-1.182787492	Down	9.36E-06	1.92E-05
FAM170B	1411	-1.025442401	Down	2.14E-08	6.75E-08
FAM208	7247	-1.872889437	Down	3.55E-06	7.90E-06

A					
fam57a	2072	-5.165300065	Down	2.03E-05	3.89E-05
FAM63A	2176	-2.811250659	Down	9.31E-06	1.91E-05
FAM71E 1	1228	-1.14535233	Down	0	0
FAM98A	2628	-6.165300065	Down	1.46E-09	5.33E-09
FANCA	5464	-4.646426755	Down	3.47E-14	1.92E-13
FBRS	4108	-1.24775593	Down	0	0
FBXL16	3543	-1.099066366	Down	2.17E-08	6.85E-08
FBXL19	3664	-1.381358429	Down	1.52E-05	2.98E-05
FBXO11	3844	-4.750262566	Down	0.000265439	0.00039105
FBXO38	4199	-3.688862021	Down	2.45E-06	5.60E-06
FBXO7	1758	-6.983434826	Down	2.91E-15	1.73E-14
FGD3	3432	-4.865739783	Down	0.000138382	0.000218116
FGFR1O P2	981	-4.865739783	Down	0.000138382	0.000218116
FHL1	2319	-4.865739783	Down	0.000138382	0.000218116
FLII	4357	-1.354716364	Down	0.00027436	0.000403179
FLNB	9434	-6.809156255	Down	8.10E-14	4.37E-13
FNDC11	1136	-1.082837905	Down	4.74E-06	1.03E-05
FOXD3	2068	-5.335225066	Down	5.82E-06	1.24E-05
FOXP4	5910	-1.508850412	Down	1.09E-134	6.07E-133
FRS2	6666	-4.557617488	Down	1.70E-06	4.00E-06
FUBP1	2563	-5.204438459	Down	1.54E-05	3.02E-05
FUS	5116	-5.393349113	Down	3.68E-06	8.17E-06
FUT3	2049	-1.34011964	Down	0.000401524	0.000567237
FUZ	1746	-1.068860957	Down	2.59E-13	1.34E-12
FXN	7176	-1.02816771	Down	4.46E-05	7.91E-05
FZD1	4350	-4.865739783	Down	0.000138382	0.000218116
FZD10	3282	-4.865739783	Down	0.000138382	0.000218116
G3BP2	4311	-1.094750118	Down	5.79E-06	1.24E-05
GABPB1	3016	-5.273824522	Down	9.28E-06	1.90E-05
GAGE8	10020	-1.239177052	Down	1.47E-05	2.90E-05
GALC	3814	-2.193451105	Down	1.32E-05	2.62E-05
GALE	1607	-5.413227578	Down	3.13E-06	7.04E-06
GALNT1 2	2735	-1.37680417	Down	0.000281739	0.000412998
GAREM2	4161	-3.643347362	Down	5.67E-18	3.98E-17
GCHFR	713	-4.165300065	Down	4.38E-05	7.78E-05
gcnt2	4672	-2.902265659	Down	7.42E-05	0.000125097
GDF5	2328	-4.865739783	Down	0.000138382	0.000218116
GDI2	2277	-1.580337564	Down	0.000124714	0.000199569
GFM1	2147	-2.07154932	Down	0.000533483	0.000728765
GGH	1492	-1.949571374	Down	9.29E-05	0.000153005

GGTLC1	974	-4.112897806	Down	1.03E-08	3.41E-08
GIPR	3181	-3.824082799	Down	0.000405828	0.000572543
GJB3	2217	-5.703838228	Down	2.39E-07	6.51E-07
GLB1L2	3155	-2.445407984	Down	2.46E-06	5.62E-06
GLIS3	7650	-4.972654987	Down	7.26E-05	0.000122618
GLRA3	3629	-2.701352965	Down	4.26E-06	9.34E-06
GLRX	1647	-1.624731683	Down	6.41E-09	2.17E-08
GNA13	6001	-5.404850861	Down	3.35E-06	7.49E-06
GNAL	5834	-7.084163302	Down	3.65E-16	2.30E-15
GOLGA8A	4263	-2.094569761	Down	5.63E-56	1.26E-54
GON4L	7775	-1.100849313	Down	0.000144283	0.000226555
GPATCH8	6959	-6.456240467	Down	2.71E-11	1.19E-10
GPC6	7102	-3.631499616	Down	0.000468488	0.00065127
GPD1	3083	-5.972654987	Down	1.45E-08	4.69E-08
GPR12	4849	-1.052825336	Down	0.000139357	0.000219573
gpr17	2305	-1.750262566	Down	0.000699489	0.000925408
GPR183	1785	-3.224193754	Down	3.25E-06	7.28E-06
GPR89A	2012	-6.208819558	Down	8.39E-10	3.14E-09
GPSM3	1456	-1.633332906	Down	2.02E-38	3.09E-37
GPT2	3958	-1.175869307	Down	1.46E-08	4.70E-08
GRID1	5849	-1.469080813	Down	3.22E-07	8.57E-07
GRINA	1968	-2.110207508	Down	1.56E-24	1.50E-23
GRK2	3444	-1.276791468	Down	9.59E-08	2.76E-07
GRM8	3860	-4.820651893	Down	0.000179431	0.000275872
GstT2	1172	-4.865739783	Down	0.000138382	0.000218116
GTF3C1	7032	-1.061191661	Down	6.53E-08	1.93E-07
GTF3C6	950	-4.750262566	Down	0.000265439	0.00039105
GTPBP2	3026	-2.02541389	Down	9.40E-08	2.71E-07
GUCD1	3452	-1.039487179	Down	3.08E-07	8.22E-07
H2AFY2	2179	-2.48722816	Down	0.000263428	0.000389591
HARS2	2438	-6.786765899	Down	1.21E-13	6.45E-13
HAS3	4219	-2.294583082	Down	2.64E-07	7.11E-07
HBS1L	2807	-1.182216664	Down	0.000107095	0.000174155
HCFC2	5735	-4.624731683	Down	0.000512547	0.000702989
HCN2	3408	-1.331310016	Down	3.09E-07	8.24E-07
HDDC2	1615	-3.972654987	Down	0.000163599	0.000253976
HDX	6188	-3.750262566	Down	0.000617248	0.000829661
HEMK1	6187	-1.305384847	Down	6.16E-12	2.86E-11
HEPACAM2	2110	-2.387692486	Down	2.48E-05	4.66E-05
HERC4	4283	-6.629641598	Down	1.80E-12	8.74E-12
HEXIM1	4785	-1.165300065	Down	4.99E-09	1.71E-08

HFE2	1692	-4.865739783	Down	0.000138382	0.000218116
HIPK2	15144	-6.798731275	Down	9.78E-14	5.23E-13
HLA-G	1578	-1.233522574	Down	5.86E-05	0.000101274
HLCS	6953	-5.048920881	Down	4.46E-05	7.91E-05
HMBOX1	3175	-5.878445967	Down	4.08E-08	1.24E-07
HMGB2	1454	-4.165300065	Down	4.38E-05	7.78E-05
HMP19	2452	-2.058384861	Down	1.56E-07	4.37E-07
HNMT	3367	-3.371630713	Down	4.57E-05	8.08E-05
HNRNPA1	1783	-4.048740257	Down	8.18E-14	4.41E-13
HNRNPLL	2779	-2.879810808	Down	2.66E-07	7.17E-07
HOOK2	2604	-2.004934588	Down	2.20E-08	6.93E-08
HPN	2365	-1.024956989	Down	6.95E-15	4.03E-14
HS3ST3A1	4199	-4.335225066	Down	1.19E-05	2.38E-05
HS3ST3B1	5400	-2.327206023	Down	8.18E-07	2.03E-06
HSPA4L	3938	-8.972912588	Down	1.66E-46	3.07E-45
HSPH1	5221	-5.403322583	Down	3.40E-06	7.57E-06
HTR4	2985	-1.972654987	Down	0.000181462	0.000278624
HYOU1	4643	-3.259086626	Down	1.28E-57	2.96E-56
ID2	1364	-1.357945143	Down	1.26E-06	3.04E-06
IDH2	1551	-3.224193754	Down	3.25E-06	7.28E-06
IDH3G	1485	-1.800731199	Down	3.05E-05	5.63E-05
IDI1	2929	-1.209145704	Down	1.24E-22	1.10E-21
IDS	7582	-4.745445549	Down	0.000272483	0.000400607
IER5	2342	-6.254882958	Down	4.59E-10	1.77E-09
IFFO1	2701	-4.865739783	Down	0.000138382	0.000218116
IFNLR1	4432	-1.131294687	Down	4.50E-25	4.42E-24
IFT88	3184	-1.190237967	Down	1.33E-05	2.64E-05
IGSF9	3996	-1.320503249	Down	6.21E-27	6.60E-26
IKZF1	5786	-2.898093796	Down	3.47E-05	6.32E-05
IL11	2378	-4.888858626	Down	0.000120788	0.000193689
IL12B	2347	-2.055117147	Down	8.07E-09	2.70E-08
IL17F	808	-1.672260054	Down	3.44E-06	7.67E-06
IL20RB	2046	-2.280777282	Down	7.69E-05	0.000129154
IL36RN	2667	-4.624731683	Down	8.96E-07	2.21E-06
IL4	618	-4.624731683	Down	0.000512547	0.000702989
IL5RA	5654	-5.07219066	Down	3.83E-05	6.89E-05
IL9R	2157	-5.628660951	Down	4.86E-07	1.26E-06
INCA1	1221	-7.415693238	Down	1.63E-19	1.25E-18
INPP5D	5189	-3.4885273	Down	1.16E-37	1.72E-36

INPP5J	3391	-3.02328106	Down	1.13E-06	2.75E-06
IQCC	2053	-1.267102858	Down	3.25E-09	1.14E-08
Iqcf6	446	-1.48722816	Down	5.05E-07	1.30E-06
IQSEC2	5238	-1.034673003	Down	1.02E-16	6.64E-16
IRGM	1659	-4.624731683	Down	0.000512547	0.000702989
ISCA1	2002	-1.361697278	Down	5.67E-05	9.84E-05
ISYNA1	2420	-1.338413922	Down	8.48E-06	1.76E-05
ITGA10	4840	-1.547786964	Down	1.70E-71	4.93E-70
ITGA2B	3431	-1.098982168	Down	5.47E-32	6.90E-31
ITGB4	5689	-1.327252224	Down	6.85E-05	0.000116645
ITPRIP	6809	-1.394118755	Down	1.70E-06	3.99E-06
IYD	7402	-2.302803589	Down	0.000253957	0.000377315
JPH2	4785	-2.357945143	Down	7.65E-06	1.59E-05
jund	1963	-1.090376946	Down	2.94E-44	5.19E-43
kat8	1529	-3.034302131	Down	1.92E-19	1.46E-18
KCNG3	3824	-7.295201247	Down	3.18E-18	2.26E-17
KCNH2	2441	-2.206271846	Down	0.000678438	0.000904622
KCNK5	3783	-1.828265078	Down	3.41E-05	6.23E-05
KCNK7	1563	-4.624731683	Down	0.000512547	0.000702989
KCNN3	11941	-1.266554256	Down	0.00016173	0.000251466
KCNQ2	3167	-5.920187567	Down	2.60E-08	8.11E-08
KCP	2774	-1.286239869	Down	0.000167351	0.000259426
kctd13	1725	-1.010240413	Down	1.82E-39	2.85E-38
KCTD20	5345	-3.9207218	Down	1.85E-07	5.10E-07
KDM1A	3035	-1.088849	Down	4.82E-10	1.85E-09
KHDRBS1	2891	-6.557617488	Down	5.74E-12	2.67E-11
KIAA0040	4584	-5.715585114	Down	2.14E-07	5.85E-07
KIAA0586	5591	-5.054773607	Down	4.30E-05	7.65E-05
KIAA0895	4465	-1.166625767	Down	2.15E-10	8.60E-10
KIAA1191	2456	-4.858787022	Down	4.59E-07	1.19E-06
KIAA1522	5327	-3.123236005	Down	8.22E-13	4.09E-12
KIAA1551	6230	-3.865739783	Down	0.00031738	0.00045908
KIAA1683	3882	-5.552111011	Down	9.69E-07	2.37E-06
KIF13A	5995	-5.79232317	Down	9.99E-08	2.87E-07
KIF13B	8766	-1.445531959	Down	7.78E-30	9.17E-29
KIF19	3643	-2.428334471	Down	8.06E-05	0.000134849

KIF1A	9223	-6.112966222	Down	2.80E-09	9.86E-09
KIF22	2257	-2.49484844	Down	3.43E-06	7.65E-06
KIF3A	6275	-6.507641462	Down	1.25E-11	5.64E-11
KIFC3	3366	-1.005896869	Down	2.80E-12	1.34E-11
KLHL38	1834	-5.750262566	Down	1.52E-07	4.25E-07
KLHL7	3175	-5.018297652	Down	5.44E-05	9.49E-05
KLK10	3106	-4.865739783	Down	0.000138382	0.000218116
KLK6	1660	-1.2702431	Down	4.31E-11	1.85E-10
KNOP1	1950	-4.624731683	Down	0.000512547	0.000702989
KPNB1	3947	-5.079864588	Down	3.64E-05	6.61E-05
KREMEN2	1978	-2.561628201	Down	1.68E-14	9.51E-14
KRT16	1717	-2.177554327	Down	3.09E-07	8.23E-07
KRT8	1796	-1.293294386	Down	3.88E-06	8.58E-06
KRT80	3894	-6.209694184	Down	8.30E-10	3.11E-09
KRT81	1910	-2.170911488	Down	6.68E-08	1.97E-07
KRTAP10-10	1100	-2.50307414	Down	8.39E-40	1.33E-38
KRTAP10-6	1238	-1.378342243	Down	5.85E-15	3.41E-14
KRTAP13-4	483	-3.165300065	Down	0.0002217	0.000333191
KRTAP5-10	1058	-1.06908475	Down	0.000638086	0.000855364
KRTAP5-3	899	-3.972654987	Down	0.000163599	0.000253976
LACTB	1976	-2.750262566	Down	0.000255065	0.00037864
LAD1	2839	-1.349969601	Down	0.00018125	0.000278376
LAIR1	2733	-1.848200502	Down	1.57E-06	3.71E-06
LAMA3	10492	-3.07219066	Down	1.00E-09	3.73E-09
LARGE2	2532	-5.920187567	Down	2.60E-08	8.11E-08
LARP1	6595	-1.469770696	Down	1.15E-11	5.23E-11
LCNL1	1836	-3.972654987	Down	0.000163599	0.000253976
LDLRAD2	4012	-2.445407984	Down	2.46E-06	5.62E-06
LENG8	3725	-1.118339463	Down	0	0
LEPROT	4625	-1.142499195	Down	2.12E-06	4.90E-06
LGALS8	6153	-4.672460414	Down	0.000401469	0.000567177
lgals9	1668	-6.901363693	Down	1.45E-14	8.23E-14
LGR5	4537	-5.571292425	Down	8.17E-07	2.03E-06
LIF	3969	-5.265436736	Down	9.88E-06	2.02E-05
LIG1	3180	-1.651076515	Down	1.32E-23	1.22E-22
LILRB1	1690	-1.827595924	Down	4.35E-10	1.68E-09
LIMA1	3741	-5.103534789	Down	4.71E-31	5.77E-30

LIMK1	3382	-1.964516917	Down	9.65E-14	5.16E-13
LIMS1	4674	-4.58618664	Down	0.000621115	0.000834629
LIN54	5864	-2.731593354	Down	9.20E-05	0.000151753
LIPG	4167	-1.545495815	Down	3.55E-05	6.46E-05
LMF2	2599	-1.407088475	Down	8.77E-63	2.22E-61
LNX1	2840	-1.678225385	Down	7.51E-06	1.57E-05
LOC200726	1376	-4.624731683	Down	0.000512547	0.000702989
LOC653602	2058	-2.749441686	Down	0.000303131	0.000442024
LOXHD1	3633	-1.813242214	Down	1.70E-06	3.99E-06
lrch4	3236	-1.11948433	Down	8.13E-108	3.56E-106
LRFN3	2515	-3.750262566	Down	0.000617248	0.000829661
LRIF1	1807	-5.610894356	Down	5.72E-07	1.46E-06
LRP1	14888	-5.526366554	Down	1.21E-06	2.93E-06
LRP5	5159	-1.109590632	Down	1.91E-07	5.26E-07
LRP5L	3605	-1.121966881	Down	3.76E-07	9.89E-07
LRRC20	3417	-2.262910861	Down	1.43E-15	8.67E-15
LRRC24	1772	-1.396080272	Down	2.97E-09	1.04E-08
LRRTM3	6051	-1.660064757	Down	0.000466494	0.000648745
LSR	2218	-2.893349521	Down	2.90E-06	6.57E-06
LZTR1	4315	-3.165300065	Down	0.0002217	0.000333191
MAB21L2	2769	-2.701352965	Down	4.26E-06	9.34E-06
MAD1L1	2360	-8.235904064	Down	6.03E-31	7.37E-30
MAL	843	-4.624731683	Down	0.000512547	0.000702989
MAL2	2819	-3.533031849	Down	0.00010997	0.000178382
MAN2B1	3221	-2.195097058	Down	1.35E-07	3.82E-07
MAP4	6036	-6.683520445	Down	7.34E-13	3.66E-12
MAP4K3	4299	-1.025535854	Down	2.25E-05	4.26E-05
MAP7D1	3471	-1.35642307	Down	1.60E-27	1.73E-26
MAPK12	1823	-1.853356059	Down	0.000258042	0.000382606
MAPK8IP2	3380	-1.95007305	Down	1.37E-05	2.72E-05
MAPT	5636	-7.391615914	Down	3.00E-19	2.26E-18
MARK2	4516	-1.73033475	Down	1.38E-08	4.47E-08
MAST3	5893	-1.321883226	Down	5.74E-41	9.36E-40
mbd6	4150	-1.363600284	Down	1.05E-23	9.75E-23
MBOAT7	2393	-2.478541956	Down	8.89E-07	2.19E-06
MCFD2	4324	-1.19606872	Down	8.62E-11	3.58E-10
MCL1	3837	-2.0075972	Down	1.53E-27	1.66E-26
MCM3AP	6194	-1.012533439	Down	0.0001931	0.000295181
MCM7	3085	-1.87520792	Down	1.10E-14	6.26E-14

MCU	2886	-4.809156255	Down	1.33E-07	3.75E-07
MDGA2	5248	-1.750262566	Down	1.17E-09	4.30E-09
MDM2	6657	-4.798731275	Down	0.000203069	0.000308966
MDM4	10077	-5.245957728	Down	1.14E-05	2.30E-05
MED15	3261	-5.489390581	Down	1.67E-06	3.93E-06
MED16	2924	-5.432835863	Down	2.67E-06	6.08E-06
MED29	3661	-5.192100124	Down	1.68E-05	3.28E-05
MEF2A	5618	-4.972654987	Down	7.26E-05	0.000122618
MEGF8	10966	-1.058789828	Down	9.95E-46	1.82E-44
MEN1	3165	-5.165300065	Down	2.03E-05	3.89E-05
MEPCE	1882	-6.560705669	Down	5.47E-12	2.55E-11
MEST	2463	-1.573851881	Down	0.000152319	0.000237938
MFAP4	1852	-2.280777282	Down	7.69E-05	0.000129154
MGA	11415	-7.205534982	Down	2.55E-17	1.73E-16
MGAT1	2926	-4.57808159	Down	0.00064635	0.000865808
MGAT5B	4244	-6.377869404	Down	8.48E-11	3.52E-10
MIB2	2936	-2.407563192	Down	5.15E-06	1.11E-05
MIDN	3793	-1.02675762	Down	3.13E-92	1.18E-90
MIEF1	4695	-1.568930247	Down	1.04E-47	1.98E-46
MKRN2 OS	1084	-5.165300065	Down	2.03E-05	3.89E-05
MLEC	6371	-6.335225066	Down	1.54E-10	6.24E-10
MLLT1	4505	-1.397156664	Down	7.48E-15	4.33E-14
MLX	2562	-1.173815871	Down	0.000192733	0.000294703
MMAB	4138	-1.063420451	Down	1.08E-08	3.56E-08
MNAT1	1383	-4.750262566	Down	0.000265439	0.00039105
MNX1	1620	-1.448762219	Down	9.44E-10	3.52E-09
MOB3C	2822	-7.260591585	Down	7.19E-18	5.03E-17
MON2	10379	-5.335225066	Down	5.82E-06	1.24E-05
MOSPD3	1104	-2.029243027	Down	3.63E-16	2.29E-15
MPG	1039	-3.750262566	Down	0.000617248	0.000829661
mplkip	941	-1.195047408	Down	0.000597292	0.000806469
MPPE1	2961	-2.328154593	Down	0.000203371	0.000309394
MPZL1	5010	-5.335225066	Down	5.82E-06	1.24E-05
MRFAP1	2063	-5.146239331	Down	2.32E-05	4.39E-05
MRPS34	1024	-1.746399786	Down	7.09E-104	2.97E-102
MRVI1	6313	-8.035664784	Down	1.14E-27	1.24E-26
MS4A14	3400	-6.256153495	Down	4.52E-10	1.74E-09
MSH2	3025	-1.701352965	Down	0.000573219	0.000775922
MSH4	3277	-2.413227578	Down	0.00047376	0.000657454
MSH6	4158	-5.165300065	Down	2.03E-05	3.89E-05
MSI1	2975	-1.327446789	Down	3.95E-21	3.27E-20
MSL3	2262	-5.165300065	Down	2.03E-05	3.89E-05
MST1R	4535	-2.971099958	Down	3.83E-39	5.96E-38

MTMR3	8992	-6.335225066	Down	1.54E-10	6.24E-10
MTUS1	3803	-6.322343775	Down	1.84E-10	7.41E-10
MUC19	24831	-8.758130809	Down	2.48E-41	4.09E-40
MUL1	2458	-4.07219066	Down	8.45E-05	0.000140663
MVB12A	1384	-1.145245984	Down	1.40E-06	3.34E-06
MVK	1921	-1.827769172	Down	4.83E-15	2.83E-14
MYBPC1	3910	-1.819402266	Down	8.06E-05	0.000134849
MYL12B	947	-2.343362771	Down	2.69E-08	8.37E-08
MYO19	4422	-5.177868738	Down	1.86E-05	3.59E-05
MYOCD	8564	-5.252762906	Down	1.08E-05	2.19E-05
MYOD1	1806	-5.413227578	Down	3.13E-06	7.04E-06
MYT1	5535	-5.07219066	Down	3.83E-05	6.89E-05
NAGK	1801	-1.750262566	Down	0.000699489	0.000925408
NAPEPLD	5062	-2.667800405	Down	1.38E-09	5.07E-09
NCALD	3569	-4.750262566	Down	0.000265439	0.00039105
NCAM1	6070	-5.624731683	Down	5.04E-07	1.30E-06
NCAPH2	1425	-5.123695549	Down	2.07E-14	1.16E-13
NCKAP1L	3764	-3.865739783	Down	4.47E-10	1.72E-09
NCOA7	6203	-4.624731683	Down	0.000512547	0.000702989
NCOR2	8827	-5.252762906	Down	1.08E-05	2.19E-05
NDRG2	2237	-5.537555713	Down	1.10E-06	2.67E-06
NDUFAF3	943	-5.250214725	Down	1.11E-05	2.23E-05
NDUFAF5	2292	-5.413227578	Down	3.13E-06	7.04E-06
NDUFB9	684	-3.07219066	Down	0.00042043	0.000591129
NECAB2	1606	-4.624731683	Down	0.000512547	0.000702989
NEDD4	7022	-4.335225066	Down	1.19E-05	2.38E-05
NEIL1	1820	-2.154389791	Down	2.81E-11	1.23E-10
NEK10	2101	-6.932954863	Down	7.89E-15	4.55E-14
NEURL1B	5694	-4.809156255	Down	1.33E-07	3.75E-07
NEXN	3197	-5.626697655	Down	4.95E-07	1.28E-06
NGFR	3420	-1.165300065	Down	4.02E-06	8.85E-06
NIF3L1	1275	-5.253611301	Down	1.08E-05	2.18E-05
NIPSNAP3B	5935	-5.07219066	Down	3.83E-05	6.89E-05
NKX2-3	2096	-3.335225066	Down	6.14E-05	0.00010573
NLN	8661	-4.750262566	Down	0.000265439	0.00039105
NLRP1	5523	-6.165300065	Down	1.46E-09	5.33E-09
NLRP4	3674	-1.139304856	Down	0.000463589	0.000645484
NME9	2133	-4.165300065	Down	4.38E-05	7.78E-05

NONO	2887	-3.07219066	Down	0.00042043	0.000591129
NOP9	5877	-5.256999899	Down	1.05E-05	2.14E-05
NOXO1	1636	-1.017763183	Down	6.25E-08	1.85E-07
NPIPA1	1102	-1.424701763	Down	1.18E-15	7.20E-15
NPIPA2	1088	-1.537547528	Down	1.59E-86	5.63E-85
NPIPA5	1088	-1.524946963	Down	2.18E-52	4.59E-51
NPIPA7	1159	-1.396827251	Down	2.90E-51	5.92E-50
NPIPA8	1159	-1.396827251	Down	2.90E-51	5.92E-50
NPIPB4	4049	-1.060979619	Down	2.21E-217	2.04E-215
NPIPB5	3816	-1.045048128	Down	0	0
NPIPB9	1405	-9.173224356	Down	6.90E-52	1.43E-50
NPM1	1449	-4.838856489	Down	0.000161703	0.000251442
NPPA	854	-1.143708433	Down	9.36E-21	7.61E-20
NQO2	1158	-3.119496375	Down	3.29E-07	8.72E-07
NREP	2201	-6.271313302	Down	3.69E-10	1.43E-09
NRG4	2182	-2.165300065	Down	1.96E-07	5.39E-07
NRP2	6596	-3.441319096	Down	1.25E-06	3.01E-06
NRROS	2553	-5.252762906	Down	1.08E-05	2.19E-05
NSMF	3573	-1.600076187	Down	1.58E-21	1.33E-20
NT5C3B	1422	-5.165300065	Down	2.03E-05	3.89E-05
NUDT22	897	-1.563586307	Down	7.28E-12	3.36E-11
NUGGC	3887	-1.638307633	Down	1.74E-06	4.07E-06
NUMA1	7405	-1.327356975	Down	4.06E-247	4.10E-245
NUMBL	3366	-2.612760228	Down	0	0
NUP205	6265	-1.372895484	Down	7.43E-08	2.18E-07
NUP62	3226	-4.615521561	Down	0.000536846	0.000732995
NUTM2G	2516	-1.261581602	Down	7.26E-05	0.00012267
NXF1	4147	-1.207256393	Down	1.38E-11	6.19E-11
NXPH1	2921	-2.165300065	Down	0.000233796	0.000349812
OAS2	3539	-4.91590657	Down	0.000102772	0.000167616
OAZ2	1934	-2.48722816	Down	0.000263428	0.000389591
OGDH	4290	-1.459033969	Down	1.60E-75	4.87E-74
OLFML3	1832	-2.039769183	Down	0.000695125	0.000920328
OR13H1	927	-4.624731683	Down	0.000512547	0.000702989
OR2L3	939	-1.188866336	Down	0.000124592	0.000199395
OR2T8	939	-2.001801333	Down	0.000354982	0.000508177
OR5J2	939	-4.750262566	Down	0.000265439	0.00039105
OR8B2	942	-4.809156255	Down	0.000191501	0.000293003
OR9Q1	2438	-3.750262566	Down	0.000617248	0.000829661
ORC4	6598	-7.34102425	Down	1.05E-18	7.69E-18
OSMR	5539	-5.227939893	Down	1.30E-05	2.58E-05
OTOF	6958	-1.624731683	Down	0.000377597	0.000537954
OTOG	8742	-1.259806183	Down	2.23E-76	6.89E-75
OXR1	4476	-1.529323507	Down	6.09E-07	1.55E-06

PABPC4	3117	-1.643347362	Down	9.75E-05	0.000159892
PACRGL	1874	-4.575369757	Down	0.000654992	0.000876287
PACS2	6694	-1.75275136	Down	4.67E-20	3.66E-19
PAF1	2074	-1.912823851	Down	8.82E-05	0.000146144
PAK6	2546	-7.154439072	Down	8.00E-17	5.26E-16
PALD1	4569	-2.509254466	Down	2.71E-11	1.18E-10
PANX2	3052	-4.865739783	Down	0.000138382	0.000218116
PAQR6	2206	-5.165300065	Down	2.03E-05	3.89E-05
PARP10	3522	-1.891125101	Down	6.46E-06	1.37E-05
PARP11	4499	-4.624731683	Down	0.000512547	0.000702989
PARVB	1541	-5.48722816	Down	1.70E-06	3.99E-06
PARVG	3523	-2.559066122	Down	7.66E-13	3.82E-12
PAX4	2010	-2.287290589	Down	2.36E-11	1.04E-10
PCCA	2423	-2.48722816	Down	0.000263428	0.000389591
PCDH11X	8214	-5.413227578	Down	3.13E-06	7.04E-06
PCDHA10	4631	-3.133775976	Down	1.67E-06	3.94E-06
PCDHGC3	4724	-5.305260634	Down	7.32E-06	1.53E-05
PCDHGC4	4623	-1.638529484	Down	4.79E-27	5.11E-26
PCGF6	2005	-1.527870144	Down	1.37E-05	2.71E-05
PCNX3	6566	-1.217388576	Down	3.44E-09	1.20E-08
PCYT1A	2195	-1.110509236	Down	1.38E-06	3.28E-06
pde1b	3021	-6.110626842	Down	2.88E-09	1.01E-08
PDE2A	4316	-5.252762906	Down	1.08E-05	2.19E-05
PDE5A	6820	-7.920187567	Down	6.17E-26	6.28E-25
PDE6D	1087	-1.902265659	Down	0.000155262	0.000242096
PECAM1	4453	-4.971624122	Down	7.31E-05	0.000123339
PELI3	2511	-1.352927068	Down	0.000542111	0.000738919
PES1	2295	-2.889891397	Down	2.30E-54	5.01E-53
PEX3	2772	-4.750262566	Down	0.000265439	0.00039105
PEX5	3172	-4.993119089	Down	6.39E-05	0.000109683
PEX6	3234	-5.750262566	Down	1.52E-07	4.25E-07
PFAS	5385	-2.408597953	Down	4.88E-31	5.98E-30
PFKFB4	3505	-6.194311151	Down	1.01E-09	3.76E-09
PFN3	530	-1.610084908	Down	1.68E-05	3.27E-05
PGA4	1383	-1.844654936	Down	0.000440367	0.000616533
PGD	2419	-4.865739783	Down	0.000138382	0.000218116
PHACTR2	9531	-5.07219066	Down	3.83E-05	6.89E-05
PHF1	2189	-4.885673426	Down	8.17E-45	1.47E-43
PHF12	2957	-1.412662057	Down	3.96E-05	7.10E-05

PHF21A	7178	-1.379610516	Down	5.49E-11	2.33E-10
PHGDH	2015	-1.396625611	Down	4.76E-05	8.40E-05
PHPT1	1195	-5.333621181	Down	5.89E-06	1.26E-05
PI4K2A	4187	-1.204828429	Down	2.18E-05	4.13E-05
PIANP	2739	-1.994811401	Down	4.64E-11	1.98E-10
PIFO	2260	-5.165300065	Down	2.03E-05	3.89E-05
pigbos1	1015	-6.345209155	Down	1.34E-10	5.47E-10
PIGG	3449	-4.747856068	Down	0.000268937	0.000395739
PIGN	4798	-5.688862021	Down	2.76E-07	7.43E-07
PIH1D1	1246	-5.066408308	Down	3.98E-05	7.14E-05
PIK3R5	4380	-1.299670947	Down	9.09E-17	5.96E-16
PITPNM3	7043	-1.365826574	Down	8.00E-17	5.26E-16
PITX3	1388	-2.165300065	Down	0.000233796	0.000349812
PKHD1	16235	-1.292461644	Down	5.12E-06	1.11E-05
PKIG	1180	-4.624731683	Down	0.000512547	0.000702989
PKN1	3080	-5.809156255	Down	8.41E-08	2.44E-07
PLA2G4B	2735	-4.602705377	Down	3.26E-17	2.19E-16
PLA2G6	3016	-4.972654987	Down	7.26E-05	0.000122618
PLAC8	1432	-5.85683423	Down	5.13E-08	1.54E-07
plcb3	5527	-1.105494076	Down	1.93E-05	3.72E-05
PLCG1	5205	-1.028824046	Down	1.60E-09	5.82E-09
PLEC	15255	-2.053068507	Down	1.12E-07	3.19E-07
PLEKHB1	2121	-6.103115234	Down	3.15E-09	1.11E-08
PLEKHH3	3061	-1.42433254	Down	3.04E-61	7.47E-60
PLEKHJ1	1150	-1.287622011	Down	9.50E-05	0.000155998
PLEKHO2	3721	-1.029205674	Down	6.46E-10	2.45E-09
PLVAP	2304	-6.07219066	Down	4.58E-09	1.58E-08
PLXNB1	7143	-1.855778612	Down	2.84E-29	3.29E-28
PLXNB2	6335	-1.197890565	Down	4.55E-63	1.16E-61
PMEPA1	4918	-5.111095022	Down	2.95E-05	5.46E-05
PMFBP1	3675	-6.46395838	Down	2.42E-11	1.06E-10
PNOC	1006	-2.34534181	Down	3.93E-05	7.05E-05
PNPLA1	2367	-4.972654987	Down	7.26E-05	0.000122618
PNPLA2	2427	-3.750262566	Down	0.000617248	0.000829661
POC1A	2196	-8.165863508	Down	9.25E-30	1.09E-28
POLD1	3464	-1.170057659	Down	3.91E-14	2.15E-13
POLR2A	6730	-1.023098821	Down	4.56E-163	3.10E-161
POLR2E	2866	-5.07219066	Down	3.83E-05	6.89E-05
POU2F1	13716	-4.918048656	Down	0.000101454	0.000165639
POU2F2	6760	-2.103121074	Down	5.21E-07	1.34E-06

PPAN	1728	-1.342707475	Down	3.56E-09	1.24E-08
PPAN-P2RY11	3207	-2.194323681	Down	6.48E-07	1.64E-06
ppdpf	777	-3.307519064	Down	9.31E-10	3.47E-09
PPFIBP2	3571	-3.437820992	Down	2.61E-07	7.04E-07
PPIA	2288	-1.643487477	Down	0.000712773	0.000941846
PPIF	2200	-1.573483893	Down	6.03E-06	1.28E-05
PPM1F	5185	-1.705868446	Down	0.000289523	0.00042349
PPM1M	2197	-1.282894739	Down	3.60E-20	2.85E-19
ppp1cb	4986	-5.236547884	Down	1.22E-05	2.44E-05
PPP1R12A	5337	-2.227955673	Down	1.18E-07	3.36E-07
PPP1R16A	2274	-1.052206286	Down	1.42E-11	6.37E-11
PPP1R9A	10519	-5.557617488	Down	9.23E-07	2.26E-06
PPP3R1	3011	-1.598259472	Down	6.98E-06	1.46E-05
PRB2	1428	-1.828761275	Down	0.000287797	0.000421232
PRB4	918	-4.226212114	Down	3.11E-09	1.09E-08
Prdm11	10744	-5.07219066	Down	3.83E-05	6.89E-05
PRDM6	3027	-5.165300065	Down	2.03E-05	3.89E-05
PRELID1	1240	-2.488699547	Down	0.000300087	0.000437919
PRELP	5809	-3.07219066	Down	0.00042043	0.000591129
PREX1	6633	-1.041730866	Down	3.78E-08	1.16E-07
PRKAA2	9352	-1.78979093	Down	5.58E-05	9.69E-05
PRKACA	2673	-4.923389999	Down	9.82E-05	0.000160975
PRKAR1A	4435	-1.887558729	Down	6.43E-11	2.71E-10
prkd2	3322	-3.446586175	Down	0.000340406	0.000488983
PRM2	727	-1.071995398	Down	1.57E-05	3.08E-05
PRMT6	2652	-4.865739783	Down	0.000138382	0.000218116
PRMT9	3528	-7.315047184	Down	1.97E-18	1.42E-17
PROK1	1372	-1.56969032	Down	5.13E-05	9.00E-05
PROM2	4732	-2.032801216	Down	1.31E-05	2.61E-05
PROSER3	2147	-2.072699458	Down	2.76E-84	9.54E-83
PROX1	8500	-1.179952913	Down	0.000723207	0.000953824
PRPS1	1748	-3.143495695	Down	7.65E-06	1.59E-05
PRPSAP1	2359	-3.624731683	Down	4.66E-06	1.01E-05
PRRT1	1896	-4.252762906	Down	2.28E-05	4.31E-05
PRSS55	1129	-5.40026116	Down	3.48E-06	7.75E-06
PRSS8	1872	-1.646608141	Down	6.42E-19	4.74E-18
PSD	4185	-1.30348935	Down	3.39E-21	2.81E-20
PSMD1	3232	-3.406377523	Down	3.42E-05	6.24E-05
PSMD9	2352	-1.479476308	Down	1.47E-09	5.35E-09

PTCH2	3773	-1.252762906	Down	0.000278192	0.000408396
PTGDR	2942	-1.524381158	Down	5.67E-06	1.21E-05
ptger3	1963	-5.624731683	Down	5.04E-07	1.30E-06
PTGES3L-AARSD1	2174	-1.155226136	Down	1.95E-08	6.20E-08
PTHLH	1331	-4.865739783	Down	0.000138382	0.000218116
PTMS	1150	-5.962313043	Down	1.63E-08	5.24E-08
PTPN13	8577	-2.648141986	Down	3.22E-12	1.53E-11
PTPRE	5502	-5.165300065	Down	2.03E-05	3.89E-05
PTRH2	2220	-5.304442084	Down	7.37E-06	1.54E-05
PTS	934	-5.335225066	Down	5.82E-06	1.24E-05
PWWP2B	2612	-1.173781185	Down	2.34E-11	1.03E-10
PXMP4	5525	-1.052599932	Down	0.000555872	0.000755003
PYCR1	1809	-1.855864103	Down	1.08E-18	7.90E-18
PYGM	3616	-1.116863557	Down	6.45E-10	2.44E-09
QRFP	932	-1.250188962	Down	2.12E-05	4.04E-05
QTRT2	3916	-4.762235207	Down	0.000248627	0.000369965
R3HDM1	4714	-5.558304322	Down	9.17E-07	2.25E-06
RAB32	1209	-2.438318559	Down	1.40E-05	2.76E-05
RAB3IL1	2130	-7.293552217	Down	3.30E-18	2.35E-17
RAB43	4467	-4.865739783	Down	0.000138382	0.000218116
RABEP1	955	-5.540339496	Down	1.07E-06	2.61E-06
RAD50	6597	-1.141052519	Down	0.00028288	0.000414559
RAD9B	1569	-2.85683423	Down	0.000108997	0.00017694
RAE1	1836	-6.768778053	Down	1.67E-13	8.79E-13
RAF1	3275	-1.465033073	Down	1.21E-13	6.45E-13
RANGAP1	3809	-2.668809656	Down	3.67E-54	7.96E-53
RAPH1	9733	-1.262288195	Down	1.23E-25	1.24E-24
RARA	3105	-5.45513453	Down	2.22E-06	5.13E-06
rasa4	5606	-5.034184338	Down	4.91E-05	8.65E-05
RASGRF1	6346	-3.153327423	Down	5.38E-05	9.39E-05
RASL11A	1543	-2.001801333	Down	0.000354982	0.000508177
RASSF3	3492	-2.902265659	Down	7.42E-05	0.000125097
RBBP6	1763	-1.254707543	Down	1.88E-76	5.81E-75
RBM26	5152	-5.788815806	Down	1.03E-07	2.96E-07
RBM4	1007	-4.596923025	Down	0.000589018	0.000795909
RCAN1	2322	-1.24905333	Down	0.000252225	0.000374918
RCCD1	2683	-1.411056479	Down	5.49E-06	1.18E-05
RCL1	1461	-3.863518543	Down	0.000321623	0.000464498
RELA	2269	-2.094670268	Down	1.66E-05	3.24E-05

REPIN1	3292	-5.921255834	Down	2.57E-08	8.02E-08
RET	4159	-1.255031536	Down	9.20E-05	0.000151717
RGPD6	7640	-1.23906897	Down	3.40E-46	6.26E-45
RGS7	2495	-4.71373669	Down	0.000323135	0.000466528
RHBDL1	1238	-2.28847989	Down	7.18E-07	1.80E-06
RHOB	2373	-3.865739783	Down	0.00031738	0.00045908
RHOBTB2	4957	-1.972654987	Down	0.000181462	0.000278624
RHOD	935	-2.785309513	Down	2.01E-06	4.66E-06
RIMBP2	6322	-1.458081814	Down	6.29E-05	0.000108086
RIOK1	2477	-3.376467592	Down	9.53E-08	2.74E-07
RMDN1	2935	-5.335225066	Down	5.82E-06	1.24E-05
RNF185	3212	-5.96956018	Down	1.50E-08	4.85E-08
RNF4	2961	-4.865739783	Down	0.000138382	0.000218116
RNPEPL1	3018	-1.232414261	Down	7.79E-09	2.61E-08
ROBO2	8688	-5.069302381	Down	3.90E-05	7.01E-05
ROBO4	3631	-6.02328106	Down	8.14E-09	2.72E-08
RPL18A	671	-4.165300065	Down	4.38E-05	7.78E-05
RPL24	562	-3.750262566	Down	0.000617248	0.000829661
RPL28	682	-5.006266769	Down	5.88E-05	0.000101584
RPS13	529	-3.48722816	Down	1.37E-07	3.87E-07
RPS24	633	-1.545495815	Down	0.000342159	0.000491182
RPS26	682	-1.280460172	Down	7.88E-07	1.96E-06
RPUSD3	1182	-2.413227578	Down	0.00047376	0.000657454
RRAD	1476	-4.972654987	Down	7.26E-05	0.000122618
RRBP1	3792	-7.119496375	Down	1.71E-16	1.11E-15
RREB1	8568	-5.307713503	Down	7.19E-06	1.50E-05
RRP1	1807	-1.064371156	Down	0.000206222	0.000313395
rsph1	1316	-2.617497981	Down	1.05E-06	2.56E-06
RSPO4	2523	-3.08345059	Down	1.25E-276	1.38E-274
RWDD4	2631	-3.486506632	Down	1.70E-05	3.31E-05
RYR1	15391	-8.751914707	Down	3.43E-41	5.65E-40
sall2	3932	-4.650726892	Down	0.000449097	0.000626936
SATB1	5556	-2.423917303	Down	1.83E-06	4.29E-06
SCAF4	4203	-1.492307264	Down	2.67E-07	7.20E-07
SCAPER	4709	-1.643347362	Down	3.57E-08	1.10E-07
SCIN	2571	-3.039769183	Down	2.14E-05	4.06E-05
SCLY	2527	-1.537268842	Down	2.45E-08	7.66E-08
SCML4	4029	-5.165300065	Down	2.03E-05	3.89E-05
SCNN1D	3054	-1.314255292	Down	2.32E-43	4.02E-42
SDHC	2693	-1.67419773	Down	1.22E-05	2.44E-05
SEC16A	9042	-1.184725775	Down	6.44E-09	2.18E-08
SEC31A	4263	-6.780009909	Down	1.37E-13	7.25E-13

SEL1L	1657	-1.353745154	Down	8.78E-08	2.54E-07
SEMA3B	2960	-2.731535357	Down	0.000127352	0.000203271
SEMA4B	3780	-2.618472693	Down	2.17E-19	1.65E-18
SEMA4D	4628	-1.99974181	Down	0.000358251	0.000512522
SEMA4F	5642	-5.480721381	Down	1.79E-06	4.20E-06
SERAC1	3969	-1.279347692	Down	4.45E-90	1.64E-88
SERF2	3429	-1.665604826	Down	1.23E-05	2.46E-05
SESTD1	10448	-4.865739783	Down	0.000138382	0.000218116
SET	2919	-6.09461766	Down	3.50E-09	1.22E-08
SETD4	1167	-4.287144363	Down	1.74E-05	3.38E-05
SEZ6L2	3215	-2.784209897	Down	1.28E-08	4.18E-08
SFI1	4109	-1.387692486	Down	3.76E-05	6.80E-05
SFTPB	2840	-1.692186499	Down	0.000710189	0.000938631
SGSM2	4867	-1.347292593	Down	1.46E-33	1.93E-32
SGTA	2315	-1.144719463	Down	7.50E-118	3.68E-116
SH2D3C	2683	-1.009478664	Down	0.000120953	0.000193939
SH3BP2	9209	-1.767767865	Down	1.74E-117	8.50E-116
SH3GL3	1797	-3.033352134	Down	6.50E-35	8.95E-34
SH3RF1	5265	-1.424581251	Down	0.000297561	0.000434436
SH3TC2	26580	-1.394608314	Down	1.16E-06	2.80E-06
SHISA5	2153	-1.026290869	Down	1.47E-08	4.76E-08
SHPRH	7333	-5.07219066	Down	3.83E-05	6.89E-05
SIGLEC1 1	3165	-5.383306215	Down	3.99E-06	8.79E-06
SIGLEC1 4	2113	-1.097486122	Down	5.90E-11	2.49E-10
SIK3	6151	-5.224482264	Down	1.33E-05	2.64E-05
SIKE1	5491	-3.750262566	Down	0.000617248	0.000829661
SIRT2	1912	-5.508707887	Down	1.41E-06	3.37E-06
SLA2	2504	-2.079635473	Down	1.96E-05	3.77E-05
SLC13A3	3898	-7.774257117	Down	6.80E-24	6.35E-23
SLC16A1 2	4622	-4.751464311	Down	0.000263707	0.000389965
SLC16A8	1896	-1.603421177	Down	1.93E-08	6.12E-08
SLC17A7	2976	-1.425370866	Down	1.07E-155	6.93E-154
SLC17A9	2592	-1.56969032	Down	5.13E-05	9.00E-05
SLC18A1	2753	-3.865739783	Down	0.00031738	0.00045908
SLC19A3	3767	-2.341566593	Down	0.000359079	0.000513522
SLC22A2 3	5928	-1.267380765	Down	6.58E-06	1.39E-05
SLC24A5	1617	-3.165300065	Down	0.0002217	0.000333191
SLC25A1 7	2332	-5.865739783	Down	4.67E-08	1.41E-07
SLC25A2	2526	-5.688862021	Down	3.66E-13	1.87E-12

7					
SLC26A9	4799	-5.413227578	Down	3.13E-06	7.04E-06
SLC29A1	2183	-5.413227578	Down	4.32E-11	1.85E-10
SLC29A4	2846	-2.402339262	Down	4.35E-06	9.51E-06
SLC2A6	2546	-5.182331145	Down	1.80E-05	3.49E-05
SLC35B2	2015	-4.869065244	Down	0.000135717	0.000215339
SLC35C1	3214	-1.400735415	Down	1.60E-07	4.46E-07
SLC35D2	1626	-5.413227578	Down	3.13E-06	7.04E-06
SLC38A6	1751	-7.224914921	Down	1.64E-17	1.13E-16
SLC41A3	1692	-5.796404347	Down	9.58E-08	2.76E-07
SLC44A3	2394	-5.07219066	Down	3.83E-05	6.89E-05
SLC45A1	2527	-4.750262566	Down	0.000265439	0.00039105
SLC46A1	6490	-7.47272859	Down	3.74E-20	2.95E-19
slc4a5	6348	-5.069302381	Down	3.90E-05	7.01E-05
SLC51A	1455	-3.290395285	Down	6.58E-05	0.000112726
SLC51B	940	-1.532082396	Down	3.31E-05	6.05E-05
SLC6A13	1950	-3.07219066	Down	0.00042043	0.000591129
SLC6A16	2933	-1.037466397	Down	6.87E-28	7.58E-27
SLC6A6	1301	-1.607748013	Down	8.90E-14	4.78E-13
SLC6A7	3739	-1.684674224	Down	3.12E-05	5.74E-05
slc9a3r2	2194	-5.252762906	Down	1.08E-05	2.19E-05
SLC9A5	3735	-1.504101978	Down	0.000124854	0.000199756
SLFNL1	1877	-2.325161691	Down	4.05E-14	2.23E-13
SLX1A	828	-5.766996581	Down	1.29E-07	3.64E-07
slx1b	828	-5.766996581	Down	1.29E-07	3.64E-07
SMARCAD1	3936	-4.77529136	Down	0.00023138	0.000346622
SMARCC2	5336	-6.151255819	Down	1.74E-09	6.31E-09
SMIM21	1834	-1.491805726	Down	6.30E-05	0.000108274
SMPD4	3833	-1.049927711	Down	1.62E-05	3.16E-05
SMTN	3228	-6.263332148	Down	4.10E-10	1.59E-09
SNAPC4	4650	-1.45160425	Down	1.21E-08	3.97E-08
SNPH	5596	-2.474860834	Down	2.37E-22	2.07E-21
SNX1	8120	-4.870172029	Down	0.000134841	0.000214072
SNX17	2400	-1.290752676	Down	2.26E-52	4.74E-51
SNX21	3173	-4.611556295	Down	0.000547616	0.00074589
SOCS6	5846	-4.750262566	Down	2.50E-07	6.79E-07
SORBS2	3993	-4.999202142	Down	6.14E-05	0.00010584
SORL1	10973	-1.04330954	Down	0.000289432	0.000423427
SOX9	3934	-1.294033379	Down	3.27E-21	2.71E-20
SPAG4	1487	-5.252762906	Down	1.08E-05	2.19E-05
SPATA2	4057	-6.922056516	Down	9.75E-15	5.59E-14
SPATA20	2719	-9.453335572	Down	2.05E-60	4.96E-59

SPIRE2	3244	-1.494382034	Down	5.78E-06	1.23E-05
SPOCD1	2501	-5.239976751	Down	1.19E-05	2.39E-05
SPOCK2	1284	-3.11905588	Down	5.50E-06	1.18E-05
SPRY3	8950	-3.07219066	Down	0.00042043	0.000591129
SPSB3	1544	-1.233172178	Down	9.38E-62	2.33E-60
SPTA1	8017	-2.10389952	Down	5.66E-07	1.44E-06
SPTAN1	7907	-7.435268265	Down	9.90E-20	7.66E-19
SPTBN5	11723	-2.209694184	Down	1.52E-22	1.34E-21
SRC	4038	-1.993119089	Down	1.95E-09	7.02E-09
SRSF7	2453	-5.014298495	Down	5.58E-05	9.70E-05
SSBP4	1721	-3.037224614	Down	3.95E-13	2.01E-12
SSFA2	2645	-5.07794993	Down	3.69E-05	6.69E-05
SSH2	9483	-5.688862021	Down	2.76E-07	7.43E-07
ST5	4544	-5.657153161	Down	3.73E-07	9.81E-07
STAT3	4973	-3.482782255	Down	0.00053772	0.00073364
STEAP4	3945	-5.142579988	Down	2.38E-05	4.49E-05
STH	445	-2.667800405	Down	0.000470282	0.000653455
STK19	1620	-1.577894473	Down	2.71E-09	9.55E-09
STK25	2296	-4.972654987	Down	7.26E-05	0.000122618
STK31	3600	-4.972654987	Down	7.26E-05	0.000122618
STK32C	2129	-2.723295518	Down	4.29E-10	1.66E-09
STK39	3274	-1.08610484	Down	0.000676639	0.00090247
STMN3	2351	-1.788513556	Down	6.04E-05	0.000104232
STRADB	2322	-4.59424641	Down	0.000596881	0.000806012
STRIP2	5114	-1.044877025	Down	0.000194586	0.000297319
STT3A	4103	-2.48722816	Down	0.000263428	0.000389591
STX16	4904	-3.611792628	Down	1.45E-06	3.44E-06
STX2	3338	-1.494655178	Down	0.000278766	0.000409214
STXBP2	1886	-7.157615317	Down	7.46E-17	4.92E-16
STYXL1	931	-8.886399254	Down	2.37E-44	4.20E-43
SUGT1	1690	-1.972654987	Down	2.85E-05	5.28E-05
SWI5	981	-1.469499504	Down	6.02E-09	2.04E-08
SYCP1	3521	-5.042062556	Down	4.67E-05	8.24E-05
SYNM	6418	-1.518937019	Down	6.42E-05	0.000110243
SYNPO2L	4131	-5.59424641	Down	6.65E-07	1.68E-06
SYT5	1775	-1.228431896	Down	1.99E-07	5.46E-07
SYT6	4352	-2.667800405	Down	7.59E-07	1.89E-06
SYT7	6175	-4.865739783	Down	0.000138382	0.000218116
SYVN1	3051	-2.620744716	Down	1.26E-20	1.02E-19
TAC3	784	-2.688862021	Down	4.41E-05	7.82E-05
TAF6	2762	-4.527769954	Down	2.25E-06	5.18E-06
TAMM41	1297	-4.865739783	Down	0.000138382	0.000218116
TARP	1027	-3.422102517	Down	7.46E-10	2.81E-09

TAS2R60	957	-4.750262566	Down	0.000265439	0.00039105
TBATA	1669	-2.106327693	Down	2.88E-32	3.66E-31
TBC1D16	9791	-4.865739783	Down	0.000138382	0.000218116
TBC1D3	2260	-1.027480406	Down	7.74E-304	9.13E-302
TBC1D3B	1227	-1.124692661	Down	6.24E-276	6.84E-274
TBC1D3C	899	-1.301042167	Down	0	0
TBC1D3E	961	-1.113212669	Down	0	0
TBC1D3F	1816	-1.347099128	Down	1.48E-100	6.01E-99
TBC1D3K	1955	-1.565935179	Down	7.74E-276	8.47E-274
TBL3	2594	-1.003433648	Down	1.01E-31	1.26E-30
TBX3	4723	-1.452876655	Down	2.05E-07	5.64E-07
TCEA1	2787	-4.865739783	Down	0.000138382	0.000218116
TCF12	4714	-5.413227578	Down	3.13E-06	7.04E-06
TCF19	3306	-4.889950337	Down	0.000120009	0.000192574
TCF7	3277	-4.428334471	Down	5.44E-06	1.17E-05
TCF7L1	2918	-1.417784551	Down	1.46E-31	1.82E-30
TDRD5	3956	-5.123214663	Down	2.72E-05	5.07E-05
TEAD2	2215	-1.529443318	Down	4.29E-09	1.48E-08
TERB1	2446	-1.660064757	Down	0.000466494	0.000648745
TESK1	2433	-4.823879715	Down	0	0
TFAM	5206	-1.150633287	Down	0.000116664	0.000188298
tfcp2	3712	-2.815381486	Down	0.000169324	0.00026191
TFIP11	2848	-2.08746757	Down	5.12E-19	3.80E-18
TGFA	4307	-5.116701357	Down	2.84E-05	5.28E-05
Tgfbr3l	1256	-1.832724726	Down	7.48E-07	1.87E-06
thap7	1242	-2.413227578	Down	0.00047376	0.000657454
THBS3	3229	-6.48722816	Down	1.70E-11	7.58E-11
THEMIS2	2724	-2.335225066	Down	1.32E-07	3.73E-07
THY1	2065	-5.103585857	Down	3.11E-05	5.72E-05
TIE1	3982	-4.972654987	Down	7.26E-05	0.000122618
TIFAB	1273	-3.165300065	Down	6.10E-06	1.30E-05
TIMM9	903	-4.946659778	Down	0.000440874	0.000617145
TIMP1	916	-5.48722816	Down	1.70E-06	3.99E-06
TIRAP	2348	-1.03569987	Down	2.62E-06	5.97E-06
TJAP1	2572	-2.009649194	Down	1.95E-10	7.83E-10
TJP3	3110	-2.2119933	Down	1.89E-41	3.12E-40
TKTL2	2801	-2.07219066	Down	6.31E-05	0.000108503
TLE1	4123	-5.165300065	Down	2.03E-05	3.89E-05

TLR9	3870	-2.02328106	Down	0.000107318	0.000174466
TLX3	1493	-3.972654987	Down	0.000163599	0.000253976
tmc8	4419	-1.380068911	Down	9.00E-08	2.60E-07
TMED7	4063	-5.225347449	Down	1.33E-05	2.63E-05
TMEM189	2286	-6.413227578	Down	5.10E-11	2.17E-10
TMEM191C	1307	-1.55735476	Down	6.44E-179	4.83E-177
TMEM246	4285	-5.056719252	Down	4.24E-05	7.56E-05
TMEM259	2608	-1.191600416	Down	8.52E-32	1.07E-30
TMEM262	480	-3.972654987	Down	0.000163599	0.000253976
TMEM266	2399	-1.020102149	Down	6.94E-05	0.00011807
TMEM31	732	-1.674217611	Down	1.73E-05	3.36E-05
TMEM8B	2153	-2.956276594	Down	6.61E-06	1.40E-05
TMOD2	9184	-5.48722816	Down	1.70E-06	3.99E-06
TMPPE	3456	-2.828265078	Down	0.000137834	0.000218116
TMPRSS13	2233	-1.547321672	Down	1.28E-07	3.61E-07
TNFRSF10D	3535	-4.972654987	Down	7.26E-05	0.000122618
TNK2	4476	-5.273824522	Down	9.28E-06	1.90E-05
TNPO3	4220	-5.444028278	Down	2.44E-06	5.58E-06
TNRC18	10570	-1.466365275	Down	0	0
TNXB	3125	-4.085923024	Down	5.50E-26	5.61E-25
TOM1	2392	-1.490174279	Down	4.09E-07	1.07E-06
TOM1L2	5736	-5.750262566	Down	1.52E-07	4.25E-07
TOR2A	2267	-5.556509001	Down	0.000112192	0.000181718
TOX	4131	-1.715497147	Down	3.81E-05	6.87E-05
TOX2	2489	-2.903707633	Down	7.33E-05	0.000123764
TP53TG3C	869	-1.105542567	Down	1.09E-09	4.05E-09
TP73	4738	-3.750262566	Down	0.000617248	0.000829661
TPCN1	4948	-1.56173121	Down	6.16E-10	2.34E-09
TPD52L2	2310	-4.972654987	Down	7.26E-05	0.000122618
TPSB2	1166	-3.865739783	Down	0.00031738	0.00045908
TRAF6	7928	-1.581323165	Down	8.71E-06	1.80E-05
TRIM17	1782	-1.325824747	Down	8.92E-84	3.03E-82
TRIM24	3902	-7.293758449	Down	3.29E-18	2.34E-17
TRIM28	2959	-1.063620822	Down	6.03E-144	3.60E-142
TRIM46	3251	-1.379128567	Down	2.12E-07	5.81E-07

TRIP12	9875	-6.521796053	Down	1.00E-11	4.58E-11
TRMT12	2219	-1.098185869	Down	0.000275268	0.000404378
TRMT2A	2840	-6.552111011	Down	6.26E-12	2.90E-11
TRMT44	2846	-4.809156255	Down	1.33E-07	3.75E-07
TRPM5	3928	-1.972654987	Down	7.04E-14	3.81E-13
TRPM8	5621	-3.972654987	Down	0.000163599	0.000253976
TRPT1	969	-5.417019157	Down	3.04E-06	6.85E-06
TSGA10IP	1726	-2.001801333	Down	0.000354982	0.000508177
TSHZ1	4992	-1.895531948	Down	0.000235105	0.00035159
TSLP	2411	-2.165300065	Down	0.000233796	0.000349812
TSPAN14	5210	-1.39723839	Down	0.000467991	0.0006506
TTC14	4787	-1.000412182	Down	0.000561953	0.000762727
TTC22	2219	-2.431561978	Down	1.30E-07	3.68E-07
TTC39A	2848	-6.275078494	Down	3.51E-10	1.37E-09
TTC5	1866	-4.865739783	Down	0.000138382	0.000218116
TTYH1	1858	-1.32057829	Down	0.000205784	0.000312815
TUBB3	1769	-1.63775009	Down	2.44E-06	5.59E-06
TUBB6	1952	-3.59606033	Down	6.39E-12	2.96E-11
TUBGCP4	4182	-6.029830159	Down	4.09E-12	1.93E-11
TUBGCP5	3848	-1.842469324	Down	2.27E-31	2.81E-30
TULP4	8618	-7.310773735	Down	2.19E-18	1.57E-17
TWF2	1625	-1.547769702	Down	3.31E-07	8.77E-07
UBE2D3	4126	-4.714969236	Down	0.000321021	0.000463684
UBE2Q1	3223	-1.07676339	Down	6.10E-05	0.00010527
UBE2S	1191	-1.828265078	Down	3.41E-05	6.23E-05
UBE3A	5211	-2.173567681	Down	0.000560699	0.000761112
UBTD1	1693	-1.828265078	Down	7.07E-13	3.53E-12
UBTF	4689	-6.02328106	Down	8.14E-09	2.72E-08
UGT8	2898	-1.153292662	Down	1.33E-06	3.17E-06
UNC13D	4392	-1.193995498	Down	1.16E-49	2.29E-48
UNC5B	6857	-2.991532997	Down	1.29E-08	4.21E-08
UNC5C	9878	-2.383605529	Down	4.82E-08	1.45E-07
UNG	2148	-4.972654987	Down	7.26E-05	0.000122618
UPB1	2067	-3.750262566	Down	0.000617248	0.000829661
USP2	2918	-2.438064461	Down	2.89E-08	8.96E-08
USP33	4531	-4.777652564	Down	0.000228377	0.000342532
USP36	5234	-1.08901648	Down	1.07E-21	9.11E-21
USP38	7197	-3.179727135	Down	0.000207488	0.000315138
VAMP2	2155	-1.817273215	Down	2.30E-07	6.26E-07
VARS	4292	-1.068901794	Down	2.88E-82	9.67E-81
VCAN	7154	-4.624731683	Down	0.000512547	0.000702989

VCX	967	-1.266491897	Down	1.22E-24	1.18E-23
VCY	549	-8.486687048	Down	1.42E-35	1.98E-34
VN1R4	906	-1.330044827	Down	6.11E-19	4.52E-18
VPS13D	16345	-6.688862021	Down	6.70E-13	3.36E-12
VPS37A	4585	-6.209694184	Down	8.30E-10	3.11E-09
VTN	1677	-1.214209665	Down	0.000119679	0.000192149
WDR25	1990	-4.624731683	Down	0.000512547	0.000702989
WDR4	1998	-1.360437767	Down	0.00055111	0.000749138
WDR47	4353	-1.684841969	Down	3.85E-09	1.34E-08
WDTC1	4816	-5.921789672	Down	2.55E-08	7.98E-08
WEE1	3358	-5.314234169	Down	6.84E-06	1.44E-05
WNK2	6834	-3.291832471	Down	4.59E-07	1.19E-06
WRB-SH3BGR	1416	-2.476930507	Down	1.52E-06	3.60E-06
WT1	2427	-5.165300065	Down	2.03E-05	3.89E-05
WWC3	6454	-5.07219066	Down	3.83E-05	6.89E-05
XAB2	2655	-1.413496938	Down	4.18E-12	1.97E-11
XBP1	1794	-1.750262566	Down	3.10E-08	9.58E-08
XIRP1	6035	-2.184646154	Down	0.000673381	0.000898451
XIRP2	2892	-3.559579004	Down	0.000199012	0.000303228
YIF1A	965	-7.599928292	Down	4.22E-07	1.10E-06
ZAP70	2429	-2.119496375	Down	3.69E-05	6.70E-05
ZBTB17	2566	-2.277666588	Down	0.000315079	0.000456413
ZBTB20	27291	-6.688548357	Down	6.74E-13	3.37E-12
ZBTB32	2108	-1.351355333	Down	2.38E-09	8.47E-09
ZBTB4	5866	-1.436465235	Down	1.17E-08	3.85E-08
ZBTB7A	6482	-5.920187567	Down	2.60E-08	8.11E-08
ZC3H18	3727	-1.181941942	Down	1.22E-217	1.12E-215
ZCCHC7	2685	-1.87833415	Down	3.14E-07	8.36E-07
ZCWPW1	2255	-2.381126735	Down	0.000524514	0.000717586
ZFAT	4694	-6.165300065	Down	1.46E-09	5.33E-09
ZFHX3	12776	-1.471571565	Down	1.31E-10	5.33E-10
ZFYVE28	4022	-2.824483437	Down	9.00E-18	6.27E-17
ZKSCAN5	4344	-6.209256938	Down	8.34E-10	3.13E-09
ZMAT3	8993	-2.759577242	Down	4.05E-08	1.24E-07
ZMIZ2	4974	-8.317483484	Down	2.20E-32	2.80E-31
ZMYND10	1779	-1.173855309	Down	1.11E-06	2.70E-06
ZNF140	3198	-9.432086606	Down	1.01E-59	2.41E-58
ZNF143	2854	-1.352927068	Down	0.000542111	0.000738919
ZNF17	2678	-2.165300065	Down	0.000233796	0.000349812

ZNF180	4336	-4.620791685	Down	0.000522821	0.000715515
ZNF319	4221	-1.71278786	Down	7.46E-05	0.000125699
ZNF365	2695	-3.088924676	Down	4.92E-07	1.27E-06
ZNF385A	2349	-2.255777156	Down	1.85E-06	4.33E-06
ZNF385C	2596	-1.31707772	Down	3.28E-45	5.94E-44
ZNF410	2415	-7.010037209	Down	1.70E-15	1.03E-14
ZNF444	2064	-1.155752046	Down	1.22E-55	2.72E-54
ZNF500	3305	-1.962674735	Down	0.000330093	0.000475571
ZNF512	3543	-1.244530319	Down	4.28E-05	7.61E-05
ZNF518A	6626	-3.178762325	Down	1.46E-07	4.11E-07
ZNF540	3236	-4.865739783	Down	0.000138382	0.000218116
ZNF559	3071	-4.756261306	Down	0.000256894	0.000381097
ZNF568	3788	-3.191601936	Down	5.82E-05	0.000100728
ZNF570	2023	-5.413227578	Down	3.13E-06	7.04E-06
ZNF573	2257	-2.624731683	Down	8.03E-05	0.000134407
ZNF586	2220	-4.96956018	Down	7.40E-05	0.000124768
znf605	6185	-5.073161845	Down	6.80E-09	2.29E-08
ZNF618	9113	-1.865739783	Down	2.07E-05	3.96E-05
ZNF669	1722	-3.253211854	Down	1.17E-07	3.33E-07
ZNF683	1729	-1.673695321	Down	9.80E-51	1.98E-49
ZNF688	1136	-4.626042629	Down	0.000509168	0.000701379
ZNF706	2658	-1.047619078	Down	1.79E-05	3.46E-05
ZNF710	4518	-2.357945143	Down	7.65E-06	1.59E-05
ZNF746	3800	-4.569930749	Down	0.000672628	0.000897475
ZNF781	3142	-2.413227578	Down	0.00047376	0.000657454
ZNF821	1859	-2.909364598	Down	0.000606494	0.00081746
ZNF831	9403	-6.07219066	Down	4.58E-09	1.58E-08
ZNHIT3	2064	-4.332178191	Down	3.82E-08	1.17E-07
ZPBP	1223	-4.165300065	Down	4.38E-05	7.78E-05
ZSWIM4	4339	-1.701352965	Down	0.000573219	0.000775922

2.5 Supplementary Table 5

Table S5. The 218 upregulated intersecting-gene in two datasets.

Gene ID	
CMTR2	GSE22331 UP GSE92578 UP
CHRNA10	GSE22331 UP GSE92578 UP
SLC15A2	GSE22331 UP GSE92578 UP
COPS7A	GSE22331 UP GSE92578 UP
TAOK3	GSE22331 UP GSE92578 UP
BBS12	GSE22331 UP GSE92578 UP
PILRA	GSE22331 UP GSE92578 UP
JADE1	GSE22331 UP GSE92578 UP
TRAF7	GSE22331 UP GSE92578 UP

ZNF146	GSE22331 UP GSE92578 UP
SCAMP3	GSE22331 UP GSE92578 UP
ISCU	GSE22331 UP GSE92578 UP
PLEKHM3	GSE22331 UP GSE92578 UP
SUMF1	GSE22331 UP GSE92578 UP
BTBD9	GSE22331 UP GSE92578 UP
DVL1	GSE22331 UP GSE92578 UP
FBXO3	GSE22331 UP GSE92578 UP
RPL39	GSE22331 UP GSE92578 UP
CCDC14	GSE22331 UP GSE92578 UP
CEP72	GSE22331 UP GSE92578 UP
RHBG	GSE22331 UP GSE92578 UP
SP7	GSE22331 UP GSE92578 UP
FCGR2A	GSE22331 UP GSE92578 UP
FSD2	GSE22331 UP GSE92578 UP
NDRG1	GSE22331 UP GSE92578 UP
CLASP1	GSE22331 UP GSE92578 UP
AFAP1	GSE22331 UP GSE92578 UP
GLT8D1	GSE22331 UP GSE92578 UP
CHRDL2	GSE22331 UP GSE92578 UP
MYOC	GSE22331 UP GSE92578 UP
SYF2	GSE22331 UP GSE92578 UP
DHX29	GSE22331 UP GSE92578 UP
FSIP2	GSE22331 UP GSE92578 UP
MTPN	GSE22331 UP GSE92578 UP
PPP4R2	GSE22331 UP GSE92578 UP
SLC6A12	GSE22331 UP GSE92578 UP
PAK4	GSE22331 UP GSE92578 UP
FAXDC2	GSE22331 UP GSE92578 UP
TAF1C	GSE22331 UP GSE92578 UP
NOVA1	GSE22331 UP GSE92578 UP
MIA3	GSE22331 UP GSE92578 UP
KDM2B	GSE22331 UP GSE92578 UP
C17orf78	GSE22331 UP GSE92578 UP
SUCLG2	GSE22331 UP GSE92578 UP
UCP3	GSE22331 UP GSE92578 UP
CCP110	GSE22331 UP GSE92578 UP
FZD4	GSE22331 UP GSE92578 UP
CTTN	GSE22331 UP GSE92578 UP
TMEM206	GSE22331 UP GSE92578 UP
YPEL2	GSE22331 UP GSE92578 UP
RPTOR	GSE22331 UP GSE92578 UP
DFFB	GSE22331 UP GSE92578 UP
ZNF783	GSE22331 UP GSE92578 UP

TEX36	GSE22331 UP GSE92578 UP
CD4	GSE22331 UP GSE92578 UP
FBXW8	GSE22331 UP GSE92578 UP
ACACA	GSE22331 UP GSE92578 UP
ARL11	GSE22331 UP GSE92578 UP
TRMT112	GSE22331 UP GSE92578 UP
LRRC37A2	GSE22331 UP GSE92578 UP
TRAF3	GSE22331 UP GSE92578 UP
FAM135A	GSE22331 UP GSE92578 UP
GPR22	GSE22331 UP GSE92578 UP
PEX1	GSE22331 UP GSE92578 UP
MASTL	GSE22331 UP GSE92578 UP
PCLO	GSE22331 UP GSE92578 UP
SV2B	GSE22331 UP GSE92578 UP
EYA1	GSE22331 UP GSE92578 UP
SOX30	GSE22331 UP GSE92578 UP
CHL1	GSE22331 UP GSE92578 UP
ATP6AP1	GSE22331 UP GSE92578 UP
GRHPR	GSE22331 UP GSE92578 UP
GTF2H4	GSE22331 UP GSE92578 UP
CENPC	GSE22331 UP GSE92578 UP
SLC6A20	GSE22331 UP GSE92578 UP
SIGMAR1	GSE22331 UP GSE92578 UP
PHRF1	GSE22331 UP GSE92578 UP
DIRAS3	GSE22331 UP GSE92578 UP
TMEM51	GSE22331 UP GSE92578 UP
PCDH1	GSE22331 UP GSE92578 UP
BPIFA1	GSE22331 UP GSE92578 UP
GDAP2	GSE22331 UP GSE92578 UP
DMKN	GSE22331 UP GSE92578 UP
DGKI	GSE22331 UP GSE92578 UP
STIL	GSE22331 UP GSE92578 UP
BTN3A3	GSE22331 UP GSE92578 UP
NOS3	GSE22331 UP GSE92578 UP
MED9	GSE22331 UP GSE92578 UP
PHF14	GSE22331 UP GSE92578 UP
SAP30BP	GSE22331 UP GSE92578 UP
RRP12	GSE22331 UP GSE92578 UP
ZDHHC14	GSE22331 UP GSE92578 UP
DGCR6L	GSE22331 UP GSE92578 UP
GLIPR1	GSE22331 UP GSE92578 UP
COBL	GSE22331 UP GSE92578 UP
AKT1	GSE22331 UP GSE92578 UP
CIRBP	GSE22331 UP GSE92578 UP

FZD6	GSE22331 UP GSE92578 UP
STRN4	GSE22331 UP GSE92578 UP
ZNF490	GSE22331 UP GSE92578 UP
KIF21B	GSE22331 UP GSE92578 UP
SLC25A38	GSE22331 UP GSE92578 UP
LPAR1	GSE22331 UP GSE92578 UP
PRRT2	GSE22331 UP GSE92578 UP
SYTL3	GSE22331 UP GSE92578 UP
CDKAL1	GSE22331 UP GSE92578 UP
PITPNB	GSE22331 UP GSE92578 UP
GNL2	GSE22331 UP GSE92578 UP
PRR3	GSE22331 UP GSE92578 UP
KLHL2	GSE22331 UP GSE92578 UP
AES	GSE22331 UP GSE92578 UP
DCP1A	GSE22331 UP GSE92578 UP
BBS7	GSE22331 UP GSE92578 UP
A2ML1	GSE22331 UP GSE92578 UP
PRDM16	GSE22331 UP GSE92578 UP
STARD13	GSE22331 UP GSE92578 UP
RGL1	GSE22331 UP GSE92578 UP
FOCAD	GSE22331 UP GSE92578 UP
ATP1A1	GSE22331 UP GSE92578 UP
CLTCL1	GSE22331 UP GSE92578 UP
MAP3K7CL	GSE22331 UP GSE92578 UP
KCND3	GSE22331 UP GSE92578 UP
UBALD1	GSE22331 UP GSE92578 UP
KIT	GSE22331 UP GSE92578 UP
ZNF439	GSE22331 UP GSE92578 UP
FDFT1	GSE22331 UP GSE92578 UP
ZNF362	GSE22331 UP GSE92578 UP
IL4I1	GSE22331 UP GSE92578 UP
TRIM35	GSE22331 UP GSE92578 UP
PCDH15	GSE22331 UP GSE92578 UP
PIP5K1C	GSE22331 UP GSE92578 UP
ARL8A	GSE22331 UP GSE92578 UP
OLFM2	GSE22331 UP GSE92578 UP
IL19	GSE22331 UP GSE92578 UP
GLO1	GSE22331 UP GSE92578 UP
HNRNPD	GSE22331 UP GSE92578 UP
CTNNBIP1	GSE22331 UP GSE92578 UP
HMGN2	GSE22331 UP GSE92578 UP
PLCB4	GSE22331 UP GSE92578 UP
PLAT	GSE22331 UP GSE92578 UP
UBE3B	GSE22331 UP GSE92578 UP

ZNF808	GSE22331 UP GSE92578 UP
MYLK	GSE22331 UP GSE92578 UP
STEAP1B	GSE22331 UP GSE92578 UP
ANKHD1	GSE22331 UP GSE92578 UP
SLC38A3	GSE22331 UP GSE92578 UP
NLRP11	GSE22331 UP GSE92578 UP
TTI1	GSE22331 UP GSE92578 UP
ZNF415	GSE22331 UP GSE92578 UP
TFAP2A	GSE22331 UP GSE92578 UP
RNPS1	GSE22331 UP GSE92578 UP
SLC25A10	GSE22331 UP GSE92578 UP
GTF2I	GSE22331 UP GSE92578 UP
S100PBP	GSE22331 UP GSE92578 UP
PRKCH	GSE22331 UP GSE92578 UP
PREPL	GSE22331 UP GSE92578 UP
IMPDH1	GSE22331 UP GSE92578 UP
COMMD9	GSE22331 UP GSE92578 UP
MECR	GSE22331 UP GSE92578 UP
ATP6V0E2	GSE22331 UP GSE92578 UP
LRTM2	GSE22331 UP GSE92578 UP
ZNF850	GSE22331 UP GSE92578 UP
SLC30A9	GSE22331 UP GSE92578 UP
ARHGAP11A	GSE22331 UP GSE92578 UP
MOB2	GSE22331 UP GSE92578 UP
TC2N	GSE22331 UP GSE92578 UP
SQRDL	GSE22331 UP GSE92578 UP
IFT46	GSE22331 UP GSE92578 UP
ZNF506	GSE22331 UP GSE92578 UP
WFDC5	GSE22331 UP GSE92578 UP
ZDHHC16	GSE22331 UP GSE92578 UP
MRGBP	GSE22331 UP GSE92578 UP
IGSF1	GSE22331 UP GSE92578 UP
NUP153	GSE22331 UP GSE92578 UP
SLC4A3	GSE22331 UP GSE92578 UP
CYP4F8	GSE22331 UP GSE92578 UP
PPP3CC	GSE22331 UP GSE92578 UP
FAM169A	GSE22331 UP GSE92578 UP
SGSM1	GSE22331 UP GSE92578 UP
ARMC4	GSE22331 UP GSE92578 UP
ASCC2	GSE22331 UP GSE92578 UP
CYB561D2	GSE22331 UP GSE92578 UP
ADAM10	GSE22331 UP GSE92578 UP
LINGO1	GSE22331 UP GSE92578 UP
SPECC1	GSE22331 UP GSE92578 UP

MSANTD1	GSE22331 UP GSE92578 UP
DDX46	GSE22331 UP GSE92578 UP
HYDIN	GSE22331 UP GSE92578 UP
ZG16B	GSE22331 UP GSE92578 UP
RGS9BP	GSE22331 UP GSE92578 UP
FAM169B	GSE22331 UP GSE92578 UP
RANBP3	GSE22331 UP GSE92578 UP
COPS7B	GSE22331 UP GSE92578 UP
C2CD5	GSE22331 UP GSE92578 UP
NSUN2	GSE22331 UP GSE92578 UP
TBC1D4	GSE22331 UP GSE92578 UP
HPD	GSE22331 UP GSE92578 UP
KCNT2	GSE22331 UP GSE92578 UP
DAB2	GSE22331 UP GSE92578 UP
NCKAP5	GSE22331 UP GSE92578 UP
SLC36A1	GSE22331 UP GSE92578 UP
ETFDH	GSE22331 UP GSE92578 UP
KLHL8	GSE22331 UP GSE92578 UP
TMEM234	GSE22331 UP GSE92578 UP
USF1	GSE22331 UP GSE92578 UP
ST6GALNAC6	GSE22331 UP GSE92578 UP
NT5C2	GSE22331 UP GSE92578 UP
TMEM30A	GSE22331 UP GSE92578 UP
SLC6A9	GSE22331 UP GSE92578 UP
C6orf25	GSE22331 UP GSE92578 UP
C3orf35	GSE22331 UP GSE92578 UP
LPCAT3	GSE22331 UP GSE92578 UP
STAC3	GSE22331 UP GSE92578 UP
HOXD9	GSE22331 UP GSE92578 UP
CXCL16	GSE22331 UP GSE92578 UP
NDRG3	GSE22331 UP GSE92578 UP
PEPD	GSE22331 UP GSE92578 UP
ADIPOQ	GSE22331 UP GSE92578 UP

2.6 Supplementary Table 6

Table S6. The 53 downregulated intersecting-gene in two datasets.

Gene ID	
ESYT1	GSE22331 DOWN GSE92578 DOWN
DMRT3	GSE22331 DOWN GSE92578 DOWN
KLHL7	GSE22331 DOWN GSE92578 DOWN
AGTRAP	GSE22331 DOWN GSE92578 DOWN
NGFR	GSE22331 DOWN GSE92578 DOWN
DNAJB4	GSE22331 DOWN GSE92578 DOWN

PHGDH	GSE22331 DOWN GSE92578 DOWN
TOR2A	GSE22331 DOWN GSE92578 DOWN
CUL3	GSE22331 DOWN GSE92578 DOWN
TIMP1	GSE22331 DOWN GSE92578 DOWN
GNA13	GSE22331 DOWN GSE92578 DOWN
CTDP1	GSE22331 DOWN GSE92578 DOWN
MYOCD	GSE22331 DOWN GSE92578 DOWN
HLCS	GSE22331 DOWN GSE92578 DOWN
GGH	GSE22331 DOWN GSE92578 DOWN
COL5A3	GSE22331 DOWN GSE92578 DOWN
CD276	GSE22331 DOWN GSE92578 DOWN
SLC44A3	GSE22331 DOWN GSE92578 DOWN
MSH4	GSE22331 DOWN GSE92578 DOWN
ZFYVE28	GSE22331 DOWN GSE92578 DOWN
KIAA0895	GSE22331 DOWN GSE92578 DOWN
PLXNB2	GSE22331 DOWN GSE92578 DOWN
CKAP2L	GSE22331 DOWN GSE92578 DOWN
FBXL16	GSE22331 DOWN GSE92578 DOWN
HARS2	GSE22331 DOWN GSE92578 DOWN
POLD1	GSE22331 DOWN GSE92578 DOWN
DEXI	GSE22331 DOWN GSE92578 DOWN
LOXHD1	GSE22331 DOWN GSE92578 DOWN
SYNM	GSE22331 DOWN GSE92578 DOWN
ZNF17	GSE22331 DOWN GSE92578 DOWN
OR8B2	GSE22331 DOWN GSE92578 DOWN
CDH13	GSE22331 DOWN GSE92578 DOWN
MPPE1	GSE22331 DOWN GSE92578 DOWN
CLCN6	GSE22331 DOWN GSE92578 DOWN
NEDD4	GSE22331 DOWN GSE92578 DOWN
ANKRD35	GSE22331 DOWN GSE92578 DOWN
RBM4	GSE22331 DOWN GSE92578 DOWN
ARF1	GSE22331 DOWN GSE92578 DOWN
ABTB2	GSE22331 DOWN GSE92578 DOWN
GLB1L2	GSE22331 DOWN GSE92578 DOWN
ELN	GSE22331 DOWN GSE92578 DOWN
RSPO4	GSE22331 DOWN GSE92578 DOWN
C6orf99	GSE22331 DOWN GSE92578 DOWN
SPTBN5	GSE22331 DOWN GSE92578 DOWN
SEMA4B	GSE22331 DOWN GSE92578 DOWN
CHKA	GSE22331 DOWN GSE92578 DOWN
MLLT1	GSE22331 DOWN GSE92578 DOWN
HDX	GSE22331 DOWN GSE92578 DOWN
SPOCK2	GSE22331 DOWN GSE92578 DOWN
EIF3C	GSE22331 DOWN GSE92578 DOWN

CECR6	GSE22331 DOWN GSE92578 DOWN	
CERCAM	GSE22331 DOWN GSE92578 DOWN	
PYGM	GSE22331 DOWN GSE92578 DOWN	

2.7 Supplementary Table 7

Table S7. Construction of Protein-protein interaction (PPI) network.

#node1	node2	coexpression	automated_textmining	combined_score
ABTB2	CUL3	0	0.199	0.55
ABTB2	NDRG1	0.064	0.5	0.512
ACACA	UCP3	0.049	0.404	0.409
ACACA	HLCS	0.492	0.792	0.99
ACACA	ADIPOQ	0.065	0.553	0.564
ACACA	SUCLG2	0.134	0.214	0.852
ACACA	AKT1	0	0.46	0.46
ACACA	C17orf78	0	0.414	0.414
ADAM10	TIMP1	0	0.816	0.817
ADAM10	AKT1	0.062	0.484	0.495
ADAM10	CXCL16	0	0.536	0.536
ADIPOQ	CD4	0	0.456	0.456
ADIPOQ	TIMP1	0	0.469	0.469
ADIPOQ	CDH13	0.065	0.989	0.992
ADIPOQ	MED9	0	0	0.9
ADIPOQ	PRDM16	0.062	0.651	0.658
ADIPOQ	NOS3	0.062	0.681	0.687
ADIPOQ	UCP3	0.064	0.57	0.581
ADIPOQ	TBC1D4	0	0.458	0.457
ADIPOQ	SP7	0	0.417	0.417
ADIPOQ	ACACA	0.065	0.553	0.564
ADIPOQ	AKT1	0	0.692	0.692
AFAP1	MYOC	0	0.417	0.417
AFAP1	CTTN	0.077	0.692	0.704
AGTRAP	CLCN6	0	0.569	0.82
AKT1	CD4	0.049	0.69	0.697
AKT1	PYGM	0.062	0.442	0.467
AKT1	NGFR	0	0.586	0.593
AKT1	TIMP1	0	0.603	0.61
AKT1	ADAM10	0.062	0.484	0.495
AKT1	CUL3	0.1	0.481	0.546
AKT1	CHKA	0.062	0.463	0.525
AKT1	SIGMAR1	0	0.692	0.692
AKT1	KIT	0	0.736	0.758
AKT1	NOS3	0.049	0.988	0.999
AKT1	RPTOR	0.098	0.923	0.994

AKT1	DAB2	0.062	0.51	0.558
AKT1	TRAF7	0.096	0.423	0.512
AKT1	CD276	0	0.458	0.457
AKT1	UCP3	0.063	0.425	0.438
AKT1	ELN	0.049	0.475	0.479
AKT1	PHGDH	0	0.408	0.431
AKT1	LPAR1	0	0.459	0.467
AKT1	CTTN	0	0.837	0.842
AKT1	TBC1D4	0.049	0.844	0.987
AKT1	DVL1	0.098	0.927	0.939
AKT1	ADIPOQ	0	0.692	0.692
AKT1	SEMA4B	0.062	0.375	0.401
AKT1	GNA13	0	0.336	0.417
AKT1	MYOCD	0	0.4	0.4
AKT1	NDRG1	0.089	0.508	0.532
AKT1	PLEKHM3	0	0.418	0.418
AKT1	SUCLG2	0.064	0.46	0.472
AKT1	NEDD4	0.065	0.502	0.644
AKT1	TMEM206	0	0.474	0.474
AKT1	ARF1	0.063	0.395	0.47
AKT1	SP7	0.055	0.684	0.697
AKT1	ATP1A1	0.083	0.157	0.407
AKT1	GTF2I	0	0.454	0.454
AKT1	ACACA	0	0.46	0.46
AKT1	PIP5K1C	0.078	0.249	0.467
AKT1	TRAF3	0.089	0.468	0.526
ANKHD1	NUP153	0.175	0.303	0.4
ARF1	CD4	0	0.167	0.913
ARF1	SCAMP3	0.107	0.432	0.48
ARF1	SPTBN5	0.062	0.114	0.652
ARF1	CTTN	0.061	0.426	0.438
ARF1	CLTCL1	0.066	0.346	0.455
ARF1	AKT1	0.063	0.395	0.47
ARHGAP11A	CKAP2L	0.434	0	0.434
ARL8A	PLEKHM3	0	0.432	0.432
ARL8A	SUMF1	0	0.62	0.62
ARMC4	HYDIN	0.063	0.683	0.69
ATP1A1	PRKCH	0.062	0.45	0.462
ATP1A1	NDRG1	0.07	0.178	0.411
ATP1A1	AKT1	0.083	0.157	0.407
ATP6AP1	PLXNB2	0.07	0	0.541
ATP6AP1	ATP6V0E2	0.092	0.582	0.963
ATP6V0E2	RPTOR	0	0	0.6

ATP6V0E2	ATP6AP1	0.092	0.582	0.963
BBS12	BBS7	0.062	0.956	0.998
BBS7	IFT46	0.064	0.628	0.637
BBS7	BBS12	0.062	0.956	0.998
BTBD9	CUL3	0.062	0.646	0.656
BTBD9	GLO1	0	0.569	0.569
C17orf78	ACACA	0	0.414	0.414
CCDC14	CEP72	0.062	0.649	0.74
CCDC14	MYLK	0	0.412	0.412
CCDC14	STIL	0.076	0.498	0.516
CCDC14	CCP110	0.084	0.479	0.599
CCP110	STIL	0.097	0.654	0.674
CCP110	CCDC14	0.084	0.479	0.599
CD276	CD4	0	0.832	0.832
CD276	FCGR2A	0	0.464	0.463
CD276	AKT1	0	0.458	0.457
CD4	IL19	0	0.402	0.402
CD4	TRAF3	0	0.421	0.42
CD4	ELN	0	0.433	0.433
CD4	ADIPOQ	0	0.456	0.456
CD4	TIMP1	0	0.458	0.457
CD4	CXCL16	0.07	0.46	0.476
CD4	AKT1	0.049	0.69	0.697
CD4	KIT	0	0.745	0.745
CD4	FCGR2A	0.14	0.813	0.832
CD4	CD276	0	0.832	0.832
CD4	ARF1	0	0.167	0.913
CDH13	ADIPOQ	0.065	0.989	0.992
CDKAL1	PEPD	0.122	0.48	0.542
CDKAL1	NSUN2	0.135	0.323	0.41
CDKAL1	CMTR2	0	0.64	0.64
CEP72	CCDC14	0.062	0.649	0.74
CERCAM	GGH	0	0	0.493
CERCAM	SLC6A12	0	0.458	0.457
CHKA	LPCAT3	0.062	0.411	0.424
CHKA	PHGDH	0.063	0.422	0.445
CHKA	AKT1	0.062	0.463	0.525
CHL1	NGFR	0	0.47	0.47
CHL1	LINGO1	0.108	0.38	0.46
CKAP2L	ARHGAP11A	0.434	0	0.434
CLCN6	AGTRAP	0	0.569	0.82
CLTCL1	DGCR6L	0	0.576	0.576
CLTCL1	DAB2	0.065	0.329	0.727

CLTCL1	UBE3B	0.064	0.459	0.482
CLTCL1	FZD4	0	0.042	0.6
CLTCL1	ARF1	0.066	0.346	0.455
CMTR2	NSUN2	0.086	0.483	0.507
CMTR2	CDKAL1	0	0.64	0.64
CMTR2	GDAP2	0.063	0.508	0.52
COL5A3	TBC1D4	0	0.433	0.433
COL5A3	OLFM2	0.153	0.624	0.668
COMMD9	KLHL2	0	0	0.6
COMMD9	CUL3	0.061	0	0.608
COMMD9	COPS7A	0.049	0	0.9
COMMD9	COPS7B	0.069	0	0.902
COPS7A	COMMD9	0.049	0	0.9
COPS7A	CUL3	0.058	0.556	0.886
COPS7A	COPS7B	0	0.717	0.967
COPS7B	COMMD9	0.069	0	0.902
COPS7B	CUL3	0.102	0.357	0.668
COPS7B	COPS7A	0	0.717	0.967
CTDP1	MLLT1	0.098	0.198	0.842
CTDP1	GTF2H4	0.102	0	0.631
CTDP1	NT5C2	0	0.59	0.59
CTDP1	MASTL	0.062	0.601	0.61
CTTN	MYLK	0.06	0.975	0.99
CTTN	ARF1	0.061	0.426	0.438
CTTN	GNA13	0.062	0.539	0.601
CTTN	AFAP1	0.077	0.692	0.704
CTTN	AKT1	0	0.837	0.842
CTTN	PAK4	0.049	0.353	0.933
CUL3	COMMD9	0.061	0	0.608
CUL3	TRAF7	0.064	0.173	0.474
CUL3	FBXW8	0.062	0.363	0.489
CUL3	AKT1	0.1	0.481	0.546
CUL3	ABTB2	0	0.199	0.55
CUL3	NEDD4	0.064	0.527	0.558
CUL3	BTBD9	0.062	0.646	0.656
CUL3	COPS7B	0.102	0.357	0.668
CUL3	COPS7A	0.058	0.556	0.886
CUL3	DVL1	0.063	0.205	0.925
CUL3	KLHL8	0.062	0.691	0.982
CUL3	KLHL2	0.062	0.936	0.995
CUL3	KLHL7	0.065	0.979	0.998
CXCL16	CD4	0.07	0.46	0.476
CXCL16	ADAM10	0	0.536	0.536
DAB2	DVL1	0	0.195	0.425

DAB2	AKT1	0.062	0.51	0.558
DAB2	CLTCL1	0.065	0.329	0.727
DDX46	DHX29	0.148	0.35	0.496
DGCR6L	ZNF490	0	0.575	0.575
DGCR6L	CLTCL1	0	0.576	0.576
DGCR6L	PAK4	0	0.866	0.898
DGKI	PLCB4	0.099	0.201	0.768
DHX29	DDX46	0.148	0.35	0.496
DHX29	EIF3C	0.094	0.532	0.712
DNAJB4	RANBP3	0.058	0	0.416
DVL1	CUL3	0.063	0.205	0.925
DVL1	DAB2	0	0.195	0.425
DVL1	FZD6	0.06	0.729	0.979
DVL1	SUCLG2	0	0.441	0.441
DVL1	NEDD4	0	0.222	0.452
DVL1	PAK4	0.062	0	0.907
DVL1	AKT1	0.098	0.927	0.939
DVL1	FZD4	0.062	0.844	0.953
EIF3C	DHX29	0.094	0.532	0.712
EIF3C	ISCU	0.046	0.404	0.407
EIF3C	GNL2	0.554	0	0.554
EIF3C	RRP12	0.659	0	0.659
ELN	CD4	0	0.433	0.433
ELN	MYOC	0.089	0.393	0.424
ELN	TIMP1	0.062	0.71	0.716
ELN	NOS3	0.062	0.561	0.57
ELN	MYOCD	0.07	0.415	0.433
ELN	GTF2I	0	0.468	0.468
ELN	AKT1	0.049	0.475	0.479
ETFDH	SQRDL	0.062	0.509	0.582
ETFDH	UCP3	0	0.332	0.411
ETFDH	MRGBP	0	0.494	0.494
ETFDH	SUCLG2	0.164	0.324	0.495
ETFDH	KLHL2	0.063	0.391	0.405
FAM169A	YPEL2	0	0.509	0.509
FAXDC2	SIGMAR1	0.099	0.443	0.86
FAXDC2	FDFT1	0.171	0.498	0.83
FBXW8	CUL3	0.062	0.363	0.489
FCGR2A	CD4	0.14	0.813	0.832
FCGR2A	CD276	0	0.464	0.463
FCGR2A	KIT	0.062	0.607	0.616
FDFT1	SIGMAR1	0.064	0.658	0.725
FDFT1	FAXDC2	0.171	0.498	0.83
FDFT1	PHGDH	0.084	0.381	0.41

FSD2	ZNF362	0.056	0.541	0.558
FZD4	FZD6	0.062	0.848	0.914
FZD4	DVL1	0.062	0.844	0.953
FZD4	CLTCL1	0	0.042	0.6
FZD6	RSPO4	0	0.773	0.773
FZD6	FZD4	0.062	0.848	0.914
FZD6	DVL1	0.06	0.729	0.979
GDAP2	CMTR2	0.063	0.508	0.52
GGH	CERCAM	0	0	0.493
GLIPR1	NCKAP5	0	0.464	0.464
GLO1	GRHPR	0.085	0.19	0.943
GLO1	BTBD9	0	0.569	0.569
GLT8D1	SUCLG2	0	0.699	0.699
GLT8D1	SCAMP3	0	0.713	0.713
GNA13	LPAR1	0	0.439	0.948
GNA13	CTTN	0.062	0.539	0.601
GNA13	AKT1	0	0.336	0.417
GNL2	NSUN2	0.241	0.063	0.523
GNL2	EIF3C	0.554	0	0.554
GNL2	IMPDH1	0.145	0.101	0.463
GNL2	RPL39	0.064	0.294	0.933
GNL2	RRP12	0.856	0.663	0.949
GPR22	ZNF146	0	0.404	0.413
GRHPR	PYGM	0.177	0.19	0.406
GRHPR	GLO1	0.085	0.19	0.943
GTF2H4	TAF1C	0.09	0	0.62
GTF2H4	CTDP1	0.102	0	0.631
GTF2H4	POLD1	0.146	0.313	0.745
GTF2I	MSH4	0	0.421	0.421
GTF2I	ELN	0	0.468	0.468
GTF2I	USF1	0	0.963	0.993
GTF2I	HYDIN	0	0.764	0.764
GTF2I	AKT1	0	0.454	0.454
HARS2	PEPD	0.093	0.565	0.604
HLCS	ACACA	0.492	0.792	0.99
HMGN2	SYF2	0	0.523	0.523
HMGN2	SLC6A9	0	0.539	0.539
HPD	IL4I1	0	0.147	0.912
HYDIN	ARMC4	0.063	0.683	0.69
HYDIN	GTF2I	0	0.764	0.764
IFT46	BBS7	0.064	0.628	0.637
IGSF1	LINGO1	0.065	0.46	0.473
IL19	CD4	0	0.402	0.402
IL4I1	HPD	0	0.147	0.912

IMPDH1	NT5C2	0	0.188	0.915
IMPDH1	KLHL7	0.062	0.518	0.528
IMPDH1	GNL2	0.145	0.101	0.463
IMPDH1	RRP12	0.444	0.176	0.522
ISCU	EIF3C	0.046	0.404	0.407
JADE1	TMEM51	0	0.415	0.415
JADE1	TMEM206	0	0.723	0.723
KCND3	KCNT2	0.086	0.4	0.428
KCNT2	KCND3	0.086	0.4	0.428
KIT	CD4	0	0.745	0.745
KIT	NGFR	0	0.503	0.503
KIT	MLLT1	0	0.462	0.462
KIT	FCGR2A	0.062	0.607	0.616
KIT	NOS3	0	0.459	0.459
KIT	PLCB4	0	0.142	0.657
KIT	AKT1	0	0.736	0.758
KIT	PRRT2	0	0.066	0.902
KIT	TFAP2A	0	0.301	0.927
KLHL2	COMMD9	0	0	0.6
KLHL2	CUL3	0.062	0.936	0.995
KLHL2	KLHL8	0	0.909	0.765
KLHL2	KLHL7	0	0.326	0.734
KLHL2	ETFDH	0.063	0.391	0.405
KLHL7	CUL3	0.065	0.979	0.998
KLHL7	KLHL8	0	0	0.9
KLHL7	IMPDH1	0.062	0.518	0.528
KLHL7	KLHL2	0	0.326	0.734
KLHL8	CUL3	0.062	0.691	0.982
KLHL8	ZNF146	0	0.508	0.518
KLHL8	KLHL2	0	0.909	0.765
KLHL8	KLHL7	0	0	0.9
LINGO1	MYOC	0	0.707	0.711
LINGO1	NGFR	0.098	0.988	0.999
LINGO1	CHL1	0.108	0.38	0.46
LINGO1	SEMA4B	0	0.423	0.422
LINGO1	IGSF1	0.065	0.46	0.473
LOXHD1	PLAT	0	0.727	0.727
LOXHD1	PCDH15	0	0.458	0.457
LPAR1	AKT1	0	0.459	0.467
LPAR1	GNA13	0	0.439	0.948
LPCAT3	CHKA	0.062	0.411	0.424
MASTL	CTDP1	0.062	0.601	0.61
MED9	ADIPOQ	0	0	0.9
MLLT1	KIT	0	0.462	0.462

MLLT1	MRGBP	0.087	0.37	0.509
MLLT1	CTDP1	0.098	0.198	0.842
MRGBP	MLLT1	0.087	0.37	0.509
MRGBP	ETFDH	0	0.494	0.494
MSH4	GTF2I	0	0.421	0.421
MSH4	POLD1	0.138	0.381	0.525
MYLK	NOS3	0	0.45	0.45
MYLK	STARD13	0.092	0.396	0.428
MYLK	CCDC14	0	0.412	0.412
MYLK	MYOCD	0.096	0.466	0.496
MYLK	PAK4	0	0.267	0.638
MYLK	CTTN	0.06	0.975	0.99
MYOC	AFAP1	0	0.417	0.417
MYOC	ELN	0.089	0.393	0.424
MYOC	LINGO1	0	0.707	0.711
MYOCD	ELN	0.07	0.415	0.433
MYOCD	MYLK	0.096	0.466	0.496
MYOCD	AKT1	0	0.4	0.4
NCKAP5	GLIPR1	0	0.464	0.464
NDRG1	ATP1A1	0.07	0.178	0.411
NDRG1	ABTB2	0.064	0.5	0.512
NDRG1	AKT1	0.089	0.508	0.532
NEDD4	CUL3	0.064	0.527	0.558
NEDD4	TRAF7	0.062	0.354	0.402
NEDD4	DVL1	0	0.222	0.452
NEDD4	PIP5K1C	0.063	0.425	0.573
NEDD4	AKT1	0.065	0.502	0.644
NGFR	CHL1	0	0.47	0.47
NGFR	KIT	0	0.503	0.503
NGFR	AKT1	0	0.586	0.593
NGFR	PLCB4	0	0	0.6
NGFR	LINGO1	0.098	0.988	0.999
NOS3	TIMP1	0	0.469	0.469
NOS3	PLAT	0	0.458	0.457
NOS3	KIT	0	0.459	0.459
NOS3	MYLK	0	0.45	0.45
NOS3	ELN	0.062	0.561	0.57
NOS3	ADIPOQ	0.062	0.681	0.687
NOS3	AKT1	0.049	0.988	0.999
NOVA1	RBM4	0.062	0.501	0.512
NSUN2	PAK4	0	0.41	0.41
NSUN2	CDKAL1	0.135	0.323	0.41
NSUN2	CMTR2	0.086	0.483	0.507
NSUN2	GNL2	0.241	0.063	0.523

NSUN2	TRMT112	0.088	0.66	0.704
NSUN2	RRP12	0.852	0.323	0.895
NT5C2	CTDP1	0	0.59	0.59
NT5C2	IMPDH1	0	0.188	0.915
NUP153	RANBP3	0.086	0.661	0.714
NUP153	ANKHD1	0.175	0.303	0.4
NUP153	PHGDH	0	0.506	0.506
NUP153	RNPS1	0.091	0.063	0.629
OLFM2	COL5A3	0.153	0.624	0.668
PAK4	DGCR6L	0	0.866	0.898
PAK4	NSUN2	0	0.41	0.41
PAK4	MYLK	0	0.267	0.638
PAK4	CTTN	0.049	0.353	0.933
PAK4	DVL1	0.062	0	0.907
PCDH15	LOXHD1	0	0.458	0.457
PEPD	HARS2	0.093	0.565	0.604
PEPD	CDKAL1	0.122	0.48	0.542
PEPD	PHGDH	0.064	0.124	0.813
PHGDH	PEPD	0.064	0.124	0.813
PHGDH	CHKA	0.063	0.422	0.445
PHGDH	FDFT1	0.084	0.381	0.41
PHGDH	AKT1	0	0.408	0.431
PHGDH	SLC6A12	0	0.467	0.467
PHGDH	NUP153	0	0.506	0.506
PIP5K1C	PLCB4	0	0.261	0.929
PIP5K1C	NEDD4	0.063	0.425	0.573
PIP5K1C	AKT1	0.078	0.249	0.467
PLAT	TIMP1	0.106	0.556	0.586
PLAT	NOS3	0	0.458	0.457
PLAT	LOXHD1	0	0.727	0.727
PLCB4	NGFR	0	0	0.6
PLCB4	KIT	0	0.142	0.657
PLCB4	DGKI	0.099	0.201	0.768
PLCB4	PPP3CC	0.064	0.171	0.669
PLCB4	PIP5K1C	0	0.261	0.929
PLEKHM3	ARL8A	0	0.432	0.432
PLEKHM3	AKT1	0	0.418	0.418
PLXNB2	ATP6AP1	0.07	0	0.541
PLXNB2	SEMA4B	0.086	0.861	0.808
POLD1	GTF2H4	0.146	0.313	0.745
POLD1	MSH4	0.138	0.381	0.525
PPP3CC	PPP4R2	0.062	0.107	0.636
PPP3CC	PLCB4	0.064	0.171	0.669
PPP4R2	PPP3CC	0.062	0.107	0.636

PRDM16	UCP3	0	0.555	0.555
PRDM16	ADIPOQ	0.062	0.651	0.658
PREPL	SQRDL	0	0.47	0.47
PREPL	PRKCH	0	0.445	0.445
PRKCH	PREPL	0	0.445	0.445
PRKCH	ATP1A1	0.062	0.45	0.462
PRRT2	KIT	0	0.066	0.902
PYGM	GRHPR	0.177	0.19	0.406
PYGM	AKT1	0.062	0.442	0.467
RANBP3	DNAJB4	0.058	0	0.416
RANBP3	NUP153	0.086	0.661	0.714
RBM4	NOVA1	0.062	0.501	0.512
RGS9BP	SUCLG2	0	0.625	0.625
RNPS1	NUP153	0.091	0.063	0.629
RNPS1	RRP12	0	0.523	0.523
RPL39	GNL2	0.064	0.294	0.933
RPTOR	ATP6V0E2	0	0	0.6
RPTOR	TTI1	0.057	0.845	0.988
RPTOR	AKT1	0.098	0.923	0.994
RRP12	NSUN2	0.852	0.323	0.895
RRP12	EIF3C	0.659	0	0.659
RRP12	IMPDH1	0.444	0.176	0.522
RRP12	GNL2	0.856	0.663	0.949
RRP12	ZDHHC16	0.062	0.415	0.428
RRP12	RNPS1	0	0.523	0.523
RSPO4	FZD6	0	0.773	0.773
S100PBP	ZG16B	0	0.608	0.608
SCAMP3	GLT8D1	0	0.713	0.713
SCAMP3	ARF1	0.107	0.432	0.48
SCAMP3	SUCLG2	0.049	0.569	0.573
SEMA4B	LINGO1	0	0.423	0.422
SEMA4B	AKT1	0.062	0.375	0.401
SEMA4B	PLXNB2	0.086	0.861	0.808
SIGMAR1	ZDHHC16	0.065	0.439	0.453
SIGMAR1	AKT1	0	0.692	0.692
SIGMAR1	FDFT1	0.064	0.658	0.725
SIGMAR1	FAXDC2	0.099	0.443	0.86
SLC15A2	SLC36A1	0.064	0.423	0.449
SLC36A1	SLC15A2	0.064	0.423	0.449
SLC36A1	SLC38A3	0.062	0.434	0.458
SLC36A1	SLC6A20	0.062	0.46	0.472
SLC38A3	SLC36A1	0.062	0.434	0.458
SLC6A12	PHGDH	0	0.467	0.467

SLC6A12	CERCAM	0	0.458	0.457
SLC6A20	SLC36A1	0.062	0.46	0.472
SLC6A9	HMGN2	0	0.539	0.539
SP7	ADIPOQ	0	0.417	0.417
SP7	AKT1	0.055	0.684	0.697
SPTBN5	ARF1	0.062	0.114	0.652
SQRDL	PREPL	0	0.47	0.47
SQRDL	ETFDH	0.062	0.509	0.582
STARD13	MYLK	0.092	0.396	0.428
STIL	CCDC14	0.076	0.498	0.516
STIL	CCP110	0.097	0.654	0.674
SUCLG2	GLT8D1	0	0.699	0.699
SUCLG2	SCAMP3	0.049	0.569	0.573
SUCLG2	RGS9BP	0	0.625	0.625
SUCLG2	DVL1	0	0.441	0.441
SUCLG2	AKT1	0.064	0.46	0.472
SUCLG2	ETFDH	0.164	0.324	0.495
SUCLG2	ACACA	0.134	0.214	0.852
SUMF1	ARL8A	0	0.62	0.62
SYF2	HMGN2	0	0.523	0.523
TAF1C	GTF2H4	0.09	0	0.62
TBC1D4	COL5A3	0	0.433	0.433
TBC1D4	ADIPOQ	0	0.458	0.457
TBC1D4	AKT1	0.049	0.844	0.987
TFAP2A	KIT	0	0.301	0.927
TIMP1	CD4	0	0.458	0.457
TIMP1	ADIPOQ	0	0.469	0.469
TIMP1	NOS3	0	0.469	0.469
TIMP1	PLAT	0.106	0.556	0.586
TIMP1	AKT1	0	0.603	0.61
TIMP1	ELN	0.062	0.71	0.716
TIMP1	ADAM10	0	0.816	0.817
TMEM206	JADE1	0	0.723	0.723
TMEM206	AKT1	0	0.474	0.474
TMEM234	ZFYVE28	0	0.403	0.403
TMEM30A	TRMT112	0	0.462	0.461
TMEM51	JADE1	0	0.415	0.415
TRAF3	CD4	0	0.421	0.42
TRAF3	AKT1	0.089	0.468	0.526
TRAF7	CUL3	0.064	0.173	0.474
TRAF7	NEDD4	0.062	0.354	0.402
TRAF7	AKT1	0.096	0.423	0.512
TRMT112	TMEM30A	0	0.462	0.461

TRMT112	NSUN2	0.088	0.66	0.704
TTI1	RPTOR	0.057	0.845	0.988
UBE3B	CLTCL1	0.064	0.459	0.482
UCP3	PRDM16	0	0.555	0.555
UCP3	ACACA	0.049	0.404	0.409
UCP3	ETFDH	0	0.332	0.411
UCP3	AKT1	0.063	0.425	0.438
UCP3	ADIPOQ	0.064	0.57	0.581
USF1	GTF2I	0	0.963	0.993
YPEL2	FAM169A	0	0.509	0.509
ZDHHC16	SIGMAR1	0.065	0.439	0.453
ZDHHC16	RRP12	0.062	0.415	0.428
ZFYVE28	TMEM234	0	0.403	0.403
ZG16B	S100PBP	0	0.608	0.608
ZNF146	KLHL8	0	0.508	0.518
ZNF146	GPR22	0	0.404	0.413
ZNF362	FSD2	0.056	0.541	0.558
ZNF490	DGCR6L	0	0.575	0.575

2.8 Supplementary Table 8

Table S8. The details of miRNA-TFs-genes network

Gene	TF	miRNA	Gene	TF	miRNA
COPS7A	MAX	hsa-miR-103	CUL3	CDC5L	hsa-miR-101
COPS7A	MXI1::CLEC5A	hsa-miR-107	CUL3	ELK1	hsa-miR-106a
COPS7A	MYC	hsa-miR-135a	CUL3	GABPA	hsa-miR-106b
COPS7A	NFE2L2	hsa-miR-135b	CUL3	MAX	hsa-miR-130a
COPS7A	SPI1	hsa-miR-142-3p	CUL3	MEF2A	hsa-miR-130b
COPS7A	TP53	hsa-miR-15a	CUL3	MYC	hsa-miR-137
COPS7A	USF1	hsa-miR-15b	CUL3	PATZ1	hsa-miR-139-5p
COPS7A	ARNT	hsa-miR-16	CUL3	PAX2	hsa-miR-140
COPS7A	COPS2	hsa-miR-186	CUL3	REL	hsa-miR-140-5p
COPS7A	COPS5	hsa-miR-191	CUL3	SP1	hsa-miR-141
COPS7A	EGR1	hsa-miR-195	CUL3	SPI1	hsa-miR-144
COPS7A	GABPA	hsa-miR-215	CUL3	TBP	hsa-miR-17-5p
COPS7A	GATA1	hsa-miR-326	CUL3	YY1	hsa-miR-181a
COPS7A	-	hsa-miR-337-5p	CUL3	-	hsa-miR-181b
COPS7A	-	hsa-miR-424	CUL3	-	hsa-miR-181c
COPS7A	-	hsa-miR-497	CUL3	-	hsa-miR-181d
COPS7A	-	hsa-miR-516a-3p	CUL3	-	hsa-miR-192
COPS7A	-	hsa-miR-570	CUL3	-	hsa-miR-193a-5p
COPS7A	-	hsa-miR-611	CUL3	-	hsa-miR-200a
COPS7A	-	hsa-miR-614	CUL3	-	hsa-miR-20a
COPS7A	-	hsa-miR-637	CUL3	-	hsa-miR-216a

COPS7A	-	hsa-miR-760	CUL3	-	hsa-miR-218
COPS7A	-	hsa-miR-885-3p	CUL3	-	hsa-miR-22
COPS7A	-	hsa-miR-892b	CUL3	-	hsa-miR-23a
NEDD4	CREB1	hsa-let-7b	CUL3	-	hsa-miR-23b
NEDD4	CTCF	hsa-miR-27a	CUL3	-	hsa-miR-301
NEDD4	DAZAP2	hsa-miR-27b	CUL3	-	hsa-miR-301a
NEDD4	E2F1	hsa-miR-30a	CUL3	-	hsa-miR-301b
NEDD4	EGR1	hsa-miR-30a-5p	CUL3	-	hsa-miR-302a
NEDD4	FOS	hsa-miR-30b	CUL3	-	hsa-miR-302b
NEDD4	FOXD3	hsa-miR-30c	CUL3	-	hsa-miR-302c
NEDD4	FOXF2	hsa-miR-30d	CUL3	-	hsa-miR-302d
NEDD4	FOXL1	hsa-miR-30e	CUL3	-	hsa-miR-32
NEDD4	GATA1	hsa-miR-495	CUL3	-	hsa-miR-363
NEDD4	LMO2	hsa-miR-9	CUL3	-	hsa-miR-367
NEDD4	NFE2	-	CUL3	-	hsa-miR-372
NEDD4	NKX2-2	-	CUL3	-	hsa-miR-410
NEDD4	PAX2	-	CUL3	-	hsa-miR-421
NEDD4	POU2F1	-	CUL3	-	hsa-miR-450b-5p
NEDD4	POU2F2	-	CUL3	-	hsa-miR-454
NEDD4	POU3F1	-	CUL3	-	hsa-miR-455-3p
NEDD4	POU3F2	-	CUL3	-	hsa-miR-494
NEDD4	POU3F3	-	CUL3	-	hsa-miR-519a
NEDD4	POU5F1	-	CUL3	-	hsa-miR-519b-3p
NEDD4	TBP	-	CUL3	-	hsa-miR-519c-3p
NEDD4	TFAP2C	-	CUL3	-	hsa-miR-520f
NEDD4	TP73	-	CUL3	-	hsa-miR-570
KLHL7	CEBPB	hsa-miR-105	CUL3	-	hsa-miR-576-5p
KLHL7	CTCF	hsa-miR-1271	CUL3	-	hsa-miR-577
KLHL7	IRF7	hsa-miR-455-5p	CUL3	-	hsa-miR-590-3p
KLHL7	-	hsa-miR-501-5p	CUL3	-	hsa-miR-92
KLHL7	-	hsa-miR-562	CUL3	-	hsa-miR-93
KLHL7	-	hsa-miR-577	CUL3	-	hsa-miR-944
KLHL7	-	hsa-miR-610			
KLHL7	-	hsa-miR-921			
KLHL7	-	hsa-miR-96			

TF, transcription factors.
